# Supplementary material for: Synthesis of dienes from pyrrolidines using skeletal modification
Source: Nat Commun. 2023 Nov 11;14:7307. doi: 10.1038/s41467-023-43238-7 (PMC10640553; doi:10.1038/s41467-023-43238-7)
Supplement: Supplementary file 1 — Supplementary Information [file 41467_2023_43238_MOESM1_ESM.pdf]

Supplementary Information

Table of Contents

General Information 2

Reaction Optimization 3

General Procedure for the Synthesis of Dienes from Pyrrolidines 5

General C−H Functionalization Procedure for the Synthesis of *o*-Substituted Pyrrolidines 7

Synthetic Applications 8

Gram Scale Synthesis of Diene **45b** 8

Synthesis of N-deletion Product **45c** by Photocyclization of Diene **45b** 9

Mechanistic studies 10

Intermolecular Radical Trapping Experiments 10

Cyclopropane as a Radical Probe Opening Experiment 14

Oxidation Reactions with *N*-Aminopyrrolidine (**45d**) 16

The Observation of N-atom Deletion Side Products 19

Characterization Data 25

Copies of NMR Spectra 75

Supplementary References 166

General Information

All reactions were carried out in anhydrous solvents and performed under ambient conditions unless otherwise noted. All commercial reagents and solvents were used without further purification unless otherwise mentioned. Thin layer chromatography (TLC) was performed on pre-coated silica gel GF254 plates. Visualization of TLC was achieved using UV light (wavelength 254 nm), Phosphomolybdic acid hydrate ($H_3PO_4 \cdot 12MoO_3$), I_2 (dispersed in silica), and/or $KMnO_4$ stain. Column chromatography was performed on silica gel (300-400 mesh) using a proper eluent. NMR was recorded on a FT AM 400 (400 MHz). Chemical shifts were reported in parts per million (ppm) referenced to the appropriate solvent peaks (δ 7.26 ppm for $CDCl_3$, δ 2.50 ppm for DMSO-*d*6 in proton spectra, 77.0 ppm for $CDCl_3$, δ 40.0 ppm for DMSO-*d*6 in fully decoupled ^{13}C spectra). The following abbreviations were used to describe peak splitting patterns: br = broad, s = singlet, d = doublet, t = triplet, q = quartet, dd = doublet of doublet, td = triplet of doublet, ddd = doublet of doublet of doublet, m = multiplet. Coupling constants *J* were reported in hertz (Hz). High resolution mass spectra were obtained with the UHD Accurate-Mass Q-TOF in Nanjing University with ESI when available. For compounds that failed with ESI due to low polarity, EI spectra were obtained with another UHD Accurate-Mass GCT-TOF from Analysis and Testing Center of Soochow University.

Compounds **1a**, **11a**, **23a**, **33a**, **35a** are known compounds seen in reference.[1],[2],[3] Compounds **16a** and **19a** was used as purchased.

Reaction Optimization

Supplementary Figure 1. Reaction Optimization-1

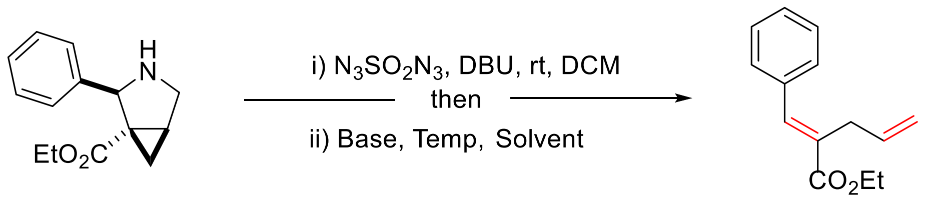

1a **1b**

entry	base	solvent	temp	yield[a]
1	^{t}BuOK	Dioxane	120 °C	44%
2	^{t}BuONa	Dioxane	120 °C	36%
3	^{t}BuOLi	Dioxane	120 °C	70%
4	Cs_2CO_3	Dioxane	120 °C	35%
5	CsOAc	Dioxane	120 °C	65%
6	K_3PO_4	Dioxane	120 °C	61%
7	KF	Dioxane	120 °C	66%
8	DBU	Dioxane	120 °C	41%
9	^{t}BuOLi	PhCl	120 °C	71%
10	^{t}BuOLi	THF	120 °C	trace
11	^{t}BuOLi	MeCN	120 °C	64%
12	^{t}BuOLi	DMF	120 °C	51
13	^{t}BuOLi	DCE	120 °C	81%
14	^{t}BuOLi	DCE	100 °C	44%
15	DBU	DCE	120 °C	41%
16[b]	^{t}BuOLi	DCE	120 °C	trace
17[c]	^{t}BuOLi	DCE	120 °C	trace

Reaction conditions: **1a** (0.2 mmol), $N_2SO_2N_3$ (0.2 M in DCM, 3 mL), DBU (0.4 mmol), DCM (0.5 mL), 0 °C - rt, 3 h; then base (0.2 mmol), solvent (2.0 mL), temp, Ar, 3 h, Schleck Tube. [a] ^{1}H-NMR yield was provided with CH_2Br_2 as internal standard. [b]using condenser tube. [c]using condenser tube and with powdered molecular sieve as additive.

Supplementary Figure 2. Reaction Optimization-2

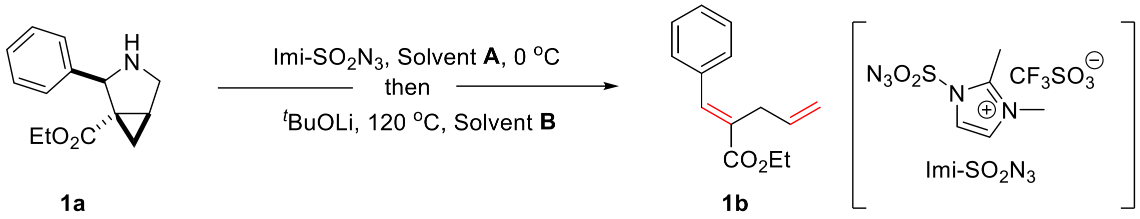

entry	Solvent **A**	Solvent **B**	temp	yield[a]
1[b, c, d, e]	CH_3CN	Dioxane	80 °C	trace
2[b, c, d]	CH_3CN	Dioxane	80 °C	trace
3[b, c, d]	Dioxane	Dioxane	120 °C	trace
4[b, c, d]	Dioxane	DCE	120 °C	trace
5[c, f]	CH_3CN	Dioxane	120 °C	67%
6	CH_3CN	Dioxane	120 °C	70%
7	CH_3CN	DCE	120 °C	94% (89%)
8	CH_3CN	DCE	100 °C	57%
9[d]	CH_3CN	CH_3CN	120 °C	50%
10[d, h]	CH_3CN	DCE	120 °C	59%
11[d, i]	CH_3CN+DCE	-----	120 °C	69%
12[d, j]	DCE	-----	120 °C	93% (80%)
13[i, k]	CH_3CN+DCE	-----	120 °C	92% (75%)

Reaction conditions: **1a** (0.2 mmol), N_3 reagent (0.21 mmol), solvent (2.0 mL), 0 °C, 3 h; then ^{t}BuOLi (0.2 mmol), solvent (2.0 mL), temp, Ar, 3 h, schleck tube. [a] ^{1}H-NMR yield was provided with CH_2Br_2 as internal standard. [b]using condenser tube. [c]with powdered molecular sieve. [d]without further treatment at the end of the first step reaction. [e]without ^{t}BuOLi. [f]removing the solvent at the end of the first step reaction. [h]0.2 mL CH_3CN as solvent **A**, 2.0 mL DCE as solvent **B** and without further treatment at the end of the first step reaction. [i]0.2 mL CH_3CN and 2.0 mL DCE as solvent **A**. [j]2.0 mL DCE as solvent **A**. [k] ^{t}BuOLi was added at the same time.

General Procedure for the Synthesis of Dienes from Pyrrolidines

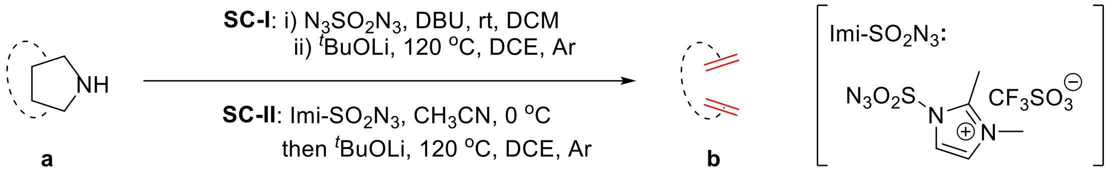

SC-I: To a solution of $N_3SO_2N_3$ (3 mL, 3.0 equiv, ~ 0.2mol/L in DCM) at 0 °C, the mixture of pyrrolidine **a** (or corresponding amine hydrochloride **a**•HCl) (0.2 mmol, 1.0 equiv) and DBU (0.4 mmol, 2.0 equiv; 0.6 mmol, 3.0 equiv if using **a**•HCl as the substrate) in the solvent of DCM (0.5 mL) was added dropwise. After the reaction was completed based on TLC (~ 3 h). The solvent was removed under reduced pressure. The residue was purified by quick flash column chromatography to provide the crude sulfamoyl azide, which was then added to a reaction vessel filled with ^{t}BuOLi (1.0 equiv, relative to sulfamoyl azide) and DCE (2 mL, 0.1 M) under argon atmosphere. After the mixture was stirred at 120 °C for 3 h with heating module, the reaction was cooled to room temperature and the solvent was removed under reduced pressure. The crude mixture was purified by column chromatography to provide desired ring opening rearrangement product **b.**

SC-II: To a solution of 1-(azidosulfonyl)-2,3-dimethyl-1H-imidazol-3-ium trifluoromethylsulfonate (0.21 mmol, 1.05 equiv) in the solvent of CH_3CN (2.0 mL) was added pyrrolidine **a** (or corresponding amine hydrochloride **a**•HCl and TEA (0.2 mmol, 1.0 equiv)) (0.2 mmol, 1.0 equiv) at 0 °C under argon atmosphere. After the reaction was completed based on TLC (~ 3 h). Most of the solvent in the schleck tube is removed in vacuo. Then ^{t}BuOLi (1.0 equiv, relative to pyrrolidine **a**) and DCE (2 mL, 0.1 M) was added into the schleck tube under argon atmosphere. After the mixture was stirred at 120 °C for 3 h with heating module, the reaction was cooled to room temperature and the solvent was removed under reduced pressure. The crude

mixture was purified by column chromatography to provide desired ring opening rearrangement product **b.**

General C−H Functionalization Procedure for the Synthesis of *o*-Substituted Pyrrolidines

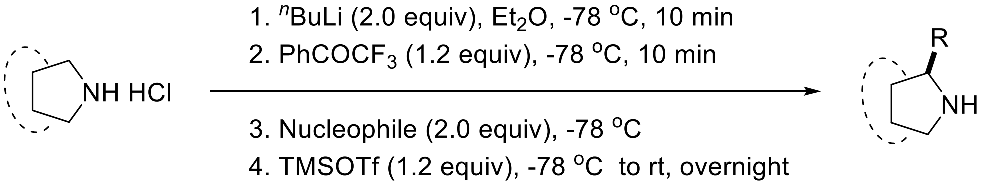

The synthesis was according to a slightly modified version of Seidel's protocol.[4] To a solution of pyrrolidine (1.0 equiv) in anhydrous ether (0.5 M) cooled to –78 °C was added n-BuLi in hexane (2.5 M, 2.0 equiv) over 10 minutes (syringe pump) under N_2 atmosphere, the resulting solution was stirred at the same temperature for another 10 minutes. To this was then added a solution of trifluoroacetophenone (1.2 equiv) in anhydrous ether (1.2 M) over 10 minutes. The resulting mixture was stirred at –78 °C for 10 min, followed by the addition of the corresponding nucleophile (2.0 equiv) over 10 minutes, followed immediately by the addition of TMSOTf (1.2 equiv) in one portion. Subsequently, the reaction vessel was taken out of the low temperature bath and stirred at room temperature overnight. The reaction mixture was then cooled to 0 °C and quenched via the addition of methanol (0.5 mL per mmol of pyrrolidine). The resulting mixture was diluted with ether and washed with 1 M NaOH solution. A slurry was formed and filtered through a celite pad and washed with ethyl acetate. The filtrate was separated, and the aqueous layer was then extracted with ethyl acetate. The combined organic layers were washed with brine, dried over anhydrous Na_2SO_4 and concentrated. The crude mixture was purified by flash column chromatography (silica gel; dichloromethane: methanol = 20:1 ~ 10:1) to afford the target compound. For hydrochloride salts, the amine was dissolved in ethyl acetate, added 1 equiv of 4M HCl/EA solution, stirred at r.t. overnight and the product was collected by filtration.

This method was used for the synthesis of substrates **12a, 13a, 14a, 15a, 24a, 28a, 29a, 30a, 36a, 37a, 38a, 39a, 40a, 41a, 42a, 43a, 44a** and **45a**.

Synthetic Applications

Gram Scale Synthesis of Diene 45b

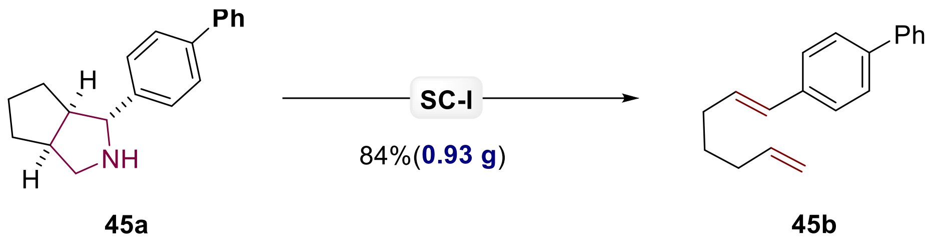

To a solution of $N_3SO_2N_3$ (44.7 mL, ~ 0.2mol/L in DCM) at 0 °C was added a solution of (±)-1-([1,1'-biphenyl]-4-yl)octahydrocyclopenta[c]pyrrole hydrochloride **45a** (1.34 g, 4.47 mmol, 1.0 equiv) and DBU (2.04 g, 13.41 mmol, 3.0 equiv) in DCM (12 mL) dropwise. After the reaction was completed based on TLC (~ 3 h), the solvent was removed under reduced pressure at room temperature and the residue was purified by quick flash column chromatography to provide the crude intermediate, which was then added to a reaction vessel filled with ^{t}BuOLi (358 mg, 4.47 mmol 1.0 equiv) and DCE (45 mL) under argon atmosphere. After being stirred at 120 °C for 3 h, the crude mixture was purified by column chromatography (petroleum ether /ethyl acetate = 20: 1) to provide (*E*)-4-(hepta-1,6-dien-1-yl)-1,1'-biphenyl **45b** (0.93 g, 84%).

Synthesis of N-deletion Product 45c by Photocyclization of Diene 45b

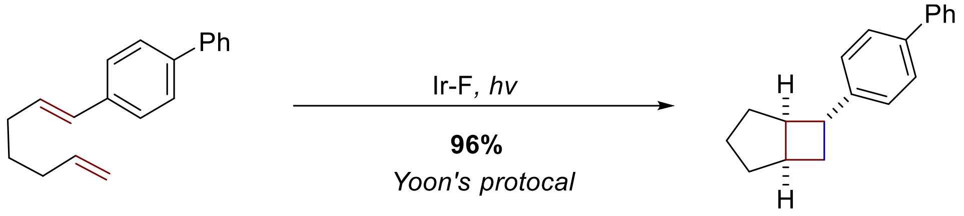

45b **45c**, *dr* = 9/1

The synthetic procedure is according to the reference.[5] A solution of Ir(dF(CF$_3$)ppy)$_2$(dtbbpy)(PF$_6$) (2.0 mg, 0.002 mmol, 1 mol%) and (*E*)-4-(hepta-1,6-dien-1-yl)-1,1'-biphenyl **45b** (49.7 mg, 0.2 mmol, 1.0 equiv) in DMSO (0.1 M) was placed in a test tube under argon atmosphere. The reaction mixture was stirred further 12 h under the irradiation of 23 W white LEDs at room temperature. Upon consumption of starting material, the reaction mixture was diluted with Et$_2$O and water. The aqueous layer was separated and extracted with ether. The combined organic layers were washed with water and brine, dried over Na$_2$SO$_4$, filtered, and concentrated in vacuo. The residue was purified by column chromatography (silica gel; hexane: ethyl acetate = 30: 1) to afford (±)-6-([1,1'-biphenyl]-4-yl)bicyclo[3.2.0]heptane **45c** (47.7 mg) in 96% yield as an oil.

Mechanistic studies

Intermolecular Radical Trapping Experiments

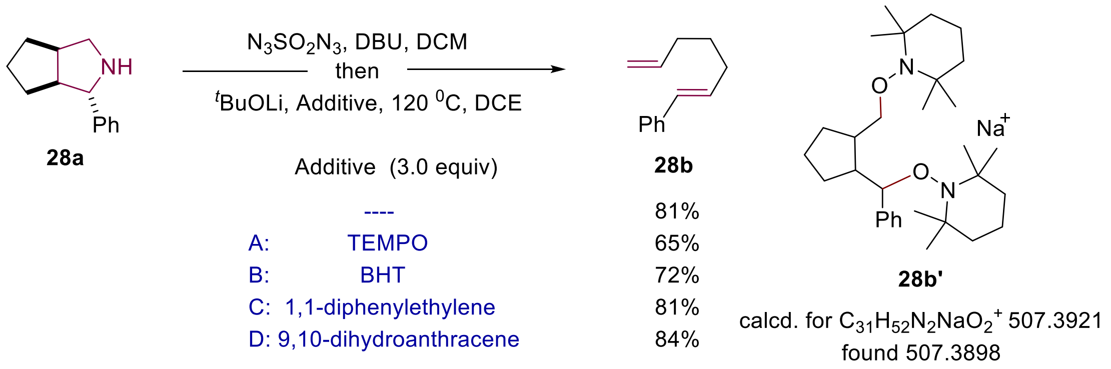

To a solution of $N_3SO_2N_3$ (15 mL, ~ 0.2mol/L in DCM) at 0 °C was added a solution of (±)-1-phenyloctahydrocyclopenta[c]pyrrole hydrochloride **28a** (224 mg, 1.0 mmol, 1.0 equiv) and DBU (456 mg, 3.0 mmol, 3.0 equiv) in DCM (12 mL) dropwise. After the reaction was completed based on TLC (~ 3 h), the solvent was removed under reduced pressure at room temperature and the residue was purified by quick flash column chromatography to provide the sulfamoyl azide intermediate.

A: The 1/5 sulfamoyl azide intermediate was then added to a reaction vessel filled with ^{t}BuOLi (16.0 mg, 0.2 mmol, 1.0 equiv), TEMPO (95.0 mg, 0.6 mmol, 3.0 equiv) and DCE (2 mL) under argon atmosphere. After being stirred at 120 °C for 3 h, the reaction mixture was filtered and concentrated in vacuo. The product (*E*)-hepta-1,6-dien-1-ylbenzene **28b** was obtained in 65% yield determined by ^{1}H-NMR spectrum with CH_2Br_2 as internal standard and captured by TEMPO product **28b'** was detected by HRMS.

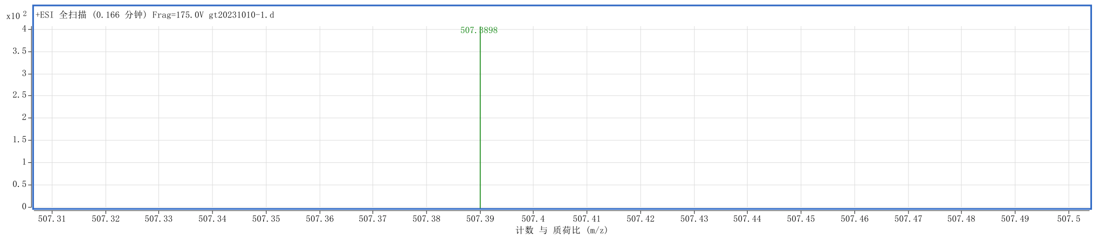

Supplementary Figure 3. The HRMS of the Product 28b'

B: The 1/5 sulfamoyl azide intermediate was then added to a reaction vessel filled with ^{t}BuOLi (16.0 mg, 0.2 mmol, 1.0 equiv), butylated hydroxytoluene (BHT, 132.2 mg, 0.6 mmol, 3.0 equiv) and DCE (2 mL) under argon atmosphere. After being stirred at 120 ^oC for 3 h, the reaction mixture was filtered and concentrated in vacuo. The product (*E*)-hepta-1,6-dien-1-ylbenzene **28b** was obtained in 72% yield determined by ^{1}H-NMR spectrum with CH_2Br_2 as internal standard.

C: The 1/5 sulfamoyl azide intermediate was then added to a reaction vessel filled with ^{t}BuOLi (16.0 mg, 0.2 mmol, 1.0 equiv), 1,1-diphenylethylene (108.2 mg, 0.6 mmol, 3.0 equiv) and DCE (2 mL) under argon atmosphere. After being stirred at 120 ^oC for 3 h, the reaction mixture was filtered and concentrated in vacuo. The product (*E*)-hepta-1,6-dien-1-ylbenzene **28b** was obtained in 81% yield determined by ^{1}H-NMR spectrum with CH_2Br_2 as internal standard.

D: The 1/5 sulfamoyl azide intermediate was then added to a reaction vessel filled with ^{t}BuOLi (16.0 mg, 0.2 mmol, 1.0 equiv), 9,10-dihydroanthracene (108.2 mg, 0.6 mmol, 3.0 equiv) and DCE (2 mL) under argon atmosphere. After being stirred at 120 ^oC for 3 h, the reaction mixture was filtered and concentrated in vacuo. The product (*E*)-hepta-1,6-dien-1-ylbenzene **28b** was obtained in 84% yield determined by ^{1}H-NMR spectrum with CH_2Br_2 as internal standard.

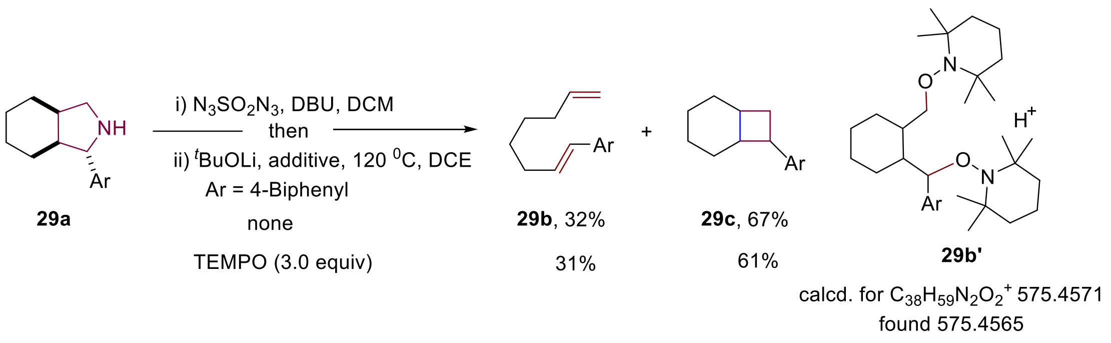

To a solution of $N_3SO_2N_3$ (15 mL, ~ 0.2mol/L in DCM) at 0 ^oC was added a solution of (±)-1-phenyloctahydrocyclopenta[c]pyrrole hydrochloride **29a** (94.2 mg, 0.3 mmol, 1.0 equiv) and DBU (137 mg, 0.9 mmol, 3.0 equiv) in DCM (3.5 mL) dropwise. After the reaction was completed based on TLC (~ 3 h), the solvent was removed under reduced pressure at room temperature and the residue was purified by

quick flash column chromatography to provide the sulfamoyl azide intermediate. The sulfamoyl azide intermediate (76.5 mg, 0.2 mmol, 1.0 equiv) was then added to a reaction vessel filled with ^{t}BuOLi (16.0 mg, 0.2 mmol, 1.0 equiv), TEMPO (95.0 mg, 0.6 mmol, 3.0 equiv) and DCE (2 mL) under argon atmosphere. After being stirred at 120 ^{o}C for 3 h, the reaction mixture was filtered and concentrated in vacuo. The product (*E*)-hepta-1,6-dien-1-ylbenzene **29b** was obtained in 31% yield as a byproduct determined by ^{1}H-NMR spectrum with CH_2Br_2 as internal standard and captured by TEMPO product **29b'** was detected by HRMS.

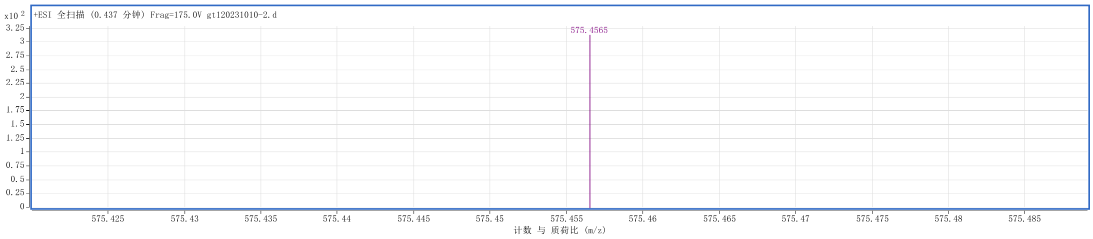

Supplementary Figure 4. The HRMS of the Product 29b'

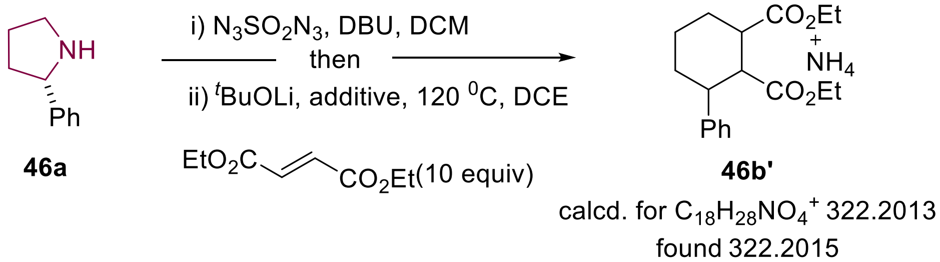

To a solution of $N_3SO_2N_3$ (15 mL, ~ 0.2mol/L in DCM) at 0 ^{o}C was added a solution of 2-phenylpyrrolidine **46a** (44.2 mg, 0.3 mmol, 1.0 equiv) and DBU (137 mg, 0.9 mmol, 3.0 equiv) in DCM (3.5 mL) dropwise. After the reaction was completed based on TLC (~ 3 h), the solvent was removed under reduced pressure at room temperature and the residue was purified by quick flash column chromatography to provide the sulfamoyl azide intermediate. The sulfamoyl azide intermediate (50.5 mg, 0.2 mmol, 1.0 equiv) was then added to a reaction vessel filled with ^{t}BuOLi (16.0 mg, 0.2 mmol, 1.0 equiv), diethyl fumarate (344.4 mg, 2.0 mmol, 3.0 equiv) and DCE (2 mL) under argon atmosphere. After being stirred at 120 ^{o}C for

3 h, the reaction **mixtu**re was filtered and concentrated in vacuo. The product **46b'** was detected by HRMS.

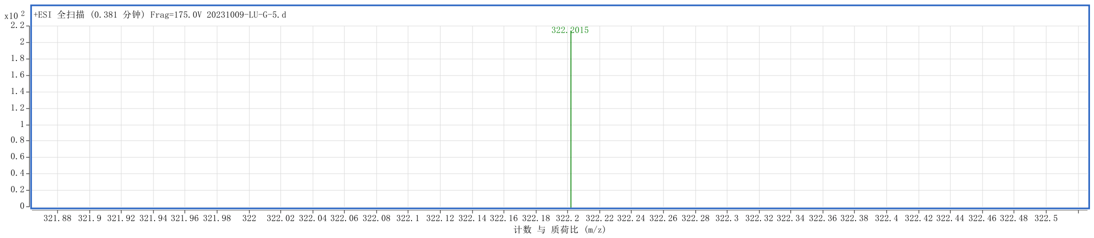

Supplementary Figure 5. The HRMS of the Product 46b'

Cyclopropane as a Radical Probe Opening Experiment

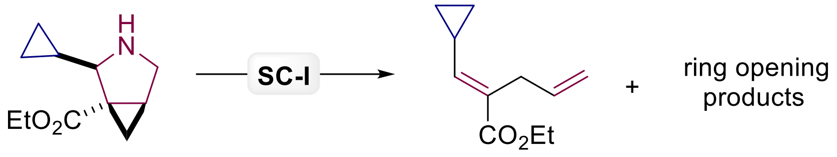

46a **46b**, 89% < 5%

To a solution of $N_3SO_2N_3$ (44.4 mL, ~ 0.2mol/L in DCM) at 0 °C was added a solution of (±)-ethyl-2-cyclopropyl-3-azabicyclo[3.1.0]hexane-1-carboxylate hydrochloride **46a** (46.3 mg, 0.2 mmol, 1.0 equiv) and DBU (91.3 mg, 0.6 mmol, 3.0 equiv) in DCM (12 mL) dropwise. After the reaction was completed based on TLC (~ 3 h), the solvent was removed under reduced pressure at room temperature and the residue was purified by quick flash column chromatography to provide the crude intermediate, which was then added to a reaction vessel filled with ^{t}BuOLi (16.0 mg, 0.2 mmol 1.0 equiv) and DCE (2 mL) under argon atmosphere. After being stirred at 120 °C for 3 h, the solvent was filtered and concentrated in vacuo to remove most solvent. The ^{1}H-NMR spectrum of crude product **46b** was achieved with $CDCl_3$ as solvent and CH_2Br_2 as internal standard (**Fig. S1**). And then the crude mixture was purified by column chromatography (petroleum ether /ethyl acetate = 20: 1) to provide ethyl (*E*)-2-(cyclopropylmethylene)pent-4-enoate **46b** (32.1 mg, 89%).

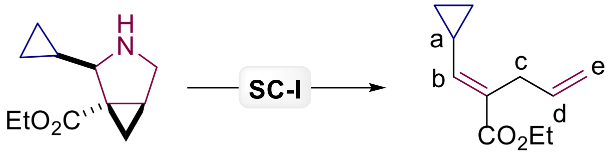

46b

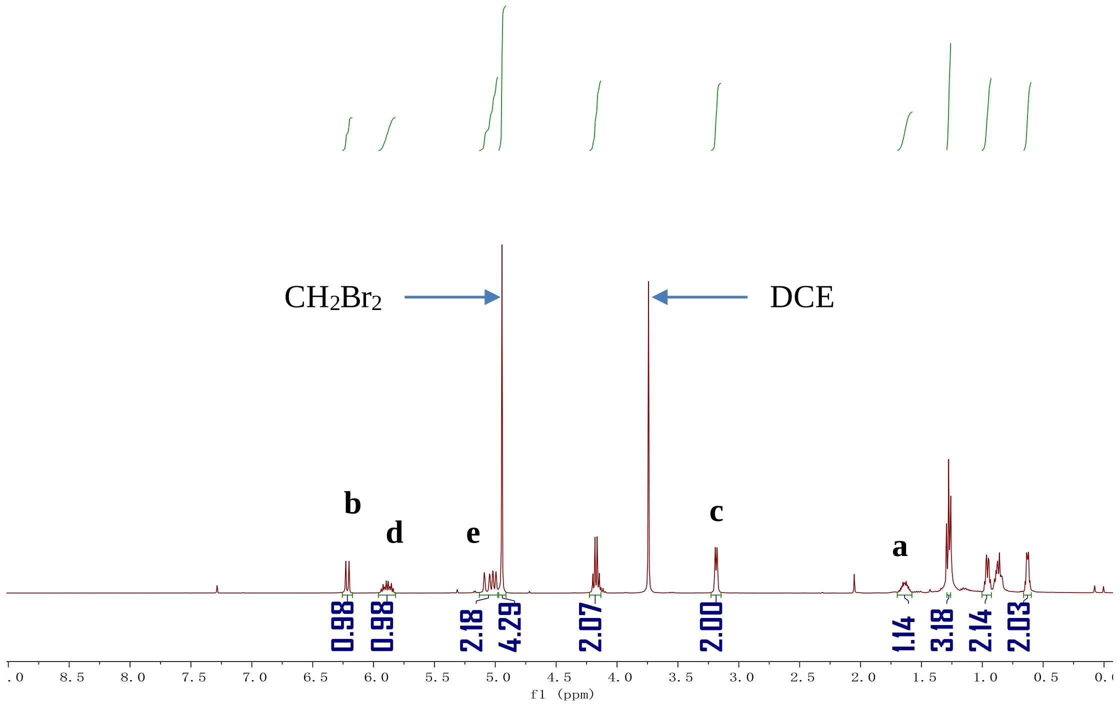

Supplementary Figure 6. The 1H NMR Spectrum of the Crude Reaction Mixture of 46a

Oxidation Reactions with *N*-Aminopyrrolidine (45d)

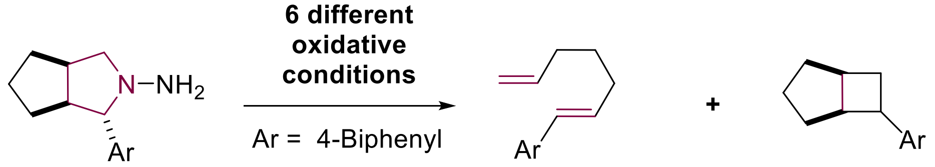

45d **45b** **45c**

Exact Mass: 248.1565 Exact Mass: 248.1565

Details:	45b	45c
**A.** HgO, 70 °C, EtOH;	No	No
**B.** HgO, rt, DCM;	No	No
**C.** $PhI(OAc)_2$, ^{t}BuOLi, DCE, 120 °C;	No	No
**D.** $PhI(OAc)_2$, 1,4-dioxane, 80 °C;	No	No
**E.** $Pb(OAc)_4$, DMSO,1,4-dioxane,100 °C ;	No	No
**F.** $Pb(OAc)_4$, PhCN, 0°C---rt---reflux.	No	No

A: The synthetic procedure is according to the reference.[6] To a solution of (±)-1-([1,1'-biphenyl]-4-yl)hexahydrocyclopenta[c]pyrrol-2(1*H*)-amine **45d** (40.5 mg, 0.2 mmol, 1.0 equiv) in EtOH (2 mL) was added HgO (86.6 mg, 0.4 mmol, 2.0 equiv). The reaction mixture was stirred at 70 °C for 7 h. The reaction mixture was filtered and concentrated to afford crude products. The N-atom removal deconstruction product (*E*)-4-(hepta-1,6-dien-1-yl)-1,1'-biphenyl **45b** and N-atom deletion product (±)-*cis*-6-([1,1'-biphenyl]-4-yl)bicyclo[3.2.0]heptane **45c** was not observed by GCMS spectrum analysis of crude products.

B: The synthetic procedure is according to the reference.[7] To a solution of (±)-1-([1,1'-biphenyl]-4-yl)hexahydrocyclopenta[c]pyrrol-2(1*H*)-amine **45d** (40.5 mg, 0.2 mmol, 1.0 equiv) in DCM (2 mL) was added HgO (86.6 mg, 0.4 mmol, 2.0 equiv). The reaction mixture was stirred at room temperature for 14 h and then at 70 °C for 14h. The reaction mixture was filtered and concentrated to afford crude products. The N-atom removal deconstruction product (*E*)-4-(hepta-1,6-dien-1-yl)-1,1'-biphenyl **45b** and N-atom deletion product (±)-*cis*-6-([1,1'-biphenyl]-4-yl)bicyclo[3.2.0]heptane **45c** was not observed by GCMS spectrum analysis of crude products.

C: To a solution of (±)-1-([1,1'-biphenyl]-4-yl)hexahydrocyclopenta[c]pyrrol-2(1*H*)-amine **45d** (40.5 mg, 0.2 mmol, 1.0 equiv) in DCE (2 mL) was added $PhI(OAc)_2$ (128.8 mg, 0.4 mmol, 2.0

equiv) and ^{t}BuOLi (16.0 mg, 0.2 mmol, 1.0 equiv). The reaction mixture was stirred at 120 °C for 3 h. The reaction mixture was filtered and concentrated to afford crude products. The N-atom removal deconstruction product (*E*)-4-(hepta-1,6-dien-1-yl)-1,1'-biphenyl **45b** and N-atom deletion product (±)-*cis*-6-([1,1'-biphenyl]-4-yl)bicyclo[3.2.0]heptane **45c** was not observed by GCMS spectrum analysis of crude products.

D: The synthetic procedure is according to the reference.[8] To a solution of (±)-1-([1,1'-biphenyl]-4-yl)hexahydrocyclopenta[c]pyrrol-2(1*H*)-amine **45d** (40.5 mg, 0.2 mmol, 1.0 equiv) in 1,4-dioxane (2 mL) was added $PhI(OAc)_2$ (128.8 mg, 0.4 mmol, 2.0 equiv). The reaction mixture was stirred at 80 °C for 24 h. The reaction mixture was filtered and concentrated to afford crude products. The N-atom removal deconstruction product (*E*)-4-(hepta-1,6-dien-1-yl)-1,1'-biphenyl **45b** and N-atom deletion product (±)-*cis*-6-([1,1'-biphenyl]-4-yl)bicyclo[3.2.0]heptane **45c** was not observed by GCMS spectrum analysis of crude products.

E: The synthetic procedure is according to the reference.[9] To a solution of (±)-1-([1,1'-biphenyl]-4-yl)hexahydrocyclopenta[c]pyrrol-2(1*H*)-amine **45d** (40.5 mg, 0.2 mmol, 1.0 equiv) in 1,4-dioxane (2 mL) was added $Pb(OAc)_4$ (355.6 mg, 0.4 mmol, 2.0 equiv) and DMSO (18.8 mg, 0.24 mmol, 1.2 equiv). The reaction mixture was stirred at 70 °C for 14 h. The reaction mixture was filtered and concentrated to afford crude products. The N-atom removal deconstruction product (*E*)-4-(hepta-1,6-dien-1-yl)-1,1'-biphenyl **45b** and N-atom deletion product (±)-*cis*-6-([1,1'-biphenyl]-4-yl)bicyclo[3.2.0]heptane **45c** was not observed by GCMS spectrum analysis of crude products.

F: The synthetic procedure is according to the reference.[10] To a solution of (±)-1-([1,1'-biphenyl]-4-yl)hexahydrocyclopenta[c]pyrrol-2(1*H*)-amine **45d** (40.5 mg, 0.2 mmol, 1.0 equiv) in PhCN (2 mL) was added $Pb(OAc)_4$ (355.6 mg, 0.4 mmol, 2.0 equiv). The reaction mixture was stirred at room temperature for 14 h and at 70 °C for 14 h. The reaction mixture was filtered and concentrated to afford crude products. The N-atom removal deconstruction product (*E*)-4-(hepta-1,6-dien-1-yl)-1,1'-biphenyl **45b**

and N-atom deletion product (±)-*cis*-6-([1,1'-biphenyl]-4-yl)bicyclo[3.2.0]heptane **45c** was not observed by GCMS spectrum analysis of crude products.

The Observation of N-atom Deletion Side Products

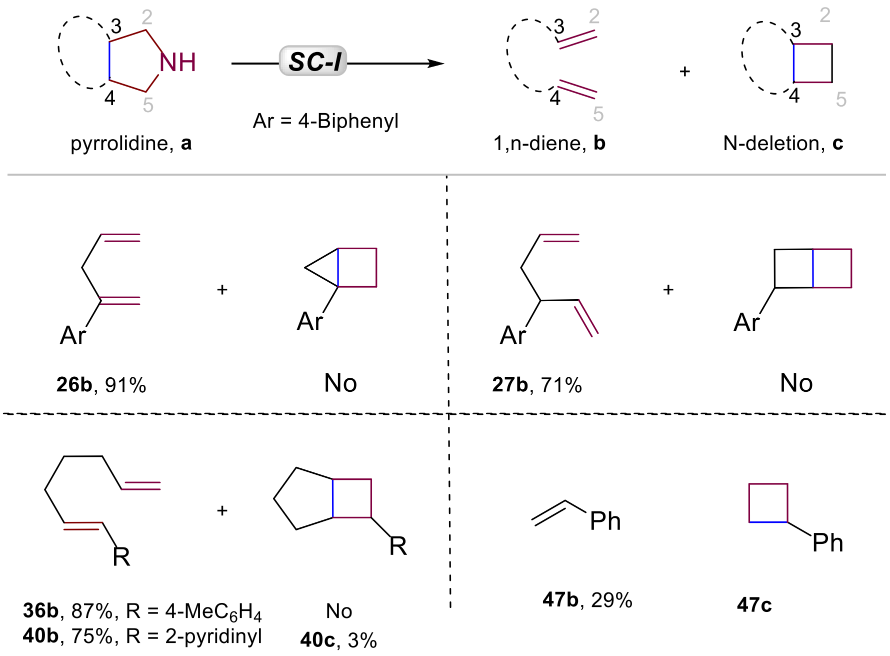

Supplementary Figure 7. The Observation of N-atom Deletion Side Products

To a solution of $N_3SO_2N_3$ (3 mL, ~ 0.2mol/L in DCM) at 0 °C was added a solution of pyrrolidine **a** (0.2 mmol, 1.0 equiv) and DBU (0.4 mmol, 2.0 equiv, 0.6 mmol, 3.0 equiv if using amine hydrochloride a•HCl as the substrate) in DCM (0.5 mL) dropwise. After the reaction was completed based on TLC (~ 3 h), the solvent was removed under reduced pressure at room temperature and the residue was purified by quick flash column chromatography to provide the crude intermediate, which was then added to a reaction vessel filled with ^{t}BuOLi (1.0 equiv, relative to sulfamoyl azide) and DCE (2 mL) under argon atmosphere. After being stirred at 120 °C for 3 h, the reaction mixture was filtered and concentrated in vacuo. The ^{1}H-NMR spectrum of crude products was achieved with $CDCl_3$ as solvent and CH_2Br_2 as an internal standard.

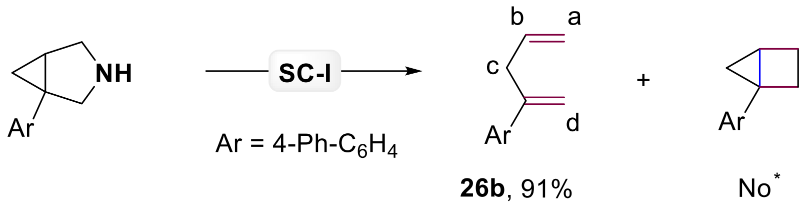

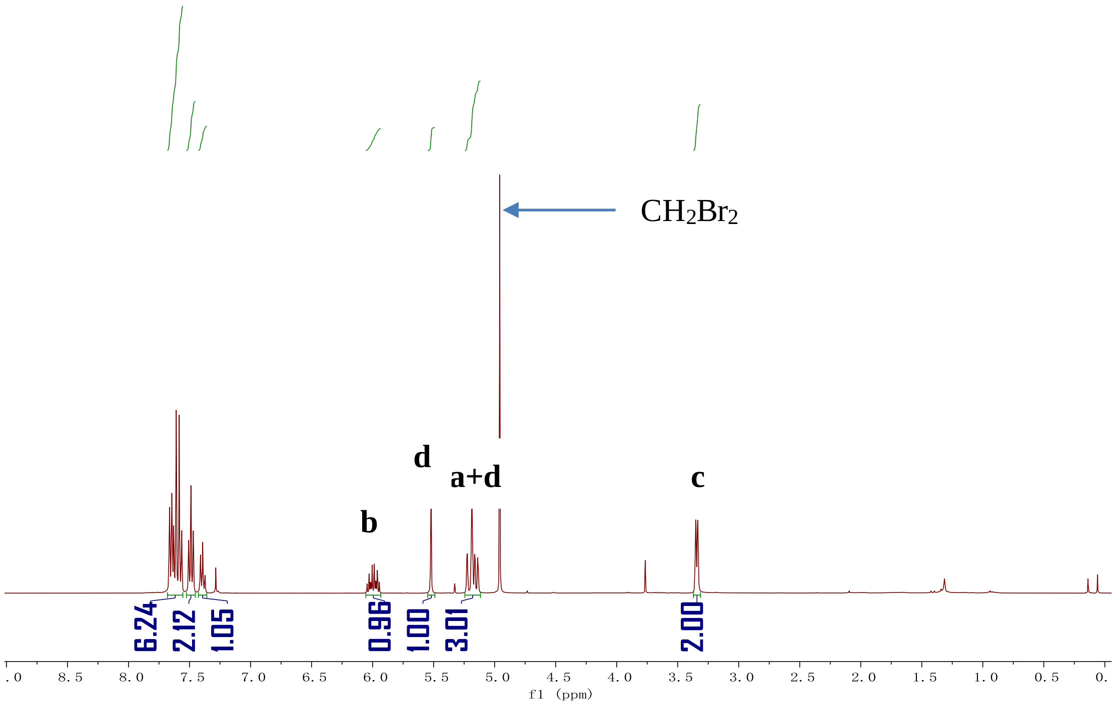

Supplementary Figure 8. The 1H NMR Spectrum of the Crude Reaction Mixture of 26a

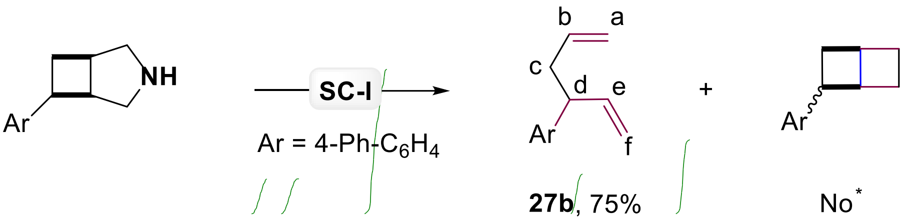

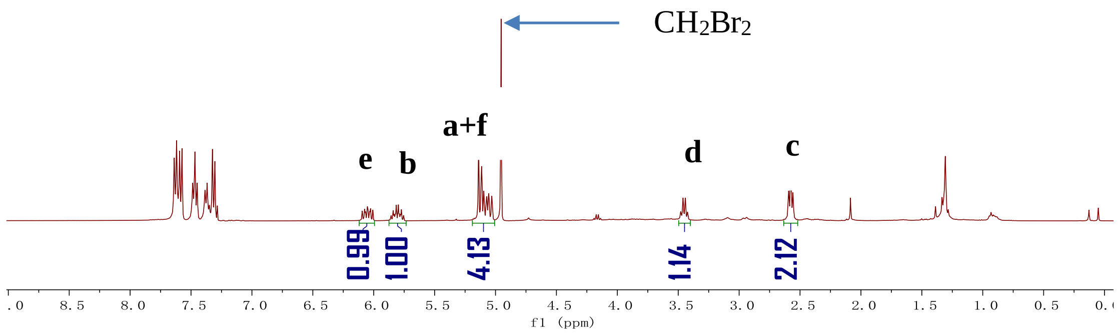

Supplementary Figure 9. The 1H NMR Spectrum of the Crude Reaction Mixture of 27a

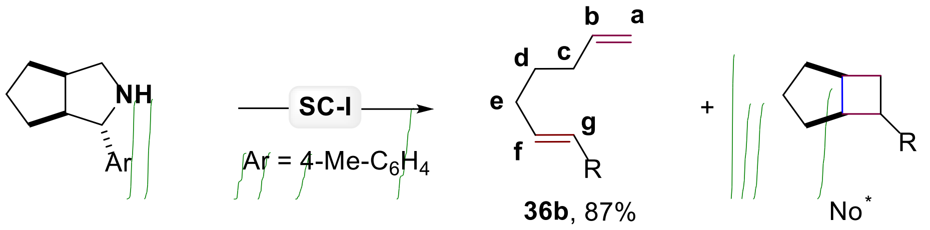

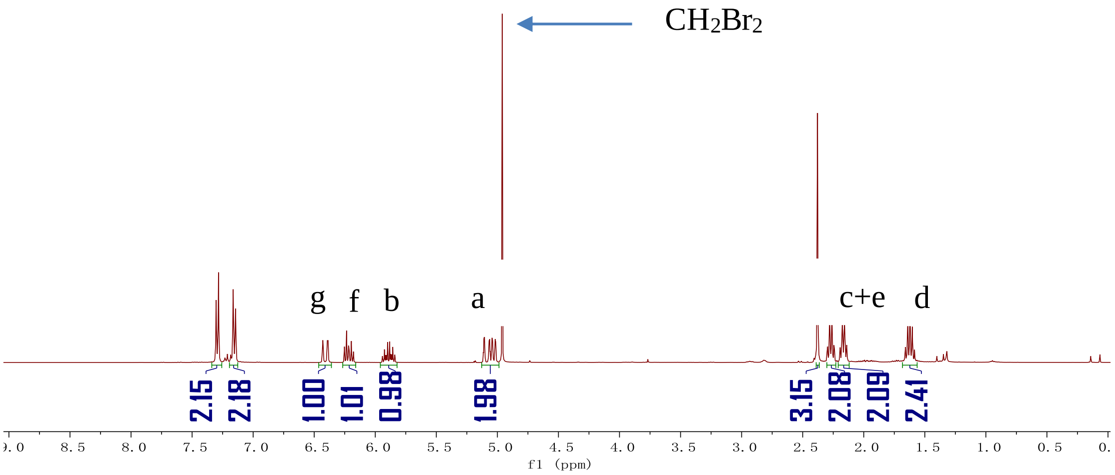

Supplementary Figure 10. The 1H NMR Spectrum of the Crude Reaction Mixture of 36a

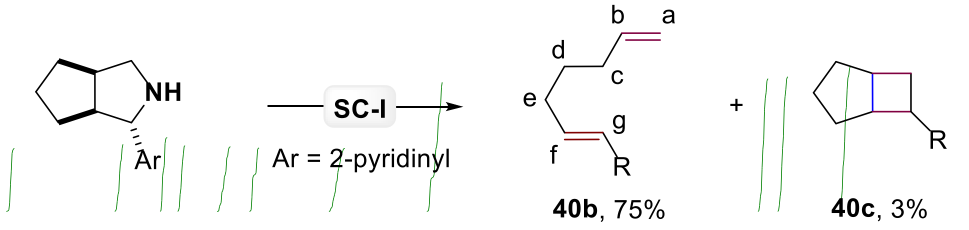

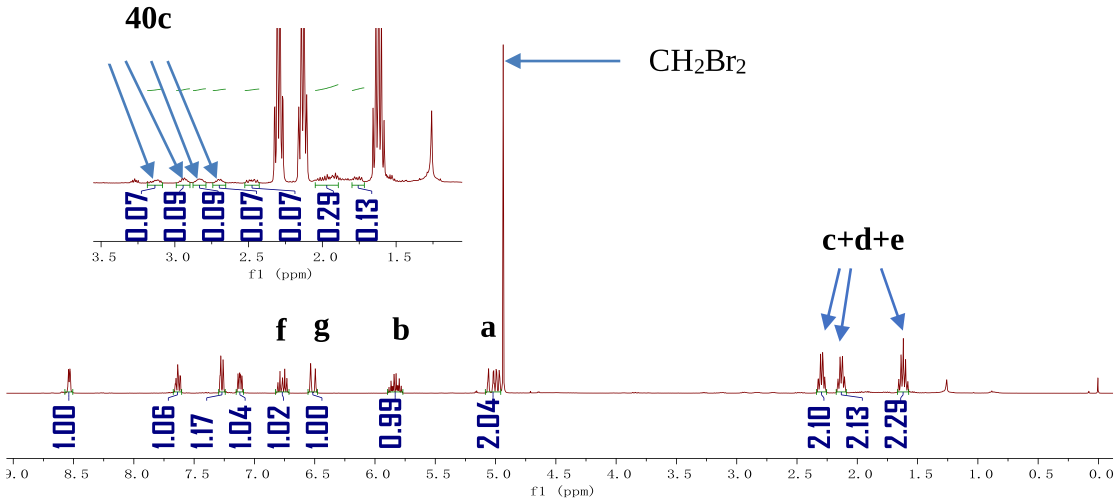

Supplementary Figure 11. The 1H NMR Spectrum of the Crude Reaction Mixture of 40a

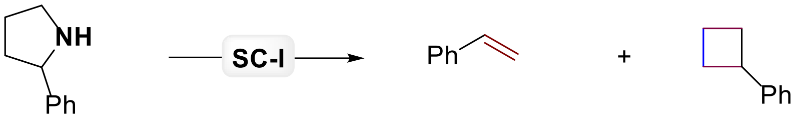

47b, 29%　　**47c**

峰表 TIC

峰号	保留时间	I.Time	F.Time	峰面积	峰面积%	峰高	峰高%	积/高度	标记	名称
1	3.714	3.680	3.765	483641	5.43	297138	3.93	1.63	MI	Styrene
2	5.349	5.320	5.415	227034	2.55	106368	1.41	2.13	MI	
3	5.793	5.765	5.835	8195581	92.02	7151792	94.66	1.15	MI	Dodecane
				8906256	100.00	7555298	100.00			

色谱图　D:\DATA\data\lu group\lk\qht-3he1.qgd

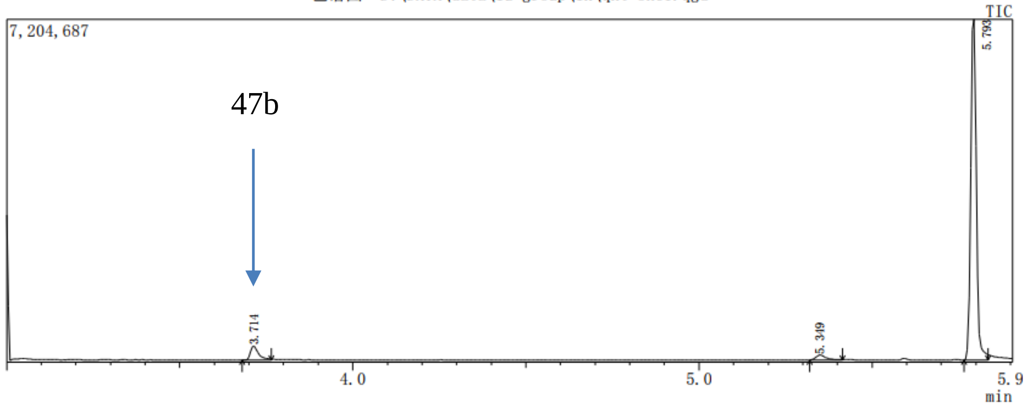

质谱

流路号:1　保留时间:3.710(扫描数:143)
质量峰:781
原始模式:单个 3.710(143)　基峰:104(98662)
背景模式:无　组 1 - 事件 1　Scan

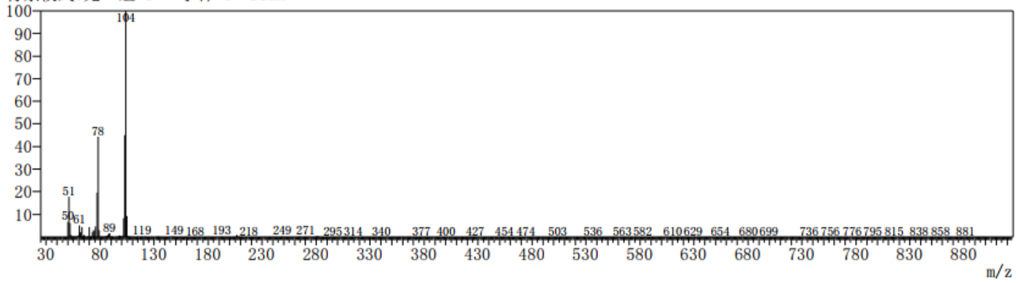

Supplementary Figure 12. The GCMS Spectrum of the Crude Reaction Mixture of 47a

Characterization Data

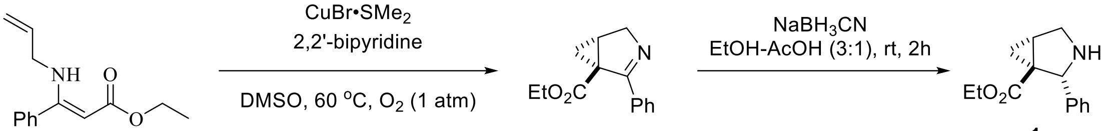

1a

1a is a known compound which was prepared from ethyl (*Z*)-3-(allylamino)-3-phenylacrylate according to the reference.[1] To a solution of ethyl 3-(allylamino)-3-phenylacrylate (1.66 g, 7.21 mmol, 1.0 equiv) in DMSO (30mL) was added CuBr•SMe$_2$ (1.63 g, 7.93 mmol, 1.1 equiv) and 2,2'-bipyridine (1.24 g, 7.93 mmol, 1.1 equiv). The reaction mixture was then stirred for 3.5 h at 60 °C under an O_2 atmosphere. The resulting mixture was quenched with water and extracted thrice with EtOAc. The combined organic extracts were washed with water, pH 9 buffer and brine, dried over $MgSO_4$ and concentrated. The crude product was purified by flash column chromatography (silica gel; hexane: ethyl acetate = 10: 1) to afford ethyl 2-phenyl-3-azabicyclo[3.1.0]hex-2-ene-1-carboxylate (1.39 g, 6.06 mmol) in 84% yield as a white solid. Then to a solution of ethyl 2-phenyl-3-azabicyclo[3.1.0]hex-2-ene-1-carboxylate (1.39 g, 6.06 mmol, 1.0 equiv) in EtOH (15 mL) and AcOH (1.5 mL) was added $NaBH_3CN$ (1.16 g, 18.18 mmol, 3.0 equiv). The reaction mixture was then stirred for 2 h at room temperature under an N_2 atmosphere. The resulting mixture was quenched with aqueous NH_4OH and the organic materials were extracted thrice with dichloromethane. The combined organic extracts were washed with brine, dried over Na_2SO_4 and concentrated. The crude mixture was purified by short flash column chromatography (silica gel; hexane: ethyl acetate = 1:1 to dichloromethane: methanol = 20:1) to afford **1a** (1.21g, 5.23 mmol) in 86% yield as a white solid.

(±)-Ethyl-2-phenyl-3-azabicyclo[3.1.0]hexane-1-carboxylate (**1a**). **^{1}H NMR** (400 MHz, Chloroform-*d*) δ 7.53 – 7.47 (m, 2H), 7.34 (dd, *J* = 8.2, 6.3 Hz, 2H), 7.31 – 7.25 (m, 1H), 4.82 (s, 1H), 4.20 – 4.08 (m, 2H), 3.23 (dd, *J* = 10.9, 3.5 Hz, 1H), 3.11

(d, J = 10.9 Hz, 1H), 2.08 – 2.04 (m, 1H), 1.74 (s, 1H), 1.58 (dd, J = 8.3, 4.9 Hz, 1H), 1.27 (t, J = 5.0 Hz, 1H), 1.23 (t, J = 7.1 Hz, 3H). **^{13}C NMR** (101 MHz, Chloroform-*d*) δ 172.9, 140.4, 128.2, 127.6, 127.3, 61.8, 60.5, 47.7, 34.8, 30.3, 14.2, 12.6. **HRMS** (ESI) m/z: $(M + Na)^+$ calcd. for $C_{14}H_{17}NO_2\,Na^+$ 254.1151, found 254.1148.

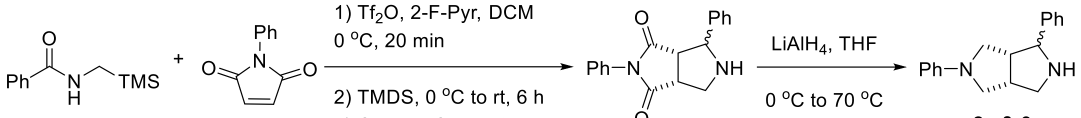

2a & 3a

2a and **3a** were prepared from *N*-((trimethylsilyl)methyl)benzamide and 1-phenyl-1H-pyrrole-2,5-dione according to the reference.[11] Into a dry 5 mL round-bottom flask equipped with a stirring bar were successively added *N*-((trimethylsilyl)methyl)benzamide (cas: 71572-22-0, 405 mg, 1.95 mmol, 1.0 equiv), 8 mL of anhydrous dichloromethane, and 2-fluoropyridine (227 mg, 2.34 mmol, 1.2 equiv). After being cooled to −78 °C for 30 min, trifluoromethanesulfonic anhydride (Tf_2O) (605 mg, 2.15 mmol, 1.1 equiv) was added dropwise via a syringe at 0 °C, and the reaction mixture was stirred for 20 min. To the resulting mixture was added 1,1,3,3-tetramethyldisiloxane (TMDS) (182 mg, 1.37 mmol, 0.7 equiv) dropwise at 0 °C, and the reaction was stirred for 10 min. The mixture was allowed to warm to rt and stirred for 6 h. The solvent was removed through a drying tube charged with anhydrous $CaCl_2$ under reduced pressure. Acetonitrile (5 mL) was added to dissolve the residue, and then a solution of 1-phenyl-1H-pyrrole-2,5-dione (676 mg, 3.90 mmol, 2.0 equiv) in acetonitrile (3 mL) was added. The resulting mixture was added dropwise to the suspension of cesium fluoride (889 mg, 5.85 mmol, 3.0 equiv) in acetonitrile (5 mL) with vigorous stirring. The reaction mixture was stirred for another 14 h at rt. The solvents were removed under reduced pressure, and the crude mixture was purified by short flash column chromatography to give corresponding cycloadduct (±)-*cis*-2,4-diphenyltetrahydropyrrolo[3,4-c]pyrrole-1,3(2H,3aH)-dione (200 mg, 0.68 mmol, 35%) and (±)-*trans*-2,4-diphenyltetrahydropyrrolo[3,4-c]pyrrole-1,3(2H,3aH)-dione (178 mg, 0.61 mmol, 31%).

To the $LiAlH_4$ (78 mg, 2.05 mmol, 3.0 equiv) in THF was added (±) -*cis*-2,4-diphenyltetrahydropyrrolo[3,4-c]pyrrole-1,3(2H,3aH)-dione (200 mg, 0.684 mmol, 1.0 equiv) at 0 ^{o}C, then the reaction mixture was warmed to 70 °C and stirred for additional 25 h. The resulting mixture was quenched with H_2O, extracted with ethyl acetate, washed with brine, dried over Na_2SO_4 and concentrated. Purification of the crude mixture by short flash column chromatography (silica gel; hexane: ethyl acetate = 1:1 to dichloromethane: methanol = 10:1) to afford **2a** (72 mg, 0.27 mmol) in 40% yield as brown oil.

The compound **3a** (65 mg, 0.25 mmol) was achieved in 36% yield as brown oil by the same reduction process from (±) -*trans*-2,4-diphenyltetrahydropyrrolo[3,4-c]-pyrrole-1,3(2H,3aH)-dione.

(±)-*cis*-1,5-Diphenyloctahydropyrrolo[3,4-c]pyrrole (2a). **^{1}H NMR** (400 MHz, Chloroform-*d*) δ 7.43 – 7.37 (m, 2H), 7.34 – 7.27 (m, 3H), 7.19 – 7.11 (m, 2H), 6.70 (tt, *J* = 7.3, 1.1 Hz, 1H), 6.53 – 6.47 (m, 2H), 6.07 (s, 1H), 4.64 (d, *J* = 6.7 Hz, 1H), 3.45 – 3.29 (m, 3H), 3.28 – 3.19 (m, 2H), 3.11 (tq, *J* = 7.9, 4.4, 3.8 Hz, 1H), 2.98 (d, *J* = 7.0 Hz, 2H). **^{13}C NMR** (101 MHz, Chloroform-*d*) δ 148.0, 135.7, 129.0, 128.8, 128.0, 127.0, 117.5, 113.7, 65.7, 54.5, 51.5, 49.6, 46.5, 41.6. **HRMS** (ESI) m/z: (M + Na)$^+$ calcd. for $C_{18}H_{21}N_2Na^+$ 287.1519, found 287.1516.

(±)-*trans*-1,5-Diphenyloctahydropyrrolo[3,4-c]pyrrole (3a). **^{1}H NMR** (400 MHz, Chloroform-*d*) δ 7.59 – 7.53 (m, 2H), 7.40 – 7.30 (m, 3H), 7.29 – 7.22 (m, 2H), 6.79 (tt, *J* = 7.4, 1.1 Hz, 1H), 6.70 – 6.64 (m, 2H), 4.92 (s, 1H), 4.07 (d, *J* = 8.6 Hz, 1H), 3.67 – 3.56 (m, 1H), 3.47 – 3.34 (m, 2H), 3.24 – 3.16 (m, 2H), 3.15 – 3.04 (m, 2H), 2.95 – 2.85 (m, 1H). **^{13}C NMR** (101 MHz, Chloroform-*d*) δ 148.4, 136.9, 129.2, 129.1, 128.8, 128.1, 118.0, 114.0, 68.7, 53.6, 52.0, 51.4, 50.0, 41.5. **HRMS** (ESI) m/z: (M + Na)$^+$ calcd. for $C_{18}H_{21}N_2Na^+$ 287.1519, found 287.1521.

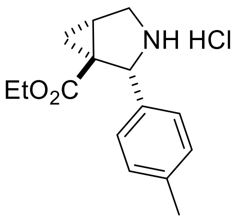

(±)-Ethyl-2-(*p*-tolyl)-3-azabicyclo[3.1.0]hexane-1-carboxylate hydrochloride (**4a**). Prepared according to the synthetic procedure for the synthesis of **1a**.[1] Purification of the crude mixture by short flash column chromatography (silica gel; hexane: ethyl acetate = 1:1 to dichloromethane: methanol = 20:1) and formation of amine hydrochloride by adding HCl (4.0 M in EA) to afford **4a** (300 mg, 1.06 mmol) in 75% yield as a white solid (last step). **^{1}H NMR** (400 MHz, Chloroform-*d*) δ 10.73 (s, 1H), 9.31 (s, 1H), 7.48 (d, J = 7.9 Hz, 2H), 7.15 (d, J = 7.9 Hz, 2H), 5.20 (dd, J = 9.9, 5.7 Hz, 1H), 4.11 (q, J = 7.1 Hz, 2H), 3.34 – 3.24 (m, 2H), 2.33 (s, 3H), 2.12 – 2.05 (m, 1H), 1.94 (q, J = 7.3, 6.7 Hz, 1H), 1.85 – 1.77 (m, 1H), 1.18 (t, J = 7.1 Hz, 3H). **^{13}C NMR** (101 MHz, Chloroform-*d*) δ 169.7, 139.2, 129.6, 129.0, 128.4, 62.2, 61.6, 45.6, 32.5, 25.4, 21.2, 14.0, 13.8. **HRMS** (ESI) m/z: $(M + Na)^+$ calcd. for $C_{15}H_{19}NO_2\ Na^+$ 268.1308, found 268.1305.

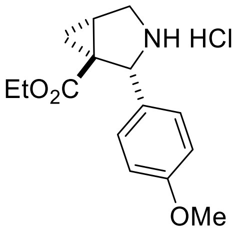

(±)-Ethyl-2-(4-methoxyphenyl)-3-azabicyclo[3.1.0]hexane-1-carboxylate hydrochloride (**5a**). Prepared according to the synthetic procedure for the synthesis of **1a**.[1] Purification of the crude mixture by short flash column chromatography (silica gel; hexane: ethyl acetate = 1:1 to dichloromethane: methanol = 20:1) and formation of amine hydrochloride by adding HCl solution (4.0 M in EA) to afford **5a** (350 mg, 1.18 mmol) in 78% yield as a white solid (last step). **^{1}H NMR** (400 MHz, Chloroform-*d*) δ 10.69 (s, 1H), 9.28 (s, 1H), 7.53 (d, J = 8.7 Hz, 2H), 6.87 (d, J = 8.7 Hz, 2H), 5.19 (dd, J = 9.9, 5.6 Hz, 1H), 4.12 (q, J = 7.1 Hz, 2H), 3.80 (s, 3H), 3.34 –

3.26 (m, 2H), 2.12 – 2.08 (m, 1H), 1.97 (t, *J* = 5.9 Hz, 1H), 1.83 (t, *J* = 7.6 Hz, 1H), 1.18 (t, *J* = 7.1 Hz, 3H). **^{13}C NMR** (101 MHz, Chloroform-*d*) δ 169.7, 160.3, 129.9, 123.8, 114.3, 62.1, 61.6, 55.2, 45.6, 32.5, 25.2, 14.0, 13.7. **HRMS** (ESI) m/z: (M + Na)$^+$ calcd. for $C_{15}H_{19}NO_3Na^+$ 284.1257, found 284.1254.

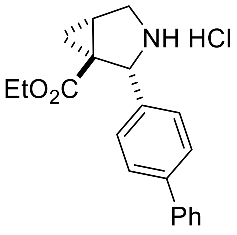

(±)-Ethyl-2-([1,1'-biphenyl]-4-yl)-3-azabicyclo[3.1.0]hexane-1-carboxylate hydrochloride (6a). Prepared according to the synthetic procedure for the synthesis of **1a**.[1] Purification of the crude mixture by short flash column chromatography (silica gel; hexane: ethyl acetate = 1:1 to dichloromethane: methanol = 20:1) and formation of amine hydrochloride by adding HCl (4.0 M in EA) to afford **6a** (312 mg, 0.91 mmol) in 90% yield as a white solid (last step). **^{1}H NMR** (400 MHz, Chloroform-d) δ 10.88 (s, 1H), 9.47 (s, 1H), 7.69 (d, *J* = 8.2 Hz, 2H), 7.62 – 7.49 (m, 4H), 7.46 – 7.32 (m, 3H), 5.32 – 5.25 (m, 1H), 4.12 (q, *J* = 7.1 Hz, 2H), 3.35 (dd, *J* = 11.9, 5.7 Hz, 2H), 2.08 (dd, *J* = 8.6, 4.3 Hz, 1H), 2.00 (t, *J* = 6.0 Hz, 1H), 1.85 (t, *J* = 7.5 Hz, 1H), 1.18 (t, *J* = 7.1 Hz, 3H). **^{13}C NMR** (101 MHz, Chloroform-d) δ 169.6, 142.2, 140.0, 130.9, 129.0, 128.8, 127.7, 127.5, 127.0, 62.2, 61.6, 45.7, 32.6, 25.3, 14.0, 13.8. **HRMS** (ESI) m/z: (M + H)$^+$ calcd. for $C_{20}H_{22}NO_2^+$ 308.1645, found 308.1637.

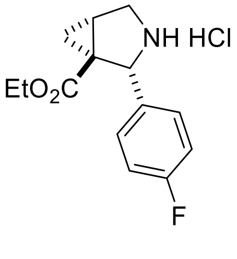

(±)-Ethyl-2-(4-fluorophenyl)-3-azabicyclo[3.1.0]hexane-1-carboxylate

hydrochloride (**7a**). Prepared according to the synthetic procedure for the synthesis of **1a**.[1] Purification of the crude mixture by short flash column chromatography (silica gel; hexane: ethyl acetate = 1:1 to dichloromethane: methanol = 20:1) and formation of amine hydrochloride by adding HCl (4.0 M in EA) to afford **7a** (280 mg, 0.98 mmol) in 72% yield as a white solid (last step). **^{1}H NMR** (400 MHz, Chloroform-*d*) δ 10.78 (s, 1H), 9.47 (s, 1H), 7.59 (dd, *J* = 8.0, 5.8 Hz, 2H), 7.04 (t, *J* = 8.6 Hz, 2H), 5.21 (dd, *J* = 9.9, 5.8 Hz, 1H), 4.17 – 4.09 (m, 2H), 3.38 – 3.31 (m, 2H), 2.17 (dt, *J* = 8.7, 4.6 Hz, 1H), 1.95 (t, *J* = 6.0 Hz, 1H), 1.86 (t, *J* = 7.7 Hz, 1H), 1.18 (t, *J* = 7.1 Hz, 3H). **^{13}C NMR** (101 MHz, Chloroform-*d*) δ 169.4, 163.2 (d, *J* = 249.4 Hz), 130.5 (d, *J* = 8.4 Hz), 127.8(d, *J* = 3.3 Hz), 115.9 (d, *J* = 21.7 Hz), 61.7 (d, *J* = 7.2 Hz), 45.7, 32.4 (d, *J* = 2.4 Hz), 25.2 (d, *J* = 2.6 Hz), 14.0, 13.8 (d, *J* = 4.0 Hz). **HRMS** m/z (ESI) calcd. for $C_{14}H_{16}NO_2Na^+$ $(M + Na)^+$ 272.1057, found 272.1055.

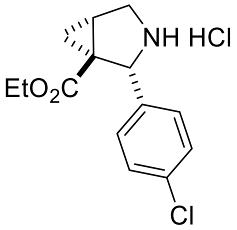

(±)-Ethyl-2-(4-chlorophenyl)-3-azabicyclo[3.1.0]hexane-1-carboxylate hydrochloride (**8a**). Prepared according to the synthetic procedure for the synthesis of **1a**.[1] Purification of the crude mixture by short flash column chromatography (silica gel; hexane: ethyl acetate = 1:1 to dichloromethane: methanol = 20:1) and formation of amine hydrochloride by adding HCl (4.0 M in EA) to afford **8a** (320 mg, 1.06 mmol) in 77% yield as a white solid (last step). **^{1}H NMR** (400 MHz, Chloroform-*d*) δ 10.78 (s, 1H), 9.51 (s, 1H), 7.60 – 7.46 (m, 2H), 7.39 – 7.31 (m, 2H), 5.21 (dd, *J* = 9.8, 5.7 Hz, 1H), 4.13 (q, *J* = 7.1 Hz, 2H), 3.31 (d, *J* = 6.2 Hz, 2H), 2.20 – 2.16 (m, 1H), 1.94 (t, *J* = 5.9 Hz, 1H), 1.90 – 1.82 (m, 1H), 1.19 (t, *J* = 7.1 Hz, 3H). **^{13}C NMR** (101 MHz, Chloroform-*d*) δ 169.4, 135.6, 130.4, 130.0, 129.2, 61.8, 45.8, 32.3, 25.3, 14.0, 13.9. **HRMS** (ESI) m/z: $(M + Na)^+$ calcd. for $C_{14}H_{16}NO_2Na^+$ 288.0762, found 288.0758.

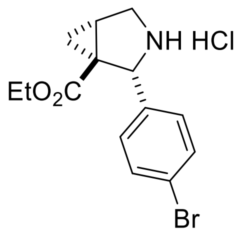

(±)-Ethyl-2-(4-bromophenyl)-3-azabicyclo[3.1.0]hexane-1-carboxylate hydrochloride (**9a**). Prepared according to the synthetic procedure for the synthesis of **1a**.[1] Purification of the crude mixture by short flash column chromatography (silica gel; hexane: ethyl acetate = 1:1 to dichloromethane: methanol = 20:1) and formation of amine hydrochloride by adding HCl (4.0 M in EA) to afford **9a** (620 mg, 1.79 mmol) in 75% yield as a white solid (last step). ^{1}**H NMR** (400 MHz, Chloroform-*d*) δ 10.82 (s, 1H), 9.52 (s, 1H), 7.50 (d, *J* = 1.6 Hz, 4H), 5.19 (dd, *J* = 10.0, 5.7 Hz, 1H), 4.14 (q, *J* = 7.1 Hz, 2H), 3.32 (d, *J* = 6.0 Hz, 2H), 2.20 (d, *J* = 6.3 Hz, 1H), 1.93 (t, *J* = 6.0 Hz, 1H), 1.89 – 1.81 (m, 1H), 1.19 (t, *J* = 7.1 Hz, 3H). ^{13}**C NMR** (101 MHz, Chloroform-*d*) δ 169.4, 132.1, 130.9, 130.2, 123.9, 61.9, 61.8, 45.8, 32.3, 25.3, 14.0, 13.8. **HRMS** (ESI) m/z: $(M + Na)^+$ calcd. for $C_{14}H_{16}BrNO_2\ Na^+$ 332.0257, found 332.0252.

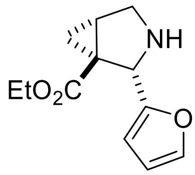

(±)-Ethyl-2-(furan-2-yl)-3-azabicyclo[3.1.0]hexane-1-carboxylate (**10a**). Prepared according to the synthetic procedure for the synthesis of **1a**.[1] Purification of the crude mixture by short flash column chromatography (silica gel; hexane: ethyl acetate = 1:1 to dichloromethane: methanol = 20:1) to afford **10a** (288 mg, 1.30 mmol) in 84% yield as a a brown oil (last step). ^{1}**H NMR** (400 MHz, Chloroform-*d*) δ 7.36 (m, 1H), 6.33 (dd, *J* = 3.3, 1.9 Hz, 1H), 6.29 (d, *J* = 3.2 Hz, 1H), 4.80 (s, 1H), 4.19 – 4.10 (m, 2H), 3.18 (dd, *J* = 11.5, 3.4 Hz, 1H), 3.09 (d, *J* = 11.4 Hz, 1H), 2.17 (s, 1H), 2.06 (m, 1H), 1.55 (d, *J* = 5.2 Hz, 1H), 1.34 (t, *J* = 5.2 Hz, 1H), 1.24 (t, *J* = 7.1 Hz, 4H). ^{13}**C NMR** (101 MHz, Chloroform-*d*) δ 172.4, 153.4, 141.8, 110.1, 106.7, 60.6, 56.6,

47.8, 34.6, 30.3, 14.2, 12.8. **HRMS** (ESI) m/z: $(M + Na)^+$ calcd. for $C_{12}H_{15}NO_3Na^+$ 244.0944, found 244.0940.

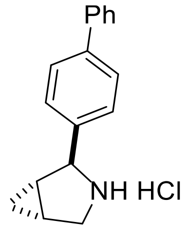

(±)-2-([1,1'-Biphenyl]-4-yl)-3-azabicyclo[3.1.0]hexane hydrochloride (**12a**). Prepared according to the General C−H Functionalization Procedure with the corresponding Grignard's reagent as the nucleophile. Purification of the crude mixture by short flash column chromatography (silica gel; dichloromethane: methanol = 20:1 ~ 10:1) and formation of amine hydrochloride by adding HCl (4.0 M in EA) to afford **12a** (350 mg, 1.29 mmol) in 50% yield as a white solid. **^{1}H NMR** (400 MHz, Chloroform-*d*) δ 10.24 (s, 1H), 9.72 (s, 1H), 7.65 (d, *J* = 7.5 Hz, 2H), 7.59 (d, *J* = 7.6 Hz, 2H), 7.53 – 7.42 (m, 4H), 7.38 (t, *J* = 7.3 Hz, 1H), 4.87 (s, 1H), 3.56 (s, 2H), 1.99 (s, 1H), 1.87 (q, *J* = 7.9, 5.8 Hz, 1H), 1.33 – 1.25 (m, 1H), 0.95 (q, *J* = 7.6 Hz, 1H). **^{13}C NMR** (101 MHz, Chloroform-*d*) δ 142.4, 140.1, 134.4, 128.8, 128.2, 127.9, 127.7, 127.2, 62.8, 46.1, 21.7, 16.2, 7.8. **HRMS** (ESI) m/z: $(M + H)^+$ calcd. for $C_{17}H_{18}N^+$ 236.1434, found 236.1425.

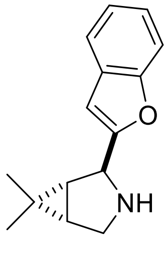

(±)-2-(Benzofuran-2-yl)-6,6-dimethyl-3-azabicyclo[3.1.0]hexane (13a). Prepared according to the General C−H Functionalization Procedure with the corresponding lithium reagent as the nucleophile. Purification of the crude mixture by short flash column chromatography (silica gel; dichloromethane: methanol = 20:1 ~ 10:1) to afford **13a** (345 mg, 1.52 mmol) in 48% yield as a brown solid. **^{1}H NMR** (400 MHz,

Chloroform-*d*) δ 7.50 (d, *J* = 7.4, 1H), 7.47 – 7.39 (m, 1H), 7.26 – 7.16 (m, 2H), 6.63 (s, 1H), 4.57(s, 1H), 4.42 (s, 1H), 3.48 (dd, *J* = 11.2, 5.2 Hz, 1H), 3.06 (d, *J* = 11.1 Hz, 1H), 1.63 (d, *J* = 7.4 Hz, 1H), 1.58 – 1.55 (m, 1H), 1.19 (s, 3H), 1.07 (s, 3H). **^{13}C NMR** (101 MHz, Chloroform-*d*) δ 158.9, 155.1, 128.3, 123.9, 122.7, 120.9, 111.2, 102.6, 56.0, 46.1, 34.6, 30.7, 26.9, 20.2, 13.8. **HRMS** (ESI) m/z: $(M + H)^+$ calcd. for $C_{15}H_{18}NO^+$ 228.1383, found 228.1380.

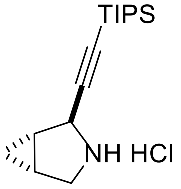

(±)-2-((Triisopropylsilyl)ethynyl)-3-azabicyclo[3.1.0]hexane hydrochloride (14a). Prepared according to the General C−H Functionalization Procedure with the corresponding lithium reagent as the nucleophile. Purification of the crude mixture by short flash column chromatography (silica gel; dichloromethane: methanol = 20:1 ~ 10:1) and formation of amine hydrochloride by adding HCl (4.0 M in EA) to afford **14a** (168 mg, 0.56 mmol) in 28% yield as a white solid. **^{1}H NMR** (400 MHz, Chloroform-*d*) δ 3.84 (s, 1H), 3.16 (dd, *J* = 10.1, 3.4 Hz, 1H), 2.95 (d, *J* = 10.1 Hz, 1H), 1.74 (d, *J* = 6.2 Hz, 2H), 1.64 – 1.53 (m, 1H), 1.48 (dt, *J* = 6.5, 3.4 Hz, 1H), 1.07 (d, *J* = 3.8 Hz, 21H), 0.44 (td, *J* = 7.7, 5.3 Hz, 1H), 0.35 (q, *J* = 4.2 Hz, 1H). **^{13}C NMR** (101 MHz, Chloroform-*d*) δ 110.5, 82.5, 50.6, 46.7, 22.6, 18.6, 15.7, 11.2, 5.7. **HRMS** (ESI) m/z: $(M + H)^+$ calcd. for $C_{16}H_{30}NSi^+$ 264.2142, found 264.2140.

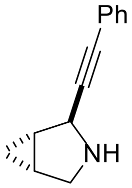

(±)-2-(Phenylethynyl)-3-azabicyclo[3.1.0]hexane (**15a**). Prepared according to the General C−H Functionalization Procedure with the corresponding lithium reagent as the nucleophile. Purification of the crude mixture by short flash column chromatography (silica gel; dichloromethane: methanol = 20:1 ~ 10:1) to afford **15a**

(158 mg, 0.86 m mol) in 36% yield as a brown oil. **^{1}H NMR** (400 MHz, Chloroform-*d*) δ 7.44 (dd, *J* = 6.0, 2.8 Hz, 2H), 7.34 – 7.27 (m, 3H), 4.09 (s, 1H), 3.24 (dd, *J* = 10.5, 3.4 Hz, 1H), 3.00 (d, *J* = 10.6 Hz, 1H), 1.89 (s, 1H), 1.72-1.67 (m, 1H), 1.59-1.53 (m, 1H), 0.54 (q, *J* = 7.3 Hz, 1H), 0.37 (q, *J* = 4.5 Hz, 1H). **^{13}C NMR** (101 MHz, Chloroform-*d*) δ 131.7, 128.2, 127.9, 123.4, 91.4, 82.5, 50.8, 47.1, 22.5, 16.3, 6.0. **HRMS** (ESI) m/z: $(M + H)^+$ calcd. for $C_{13}H_{14}N^+$ 184.1121, found 184.1120.

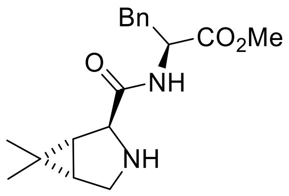

Methyl ((1*R*,2*S*,5*S*)-6,6-dimethyl-3-azabicyclo[3.1.0]hexane-2-carbonyl)-*L*-phenylalaninate (17a). The compound **17a** were prepared according to the reference.[12] To a solution of methyl *L*-phenylalaninate (0.77 g, 4.30 mmol, 1.1 equiv) in DMF (10 mL) was added (1*R*,2*S*,5*S*)-3-(tert-butoxycarbonyl)-6,6-dimethyl-3-azabicyclo[3.1.0]hexane-2-carboxylic acid (1.00 g, 3.92 mmol, 1.0 equiv), HATU (1.49 g, 3.92 mmol, 1.0 equiv) and DIEA (1.50 mL, 8.62 mmol, 2.2 equiv), and then the reaction mixture was stirred for 12 h. The reaction mixture was diluted with EtOAc, the organic phase solution was washed with 2 N HCI aqueous and saturated $NaHCO_3$ aqueous. The organic layer was dried over Na_2SO_4 and concentrated to give clear oil. The crude compound was dissolved in ethyl acetate (10 mL), HCl (0.2 mL, 4.0 M in EA) was added to the mixture, the mixture was stirred overnight, then the reaction mixture was adjusted PH with $NaHCO_3$ aqueous and extracted thrice with dichloromethane. The combined organic extracts were washed with brine, dried over Na_2SO_4 and concentrated. The crude product was purified by short flash column chromatography (silica gel; dichloromethane: methanol = 20:1 ~ 10:1) to to afford **17a** (509 mg, 1.61 mmol) in 41% yield as a white solid (two steps). **^{1}H NMR** (400 MHz, DMSO-*d*$_6$) δ 9.24 (d, *J* = 7.0 Hz, 1H), 7.35 – 7.21 (m, 5H), 4.61 – 4.52 (m, 1H), 3.96 (s, 1H), 3.65 (s, 3H), 3.55 (dd, *J* = 12.3, 6.3 Hz, 1H), 3.16 – 2.94 (m, 3H), 1.71 (t, *J* = 7.1 Hz, 1H), 1.59 – 1.56

(m, 1H), 1.06 (d, J = 2.0 Hz, 3H), 1.04 (s, 3H). **^{13}C NMR** (101 MHz, DMSO-d_6) δ 171.9, 168.6, 137.3, 129.5, 128.8, 127.2, 59.8, 54.7, 52.6, 46.3, 36.7, 35.6, 29.7, 26.3, 22.2, 14.5. **HRMS** (ESI) m/z: $(M + Na)^+$ calcd. for $C_{18}H_{24}N_2O_3Na^+$ 339.1679, found 339.1680.

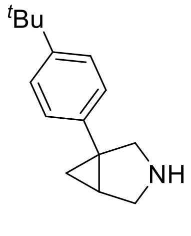

1-(4-(Tert-butyl)phenyl)-3-azabicyclo[3.1.0]hexane (**18a**). Prepared according to the synthetic procedure for the synthesis of **34a**.[13] Purification of the crude mixture by short flash column chromatography (silica gel; hexane: ethyl acetate = 1:1 to dichloromethane: methanol = 20:1) to afford **18a** (446 mg, 2.07 mmol) in 83% yield as a white solid. **^{1}H NMR** (400 MHz, Chloroform-d) δ 7.40 – 7.33 (m, 2H), 7.19 – 7.12 (m, 2H), 3.57 (d, J = 11.3 Hz, 1H), 3.48 – 3.35 (m, 3H), 1.90 – 1.83 (m, 1H), 1.33 (s, 10H), 1.30 – 1.27 (m, 1H),1.17 – 1.13 (m, 1H). **^{13}C NMR** (101 MHz, Chloroform-d) δ 149.8, 149.8, 136.7, 136.6, 126.8, 125.6, 51.6, 51.6, 48.2, 48.1, 34.5, 31.5, 31.5, 31.3, 24.2, 24.1, 15.0. **HRMS** (ESI) m/z: $(M + Na)^+$ calcd. for $C_{15}H_{21}NNa^+$ 238.1566, found 238.1563.

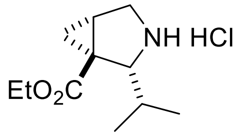

(±)-Ethyl-2-isopropyl-3-azabicyclo[3.1.0]hexane-1-carboxylate hydrochloride (**20a**). Prepared according to the synthetic procedure for the synthesis of **1a**.[1] Purification of the crude mixture by short flash column chromatography (silica gel; hexane: ethyl acetate = 1:1 to dichloromethane: methanol = 20:1) and formation of amine hydrochloride by adding HCl (4.0 M in EA) to afford **20a** (345 mg, 1.48 mmol) in 55% yield as a white solid (last step). **^{1}H NMR** (400 MHz, Chloroform-d) δ 10.49 (s, 1H), 9.31 (s, 1H), 4.26 – 4.07 (m, 3H), 3.54 – 3.43 (m, 2H), 2.23 – 2.14 (m, 1H),

2.08 – 2.01 (m, 1H), 1.78 (t, J = 7.7 Hz, 1H), 1.59 (t, J = 6.1 Hz, 1H), 1.33 – 1.25 (m, 3H), 1.22 (d, J = 6.6 Hz, 3H), 1.05 (d, J = 6.8 Hz, 3H). **^{13}C NMR** (101 MHz, Chloroform-d) δ 170.0, 66.3, 61.5, 45.7, 32.4, 30.2, 27.3, 20.6, 20.4, 14.1, 12.4. **HRMS** (ESI) m/z: $(M + H)^+$ calcd. for $C_{11}H_{20}NO_2^+$ 198.1489, found 198.1488.

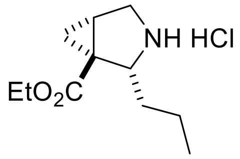

(±)-Ethyl-2-propyl-3-azabicyclo[3.1.0]hexane-1-carboxylate hydrochloride (**21a**). Prepared according to the synthetic procedure for the synthesis of **1a**.[1] Purification of the crude mixture by short flash column chromatography (silica gel; hexane: ethyl acetate = 1:1 to dichloromethane: methanol = 20:1) and formation of amine hydrochloride by adding HCl (4.0 M in EA) to afford **21a** (285 mg, 1.22 mmol) in 60% yield as a white solid (last step). **^{1}H NMR** (400 MHz, Chloroform-d) δ 10.45 (s, 1H), 9.37 (s, 1H), 4.24 – 4.15 (m, 3H), 3.50 – 3.40 (m, 2H), 2.18 – 2.10 (m, 1H), 1.95 – 1.86 (m, 2H), 1.67 – 1.47 (m, 4H), 1.28 (t, J = 7.1 Hz, 3H), 1.03 (t, J = 7.2 Hz, 3H). **^{13}C NMR** (101 MHz, Chloroform-d) δ 170.1, 61.5, 60.1, 45.6, 33.0, 31.9, 26.0, 20.2, 14.1, 13.9, 13.1. **HRMS** (ESI) m/z: $(M + H)^+$ calcd. for $C_{11}H_{20}NO_2^+$ 198.1489, found 198.1489.

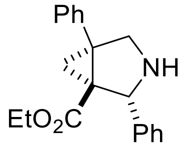

(±)-Ethyl-2,5-diphenyl-3-azabicyclo[3.1.0]hexane-1-carboxylate (22a). Prepared according to the synthetic procedure for the synthesis of **1a**.[1] Purification of the crude mixture by short flash column chromatography (silica gel; hexane: ethyl acetate = 1:1 to dichloromethane: methanol = 20:1) to afford **22a** (138 mg, 0.45 mmol) in 75% yield as a brown oil (last step). **^{1}H NMR** (400 MHz, Chloroform-d) δ 7.53 – 7.48 (m, 2H), 7.36 – 7.29 (m, 6H), 7.29 – 7.22 (m, 2H), 5.19 (s, 1H), 3.88 – 3.75 (m, 2H), 3.44 (d, J = 11.4 Hz, 1H), 3.34 (d, J = 11.4 Hz, 1H), 2.81 (s, 1H), 2.11 (d, J = 5.4

Hz, 1H), 1.66 (d, *J* = 5.4 Hz, 1H), 0.78 (t, *J* = 7.1 Hz, 3H). **^{13}C NMR** (101 MHz, Chloroform-*d*) δ 170.2, 139.4, 137.5, 129.2, 128.5, 128.4, 127.8, 127.5, 127.4, 63.4, 60.4, 55.6, 44.5, 41.7, 14.6, 13. 8. **HRMS** (ESI) m/z: $(M + Na)^+$ calcd. for $C_{20}H_{22}NO_2^+$ 308.1645, found 308.1640.

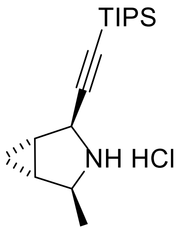

(±)-2-Methyl-4-((triisopropylsilyl)ethynyl)-3-azabicyclo[3.1.0]hexane hydrochloride (24a). Prepared according to the General C−H Functionalization Procedure from (±)-2-((triisopropylsilyl)ethynyl)-3-azabicyclo[3.1.0]hexane hydrochloride **14a** with the corresponding Grignard's reagent as the nucleophile. Purification of the crude mixture by short flash column chromatography (silica gel; dichloromethane: methanol = 20:1 ~ 10:1) and formation of amine hydrochloride by adding HCl (4.0 M in EA) to afford **24a** (277 mg, 0.88 mmol) in 44% yield as a white solid (last step). **^{1}H NMR** (400 MHz, Chloroform-*d*) δ 10.32 (s, 1H), 9.86 (s, 1H), 4.53 (s, 1H), 3.96 (q, *J* = 6.9 Hz, 1H), 1.96 – 1.92 (m, 1H), 1.72 (d, *J* = 6.9 Hz, 3H), 1.69 – 1.64 (m, 1H), 1.22 – 1.18 (m, 1H), 1.11 (s, 21H), 0.79 – 0.73 (m, 1H). **^{13}C NMR** (101 MHz, Chloroform-*d*) δ 101.2, 91.0, 57.3, 49.7, 22.6, 22.4, 19.5, 18.5, 11.1, 7.0. **HRMS** (ESI) m/z: $(M + H)^+$ calcd. for $C_{17}H_{32}NSi^+$ 278.2299, found 278.2290.

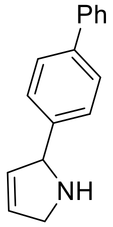

2-([1,1'-Biphenyl]-4-yl)-2,5-dihydro-1*H*-pyrrole (**25a**). The compound **25a** were prepared according to the reference.[14] To a solution of tert-butyl (1-([1,1'-biphenyl]-4-yl)allyl)(allyl)carbamate (500 mg, 1.43 mmol) was added

(1,3-Bis(2,4,6-trimethylphenyl)-2-imidazolidinylidene)dichloro(phenylmethylene)(tricyclohexylphosphine)ruthenium (121 mg, 0.143 mmol) under Ar at room temperature. The reaction mixture was concentrated and purified by flash column chromatography (silica gel; dichloromethane: methanol = 40:1) to afford the crude product tert-butyl 2-([1,1'-biphenyl]-4-yl)-2,5-dihydro-1H-pyrrole-1-carboxylate. The crude compound was dissolved in ethyl acetate, HCl (0.2 mL, 4.0 M in EA) was added to the mixture, the mixture was stirred overnight and washed with saturated $NaHCO_3$ aqueous. The organic layer was concentrated and purified by short flash column chromatography (silica gel; hexane: ethyl acetate = 1:1 to dichloromethane: methanol = 20:1) to afford **25a** (149 mg, 0.67 mmol) in 47% yield as a brown oil (two steps). **^{1}H NMR** (400 MHz, Chloroform-*d*) δ 7.62 – 7.57 (m, 4H), 7.48 – 7.44 (m, 2H), 7.41 – 7.34 (m, 3H), 6.09 – 6.06 (m, 1H), 5.94 – 5.91 (m, 1H), 5.14 – 5.11 (m, 1H), 4.86 (s, 1H), 4.08 – 3.99 (m, 1H), 3.92 – 3.87 (m, 1H). **^{13}C NMR** (101 MHz, Chloroform-*d*) δ 143.5, 141.0, 140.3, 132.0, 129.2, 128.7, 127.4, 127.4, 127.2, 127.1, 68.8, 54.2. **HRMS** m/z (ESI) calcd. for $C_{16}H_{16}N^{+}$ $(M + H)^{+}$ 222.1277, found 222.1275.

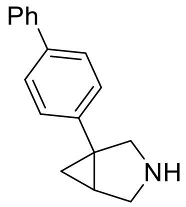

1-([1,1'-Biphenyl]-4-yl)-3-azabicyclo[3.1.0]hexane (**26a**). Prepared according to the synthetic procedure for the synthesis of **34a**.[13] Purification of the crude mixture by short flash column chromatography (silica gel; hexane: ethyl acetate = 1:1 to dichloromethane: methanol = 20:1) to afford **26a** (340 mg, 1.44 mmol) in 88% yield as a white solid. **^{1}H NMR** (400 MHz, Chloroform-*d*) δ 7.63 – 7.53 (m, 4H), 7.49 – 7.42 (m, 2H), 7.40 – 7.34 (m, 1H), 7.30 (d, *J* = 8.2 Hz, 2H), 3.46 – 3.42 (m, 1H), 3.38 – 3.26 (m, 2H), 3.24 – 3.20 (m, 1H), 1.86 – 1.82 (m, 1H), 1.12 – 1.08 (m, 2H). **^{13}C NMR** (101 MHz, Chloroform-*d*) δ 140.8, 140.5, 139.3, 139.2, 128.8, 127.4, 127.2, 127.0, 52.6, 48.9, 32.3, 25.6, 15.1. **HRMS** (ESI) m/z: $(M + H)^{+}$ calcd. for $C_{17}H_{18}N^{+}$ 236.1434, found 236.1436.

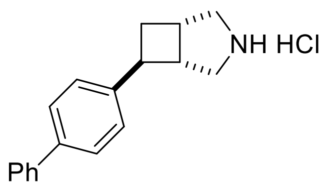

(±)-6-([1,1'-Biphenyl]-4-yl)-3-azabicyclo[3.2.0]heptane hydrochloride (**27a**). The compound **27a** was prepared according to the reference.[3] Purification of the crude mixture by short flash column chromatography (silica gel; hexane: ethyl acetate = 1:1 to dichloromethane: methanol = 20:1) and formation of amine hydrochloride by adding HCl (4.0 M in EA) to afford **27a** (98 mg, 0.34 mmol) in 81% yield as a white solid (last step). **^{1}H NMR** (400 MHz, Chloroform-*d*) δ 10.44 (s, 2H), 7.63 – 7.55 (m, 4H), 7.49 – 7.42 (m, 2H), 7.40 – 7.33 (m, 3H), 3.77 – 3.67 (m, 3H), 3.42 (dd, *J* = 12.1, 6.9 Hz, 1H), 3.38 – 3.21 (m, 3H), 2.66 – 2.45 (m, 2H). **^{13}C NMR** (101 MHz, Chloroform-*d*) δ 143.0, 140.8, 139.6, 128.8, 127.3, 127.2, 127.0, 126.9, 51.5, 45.3, 40.8, 33.8, 31.0. **HRMS** (ESI) m/z: $(M + H)^+$ calcd. for $C_{18}H_{20}N^+$ 250.1590, found 250.1582.

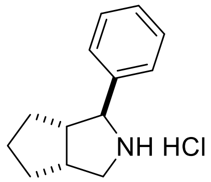

(±)-1-Phenyloctahydrocyclopenta[c]pyrrole hydrochloride (**28a**). The compound **28a** is a known compound which was prepared according to the General C−H Functionalization Procedure with the corresponding Grignard's reagent as the nucleophile. Purification of the crude mixture by short flash column chromatography (silica gel; dichloromethane: methanol = 20:1 ~ 10:1) and formation of amine hydrochloride to afford **28a** (681 mg, 3.04 mmol) in 45% yield as a white solid. **^{1}H NMR** (400 MHz, Chloroform-*d*) δ 10.04 (s, 1H), 9.58 (s, 1H), 7.66 – 7.54 (m, 2H), 7.43 – 7.30 (m, 3H), 3.88 (td, *J* = 10.2, 3.9 Hz, 1H), 3.49 – 3.35 (m, 1H), 3.07 – 2.88 (m, 2H), 2.65 – 2.50 (m, 1H), 1.78 – 1.49 (m, 6H). **^{13}C NMR** (101 MHz,

Chloroform-*d*) δ 134.1, 129.2, 129.0, 128.4, 68.6, 50.9, 49.7, 41.5, 31.4, 29.8, 24.7. **HRMS** (ESI) m/z: $(M + H)^+$ calcd. for $C_{13}H_{18}N^+$ 188.1434, found 188.1436.

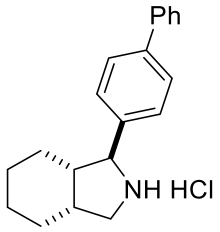

(±)-1-([1,1'-Biphenyl]-4-yl)octahydro-1*H*-isoindole hydrochloride (**29a**). Prepared according to the General C−H Functionalization Procedure with the corresponding Grignard's reagent as the nucleophile. Purification of the crude mixture by short flash column chromatography (silica gel; dichloromethane: methanol = 20:1 ~ 10:1) and formation of amine hydrochloride to afford **29a** (540 mg, 1.72 mmol) in 52% yield as a white solid. **^{1}H NMR** (400 MHz, Chloroform-*d*) δ 10.14 (s, 1H), 9.63 (s, 1H), 7.69 (d, *J* = 8.0 Hz, 2H), 7.63 – 7.50 (m, 4H), 7.42 (t, *J* = 7.4 Hz, 2H), 7.37 (t, *J* = 7.2 Hz, 1H), 4.02 – 3.88 (m, 1H), 3.50 – 3.36 (m, 1H), 3.02 (dq, *J* = 11.6, 7.2 Hz, 2H), 2.62 (s, 1H), 1.82 – 1.63 (m, 5H), 1.50 – 1.43 (m, 1H), 1.39 – 1.25 (m, 2H). **^{13}C NMR** (101 MHz, Chloroform-*d*) δ 141.7, 140.3, 134.5, 128.8, 128.7, 127.5, 127.5, 127.0, 62.6, 50.0, 44.5, 37.2, 27.0, 24.2, 23.3, 20.7. **HRMS** (ESI) m/z: $(M + H)^+$ calcd. for $C_{20}H_{24}N^+$ 278.1903, found 278.1902.

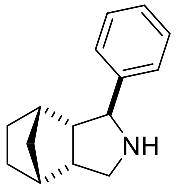

(±)-1-Phenyloctahydro-1*H*-4,7-methanoisoindole hydrochloride (30a). Prepared according to the General C−H Functionalization Procedure with phenyllithium as the nucleophile with Grignard's reagent as the nucleophile from (±) -2,3,3a,4,7,7a-hexahydro-1*H*-4,7-methanoisoindole. Purification of the crude mixture by short flash column chromatography (silica gel; dichloromethane: methanol = 20:1 ~ 10:1) to afford (±)-1-phenyl-2,3,3a,4,7,7a-hexahydro-1*H*-4,7-methanoisoindole in

32% yield and subsequent Pd/C hydrogenation with a H_2 baloon to afford **30a** (183 mg, 0.86 mmol) quantitatively as a brown oil. **^{1}H NMR** (400 MHz, Chloroform-*d*) δ 7.44 – 7.39 (m, 2H), 7.37 – 7.32 (m, 2H), 7.28 – 7.23 (m, 1H), 3.59 (d, *J* = 7.8 Hz, 1H), 3.41 (dd, *J* = 10.2, 8.3 Hz, 1H), 2.52 (dd, *J* = 10.3, 8.3 Hz, 1H), 2.28 – 2.18 (m, 2H), 2.12 – 2.11 (m, 1H), 1.89 (t, *J* = 8.2, 1.4 Hz, 1H), 1.81 (d, *J* = 4.0 Hz, 1H), 1.68 – 1.63 (m, 1H), 1.60 – 1.49 (m, 2H), 1.19 – 1.03 (m, 3H). **^{13}C NMR** (101 MHz, Chloroform-*d*) δ 144.4, 128.3, 126.9, 126.7, 68.0, 57.5, 52.0, 49.2, 39.7, 39.1, 32.6, 28.5, 28.4. **HRMS** (ESI) m/z: $(M + H)^+$ calcd. for $C_{15}H_{20}N^+$ 214.1590, found 214.1594.

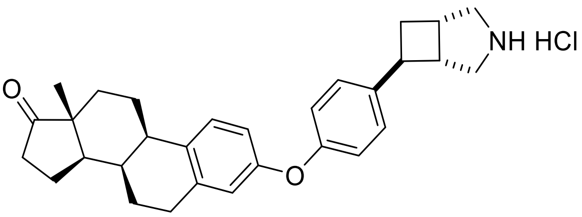

(±)-(8*R*,9*R*,13*R*,14*R*)-3-(4-(-3-Azabicyclo[3.2.0]heptan-6-yl)phenoxy)-13-methyl-6,7,8,9,11,12,13,14,15,16-decahydro-17*H*-cyclopenta[a]phenanthren-17-one hydrochloride (31a). Prepared according to the synthetic procedure for the synthesis of **35a**.[3] Purificationf the crude mixture by short flash column chromatography (silica gel; hexane: ethyl acetate = 1:1 to dichloromethane: methanol = 20:1) and formation of amine hydrochloride by adding HCl (4.0 M in EA) to afford **31a** (190 mg, 0.40 mmol) in 78% yield as a white solid (last step). **^{1}H NMR** (400 MHz, Chloroform-*d*) δ 10.43 (s, 2H), 7.24 (t, *J* = 8.9 Hz, 3H), 7.01 – 6.96 (m, 2H), 6.80 (dd, *J* = 8.5, 2.7 Hz, 1H), 6.75 (d, *J* = 2.6 Hz, 1H), 3.68 (t, *J* = 12.4 Hz, 3H), 3.24 (s, 2H), 2.89 (dd, *J* = 8.1, 3.1 Hz, 2H), 2.59 – 2.49 (m, 2H), 2.48 – 2.38 (m, 2H), 2.35 – 2.26 (m, 1H), 2.23 – 2.14 (m, 1H), 2.13 – 1.96 (m, 4H), 1.71 – 1.40 (m, 7H), 0.94 (s, 3H). **^{13}C NMR** (101 MHz, Chloroform-*d*) δ 156.0, 155.2, 138.6, 138.2, 134.7, 127.6, 126.6, 118.9, 118.7, 116.1, 51.5, 50.5, 48.0, 45.5, 44.1, 40.5, 38.2, 35.9, 33.7, 31.6, 31.2, 29.5, 26.5, 25.9, 21.6, 13.9. **HRMS** (ESI) m/z: $(M + Na)^+$ calcd. for $C_{30}H_{35}NO_2Na^+$ 464.2560, found 464.2553.

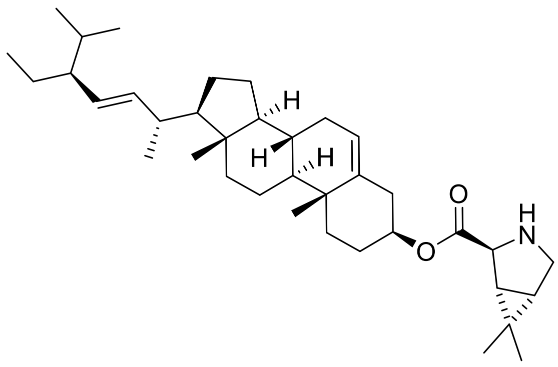

(3*S*,8*S*,9*S*,10*R*,13*R*,14*S*,17*R*)-17-((2*R*,5*S*,*E*)-5-Ethyl-6-methylhept-3-en-2-yl)-10,13-dimethyl-2,3,4,7,8,9,10,11,12,13,14,15,16,17-tetradecahydro-1*H*-cyclopenta[a]phenanthren-3-yl(1*R*,2*S*,5*S*)-6,6-dimethyl-3-azabicyclo[3.1.0]hexane-2-carboxylate (32a). The synthetic procedure was according to the reference.[12] (1*R*,2*S*,5*S*)-3-(tert-butoxycarbonyl)-6,6-dimethyl-3-azabicyclo[3.1.0]hexane-2-carboxylic acid (cas: 1373205-30-1, 1.0 g, 3.92 mmol, 1.0 equiv) was dissolved in DCM (10 mL), DMAP (527 mg, 4.31 mmol, 1.1 equiv), (3*S*,8*S*,9*S*,10*R*,13*R*,14*S*,17*R*)-17-((2*R*,5*S*,*E*)-5-ethyl-6-methylhept-3-en-2-yl)-10,13-dimethyl-2,3,4,7,8,9,10,11,12,13,14,15,16,17-tetradecahydro-1*H*-cyclopenta[a]-phenanthren-3-ol (cas: 83-48-7, 1.94g, 4.70 mmol, 1.2 equiv) were added and the reaction mixture was stirred at rt overnight. The reaction mixture was then filtered, washed with aqueous 5% HCl, aqueous 5% $NaHCO_3$, brine, dried over Na_2SO_4 and concentrate. The crude compound was dissolved in ethyl acetate, HCl (0.2 mL, 4.0 M in EA) was added to the mixture, the mixture was stirred overnight, then the reaction mixture was adjusted PH with $NaHCO_3$ aqueous and extracted with dichloromethane. The combined organic extracts were washed with brine, dried over Na_2SO_4 and concentrated. The crude product was purified by short flash column chromatography (silica gel; hexane: ethyl acetate = 1:1 to dichloromethane: methanol = 20:1) to afford **32a** (1.10 g, 2.00 mmol) in 48% yield as white solid (two steps). **^{1}H NMR** (400 MHz, Chloroform-*d*) δ 5.46 – 5.32 (m, 1H), 5.18 – 5.12 (m, 1H), 5.04 – 4.98 (m, 1H), 4.76-4.70 (m, 1H), 4.18 (s, 1H), 3.96 (dd, *J* = 12.5, 6.1 Hz, 1H), 3.34 (d, *J* = 12.4 Hz, 1H), 2.39 (d, *J* = 8.1 Hz, 2H), 2.11 – 1.81 (m, 5H), 1.76 – 1.64 (m, 4H), 1.60 – 1.39 (m, 7H), 1.35 – 0.75 (m, 27H), 0.69 (s, 3H). **^{13}C NMR** (101 MHz, Chloroform-*d*) δ

168.2, 139.1, 138.4, 129.5, 123.5, 77.4, 60.1, 56.9, 56.1, 51.4, 50.1, 46.5, 42.4, 40.6, 39.8, 37.9, 37.1, 36.7, 34.2, 32.1, 32.0, 32.0, 29.9, 29.0, 27.6, 26.3, 25.5, 24.5, 22.8, 21.4, 21.2, 21.2, 19.4, 19.2, 14.6, 12.4, 12.2. **HRMS** (ESI) m/z: $(M + H)^+$ calcd. for $C_{37}H_{60}NO_2{}^+$ 550.4619, found 550.4611.

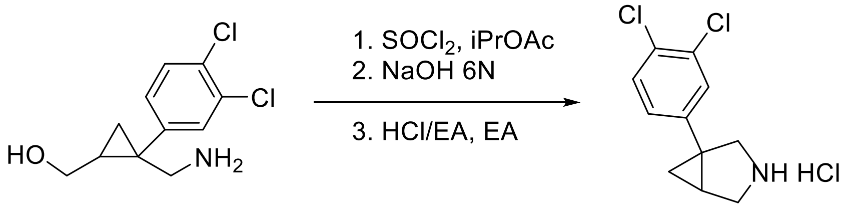

34a

34a is a known compound which was prepared from ethyl benzoylacetate and allylamine according to the reference.[13] To a solution of $SOCl_2$ (2.6 g, 21.6 mmol, 2.0 equiv) in *i*-PrOAc (10 mL) at ambient temperature over 30 min was slowly added the (2-(aminomethyl)-2-(3,4-dichlorophenyl)cyclopropyl)methanol (cas: 1004551-77-2, 2.7 g, 10.8 mmol, 1.0 equiv) in *i*-PrOAc (20 mL). After additional 5 h, 5.0 N NaOH (9.0 mL) was added over 1 h while the internal temperature was maintained at < 30 ^{O}C. The reaction mixture was stirred for 1 h at rt to allow pH to stabilize (usually to 8.5-9.0) with NaOH pH titration. the aqueous layer was extracted with ethyl acetate and the organic phase was then washed with saturated brine, dried over Na_2SO_4, concentrated. The crude product was purified by short flash column chromatography (silica gel; hexane: ethyl acetate = 1:1 to dichloromethane: methanol = 20:1) to afford crude product **34a** (1.23 g, about 50% yield, two steps). To the solution of **34a** in ethyl acetate was added HCl (4.0 M in EA), the mixture was stirred overnight and filtered to afford **34a**•HCl (1.34 g, total 47% yield, three steps).

1-(3,4-Dichlorophenyl)-3-azabicyclo[3.1.0]hexane (**34a**). **^{1}H NMR** (400 MHz, Chloroform-*d*) δ 10.34 (s, 1H), 9.87 (s, 1H), 7.42 (dd, *J* = 8.3, 1.0 Hz, 1H), 7.30 (d, *J* = 2.2 Hz, 1H), 7.06 (dd, *J* = 8.3, 2.2 Hz, 1H), 3.82 – 3.77 (m, 1H), 3.67 – 3.56 (m, 3H), 2.01 (dt, *J* = 8.4, 4.1 Hz, 1H), 1.65 (dd, *J* = 6.7, 4.7 Hz, 1H), 1.25 (t, *J* = 7.6 Hz,

1H). **^{13}C NMR** (101 MHz, Chloroform-*d*) δ 138.3, 133.0, 131.7, 130.8, 129.3, 126.6, 50.4, 47.5, 30.7, 23.5, 15.7.

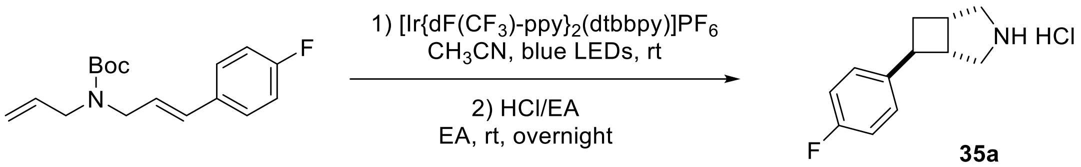

35a

35a is a known compound which was prepared from ethyl benzoylacetate and allylamine according to the reference.[3] The tert-butyl (*E*)-allyl(3-(4-fluorophenyl)allyl)carbamate (cas: 2075675-18-0, 500 mg, 1.72 mmol, 1.0 equiv) and [Ir{dF(CF_3)-ppy}2(dtbbpy)]PF_6 (1 mol %) were placed in Schlenk tube followed by acetonitrile. The reaction mixture was degassed by using Double row pipe and irradiated with Blue LED for 48 h. The crude mixture were evaporated in vacuo and was purified by short flash column chromatography (silica gel; petroleum ether: ethyl acetate = 10: 1) to afford (±)-tert-butyl-6-(4-fluorophenyl)-3-azabicyclo[3.2.0]heptane-3-carboxylate (220 mg, 0.76 mmol) in 44% yield. To the solution of (±) -tert-butyl-6-(4-fluorophenyl)-3-azabicyclo[3.2.0]heptane-3-carboxylate (220 mg, 0.76 mml, 1.0 equiv) in ethyl acetate was added HCl (0.4 mL, 4.0 M in EA, 2.1 equiv) at 0 ^{o}C, the mixture was stirred overnight and then allow pH to stabilize (9.0) with $NaHCO_3$ pH titration. the organic phase was was extracted with ethyl acetate, washed with saturated brine, dried over Na_2SO_4, concentrated. The crude product was purified by short flash column chromatography (silica gel; hexane: ethyl acetate = 1:1 to dichloromethane: methanol = 20: 1) to afford **35a** (130 mg, 0.68 mmol) in 90% yield. To the solution of **35a** in ethyl acetate was added HCl (0.2 mL, 4.0 M in EA), the mixture was stirred overnight and filtered to afford **35a**•HCl (122 mg, 0.54 mmol) in 71% yield as a white solid. The spectral data were seen in the the reference.[3]

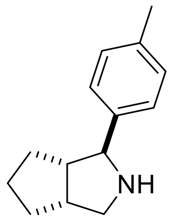

(±)-1-(*p*-Tolyl)octahydrocyclopenta[c]pyrrole (**36a**). Prepared according to the General C−H Functionalization Procedure with the corresponding Grignard's reagent as the nucleophile. Purification of the crude mixture by short flash column chromatography (silica gel; dichloromethane: methanol = 20:1 ~ 10:1) to afford **36a** (450 mg, 2.24 mmol) in 54% yield as a brown oil. **^{1}H NMR** (400 MHz, Chloroform-*d*) δ 7.36 (d, *J* = 8.0 Hz, 2H), 7.17 (d, *J* = 7.8 Hz, 2H), 3.96 (s, 1H), 3.62 (d, *J* = 8.4 Hz, 1H), 3.43 (dd, *J* = 10.6, 8.4 Hz, 1H), 2.87 – 2.81 (m, 1H), 2.64 – 2.57 (m, 1H), 2.56 – 2.50 (m, 1H), 2.36 (s, 3H), 1.74 – 1.45 (m, 6H). **^{13}C NMR** (101 MHz, Chloroform-*d*) δ 137.9, 137.4, 129.3, 127.3, 69.4, 53.1, 51.9, 43.4, 31.7, 30.5, 25.0, 21.1. **HRMS** (ESI) m/z: $(M + H)^{+}$calcd. for $C_{14}H_{20}N^{+}$ 202.1590, found 202.1595.

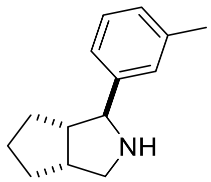

(±)-1-(*m*-Tolyl)octahydrocyclopenta[c]pyrrole (**37a**). Prepared according to the General C−H Functionalization Procedure with the corresponding Grignard's reagent as the nucleophile. Purification of the crude mixture by short flash column chromatography (silica gel; dichloromethane: methanol = 20:1 ~ 10:1) to afford **37a** (480 mg, 2.38 mmol) in 50% yield as a brown oil. **^{1}H NMR** (400 MHz, Chloroform-*d*) δ 7.33 (dd, *J* = 6.4, 1.5 Hz, 2H), 7.28 – 7.23 (m, 1H), 7.17 – 7.13 (m, 1H), 3.76 (d, *J* = 9.2 Hz, 1H), 3.46 (dd, *J* = 11.2, 8.5 Hz, 1H), 3.01 – 2.90 (m, 1H), 2.85 – 2.78 (m, 1H), 2.59 (dd, *J* = 11.3, 8.6 Hz, 1H), 2.36 (s, 3H), 1.79 – 1.64 (m, 3H), 1.64 – 1.57 (m, 2H), 1.54 – 1.50 (m, 1H). **^{13}C NMR** (101 MHz, Chloroform-*d*) δ 138.7, 136.5, 129.5, 128.9, 128.5, 124.9, 69.1, 51.8, 50.6, 42.2, 31.5, 30.1, 24.8, 21.4. **HRMS** (ESI) m/z: $(M + H)^{+}$ calcd. for $C_{14}H_{20}N^{+}$ 202.1590, found 202.1591.

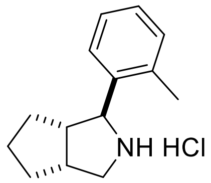

(±)-1-(*o*-Tolyl)octahydrocyclopenta[c]pyrrole hydrochloride (**38a**). Prepared according to the General C−H Functionalization Procedure with the corresponding Grignard's reagent as the nucleophile. Purification of the crude mixture by short flash column chromatography (silica gel; dichloromethane: methanol = 20:1 ~ 10:1) and formation of amine hydrochloride by adding HCl (4.0 M in EA) to afford **38a** (370 mg, 1.56 mmol) in 47% yield as a white solid. **^{1}H NMR** (400 MHz, Chloroform-*d*) δ 9.98 (s, 1H), 9.37 (s, 1H), 7.83 (d, *J* = 7.2 Hz, 1H), 7.27 – 7.11 (m, 3H), 4.26 – 4.20 (m, 1H), 3.43 – 3.37 (m, 1H), 3.08 – 2.86 (m, 2H), 2.65 – 2.52 (m, 1H), 2.46 (s, 3H), 1.75 – 1.47 (m, 6H). **^{13}C NMR** (101 MHz, Chloroform-*d*) δ 137.2, 132.8, 130.8, 128.9, 127.8, 127.0, 63.8, 51.1, 50.9, 41.7, 31.3, 29.9, 24.8, 20.1. **HRMS** (ESI) m/z: (M + H)$^+$ calcd. for $C_{14}H_{20}N^+$ 202.1590, found 202.1593.

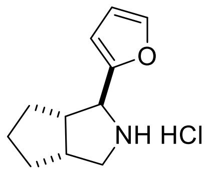

(±)-1-(Furan-2-yl)octahydrocyclopenta[c]pyrrole hydrochloride (**39a**). Prepared according to the General C−H Functionalization Procedure with the corresponding lithium reagent as the nucleophile. Purification of the crude mixture by short flash column chromatography (silica gel; dichloromethane: methanol = 20:1 ~ 10:1) and formation of amine hydrochloride by adding HCl (4.0 M in EA) to afford **39a** (454 mg, 2.12 mmol) in 50% yield as a white solid. **^{1}H NMR** (400 MHz, Chloroform-*d*) δ 10.18 (s, 1H), 9.66 (s, 1H), 7.44 (s, 1H), 6.58 (d, *J* = 3.2 Hz, 1H), 6.33 (t, *J* = 2.3 Hz, 1H), 4.26 – 4.09 (m, 1H), 3.59 (q, *J* = 6.1 Hz, 1H), 3.11 – 2.9 (m, 2H), 2.84 – 2.76 (m, 1H), 1.81 – 1.54 (m, 6H). **^{13}C NMR** (101 MHz, Chloroform-*d*) δ 147.5, 143.8, 110.8,

110.6, 61.1, 50.8, 47.4, 41.5, 31.5, 30.4, 25.1. **HRMS** (ESI) m/z: $(M + H)^+$ calcd. for $C_{11}H_{16}NO^+$ 178.1226, found 178.1230.

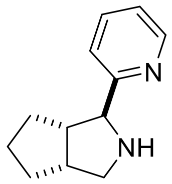

(±)-1-(Pyridin-2-yl)octahydrocyclopenta[c]pyrrole (**40a**). Prepared according to the General C−H Functionalization Procedure with the corresponding lithium reagent as the nucleophile. Purification of the crude mixture by short flash column chromatography (silica gel; dichloromethane: methanol = 20:1 ~ 10:1) to afford **40a** (290 mg, 1.54 mmol) in 48% yield as a brown oil, 290 mg, 48% yield. **^{1}H NMR** (400 MHz, Chloroform-*d*) δ 8.57 (d, *J* = 4.9 Hz, 1H), 7.65 (td, *J* = 7.7, 1.7 Hz, 1H), 7.34 (d, *J* = 7.8 Hz, 1H), 7.17 (dd, *J* = 7.5, 4.9 Hz, 1H), 3.68 (d, *J* = 7.5 Hz, 1H), 3.47 (dd, *J* = 11.0, 8.3 Hz, 1H), 2.84 – 2.71 (m, 1H), 2.61 – 2.52 (m, 2H), 2.37 (s, 1H), 1.74 – 1.57 (m, 5H), 1.52 (dt, *J* = 10.9, 3.4 Hz, 1H). **^{13}C NMR** (101 MHz, Chloroform-*d*) δ 162.5, 149.3, 136.4, 122.0, 121.7, 71.4, 54.6, 52.8, 45.3, 32.0, 31.1, 25.1. **HRMS** (ESI) m/z: $(M + H)^+$ calcd. for $C_{12}H_{17}N2^+$ 189.1386, found 189.1386.

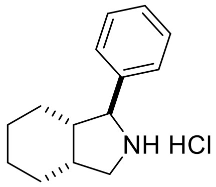

(±)-1-Phenyloctahydro-1*H*-isoindole hydrochloride (**41a**). Prepared according to the General C−H Functionalization Procedure with the corresponding Grignard's reagent as the nucleophile. Purification of the crude mixture by short flash column chromatography (silica gel; dichloromethane: methanol = 20:1 ~ 10:1) and formation of amine hydrochloride to afford **41a** (560 mg, 2.36 mmol) in 44% yield as a white solid. **^{1}H NMR** (400 MHz, Chloroform-*d*) δ 10.39 (s, 1H), 9.22 (s, 1H), 7.48 (d, *J* = 7.2 Hz, 2H), 7.36 (q, *J* = 6.7 Hz, 3H), 4.46 (d, *J* = 11.1 Hz, 1H), 3.49 (dd, *J* = 11.9, 6.7 Hz, 1H), 2.82 (dd, *J* = 11.8, 2.4 Hz, 1H), 2.47 – 2.42 (m, 1H), 2.36 – 2.30 (m, 1H),

1.74 – 1.62 (m, 2H), 1.60 – 1.34 (m, 5H), 1.27 (t, *J* = 12.2 Hz, 1H). ^{13}**C NMR** (101 MHz, Chloroform-*d*) δ 133.9, 129.2, 129.0, 128.3, 63.2, 49.9, 44.4, 36.8, 26.9, 24.1, 23.1, 20.5. **HRMS** (ESI) m/z: $(M + H)^+$ calcd. for $C_{14}H_{20}N^+$ 202.1590, found 202.1589.

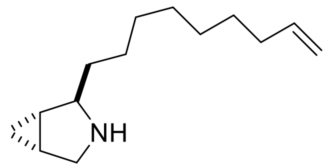

(±)-2-(Non-8-en-1-yl)-3-azabicyclo[3.1.0]hexane (42a). Prepared according to the General C−H Functionalization Procedure with the corresponding Grignard's reagent as the nucleophile. Purification of the crude mixture by short flash column chromatography (silica gel; dichloromethane: methanol = 20:1 ~ 10:1) to afford **42a** (348 mg, 1.68 mmol) in 43% yield as a colorless oil. ^{1}**H NMR** (400 MHz, Chloroform-*d*) δ 5.86 – 5.76 (m, 6.6 Hz, 1H), 5.05 – 4.89 (m, 2H), 2.99 – 2.86 (m, 2H), 2.82 (d, *J* = 11.3 Hz, 1H), 2.10 – 1.99 (m, 2H), 1.75 (s, 1H), 1.51 – 1.19 (m, 14H), 0.49 – 0.44 (m, 1H), 0.08 (q, *J* = 4.2 Hz, 1H). ^{13}**C NMR** (101 MHz, Chloroform-*d*) δ 139.4, 114.2, 60.4, 47.3, 35.9, 34.0, 29.9, 29.7, 29.3, 29.1, 27.0, 21.8, 16.8, 6.8. **HRMS** (ESI) m/z: $(M + H)^+$ calcd. for $C_{14}H_{26}N^+$ 208.2060, found 208.2063.

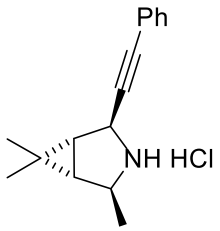

(±)-2,6,6-Trimethyl-4-(phenylethynyl)-3-azabicyclo[3.1.0]hexane hydrochloride (**43a**). Prepared according to the General C−H Functionalization Procedure with the corresponding Grignard's reagent as the nucleophile from (±)-6,6-dimethyl-2-(phenylethynyl)-3-azabicyclo[3.1.0]hexane. Purification of the crude mixture by short flash column chromatography (silica gel; dichloromethane:

methanol = 20:1 ~ 10:1) and formation of amine hydrochloride to afford **43a** (142 mg, 0.54 mmol) in 18% yield as a white solid. **^{1}H NMR** (400 MHz, Chloroform-*d*) δ 7.44 – 7.42 (m, 2H), 7.32 – 7.28 (m, 3H), 4.03 (s, 1H), 3.30 (q, *J* = 6.5 Hz, 1H), 2.10 – 2.03 (m, 2H), 1.64 (d, *J* = 7.1 Hz, 1H), 1.36 (d, *J* = 6.5 Hz, 3H), 1.26 (d, *J* = 7.1 Hz, 1H), 1.16 (s, 3H), 1.04 (s, 3H). **^{13}C NMR** (101 MHz, Chloroform-*d*) δ 131.5, 128.1, 127.8, 123.6, 93.5, 82.0, 54.0, 48.7, 37.2, 36.3, 27.0, 24.7, 19.5, 13.9. **HRMS** (ESI) m/z: $(M + H)^+$ calcd. for $C_{15}H_{20}N^+$ 226.1590, found 226.1588.

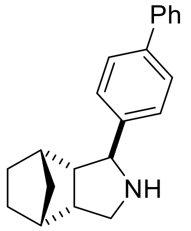

(±)-1-([1,1'-Biphenyl]-4-yl)octahydro-1*H*-4,7-methanoisoindole (44a). Prepared according to the General C−H Functionalization Procedure with the corresponding Grignard's reagent as the nucleophile from (±) -2,3,3a,4,7,7a-hexahydro-1*H*-4,7-methanoindene. Purification of the crude mixture by short flash column chromatography (silica gel; dichloromethane: methanol = 20:1 ~ 10:1) to afford (±)-1-([1,1'-biphenyl]-4-yl)-2,3,3a,4,7,7a-hexahydro-1*H*-4,7-methanoisoindole in 50% yield and subsequent Pd/C hydrogenation with a H_2 baloon to afford **44a** (687 mg, 2.37 mmol) quantitatively as a white solid. **^{1}H NMR** (400 MHz, Chloroform-*d*) δ 7.62 – 7.52 (m, 4H), 7.48 – 7.42 (m, 4H), 7.37 – 7.30 (m, 1H), 3.62 (d, *J* = 7.8 Hz, 1H), 3.41 (dd, *J* = 10.2, 8.4 Hz, 1H), 2.52 (dd, *J* = 10.2, 8.4 Hz, 1H), 2.32 – 2.17 (m, 2H), 2.11 (t, *J* = 2.2 Hz, 1H), 1.91 (t, *J* = 8.4 Hz, 1H), 1.66 (d, *J* = 10.3 Hz, 1H), 1.53 (dt, *J* = 7.6, 2.5 Hz, 2H), 1.14 – 1.09 (m, 3H). **^{13}C NMR** (101 MHz, Chloroform-*d*) δ 143.6, 141.1, 139.9, 128.7, 127.2, 127.1, 127.1, 127.1, 67.7, 57.5, 52.1, 49.2, 39.8, 39.2, 32.7, 28.5, 28.4. **HRMS** (ESI) m/z: $(M + H)^+$ calcd. for $C_{21}H_{24}N^+$ 290.1903, found 290.1903.

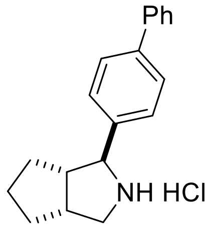

(±)-1-([1,1'-Biphenyl]-4-yl)octahydrocyclopenta[c]pyrrole hydrochloride (45a). Prepared according to the General C−H Functionalization Procedure with the corresponding Grignard’s reagent as the nucleophile. Purification of the crude mixture by short flash column chromatography (silica gel; dichloromethane: methanol = 20:1 ~ 10:1) and formation of amine hydrochloride by adding HCl (4.0 M in EA) to afford **45a** (4.20 g, 14.01 mmol) in 70% yield as a white solid**.** ^{1}**H NMR** (400 MHz, DMSO-d_6) δ 9.82 (t, *J* = 78.0 Hz, 2H), 7.74 – 7.69 (m, 6H), 7.49 (t, *J* = 7.5 Hz, 2H), 7.40 (t, *J* = 7.3 Hz, 1H), 4.13 (d, *J* = 9.8 Hz, 1H), 3.62 (dd, *J* = 11.4, 8.6 Hz, 1H), 3.02 – 2.95 (m, 1H), 2.91 – 2.73 (m, 2H), 1.85 – 1.52 (m, 6H). ^{13}**C NMR** (101 MHz, DMSO-d_6) δ 141.1, 139.9, 134.9, 129.5, 129.1, 128.2, 127.5, 127.2, 67.0, 50.7, 49.5, 41.9, 31.3, 29.7, 25.0. **HRMS** (ESI) m/z: $(M + H)^+$ calcd. for $C_{19}H_{22}N^+$ 264.1747, found 264.1738.

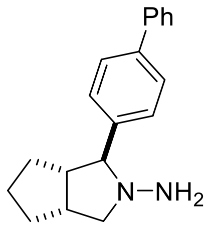

(±)-1-([1,1'-Biphenyl]-4-yl)hexahydrocyclopenta[c]pyrrol-2(1*H*)-amine (45d). **45d** were prepared from (±)-1-([1,1'-biphenyl]-4-yl)octahydrocyclopenta[c]pyrrole (**45a**) according to the reference.[15] A solution of **45a** (300 mg, 1.1 mol) in a mixture of hydrochloric acid (0.2 mL) and H_2O (1.4 mL) was cooled in an ice bath and treated dropwise with a solution of sodium nitrite (97 mg, 1.4 mol) in H_2O. The reaction mixture was stirred for 2 h at room temperature. The reaction mixture was extrated with DCM, washed with water and sodium bicarbonate solution and concentrated to afford (±)-1-([1,1'-biphenyl]-4-yl)-2-nitrosooctahydrocyclopenta[c]pyrrole without further purification. To a mixture of lithium aluminum hydride (78 mg, 2.0 mmol) and

tetrahydrofuran (3 mL) was added a solution of (±)-1-([1,1'-biphenyl]-4-yl)-2-nitrosooctahydrocyclopenta[c]pyrrole in tetrahydrofuran (5 mL) in an ice bath. The resulting reaction mixture was stirred at room temperature. The reaction mixture was treated with ethyl acetate (10 mL) and sequentially with water, 15% sodium hydroxide, water and concentrated. The residue was purified by short flash column chromatography (silica gel; dichloromethane: methanol = 20:1 ~ 10:1) to afford (210 mg, 0.75 mmol) in 66% yield as a white solid (two steps). **^{1}H NMR** (400 MHz, Chloroform-*d*) δ 7.58 – 7.52 (m, 6H), 7.43 – 7.39 (m, 2H), 7.36 – 7.30 (m, 1H), 5.79 (s, 2H), 3.73 (d, *J* = 8.6 Hz, 1H), 3.48 – 3.38 (m, 1H), 2.94 – 2.82 (m, 1H), 2.70 (tt, *J* = 9.3, 4.7 Hz, 1H), 2.55 (dd, *J* = 10.8, 8.3 Hz, 1H), 1.73 – 1.56 (m, 5H), 1.53 – 1.44 (m, 1H). **^{13}C NMR** (101 MHz, Chloroform-*d*) δ 140.8, 140.6, 138.9, 128.8, 128.0, 127.4, 127.4, 127.1, 69.0, 52.6, 51.5, 43.2, 31.7, 30.5, 25.0. **HRMS** (EI) m/z: $(M)^+$ calcd. for $C_{19}H_{22}N_2^+$ 278.1778, found 278.1775.

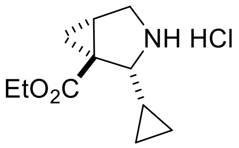

(±)-Ethyl-2-cyclopropyl-3-azabicyclo[3.1.0]hexane-1-carboxylate hydrochloride (46a). Prepared according to the synthetic procedure for the synthesis of **1a**.[1] Purification of the crude mixture by short flash column chromatography (silica gel; hexane: ethyl acetate = 1:1 to dichloromethane: methanol = 20:1) and formation of amine hydrochloride by adding HCl (4.0 M in EA) to afford **46a** (153 mg, 0.56 mmol) in 43% yield as a white solid (the last step). **^{1}H NMR** (400 MHz, Chloroform-*d*) δ 10.59 (s, 1H), 9.62 (s, 1H), 4.29 – 4.13 (m, 2H), 3.61 – 3.48 (m, 2H), 3.41 (s, 1H), 2.18 – 2.14 (m, 1H), 1.77 (d, *J* = 7.0 Hz, 2H), 1.33 – 1.27 (m, 4H), 0.81 – 0.70 (m, 4H). **^{13}C NMR** (101 MHz, Chloroform-*d*) δ 169.9, 65.7, 61.5, 45.0, 33.0, 27.0, 14.1, 13.5, 10.2, 5.3, 3.9. **HRMS** (ESI) m/z: $(M + H)^+$ calcd. for $C_{11}H_{18}NO_2^+$ 196.1332, found 196.1335.

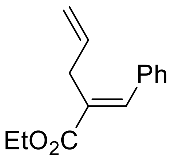

Ethyl (*E*)-2-benzylidenepent-4-enoate (**1b**). Purification of the crude mixture by short flash column chromatography (silica gel; petroleum ether /ethyl acetate = 40:1), a colorless liquid, **SC-I**: 35.0 mg, 81% yield; **SC-II**: 38.5 mg, 89% yield. **^{1}H NMR** (400 MHz, Chloroform-*d*) δ 7.83 (s, 1H), 7.40 (m, 5H), 6.08 – 5.98 (m, 1H), 5.14 (m, 2H), 4.30 (q, *J* = 7.1 Hz, 2H), 3.32 (dt, *J* = 5.7, 1.8 Hz, 2H), 1.37 (t, *J* = 7.1 Hz, 3H). **^{13}C NMR** (101 MHz, Chloroform-*d*) δ 168.0, 140.1, 135.7, 135.5, 130.6, 129.2, 128.5, 128.4, 115.6, 60.8, 31.6, 14.3. **HRMS** (ESI) m/z: $(M+H)^+$ calcd. for $C_{14}H_{17}O_2^+$ 217.1223, found 217.1226.

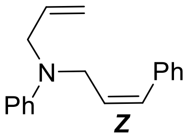

(*Z*)-*N*-Allyl-*N*-(3-phenylallyl)aniline (2b). Purification of the crude mixture by short flash column chromatography (silica gel; petroleum ether /ethyl acetate = 40:1), a colorless liquid, 36.9 mg, 74% yield. **^{1}H NMR** (400 MHz, Chloroform-*d*) δ 7.41 (t, *J* = 7.5 Hz, 2H), 7.30 (t, *J* = 7.9 Hz, 3H), 7.20 (t, *J* = 7.9 Hz, 2H), 6.74 – 6.61 (m, 4H), 5.91– 5.82 (m, 1H), 5.80 – 5.74 (m, 1H), 5.22 – 5.11 (m, 2H), 4.24 (d, *J* = 5.9 Hz, 2H), 3.94 (d, *J* = 4.8 Hz, 2H). **^{13}C NMR** (101 MHz, Chloroform-*d*) δ 148.4, 136.9, 134.1, 131.2, 130.3, 129.1, 128.9, 128.3, 127.1, 116.5, 116.1, 112.7, 53.5, 48.8. **HRMS** (ESI) m/z: $(M+Na)^+$ calcd. for $C_{18}H_{19}NNa^+$ 272.1410, found 272.1412.

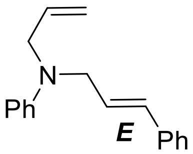

(*E*)-*N*-Allyl-*N*-cinnamylaniline (3b). [16] Purification of the crude mixture by short flash column chromatography (silica gel; petroleum ether /ethyl acetate = 40:1), a colorless liquid, 35.9 mg, 72% yield. **^{1}H NMR** (400 MHz, Chloroform-*d*) δ 7.51 –

7.46 (m, 2H), 7.45 – 7.39 (m, 2H), 7.39 – 7.31 (m, 3H), 6.93 – 6.88 (m, 2H), 6.84 (tt, *J* = 7.2, 1.1 Hz, 1H), 6.65 (dt, *J* = 15.9, 1.7 Hz, 1H), 6.41 – 6.35 (m, 1H), 6.07 – 5.97 (m, 1H), 5.39 – 5.28 (m, 2H), 4.21 (dd, *J* = 5.3, 1.7 Hz, 2H), 4.10 (dt, *J* = 4.9, 1.8 Hz, 2H). **^{13}C NMR** (101 MHz, Chloroform-*d*) δ 148.9, 137.1, 134.2, 131.2, 129.3, 128.6, 127.5, 126.4, 126.1, 116.6, 116.2, 112.6, 52.8, 52.4. **HRMS** (ESI) m/z: $(M + Na)^+$ calcd. for $C_{18}H_{19}NNa^+$ 272.1410, found 272.1411.

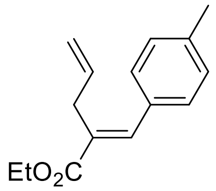

Ethyl (*E*)-2-(4-methylbenzylidene)pent-4-enoate (**4b**). Purification of the crude mixture by short flash column chromatography (silica gel; petroleum ether /ethyl acetate = 40:1), a colorless liquid, **SC-I**: 36.4 mg, 79% yield; **SC-II**: 35.0 mg, 76% yield. **^{1}H NMR** (400 MHz, Chloroform-*d*) δ 7.81 (s, 1H), 7.38 – 7.31 (m, 2H), 7.22 (d, *J* = 7.9 Hz, 2H), 6.07 – 5.99 (m, 1H), 5.19 – 5.09 (m, 2H), 4.30 (q, *J* = 7.1 Hz, 2H), 3.33 (dt, *J* = 5.6, 1.9 Hz, 2H), 2.40 (s, 3H), 1.37 (t, *J* = 7.1 Hz, 3H). **^{13}C NMR** (101 MHz, Chloroform-*d*) δ 168.2, 140.2, 138.7, 135.7, 132.6, 129.6, 129.4, 129.2, 115.6, 60.8, 31.7, 21.3, 14.3. **HRMS** (ESI) m/z: $(M + H)^+$ calcd. for $C_{15}H_{19}O_2{}^+$ 231.1380, found 231.1373.

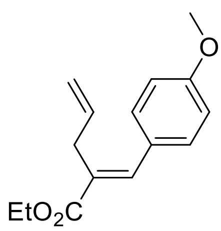

Ethyl (*E*)-2-(4-methoxybenzylidene)pent-4-enoate (**5b**). Purification of the crude mixture by short flash column chromatography (silica gel; petroleum ether /ethyl acetate = 40:1), a colorless liquid, **SC-I**: 37.9 mg, 77% yield; **SC-II**: 40.4 mg, 82% yield. **^{1}H NMR** (400 MHz, Chloroform-*d*) δ 7.79 (s, 1H), 7.45 – 7.37 (m, 2H), 6.97 – 6.91 (m, 2H), 6.08 – 5.99 (m, 1H), 5.19 – 5.10 (m, 2H), 4.29 (q, *J* = 7.1 Hz, 2H), 3.85

(s, 3H), 3.33 (dt, J = 5.5, 1.9 Hz, 2H), 1.36 (t, J = 7.1 Hz, 3H). **^{13}C NMR** (101 MHz, Chloroform-*d*) δ 168.3, 160.0, 140.0, 135.7, 131.1, 128.2, 128.0, 115.5, 113.9, 60.8, 55.3, 31.6, 14.3. **HRMS** (ESI) m/z: $(M+Na)^+$ calcd. for $C_{15}H_{18}O_3Na^+$ 269.1148, found 269.1143.

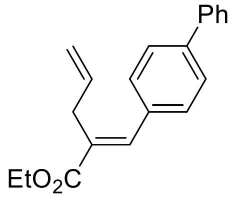

Ethyl (*E*)-2-([1,1'-biphenyl]-4-ylmethylene)pent-4-enoate (6b). Purification of the crude mixture by short flash column chromatography (silica gel; petroleum ether /ethyl acetate = 40:1), a colorless liquid, **SC-I**: 57.9 mg, 99% yield; **SC-II**: 57.8 mg, 99% yield. **^{1}H NMR** (400 MHz, Chloroform-*d*) δ 7.86 (s, 1H), 7.65 (dd, J = 8.2, 2.6 Hz, 4H), 7.50 (dd, J = 22.6, 7.8 Hz, 4H), 7.44 – 7.35 (m, 1H), 6.06 (ddt, J = 16.2, 10.7, 5.5 Hz, 1H), 5.23 – 5.13 (m, 2H), 4.32 (q, J = 7.1 Hz, 2H), 3.38 (d, J = 5.4 Hz, 2H), 1.38 (t, J = 7.0 Hz, 3H). **^{13}C NMR** (101 MHz, Chloroform-*d*) δ 168.0, 141.4, 140.4, 139.7, 135.6, 134.5, 130.5, 129.9, 128.8, 127.6, 127.1, 127.0, 115.7, 60.9, 31.8, 14.3. **HRMS** (ESI) m/z: $(M+Na)^+$ calcd. for $C_{20}H_{20}O_2Na^+$ 315.1356, found 315.1347.

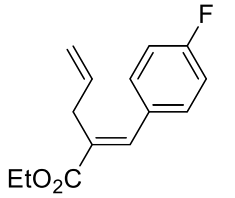

Ethyl (*E*)-2-(4-fluorobenzylidene)pent-4-enoate (**7b**). Purification of the crude mixture by short flash column chromatography (silica gel; petroleum ether /ethyl acetate = 40:1), a colorless liquid, 36.1 mg, 77% yield. **^{1}H NMR** (400 MHz, Chloroform-*d*) δ 7.78 (s, 1H), 7.41 (dd, J = 8.4, 5.4 Hz, 2H), 7.09 (t, J = 8.5 Hz, 2H), 6.06 – 5.97 (m, 1H), 5.24 – 5.05 (m, 2H), 4.29 (q, J = 7.1 Hz, 2H), 3.29 (d, J = 5.4 Hz, 2H), 1.36 (t, J = 7.1 Hz, 3H). **^{13}C NMR** (101 MHz, Chloroform-*d*) δ 167.9, 162.7 (d, J = 249.4 Hz), 139.1, 135.5, 131.6 (d, J = 3.4 Hz), 131.2 (d, J = 8.3 Hz), 130.3, 115.7

(d, *J* = 10.2 Hz), 115.4, 60.9, 31.6, 14.3. **HRMS** (ESI) m/z: $(M + H)^+$ calcd. for $C_{14}H_{16}FO_2^+$ 235.1129, found 236.1122.

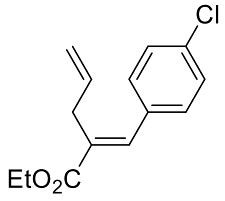

Ethyl (*E*)-2-(4-chlorobenzylidene)pent-4-enoate (**8b**). Purification of the crude mixture by short flash column chromatography (silica gel; petroleum ether /ethyl acetate = 40:1), a colorless liquid, **SC-I**: 36.6 mg, 73% yield; **SC-II**: 39.9 mg, 80% yield. ^{1}**H NMR** (400 MHz, Chloroform-*d*) δ 7.76 (s, 1H), 7.36 (s, 4H), 6.05 – 5.96 (m, 1H), 5.18 – 5.06 (m, 2H), 4.29 (q, *J* = 7.1 Hz, 2H), 3.28 (dt, *J* = 5.6, 1.9 Hz, 2H), 1.36 (t, *J* = 7.1 Hz, 3H). ^{13}**C NMR** (101 MHz, Chloroform-*d*) δ 167.8, 138.9, 135.4, 134.5, 133.9, 131.0, 130.6, 128.7, 115.8, 61.0, 31.6, 14.3. **HRMS** (ESI) m/z: $(M + H)^+$ calcd. for $C_{14}H_{16}ClO_2^+$ 251.0833, found 251.0825.

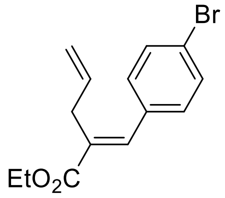

Ethyl (*E*)-2-(4-bromobenzylidene)pent-4-enoate (**9b**). Purification of the crude mixture by short flash column chromatography (silica gel; petroleum ether /ethyl acetate = 40:1), a colorless liquid, **SC-I**: 43.1 mg, 73% yield; **SC-II**: 44.6 mg, 76% yield. ^{1}**H NMR** (400 MHz, Chloroform-*d*) δ 7.73 (s, 1H), 7.52 (d, *J* = 8.2 Hz, 2H), 7.29 (d, *J* = 7.8 Hz, 2H), 6.00 (ddt, *J* = 16.3, 10.6, 5.4 Hz, 1H), 5.12 (t, *J* = 13.4 Hz, 2H), 4.29 (q, *J* = 7.1 Hz, 2H), 3.27 (d, *J* = 5.4 Hz, 2H), 1.36 (t, *J* = 7.1 Hz, 3H). ^{13}**C NMR** (101 MHz, Chloroform-*d*) δ 167.8, 138.9, 135.4, 134.4, 131.7, 130.8, 122.8, 115.8, 61.2, 31.6, 14.3. **HRMS** (ESI) m/z: $(M + Na)^+$ calcd. for $C_{14}H_{15}BrO_2Na^+$ 317.0148, found 317.0142.

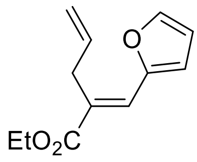

Ethyl (*E*)-2-(furan-2-ylmethylene)pent-4-enoate (**10b**). Purification of the crude mixture by short flash column chromatography (silica gel; petroleum ether /ethyl acetate = 40:1), a colorless liquid, 25.6 mg, 62% yield. **^{1}H NMR** (400 MHz, Chloroform-*d*) δ 7.55 (d, *J* = 1.7 Hz, 1H), 7.51 (s, 1H), 6.65 (d, *J* = 3.4 Hz, 1H), 6.50 (dd, *J* = 3.5, 1.8 Hz, 1H), 5.99 – 5.89 (m, 1H), 5.17 – 5.11 (m, 1H), 5.06 – 5.02 (m, 1H), 4.27 (q, *J* = 7.1 Hz, 2H), 3.51 (dt, *J* = 6.1, 1.7 Hz, 2H), 1.36 (d, *J* = 7.1 Hz, 3H). **^{13}C NMR** (101 MHz, Chloroform-*d*) δ 167.9, 151.4, 144.3, 135.1, 127.0, 126.2, 115.4, 115.2, 112.0, 60.8, 31.9, 14.3. **HRMS** (ESI) m/z: $(M + Na)^+$ calcd. for $C_{12}H_{14}O_3Na^+$ 229.0835, found 229.0829.

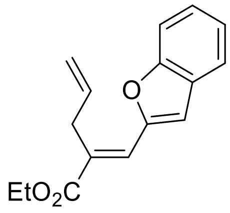

Ethyl (*E*)-2-(benzofuran-2-ylmethylene)pent-4-enoate (11b). Purification of the crude mixture by short flash column chromatography (silica gel; petroleum ether /ethyl acetate = 40:1), a colorless liquid, **SC-I**: 47.2 mg, 92% yield; **SC-II**: 45.2 mg, 88% yield. **^{1}H NMR** (400 MHz, Chloroform-*d*) δ 7.62 (d, *J* = 7.5 Hz, 2H), 7.52 (d, *J* = 8.3 Hz, 1H), 7.37 (t, *J* = 7.8 Hz, 1H), 7.27 (t, *J* = 7.5 Hz, 1H), 6.98 (s, 1H), 6.09 – 5.94 (m, 1H), 5.23 (dt, *J* = 17.2, 1.7 Hz, 1H), 5.09 (dt, *J* = 10.1, 1.6 Hz, 1H), 4.32 (q, *J* = 7.1 Hz, 2H), 3.68 (d, *J* = 6.0 Hz, 2H), 1.38 (t, *J* = 7.1 Hz, 3H). **^{13}C NMR** (101 MHz, Chloroform-*d*) δ 167.6, 155.5, 153.0, 135.1, 130.6, 128.1, 126.3, 125.9, 123.3, 121.5, 115.8, 111.4, 111.4, 61.1, 32.2, 14.3. **HRMS** (ESI) m/z: $(M + Na)^+$ calcd. for $C_{16}H_{16}O_3Na^+$ 279.0992, found 279.0993.

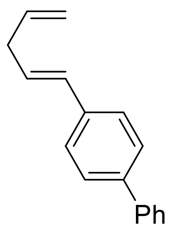

(*E*)-4-(Penta-1,4-dien-1-yl)-1,1'-biphenyl (12b). Purification of the crude mixture by short flash column chromatography (silica gel; petroleum ether /ethyl acetate = 40:1), a colorless liquid, 36.1 mg, 82% yield. **^{1}H NMR** (400 MHz, Chloroform-*d*) δ 7.60 (dd, *J* = 20.6, 7.9 Hz, 4H), 7.52 – 7.41 (m, 4H), 7.36 (t, *J* = 7.4 Hz, 1H), 6.48 (d, *J* = 15.9 Hz, 1H), 6.31 (dt, *J* = 15.7, 6.6 Hz, 1H), 6.00 –5.90 (m, 1H), 5.22 – 5.07 (m, 2H), 3.02 (t, *J* = 6.7 Hz, 2H). **^{13}C NMR** (101 MHz, Chloroform-*d*) δ 140.8, 139.8, 136.7, 136.5, 130.4, 128.8, 128.4, 127.2, 126.9, 126.5, 115.8, 37.1. **HRMS** (ESI) m/z: (M + H)$^{+}$ calcd. for $C_{17}H_{17}{}^{+}$ 221.1325, found 221.1323.

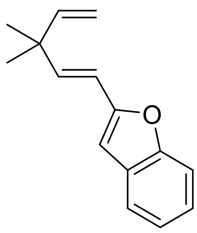

(*E*)-2-(3,3-Dimethylpenta-1,4-dien-1-yl)benzofuran (13b). Purification of the crude mixture by short flash column chromatography (silica gel; petroleum ether /ethyl acetate = 40:1), a colorless liquid, 36.1 mg, 85% yield. **^{1}H NMR** (400 MHz, Chloroform-*d*) δ 7.54 – 7.49 (m, 1H), 7.46 – 7.43 (m, 1H), 7.28 – 7.16 (m, 2H), 6.56 – 6.49 (m, 2H), 6.31 (d, *J* = 16.1 Hz, 1H), 5.94 (dd, *J* = 17.5, 10.6 Hz, 1H), 5.13 – 5.02 (m, 2H), 1.27 (s, 6H). **^{13}C NMR** (101 MHz, Chloroform-*d*) δ 155.2, 154.6, 146.2, 141.2, 129.1, 124.1, 122.7, 120.6, 115.5, 111.5, 110.8, 103.3, 39.6, 26.8. **HRMS** (ESI) m/z: (M + H)$^{+}$ calcd. for $C_{15}H_{17}O^{+}$ 213.1274, found 213.1265.

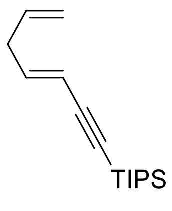

(*E*)-Hepta-3,6-dien-1-yn-1-yltriisopropylsilane (14b). Purification of the crude mixture by short flash column chromatography (silica gel; petroleum ether /ethyl acetate = 40:1), a colorless liquid, 49.2 mg, 99% yield. **^{1}H NMR** (400 MHz, Chloroform-*d*) δ 6.23 (dt, *J* = 15.9, 6.6 Hz, 1H), 5.87 – 5.77 (m, 1H), 5.59 (dt, *J* = 15.9, 1.8 Hz, 1H), 5.14 – 5.05 (m, 2H), 2.88 (tq, *J* = 6.6, 1.6 Hz, 2H), 1.10 (d, *J* = 2.2 Hz, 21H). **^{13}C NMR** (101 MHz, Chloroform-*d*) δ 142.6, 135.0, 116.5, 111.0, 105.7, 89.5, 36.92, 18.6, 11.3. **HRMS** (EI) m/z: $(M)^+$ calcd. for $C_{16}H_{28}Si^+$ 248.1955, found 248.1964.

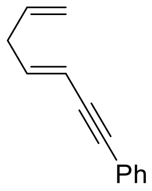

(*E*)-Hepta-3,6-dien-1-yn-1-ylbenzene (15b). [17] Purification of the crude mixture by short flash column chromatography (silica gel; petroleum ether /ethyl acetate = 40:1), a colorless liquid, **SC-I**: 27.6 mg, 82% yield; **SC-II**: 25.1 mg, 75% yield. **^{1}H NMR** (400 MHz, Chloroform-*d*) δ 7.46 (dd, *J* = 6.8, 2.8 Hz, 2H), 7.33 (dd, *J* = 5.3, 2.0 Hz, 3H), 6.29 (dt, *J* = 15.8, 6.7 Hz, 1H), 5.82 (ddt, *J* = 16.8, 10.1, 6.4 Hz, 1H), 5.72 (d, *J* = 15.9 Hz, 1H), 5.12 – 5.06 (m, 2H), 2.94 – 2.86 (m, 2H). **^{13}C NMR** (101 MHz, Chloroform-*d*) δ 141.9, 135.1, 131.5, 128.3, 128.0, 123.5, 116.5, 110.7, 88.5, 88.1, 37.0. **HRMS** (ESI) m/z: $(M + H)^+$ calcd. for $C_{13}H_{13}^+$ 169.1012, found 169.1008.

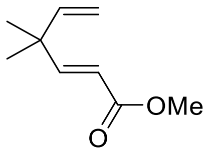

Methyl (*E*)-4,4-dimethylhexa-2,5-dienoate (16b). Purification of the crude mixture by short flash column chromatography (silica gel; petroleum ether /ethyl acetate = 40:1), a colorless liquid, 23.4 mg, 76% yield. **^{1}H NMR** (400 MHz, Chloroform-*d*) δ 6.97 (d, *J* = 15.9 Hz, 1H), 5.87 – 5.75 (m, 2H), 5.07 – 4.98 (m, 2H), 3.75 (s, 3H), 1.19 (s, 6H). **^{13}C NMR** (101 MHz, Chloroform-*d*) δ 167.4, 156.4, 144.7, 117.7, 112.4, 51.4,

39.7, 26.2. **HRMS** (ESI) m/z: $(M + Na)^+$ calcd. for $C_9H_{17}O_2Na^+$ 177.0886, found 177.0888.

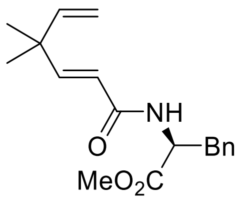

Methyl (*E*)-(4,4-dimethylhexa-2,5-dienoyl)-*L*-phenylalaninate (17b). Purification of the crude mixture by short flash column chromatography (silica gel; petroleum ether /ethyl acetate = 40:1), a colorless liquid, 51.7 mg, 82% yield. **^{1}H NMR** (400 MHz, Chloroform-*d*) δ 7.34 – 7.22 (m, 3H), 7.15 – 7.10 (m, 2H), 6.85 (d, *J* = 15.5 Hz, 1H), 6.02 (d, *J* = 7.8 Hz, 1H), 5.87 – 5.77 (m, 1H), 5.71 (d, *J* = 15.5 Hz, 1H), 5.03 (s, 1H), 5.01 – 4.95 (m, 2H), 3.74 (s, 3H), 3.17 (dd, *J* = 5.8, 3.3 Hz, 2H), 1.17 (s, 6H). **^{13}C NMR** (101 MHz, Chloroform-*d*) δ 172.1, 165.6, 152.8, 145.0, 135.9, 129.3, 128.5, 127.1, 119.7, 112.2, 53.2, 52.3, 39.4, 38.0, 26.4, 26.3. **HRMS** (ESI) m/z: $(M + H)^+$ calcd. for $C_{18}H_{24}O_3N^+$ 302.1751, found 302.1748.

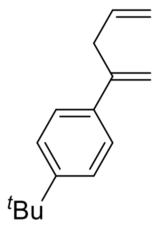

1-(Tert-butyl)-4-(penta-1,4-dien-2-yl)benzene (18b). Purification of the crude mixture by short flash column chromatography (silica gel; petroleum ether /ethyl acetate = 40:1), a colorless liquid, **SC-I**: 32.5 mg, 81% yield; **SC-II**: 32.1 mg, 80% yield. **^{1}H NMR** (400 MHz, Chloroform-*d*) δ 7.47 – 7.35 (m, 4H), 5.97 (ddt, *J* = 17.4, 10.2, 6.3 Hz, 1H), 5.43 (s, 1H), 5.22 – 5.12 (m, 1H), 5.19 – 5.10 (m, 3H), 3.28 (d, *J* = 6.6 Hz, 2H), 1.36 (s, 9H). **^{13}C NMR** (101 MHz, Chloroform-*d*) δ 150.4, 145.9, 137.9, 136.4, 125.6, 125.2, 116.3, 112.4, 39.5, 34.5, 31.3. **H HRMS** (ESI) m/z: $(M + Na)^+$ calcd. for $C_{15}H_{20}Na^+$ 223.1457, found 223.1456.

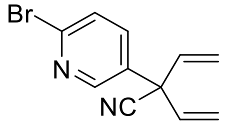

2-(6-Bromopyridin-3-yl)-2-vinylbut-3-enenitrile (19b). Purification of the crude mixture by short flash column chromatography (silica gel; petroleum ether /ethyl acetate = 10:1), a colorless liquid, 26.9 mg, 54% yield. **^{1}H NMR** (400 MHz, Chloroform-*d*) δ 8.70 (d, *J* = 2.2 Hz, 1H), 7.89 (dd, *J* = 8.4, 2.4 Hz, 1H), 7.49 (d, *J* = 8.4 Hz, 1H), 6.17 (dd, *J* = 17.1, 10.1 Hz, 2H), 5.64 (d, *J* = 17.1 Hz, 2H), 5.44 (d, *J* = 10.2 Hz, 2H). **^{13}C NMR** (101 MHz, Chloroform-*d*) δ 155.7, 150.9, 140.0, 135.0, 122.6, 120.3, 118.2, 117.5, 54.9. **HRMS** (ESI) m/z: $(M + Na)^+$ calcd. for $C_{11}H_9BrN_2Na^+$ 270.9841, found 270.9840.

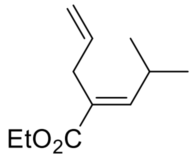

Ethyl (*E*)-2-allyl-4-methylpent-2-enoate (20b). Purification of the crude mixture by short flash column chromatography (silica gel; petroleum ether /ethyl acetate = 40:1), a colorless liquid, **SC-I**: 28.4 mg, 78% yield; **SC-II**: 30.3 mg, 83% yield. **^{1}H NMR** (400 MHz, Chloroform-*d*) δ 6.66 (d, *J* = 10.1 Hz, 1H), 5.90 – 5.80 (m, 1H), 5.07 – 4.96 (m, 2H), 4.20 (q, *J* = 7.1 Hz, 2H), 3.08 (dt, *J* = 4.0, 2.0 Hz, 2H), 2.72 – 2.62 (m, 1H), 1.31 (t, *J* = 7.1 Hz, 3H), 1.04 (d, *J* = 6.6 Hz, 6H). **^{13}C NMR** (101 MHz, Chloroform-*d*) δ 167.9, 150.1, 136.1, 127.6, 114.9, 60.5, 30.9, 27.9, 22.2, 14.3. **HRMS** (ESI) m/z: $(M + Na)^+$ calcd. for $C_{11}H_{18}O_2Na^+$ 205.1199, found 205.1198.

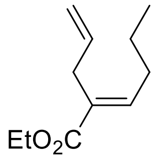

Ethyl (*E*)-2-allylhex-2-enoate (21b). Purification of the crude mixture by short flash column chromatography (silica gel; petroleum ether /ethyl acetate = 40:1), a colorless liquid, 29.9 mg, 82% yield. **^{1}H NMR** (400 MHz, Chloroform-*d*) δ 6.86 (t, *J* = 7.5 Hz,

1H), 5.88 – 5.78 (m, 1H), 5.07 – 4.96 (m, 2H), 4.21 (q, *J* = 7.1 Hz, 2H), 3.09 (dt, *J* = 6.1, 1.7 Hz, 2H), 2.18 (q, *J* = 7.5 Hz, 2H), 1.50 (m, 2H), 1.31 (t, *J* = 7.1 Hz, 3H), 0.96 (t, *J* = 7.4 Hz, 3H). **^{13}C NMR** (101 MHz, Chloroform-*d*) δ 167.6, 143.7, 135.6, 130.0, 115.0, 60.4, 30.8, 30.6, 22.0, 14.3, 14.0. **HRMS** m/z (ESI) calcd. for $C_{11}H_{18}O_2Na^+$ $(M + Na)^+$ 205.1199, found 205.1197.

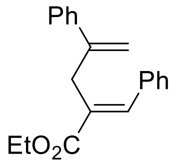

Ethyl (*E*)-2-benzylidene-4-phenylpent-4-enoate (22b). Purification of the crude mixture by short flash column chromatography (silica gel; petroleum ether /ethyl acetate = 40:1), a colorless liquid, 44.4 mg, 76% yield. **^{1}H NMR** (400 MHz, Chloroform-*d*) δ 7.96 (s, 1H), 7.53 (d, *J* = 7.3 Hz, 2H), 7.46 (d, *J* = 6.9 Hz, 2H), 7.37 (dt, *J* = 14.7, 6.8 Hz, 6H), 5.47 (s, 1H), 5.09 (s, 1H), 4.31 (q, *J* = 7.1 Hz, 2H), 3.73 (s, 2H), 1.35 (t, *J* = 7.2 Hz, 3H). **^{13}C NMR** (101 MHz, Chloroform-*d*) δ 168.1, 145.8, 141.9, 140.9, 135.4, 130.4, 129.2, 128.7, 128.5, 128.3, 127.6, 126.0, 112.3, 60.9, 33.3, 14.3. **HRMS** (ESI) m/z: $(M + Na)^+$ calcd. for $C_{20}H_{20}O_2Na^+$ 315.1356, found 315.1352.

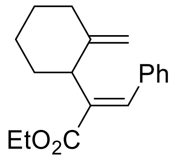

Ethyl (*E*)-2-(2-methylenecyclohexyl)-3-phenylacrylate (23b). Purification of the crude mixture by short flash column chromatography (silica gel; petroleum ether /ethyl acetate = 40:1), a colorless liquid, 36.8 mg, 68% yield. **^{1}H NMR** (400 MHz, Chloroform-*d*) δ 7.77 (s, 1H), 7.42 – 7.30 (m, 5H), 4.86 (q, *J* = 1.9 Hz, 1H), 4.65 (q, *J* = 1.8 Hz, 1H), 4.36 – 4.23 (m, 2H), 3.42 (ddd, *J* = 13.4, 3.9, 1.8 Hz, 1H), 2.50 (ddt, *J* = 13.7, 4.1, 2.2 Hz, 1H), 2.23 – 2.03 (m, 2H), 1.86 – 1.75 (m, 2H), 1.70 – 1.61 (m, 1H), 1.52 – 1.39 (m, 1H), 1.35 (d, *J* = 7.1 Hz, 3H), 1.32 – 1.25 (m, 1H). **^{13}C NMR**

(101 MHz, Chloroform-*d*) δ 167.8, 149.2, 140.0, 135.8, 135.3, 128.9, 128.3, 128.2, 107.9, 60.4, 44.1, 35.8, 31.4, 27.2, 26.4, 14.3. **HRMS** (ESI) m/z: $(M + Na)^+$ calcd. for $C_{18}H_{22}O_2Na^+$ 293.1512, found 293.1509.

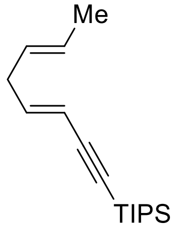

Triisopropyl((3*E*,6*E*)-octa-3,6-dien-1-yn-1-yl)silane (24b). [18] Purification of the crude mixture by short flash column chromatography (silica gel; petroleum ether /ethyl acetate = 40:1), a colorless liquid, 43.7 mg, 83% yield. **^{1}H NMR** (400 MHz, Chloroform-*d*) δ 6.22 (dt, *J* = 15.9, 6.5 Hz, 1H), 5.56 (d, *J* = 16.3 Hz,1H), 5.53 – 5.37 (m, 2H), 2.80 (t, *J* = 6.5 Hz, 2H), 1.69 (d, *J* = 6.1 Hz, 3H), 1.09 (s, 21H). **^{13}C NMR** (101 MHz, Chloroform-*d*) δ 143.9, 127.4, 127.2, 110.4, 105.9, 89.2, 35.8, 18.7, 17.9, 11.3. **HRMS** (EI) m/z: $(M)^+$ calcd. for $C_{17}H_{30}Si^+$ 262.2111, found 262.2105.

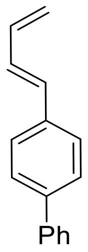

(*E*)-4-(Buta-1,3-dien-1-yl)-1,1'-biphenyl (**25b**). Purification of the crude mixture by short flash column chromatography (silica gel; petroleum ether /ethyl acetate = 40:1), a colorless liquid, 38.0 mg, 92% yield. **^{1}H NMR** (400 MHz, Chloroform-*d*) δ 7.65 – 7.57 (m, 4H), 7.53 – 7.44 (m, 4H), 7.40 – 7.33 (m, 1H), 6.86 (dd, *J* = 15.6, 10.5 Hz, 1H), 6.67 – 6.50 (m, 2H), 5.38 (dt, *J* = 16.8, 1.0 Hz, 1H), 5.25 – 5.20 (m, 1H). **^{13}C NMR** (101 MHz, Chloroform-*d*) δ 140.7, 140.4, 137.2, 136.2, 132.4, 129.7, 128.8, 127.4, 127.3, 126.9, 126.9, 117.7. **HRMS** (ESI) m/z: $(M + H)^+$ calcd. for $C_{16}H_{15}^+$ 207.1168, found 207.1165.

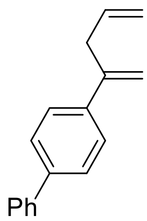

4-(Penta-1,4-dien-2-yl)-1,1'-biphenyl (26b). Purification of the crude mixture by short flash column chromatography (silica gel; petroleum ether /ethyl acetate = 40:1), a colorless liquid, **SC-I**: 40.1 mg, 91% yield; **SC-II**: 43.7 mg, 99% yield. **^{1}H NMR** (400 MHz, Chloroform-*d*) δ 7.67 – 7.53 (m, 6H), 7.51 – 7.45 (m, 2H), 7.41 – 7.35 (m, 1H), 6.03-5.93 (m, 1H), 5.50 (d, *J* = 1.2 Hz, 1H), 5.23 – 5.09 (m, 3H), 3.33 (dd, *J* = 6.6, 1.6 Hz, 2H). **^{13}C NMR** (101 MHz, Chloroform-*d*) δ 145.8, 140.7, 140.3, 139.8, 136.2, 128.8, 127.3, 127.0, 127.0, 126.4, 116.6, 113.2, 39.5. **HRMS** (ESI) m/z: $(M + H)^+$ calcd. for $C_{17}H_{17}^+$ 221.1325, found 221.1325.

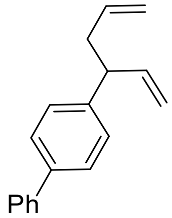

4-(Hexa-1,5-dien-3-yl)-1,1'-biphenyl (**27b**). Purification of the crude mixture by short flash column chromatography (silica gel; petroleum ether /ethyl acetate = 40:1), a colorless liquid, 35.2 mg, 75% yield. **^{1}H NMR** (400 MHz, Chloroform-*d*) δ 7.62 (dd, *J* = 17.7, 7.7 Hz, 4H), 7.48 (t, *J* = 7.4 Hz, 2H), 7.38 (t, *J* = 7.2 Hz, 3H), 7.32 (d, *J* = 7.8 Hz, 2H), 6.06 (ddd, *J* = 17.5, 10.7, 7.8 Hz, 1H), 5.89 – 5.74 (m, 1H), 5.17 – 5.01 (m, 4H), 3.46 (q, *J* = 7.5 Hz, 1H), 2.59 (t, *J* = 7.3 Hz, 2H). **^{13}C NMR** (101 MHz, Chloroform-*d*) δ 142.9, 141.5, 141.0, 139.2, 136.6, 128.8, 128.1, 127.2, 127.1, 127.0, 116.3, 114.6, 49.4, 39.8. **HRMS** (ESI) m/z: $(M + Na)^+$ calcd. for $C_{18}H_{18}Na^+$ 257.1301, found 257.1302.

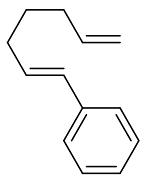

(*E*)-Hepta-1,6-dien-1-ylbenzene (28b). Purification of the crude mixture by short flash column chromatography (silica gel; petroleum ether /ethyl acetate = 40:1), a colorless liquid, **SC-I**: 26.5 mg, 81% yield; **SC-II**: 29.6 mg, 86% yield. **^{1}H NMR** (400 MHz, Chloroform-*d*) δ 7.36 (d, *J* = 7.4 Hz, 2H), 7.31 (t, *J* = 7.6 Hz, 2H), 7.21 (t, *J* = 7.2 Hz, 1H), 6.40 (d, *J* = 15.8 Hz, 1H), 6.27 – 6.20 (m, 1H), 5.92 – 5.78 (m, 1H), 5.07 – 4.98 (m, 2H), 2.25 (q, *J* = 7.1 Hz, 2H), 2.13 (q, *J* = 7.1 Hz, 2H), 1.63 – 1.54 (m, 2H). **^{13}C NMR (101 MHz, $CDCl_3$)** δ 138.7, 137.9, 130.7, 130.1, 128.5, 126.8, 125.9, 114.7, 33.3, 32.5, 28.6. **HRMS** (ESI) m/z: $(M + Na)^+$ calcd. for $C_{13}H_{16}Na^+$ 195.1144, found 195.1144.

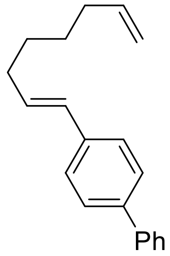

(*E*)-4-(Octa-1,7-dien-1-yl)-1,1'-biphenyl (29b). Purification of the crude mixture by short flash column chromatography (silica gel; petroleum ether /ethyl acetate = 40:1), a colorless liquid, **SC-I**: 16.3 mg, 31% yield; **SC-II**: 18.4 mg, 35% yield. **^{1}H NMR** (400 MHz, Chloroform-*d*) δ 7.67 – 7.54 (m, 4H), 7.50 – 7.42 (m, 4H), 7.36 (t, *J* = 7.3 Hz, 1H), 6.45 (d, *J* = 15.8 Hz, 1H), 6.33 – 6.26 (m, 1H), 5.91 – 5.79 (m, 1H), 5.09 – 4.95 (m, 2H), 2.27 (q, *J* = 6.8 Hz, 2H), 2.12 (q, *J* = 6.8 Hz, 2H), 1.56 – 1.44 (m, 4H). **^{13}C NMR** (101 MHz, Chloroform-*d*) δ 140.9, 139.6, 138.9, 137.0, 131.2, 129.4, 128.7, 127.2, 127.1, 126.9, 126.3, 114.4, 33.7, 33.0, 28.9, 28.5. **HRMS** (ESI) m/z: $(M + H)^+$ calcd. for $C_{20}H_{23}^+$ 263.1794, found 263.1792.

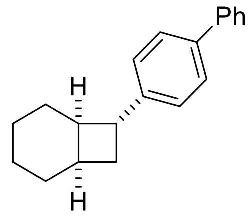

(1*R*,6*R*,7*R*)-7-([1,1'-Biphenyl]-4-yl)bicyclo[4.2.0]octane (29c). Purification of the crude mixture by short flash column chromatography (silica gel; petroleum ether /ethyl acetate = 40:1), a colorless liquid, 32.5 mg, 62% yield. **^{1}H NMR** (400 MHz, Chloroform-*d*) δ 7.68 – 7.61 (m, 2H), 7.61 – 7.56 (m, 2H), 7.48 (t, *J* = 7.7 Hz, 2H), 7.41 – 7.33 (m, 3H), 3.60 (q, *J* = 8.9 Hz, 1H), 2.60 – 2.51 (m, 1H), 2.35 – 2.17 (m, 2H), 2.10 – 2.01 (m, 1H), 1.96 (dd, *J* = 9.9, 7.8 Hz, 1H), 1.72 (dt, *J* = 12.2, 7.8 Hz, 3H), 1.54 (dtt, *J* = 13.6, 10.3, 3.3 Hz, 3H), 1.16 (dtd, *J* = 13.0, 10.0, 3.0 Hz, 1H). **^{13}C NMR** (101 MHz, Chloroform-*d*) δ 144.6, 141.2, 138.7, 128.7, 127.2, 127.0, 127.0, 41.2, 40.1, 33.2, 29.6, 29.5, 26.2, 23.9, 22.4. **HRMS** (ESI) m/z: $(M + H)^+$ calcd. for $C_{20}H_{23}^+$ 263.1794, found 263.1794.

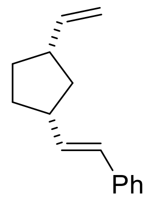

((*E*)-2-((1*R*,3*S*)-3-Vinylcyclopentyl)vinyl)benzene (30b). Purification of the crude mixture by short flash column chromatography (silica gel; petroleum ether /ethyl acetate = 40:1), a colorless liquid, 36.9 mg, 93% yield. **^{1}H NMR** (400 MHz, Chloroform-*d*) δ 7.33 (d, *J* = 7.0 Hz, 2H), 7.27 (t, *J* = 7.7 Hz, 2H), 7.21 – 7.12 (m, 1H), 6.36 (d, *J* = 15.8 Hz, 1H), 6.22 – 6.16 (m, 1H), 5.87 – 5.78 (m, 1H), 5.00 (d, *J* = 17.1 Hz, 1H), 4.90 (d, *J* = 10.2 Hz, 1H), 2.74 – 2.63 (m, 1H), 2.61 – 2.53 (m, 1H), 2.05 – 1.98 (m, 1H), 1.94 – 1.83 (m, 2H), 1.59 – 1.43 (m, 2H), 1.30 – 1.22 (m, 1H). **^{13}C NMR** (101 MHz, Chloroform-*d*) δ 143.2, 137.8, 135.3, 128.5, 128.1, 126.8, 126.0, 112.5, 44.3, 43.8, 40.6, 32.1, 31.8. **HRMS** (EI) m/z: $(M)^+$ calcd. for $C_{15}H_{18}^+$ 198.1403, found 198.1411.

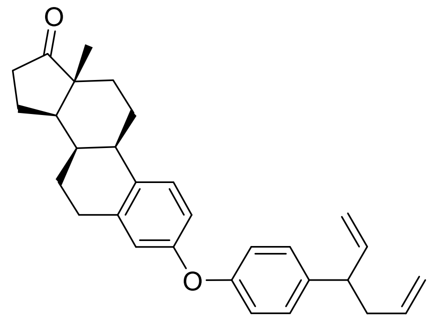

(8*R*,9*R*,13*R*,14*R*)-3-(4-(Hexa-1,5-dien-3-yl)phenoxy)-13-methyl-6,7,8,9,11,12,13,14,15,16-decahydro-17*H*-cyclopenta[a]phenanthren-17-one (31b). Purification of the crude mixture by short flash column chromatography (silica gel; petroleum ether /ethyl acetate = 40:1), a colorless liquid, 78.5 mg, 92% yield. **^{1}H NMR** (400 MHz, Chloroform-*d*) δ 7.30 – 7.23 (m, 1H), 7.20 – 7.13 (m, 2H), 7.01 – 6.93 (m, 2H), 6.85 – 6.75 (m, 2H), 6.04 – 5.95 (m, 1H), 5.76 (ddt, *J* = 17.1, 10.1, 6.9 Hz, 1H), 5.13 – 5.06 (m, 2H), 5.06 – 4.98 (m, 2H), 3.37 (q, *J* = 7.4 Hz, 1H), 2.95 – 2.84 (m, 2H), 2.59 – 2.39 (m, 4H), 2.34 – 2.38 (m, 1H), 2.24 – 1.95 (m, 4H), 1.70 – 1.46 (m, 6H), 0.95 (s, 3H). **^{13}C NMR** (101 MHz, Chloroform-*d*) δ 155.7, 155.3, 141.6, 138.4, 138.2, 136.6, 134.6, 128.8, 126.5, 118.8, 118.7, 116.2, 116.2, 114.4, 50.5, 48.9, 48.0, 44.1, 39.9, 38.3, 35.9, 31.6, 29.5, 26.5, 25.9, 21.6, 13.9. **HRMS** (ESI) m/z: $(M + Na)^+$ calcd. for $C_{30}H_{34}O_2Na^+$ 449.2451, found 449.2450.

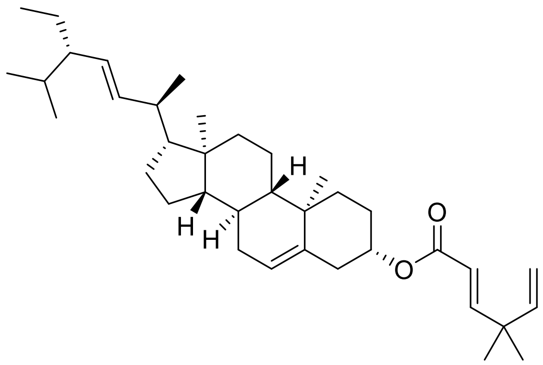

(3*S*,8*S*,9*S*,10*R*,13*R*,14*S*,17*R*)-17-((2*R*,5*S*,*E*)-5-Ethyl-6-methylhept-3-en-2-yl)-10,13-dimethyl-2,3,4,7,8,9,10,11,12,13,14,15,16,17-tetradecahydro-1*H*-cyclopenta-[a]phenanthren-3-yl (*E*)-4,4-dimethylhexa-2,5-dienoate (32b). Purification of the crude mixture by short flash column chromatography (silica gel; petroleum ether /ethyl acetate = 40:1), a colorless liquid, 79.4 mg, 74% yield. **^{1}H NMR** (400 MHz,

Chloroform-*d*) δ 6.92 (d, *J* = 15.9 Hz, 1H), 5.88 – 5.69 (m, 2H), 5.38 (d, *J* = 5.0 Hz, 1H), 5.16 (dd, *J* = 15.1, 8.5 Hz, 1H), 5.09 – 4.94 (m, 3H), 4.74 – 4.61 (m, 1H), 2.35 (d, *J* = 7.4 Hz, 2H), 2.11 – 1.81 (m, 5H), 1.70 – 1.45 (m, 10H), 1.32 – 1.09 (m, 11H), 1.02 (d, *J* = 6.5 Hz, 9H), 0.90 – 0.74 (m, 9H), 0.70 (s, 3H). **^{13}C NMR** (101 MHz, Chloroform-*d*) δ 166.6, 156.1, 145.0, 139.9, 138.5, 129.5, 122.8, 118.7, 112.4, 74.0, 57.0, 56.1, 51.4, 50.3, 42.4, 40.6, 39.8, 38.4, 37.2, 36.8, 32.5, 29.1, 28.0, 26.3, 25.6, 24.5, 21.4, 21.2, 21.2, 19.5, 19.2, 12.4, 12.2, 1.2. **HRMS** (ESI) m/z: $(M + Na)^+$ calcd. for $C_{37}H_{58}O_2Na^+$ 557.4329, found 557.4338.

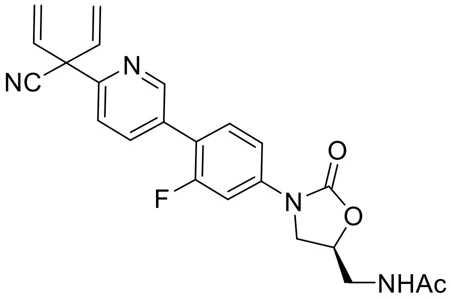

(*S*)-*N*-((3-(4-(6-(3-Cyanopenta-1,4-dien-3-yl)pyridin-3-yl)-3-fluorophenyl)-2-oxooxazolidin-5-yl)methyl)acetamide (33b). Purification of the crude mixture by short flash column chromatography (silica gel; petroleum ether /ethyl acetate = 40:1), a colorless liquid, 45.4 mg, 54% yield. **^{1}H NMR** (400 MHz, Chloroform-*d*) δ 8.77 (s, 1H), 7.97 – 7.85 (m, 1H), 7.70 – 7.51 (m, 2H), 7.44 (t, *J* = 8.5 Hz, 1H), 7.32 (dd, *J* = 8.6, 2.3 Hz, 1H), 6.27 – 6.19 (m, 3H), 5.66 (d, *J* = 17.1 Hz, 2H), 5.45 (d, *J* = 10.1 Hz, 2H), 4.86 – 4.80 (m, *J* = 8.8, 5.9, 3.6 Hz, 1H), 4.10 (t, *J* = 9.0 Hz, 1H), 3.85 (dd, *J* = 9.2, 6.6 Hz, 1H), 3.76 – 3.64 (m, 2H), 2.04 (s, 3H). **^{13}C NMR** (100 MHz, CDCl3) δ 171.3, 161.2, 156.0, 154.2, 149.3 (d, *J* = 3.6 Hz), 139.6 (d, *J* = 11.0 Hz), 137.5 (d, *J* = 3.6 Hz), 135.2, 130.6 (d, *J* = 4.6 Hz), 130.5 (d, *J* = 1.8 Hz), 120.9, 120.3 (d, *J* = 13.8 Hz), 118.5, 117.3, 113.9 (d, *J* = 3.3 Hz), 106.5 (d, *J* = 28.5 Hz), 72.1, 55.2, 47.4, 41.9, 23.1, **HRMS** (ESI) m/z: $(M + H)^+$ calcd. for $C_{23}H_{22}FN_4O_3^+$ 421.1670, found 421.1664.

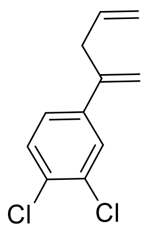

1,2-Dichloro-4-(penta-1,4-dien-2-yl)benzene (34b). Purification of the crude mixture by short flash column chromatography (silica gel; petroleum ether /ethyl acetate = 40:1), a colorless liquid, **SC-I**: 33.2 mg, 78% yield; **SC-II**: 42.3 mg, 95% yield. **^{1}H NMR** (400 MHz, Chloroform-*d*) δ 7.53 (d, *J* = 2.1 Hz, 1H), 7.40 (d, *J* = 8.4 Hz, 1H), 7.28 (dd, *J* = 8.3, 2.2 Hz, 1H), 5.95 – 5.81 (m, 1H), 5.46 – 5.41 (m, 1H), 5.24 – 5.06 (m, 3H), 3.24 – 3.21 (m, 2H). **^{13}C NMR** (101 MHz, Chloroform-*d*) δ 144.2, 141.0, 135.4, 132.4, 131.3, 130.1, 128.0, 125.4, 117.0, 114.8, 39.3. **HRMS** (ESI) m/z: $(M + H)^+$ calcd. for $C_{11}H_{11}Cl_2^+$ 213.0232, found 213.0233.

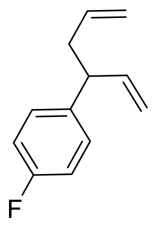

1-Fluoro-4-(hexa-1,5-dien-3-yl)benzene (**35b**). Purification of the crude mixture by short flash column chromatography (silica gel; petroleum ether /ethyl acetate = 40:1), a colorless liquid, 28.2 mg, 80% yield. **^{1}H NMR** (400 MHz, CDCl3) δ 7.31 – 7.23 (m, 2H), 7.16 – 7.06 (m, 2H), 6.02 – 5.94 (m, 1H), 5.76 – 5.70 (m, 1H), 5.11 – 4.94 (m, 4H), 3.33 (q, *J* = 7.2 Hz, 1H), 2.56 – 2.36 (m, 2H); **^{13}C NMR** (100 MHz, CDCl3) δ 161.4 (d, *J* = 244.0 Hz), 141.0, 139.3 (d, *J* = 3.2 Hz), 136.3, 129.1 (d, *J* = 7.8 Hz), 116.3, 115.1 (d, *J* = 21.2 Hz), 114.5, 48.8, 39.8, **HRMS** (EI) m/z: $(M)^+$ calcd. for $C_{12}H_{13}F^+$ 176.0996, found 176.1004.

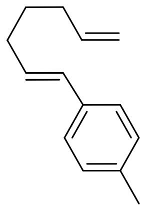

(*E*)-1-(Hepta-1,6-dien-1-yl)-4-methylbenzene (**36b**). Purification of the crude mixture by short flash column chromatography (silica gel; petroleum ether /ethyl acetate = 40:1), a colorless liquid, 32.4 mg, 87% yield. **^{1}H NMR** (400 MHz, Chloroform-*d*) δ 7.33 – 7.27 (m, 2H), 7.16 (d, *J* = 7.9 Hz, 2H), 6.41 (d, *J* = 15.8 Hz, 1H), 6.25 – 6.18 (m, 1H), 5.94-5.84 (m, 1H), 5.13 – 4.99 (m, 2H), 2.38 (s, 3H), 2.30 – 2.24 (m, 2H), 2.20 – 2.14 (m, 2H), 1.66 – 1.59 (m, 2H). **^{13}C NMR** (101 MHz, Chloroform-*d*) δ 138.7, 136.5, 135.1, 129.9, 129.7, 129.2, 125.8, 114.6, 33.3, 32.5, 28.7, 21.2. **HRMS** (ESI) m/z: $(M + H)^{+}$ calcd. for $C_{14}H_{19}^{+}$ 187.1481, found 187.1481.

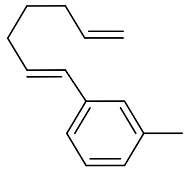

(*E*)-1-(Hepta-1,6-dien-1-yl)-3-methylbenzene (**37b**). Purification of the crude mixture by short flash column chromatography (silica gel; petroleum ether /ethyl acetate = 40:1), a colorless liquid, 33.2 mg, 89% yield. **^{1}H NMR** (400 MHz, Chloroform-*d*) δ 7.27 – 7.18 (m, 3H), 7.07 (d, *J* = 7.1 Hz, 1H), 6.41 (d, *J* = 15.8 Hz, 1H), 6.29 – 6.22 (m, 1H), 5.94 – 5.84 (m, 1H), 5.13 – 5.00 (m, 2H), 2.39 (s, 3H), 2.30 – 2.25 (m, 2H), 2.19 – 2.14 (m, 2H), 1.66 – 1.59 (m, 2H). **^{13}C NMR** (101 MHz, Chloroform-*d*) δ 138.7, 138.0, 137.8, 130.5, 130.2, 128.4, 127.6, 126.7, 123.1, 114.7, 33.3, 32.5, 28.6, 21.4. **HRMS** (ESI) m/z: $(M + H)^{+}$ calcd. for $C_{14}H_{19}^{+}$ 187.1481, found 187.1483.

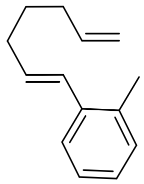

(*E*)-1-(Hepta-1,6-dien-1-yl)-2-methylbenzene (**38b**). Purification of the crude mixture by short flash column chromatography (silica gel; petroleum ether /ethyl acetate = 40:1), a colorless liquid, 25.3 mg, 68% yield. **^{1}H NMR** (400 MHz, Chloroform-*d*) δ 7.47 – 7.41 (m, 1H), 7.21 – 7.12 (m, 3H), 6.61 (dt, *J* = 15.6, 1.5 Hz,

1H), 6.07 (dt, J = 15.6, 7.0 Hz, 1H), 5.87 (m, 1H), 5.11 – 4.98 (m, 2H), 2.36 (s, 3H), 2.33 – 2.24 (m, 2H), 2.21 – 2.11 (m, 2H), 1.67 – 1.57 (m, 2H). **^{13}C NMR** (101 MHz, Chloroform-*d*) δ 138.7, 137.0, 134.9, 132.0, 130.1, 128.0, 126.8, 126.0, 125.5, 114.6, 33.2, 32.7, 28.7, 19.8. **HRMS** (EI) m/z: $(M)^+$ calcd. for $C_{14}H_{18}^+$ 186.1403, found 186.1411.

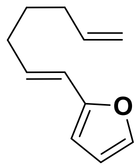

(*E*)-2-(Hepta-1,6-dien-1-yl)furan (**39b**). Purification of the crude mixture by short flash column chromatography (silica gel; petroleum ether /ethyl acetate = 40:1), a colorless liquid, 21.7 mg, 67% yield. **^{1}H NMR** (400 MHz, Chloroform-*d*) δ 7.33 (d, J = 1.7 Hz, 1H), 6.37 (dd, J = 3.3, 1.8 Hz, 1H), 6.28 – 6.17 (m, 2H), 6.15 (d, J = 3.2 Hz, 1H), 5.90 – 5.80 (m, 1H), 5.08 – 4.97 (m, 2H), 2.25 – 2.20 (m, 2H), 2.18 – 2.09 (m, 2H), 1.64 – 1.53 (m, 3H). **^{13}C NMR** (101 MHz, Chloroform-*d*) δ 153.3, 141.2, 138.6, 129.7, 118.8, 114.7, 111.1, 106.0, 33.2, 32.2, 28.4. **HRMS** (ESI) m/z: $(M + H)^+$ calcd. for $C_{11}H_{15}O^+$ 163.1117, found 163.1115.

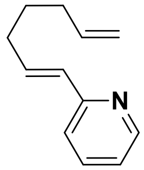

(*E*)-2-(Hepta-1,6-dien-1-yl)pyridine (**40b**). Purification of the crude mixture by short flash column chromatography (silica gel; petroleum ether /ethyl acetate = 40:1), a colorless liquid, 26.0 mg, 75% yield. **^{1}H NMR** (400 MHz, Chloroform-*d*) δ 8.54 – 8.52 (m, 1H), 7.62 (td, J = 7.7, 1.8 Hz, 1H), 7.31 – 7.23 (m, 1H), 7.12 – 7.09 (m, 1H), 6.80 – 6.73 (m, 1H), 6.51 (dt, J = 15.7, 1.5 Hz, 1H), 5.88 – 5.77 (m, 1H), 5.06 – 4.95 (m, 2H), 2.32 – 2.26 (m, 2H), 2.17 – 2.09 (m, 2H), 1.65 – 1.57 (m, 2H). **^{13}C NMR** (101 MHz, Chloroform-*d*) δ 155.8, 149.0, 138.5, 136.8, 136.1, 129.7, 121.6, 121.1,

114.8, 33.2, 32.2, 28.1. **HRMS** (ESI) m/z: $(M + H)^+$ calcd. for $C_{12}H_{16}N^+$ 174.1277, found 174.1283.

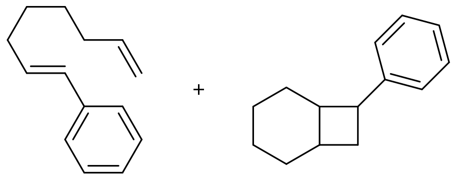

41b **41c**

(*E*)-Octa-1,7-dien-1-ylbenzene (**41b**). Purification of the crude mixture by short flash column chromatography (silica gel; petroleum ether /ethyl acetate = 60:1), a colorless liquid, 11.9 mg, 32% yield. **^{1}H NMR** (400 MHz, Chloroform-*d*) δ 7.43 – 7.17 (m, 13H), 6.31 – 6.23 (m, 1H), 5.92 – 5.82 (m, 1H), 5.11 – 4.95 (m, 2H), 3.55 (q, *J* = 9.0 Hz, 1.6H), 2.53 – 2.48 (m, 1.6H), 2.29 – 2.23 (m, 3.2H), 2.21 – 2.11 (m, 4H), 2.05 – 1.99 (m, 1.6H), 1.95 – 1.90 (t, *J* = 9.0 Hz, 1.6H), 1.76 – 1.63 (m, 4.9H), 1.56 – 1.49 (m, 9H), 1.19 – 1.10(m, 1.6H). **^{13}C NMR** (101 MHz, Chloroform-*d*) δ 145.5, 138.9, 131.0, 129.9, 128.5, 128.2, 126.8, 126.7, 125.9, 125.7, 114.4, 41.1, 40.3, 33.7, 33.1, 32.9, 29.6, 29.4, 28.9, 28.5, 26.1, 23.9, 22.3. **HRMS** (ESI) m/z: $(M + H)^+$ calcd. for $C_{14}H_{19}^+$ 187.1481, found 187.1479.

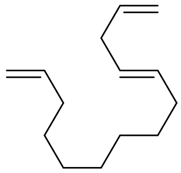

(*E*)-Tetradeca-1,4,13-triene (42b). Purification of the crude mixture by short flash column chromatography (silica gel; petroleum ether /ethyl acetate = 60:1), a colorless liquid, 26.5 mg, 69% yield. **^{1}H NMR** (400 MHz, Chloroform-*d*) δ 5.88 – 5.76 (m, 2H), 5.49 – 5.37 (m, 2H), 5.04 – 4.85 (m, 4H), 2.74 (t, *J* = 5.7 Hz, 2H), 2.07 – 1.97 (m, 4H), 1.39 – 1.26 (m, 10H). **^{13}C NMR** (101 MHz, Chloroform-*d*) δ 139.2, 137.5, 131.8, 127.5, 114.7, 114.1, 36.8, 33.8, 32.6, 29.5, 29.3, 29.1, 29.1, 28.9. **HRMS** (EI) m/z: $(M)^+$ calcd. for $C_{14}H_{24}^+$ 192.1873, found 192.1877.

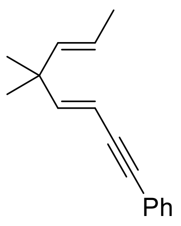

((3*E*,6*E*)-5,5-Dimethylocta-3,6-dien-1-yn-1-yl)benzene (43b). Purification of the crude mixture by short flash column chromatography (silica gel; petroleum ether /ethyl acetate = 40:1), a colorless liquid, 27.7 mg, 66% yield. **^{1}H NMR** (400 MHz, Chloroform-*d*) δ 7.49 – 7.40 (m, 2H), 7.31 (dd, *J* = 5.4, 2.0 Hz, 3H), 6.27 (d, *J* = 16.1 Hz, 1H), 5.66 (d, *J* = 16.2 Hz, 1H), 5.47 – 5.44 (m, 2H), 1.79 – 1.68 (m, 3H), 1.16 (s, 6H). **^{13}C NMR** (101 MHz, Chloroform-*d*) δ 153.4, 138.7, 131.6, 128.4, 128.0, 123.9, 122.5, 106.3, 88.8, 88.6, 39.5, 27.2, 18.2. **HRMS** (ESI) m/z: (M + H)$^+$ calcd. for $C_{16}H_{19}^+$ 211.1481, found 211.1485.

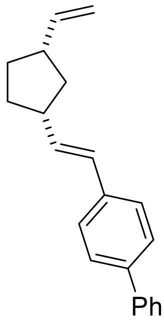

4-((*E*)-2-((1*R*,3*S*)-3-Vinylcyclopentyl)vinyl)-1,1'-biphenyl (44b). Purification of the crude mixture by short flash column chromatography (silica gel; petroleum ether /ethyl acetate = 40:1), a colorless liquid, 49.4 mg, 90% yield. **^{1}H NMR** (400 MHz, Chloroform-d) 1H NMR (400 MHz, Chloroform-d) δ 7.64 (d, *J* = 7.5 Hz, 2H), 7.58 (d, *J* = 8.0 Hz, 2H), 7.52 – 7.43 (m, 4H), 7.40 – 7.34 (m, 1H), 6.45 (d, *J* = 15.8 Hz, 1H), 6.32 – 6.26 (m, 1H), 5.93 – 5.85 (m, 1H), 5.06 (dt, *J* = 17.0, 1.5 Hz, 1H), 4.96 (d, *J* = 10.2, 1H), 2.84 – 2.71 (m, 1H), 2.70 – 2.61 (m, 1H), 2.12 – 2.05 (m, 1H), 2.03 – 1.86 (m, 2H), 1.66 – 1.52 (m, 1H), 1.39 – 1.28 (m, 2H). **^{13}C NMR** (101 MHz, Chloroform-*d*) δ 143.2, 140.9, 139.6, 136. 9, 135.5, 128.8, 127.7, 127.2, 127.2, 126.9, 126.4, 112.5, 44.4, 43.8, 40.6, 32.2, 31.8. **HRMS** (EI) m/z: (M)$^+$ calcd. for $C_{21}H_{22}^+$ 274.1716, found 274.1720.

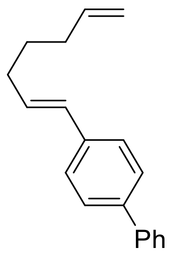

(*E*)-4-(Hepta-1,6-dien-1-yl)-1,1'-biphenyl (45b). Purification of the crude mixture by short flash column chromatography (silica gel; petroleum ether /ethyl acetate = 40:1), a colorless liquid, **SC-I**: 0.93 g, 84% yield; **SC-II**: 45.4 mg, 92% yield. **^{1}H NMR** (400 MHz, Chloroform-*d*) δ 7.65 – 7.59 (m, 2H), 7.58 – 7.55 (m, 2H), 7.49 – 7.41 (m, 4H), 7.39 – 7.32 (m, 1H), 6.45 (d, *J* = 15.9 Hz, 1H), 6.33 – 6.26 (m, 1H), 5.93 – 5.82 (m, 1H), 5.14 – 4.96 (m, 2H), 2.28 (q, *J* = 7.2 Hz, 2H), 2.16 (q, *J* = 6.7 Hz, 2H), 1.66 – 1.59 (m, 2H). **^{13}C NMR** (101 MHz, Chloroform-*d*) δ 140.9, 139.6, 138.7, 137.0, 130.9, 129.7, 128.8, 127.2, 127.2, 126.9, 126.4, 114.7, 33.3, 32.5, 28.6. **HRMS** (EI) m/z: $(M)^+$ calcd. for $C_{19}H_{2}^{+}$ 248.1560, found 248.1561.

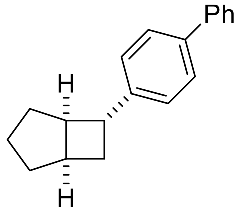

(±)-6-([1,1'-Biphenyl]-4-yl)bicyclo[3.2.0]heptane (45c). Purification of the crude mixture by short flash column chromatography (silica gel; petroleum ether /ethyl acetate = 40:1), a colorless liquid, 47.7 mg, 96% yield. **^{1}H NMR** (400 MHz, Chloroform-*d*) δ 7.66 – 7.60 (m, 2H), 7.60 – 7.55 (m, 2H), 7.48 – 7.44 (m, 2H), 7.40 – 7.35 (m, 3H), 3.03 – 2.98 (m, 1H), 2.90 – 2.81 (m, 2H), 2.39 – 2.32 (m, 1H), 2.10 – 1.89 (m, 3H), 1.79 – 1.74 (m, 1H), 1.71 – 1.54 (m, 3H). **^{13}C NMR** (101 MHz, Chloroform-*d*) δ 146.8, 141.3, 138.6, 128.7, 127.1, 127.0, 127.0, 126.9, 47.0, 42.3, 34.4, 33.4, 33.1, 32.3, 25.2. **HRMS** (EI) m/z: $(M)^+$ calcd. for $C_{19}H_{20}^{+}$ 248.1560, found 248.1569.

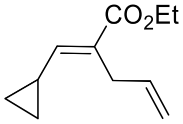

Ethyl (*E*)-2-(cyclopropylmethylene)pent-4-enoate (**46b**). Purification of the crude mixture by short flash column chromatography (silica gel; petroleum ether /ethyl acetate = 40:1), a colorless liquid, 32.1 mg, 89% yield. **^{1}H NMR** (400 MHz, Chloroform-*d*) δ 6.23 (d, *J* = 10.7 Hz, 1H), 5.93 – 5.85 (m, 1H), 5.11 – 5.00 (m, 2H), 4.19 (q, *J* = 7.1 Hz, 2H), 3.20 (dt, *J* = 6.1, 1.7 Hz, 2H), 1.67 – 1.61 (m, 1H), 1.30 (d, *J* = 7.1 Hz, 3H), 1.00 – 0.93 (m, 2H), 0.67 – 0.60 (m, 2H). **^{13}C NMR** (101 MHz, Chloroform-*d*) δ 167.5, 148.7, 135.8, 127.4, 114.9, 60.3, 30.9, 14.3, 11.5, 8.4. **HRMS** (ESI) m/z: $(M + Na)^+$ calcd. for $C_{11}H_{16}O_2Na^+$ 203.1043, found 203.1038.

Copies of NMR Spectra

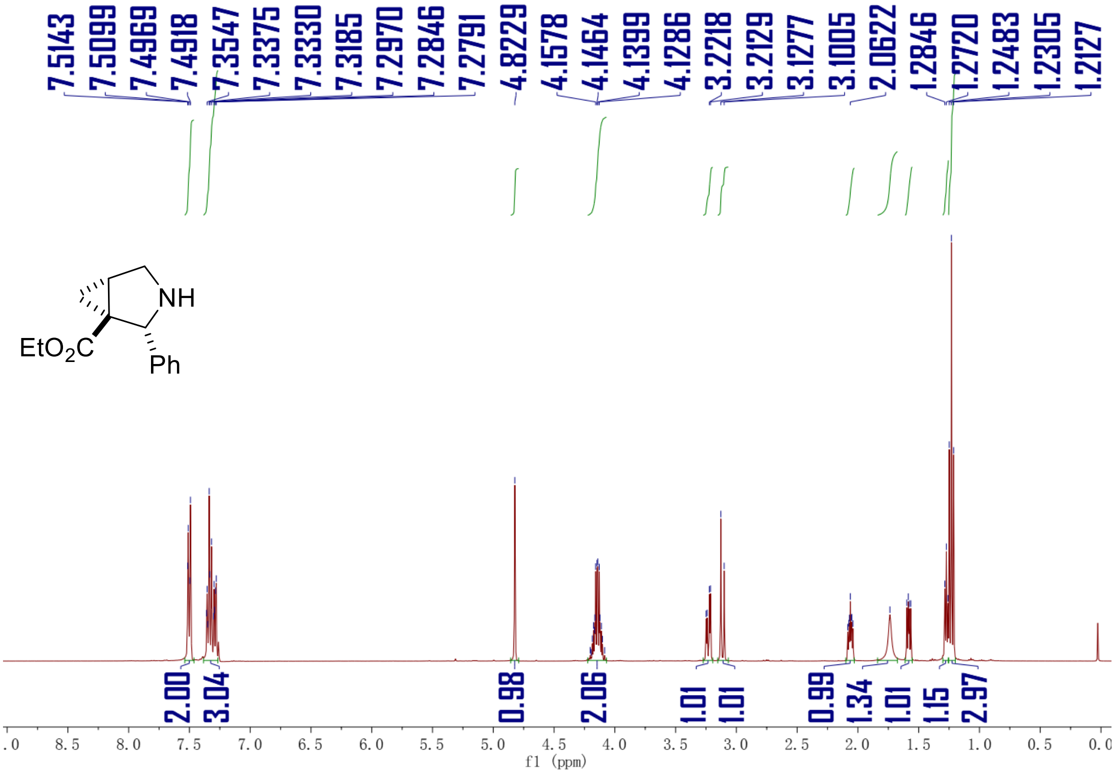

Supplementary Figure 13. ^{1}H NMR (400 MHz, $CDCl_3$) of 1a

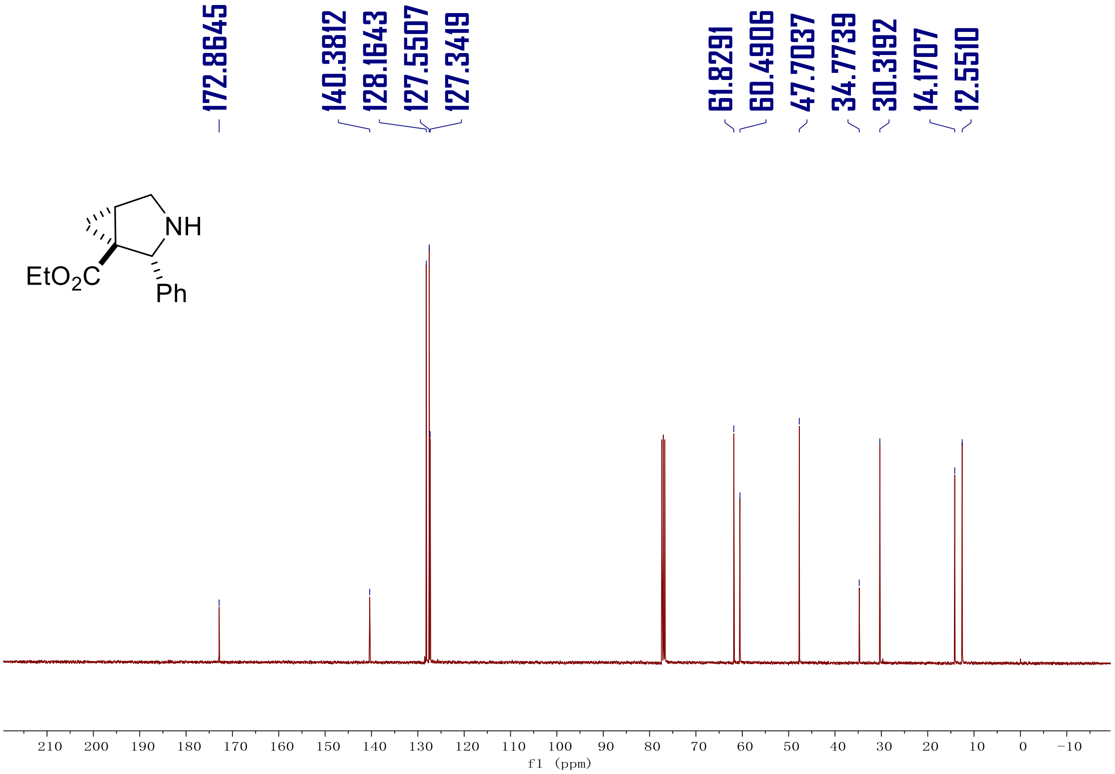

Supplementary Figure 14. ^{13}C NMR (101 MHz, $CDCl_3$) of 1a

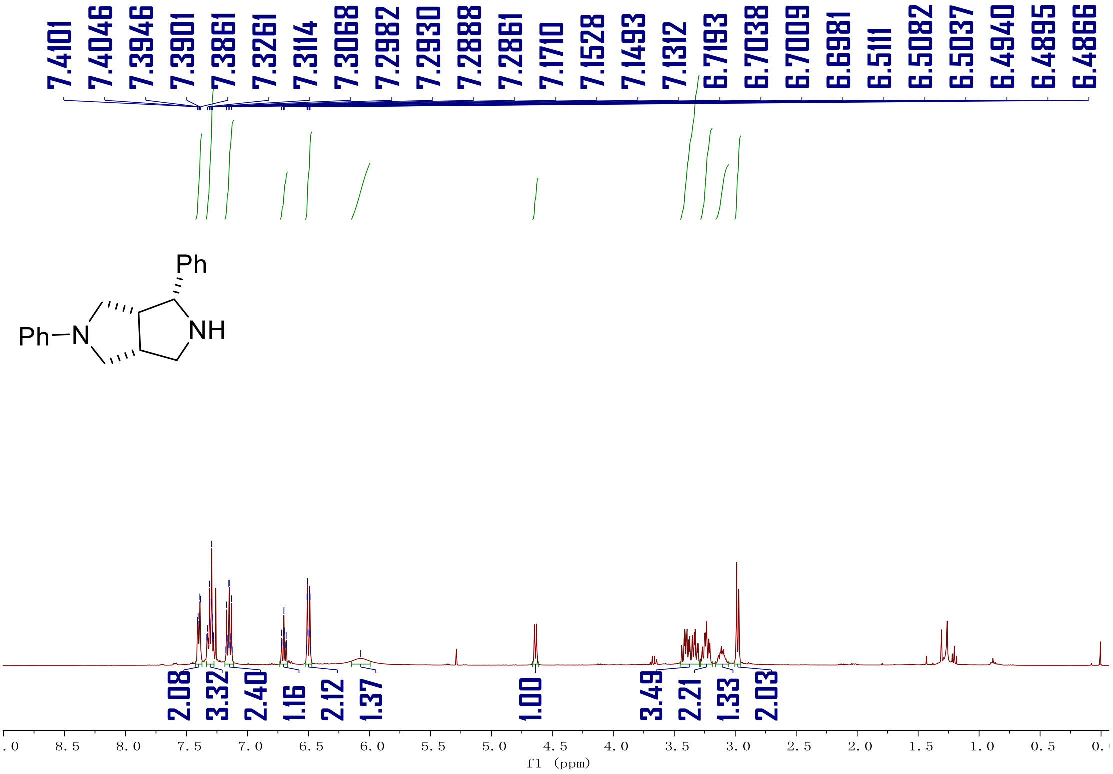

Supplementary Figure 15. ^{1}**H NMR (101 MHz, CDCl**$_{3}$**) of 2a**

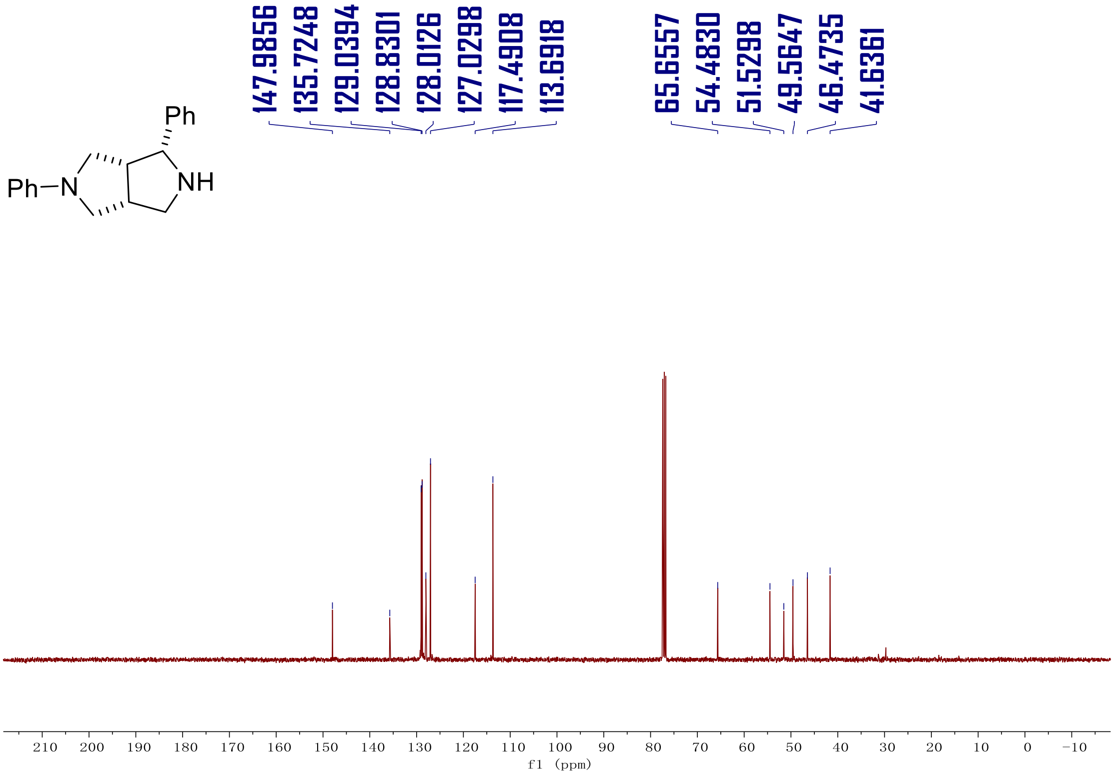

Supplementary Figure 16. ^{13}**C NMR (101 MHz, CDCl**$_{3}$**) of 2a**

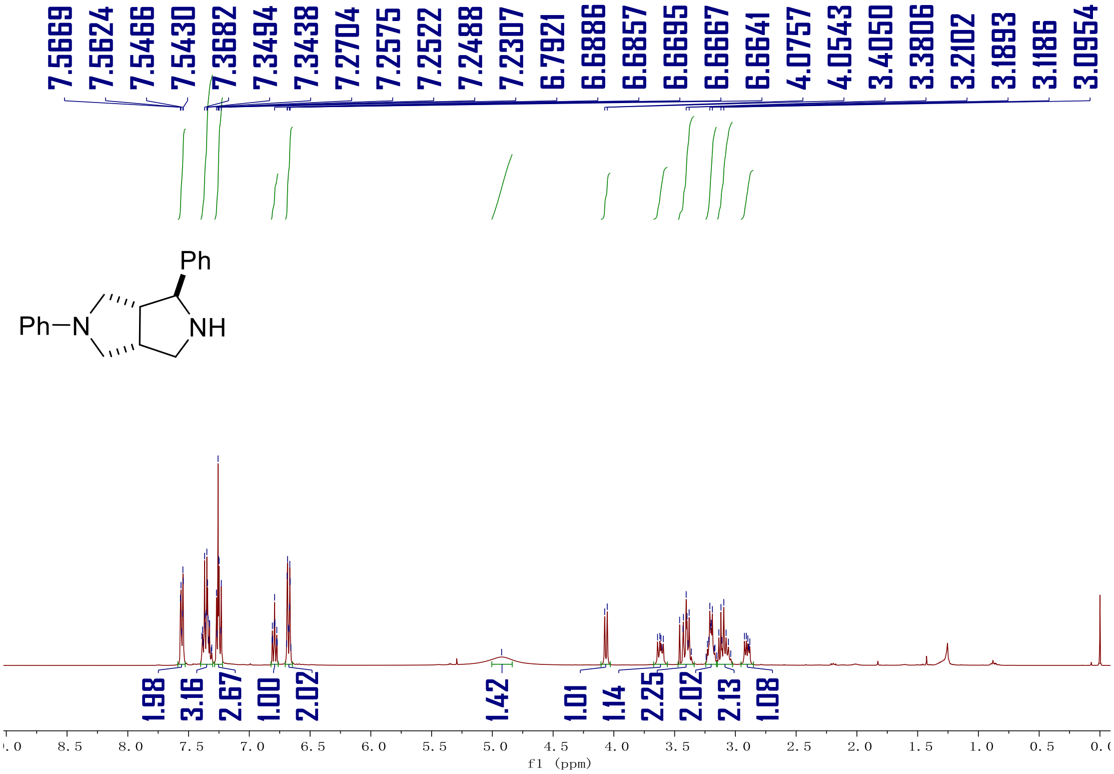

Supplementary Figure 17. ^{1}H NMR (101 MHz, $CDCl_3$) of 3a

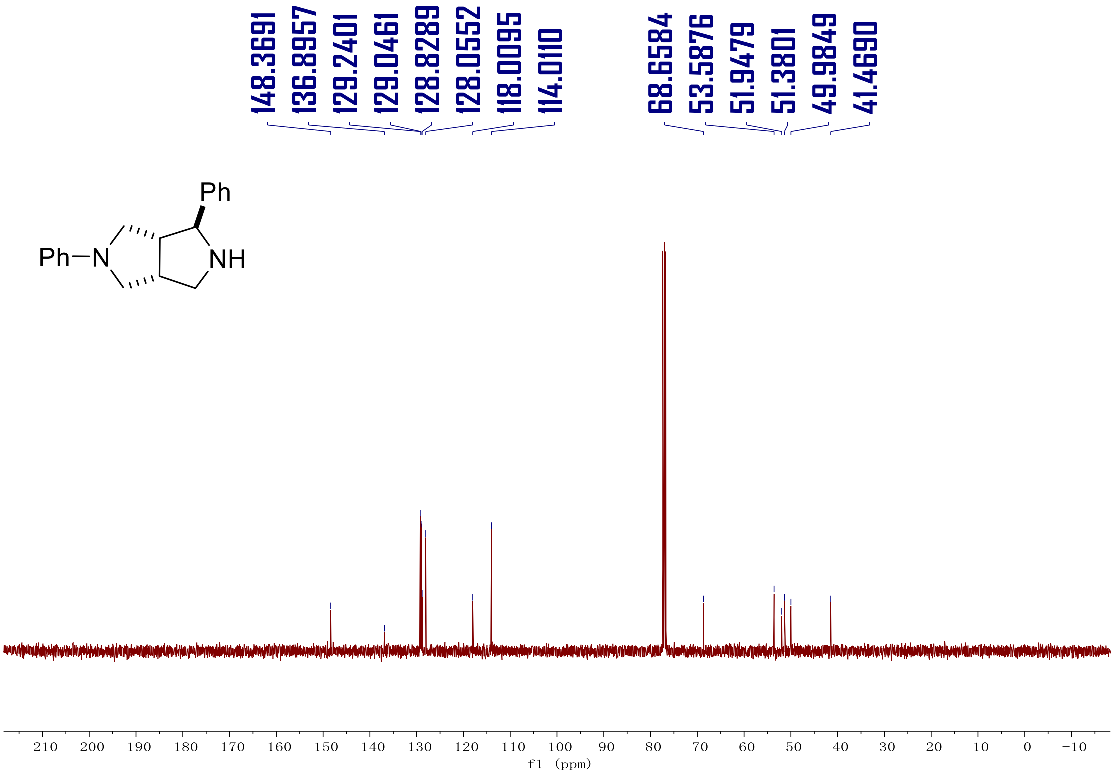

Supplementary Figure 18. ^{13}C NMR (101 MHz, $CDCl_3$) of 3a

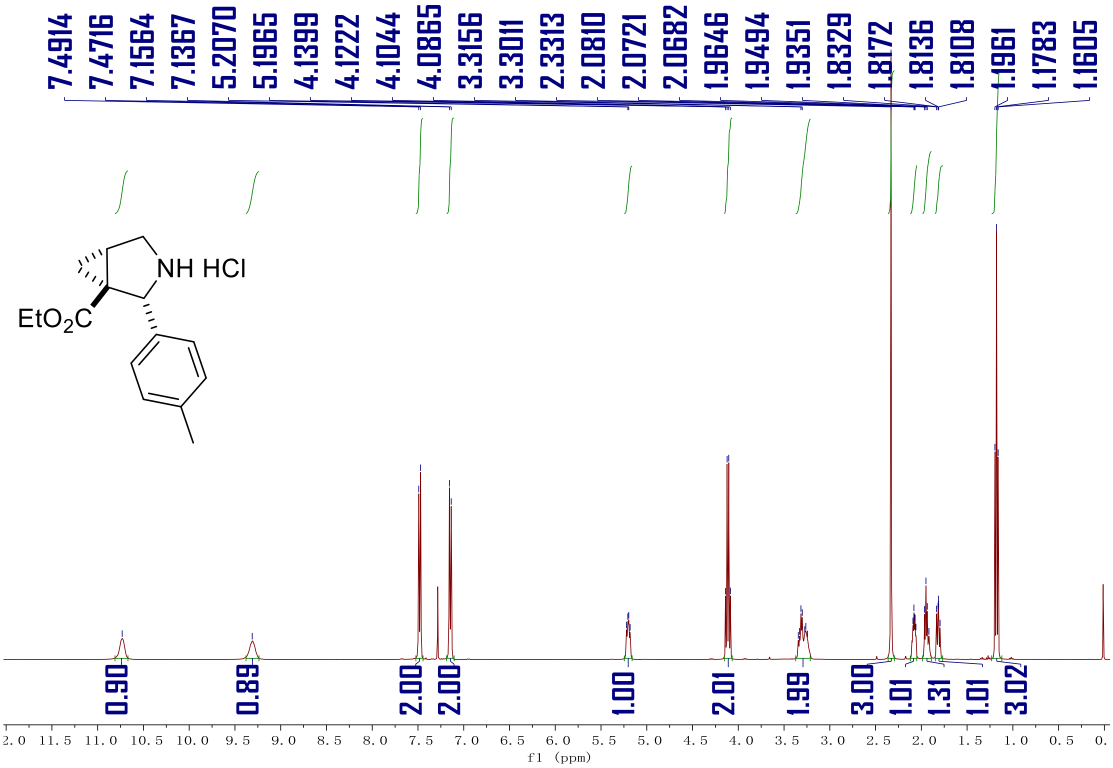

Supplementary Figure 19. ^{1}**H NMR (400 MHz, CDCl**$_3$**) of 4a**

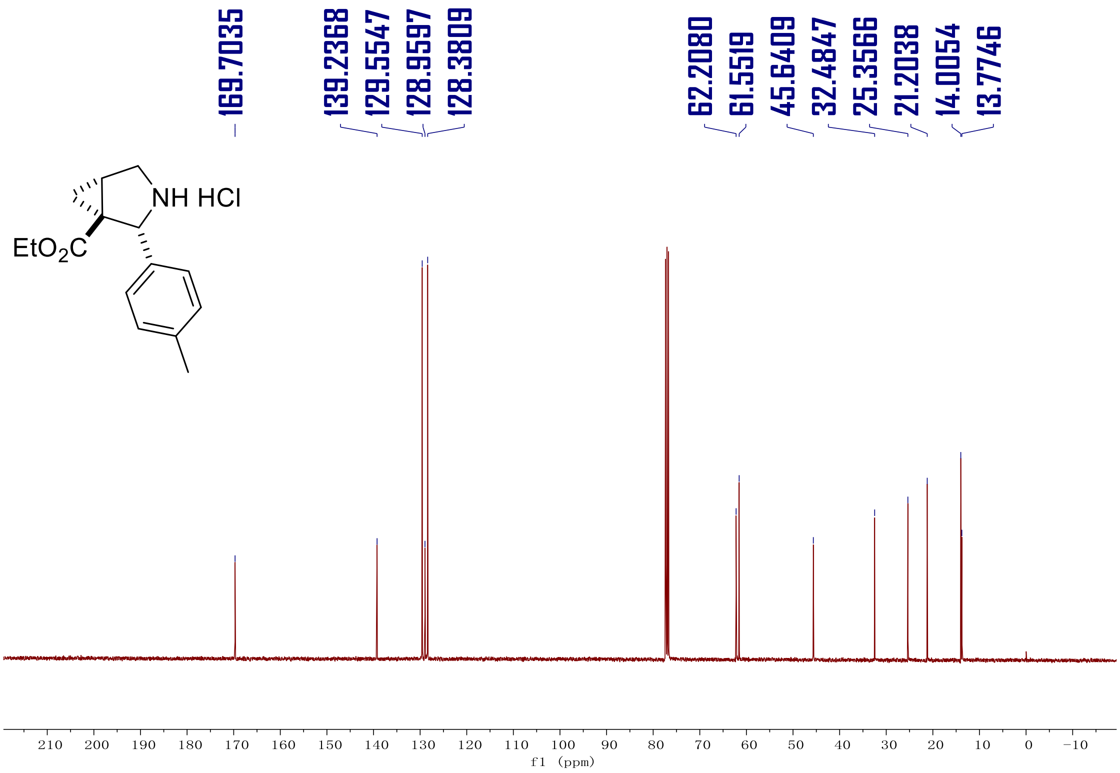

Supplementary Figure 20. ^{13}**C NMR (101 MHz, CDCl**$_3$**) of 4a**

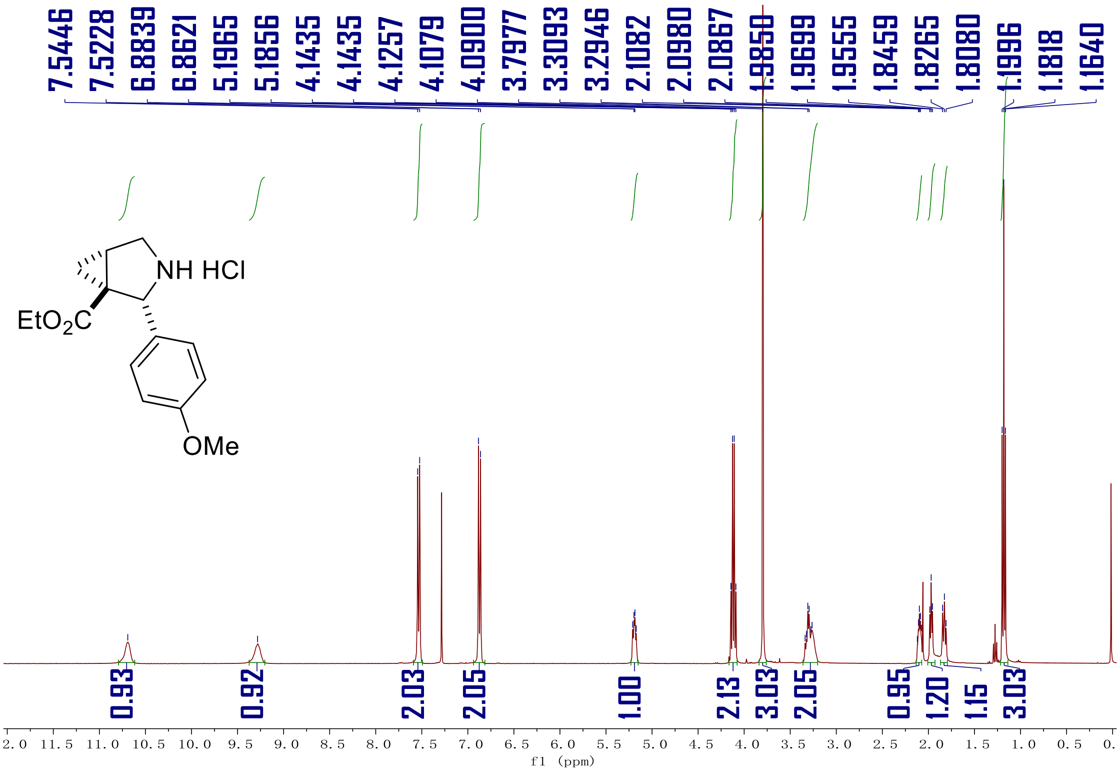

Supplementary Figure 21. ^{1}H NMR (400 MHz, $CDCl_3$) of 5a

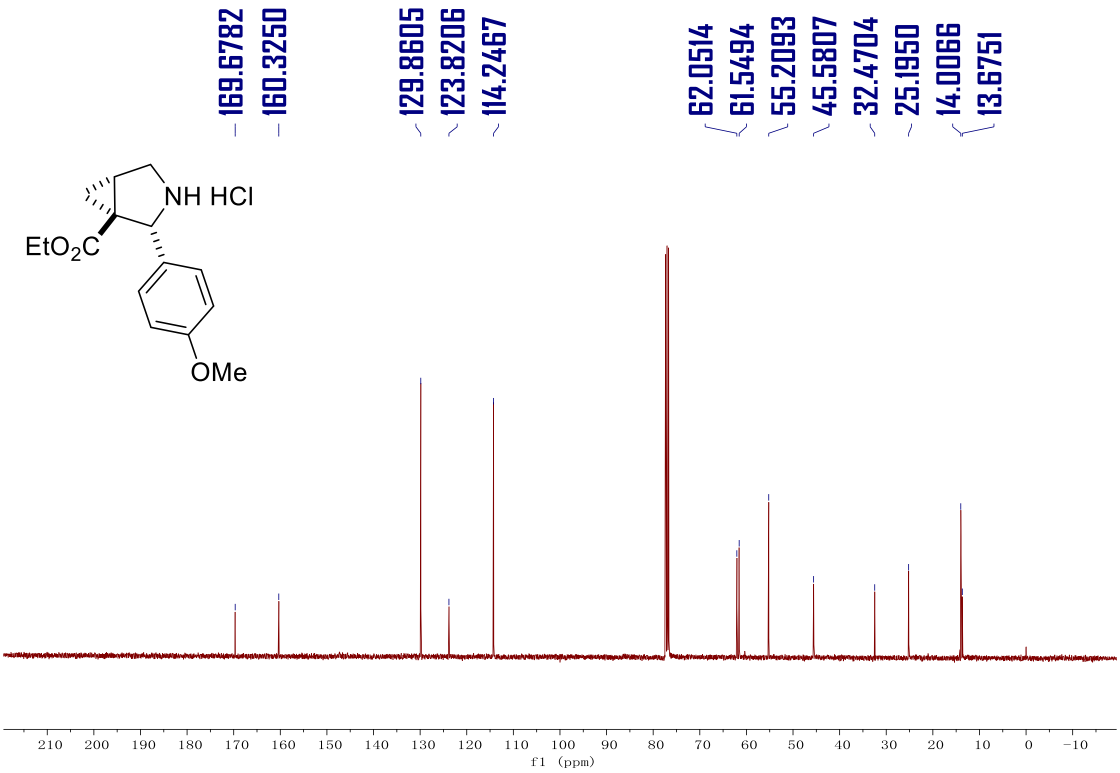

Supplementary Figure 22. ^{13}C NMR (101 MHz, $CDCl_3$) of 5a

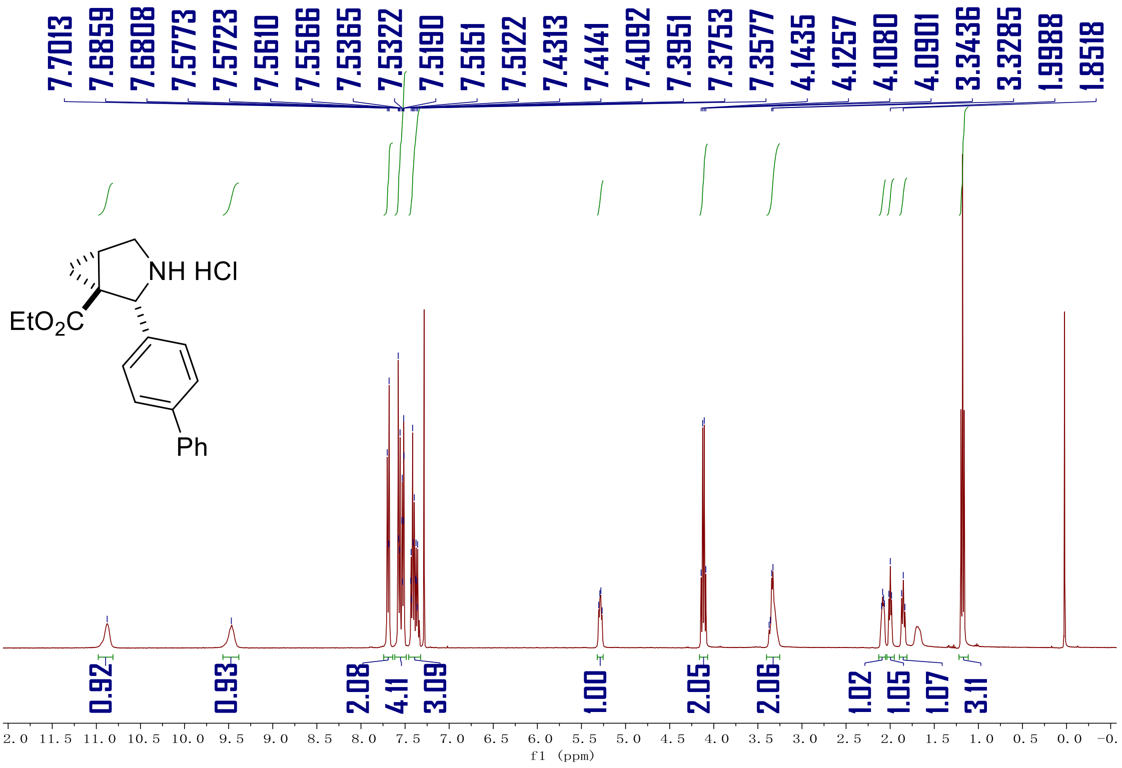

Supplementary Figure 23. ^{1}H NMR (400 MHz, $CDCl_3$) of 6a

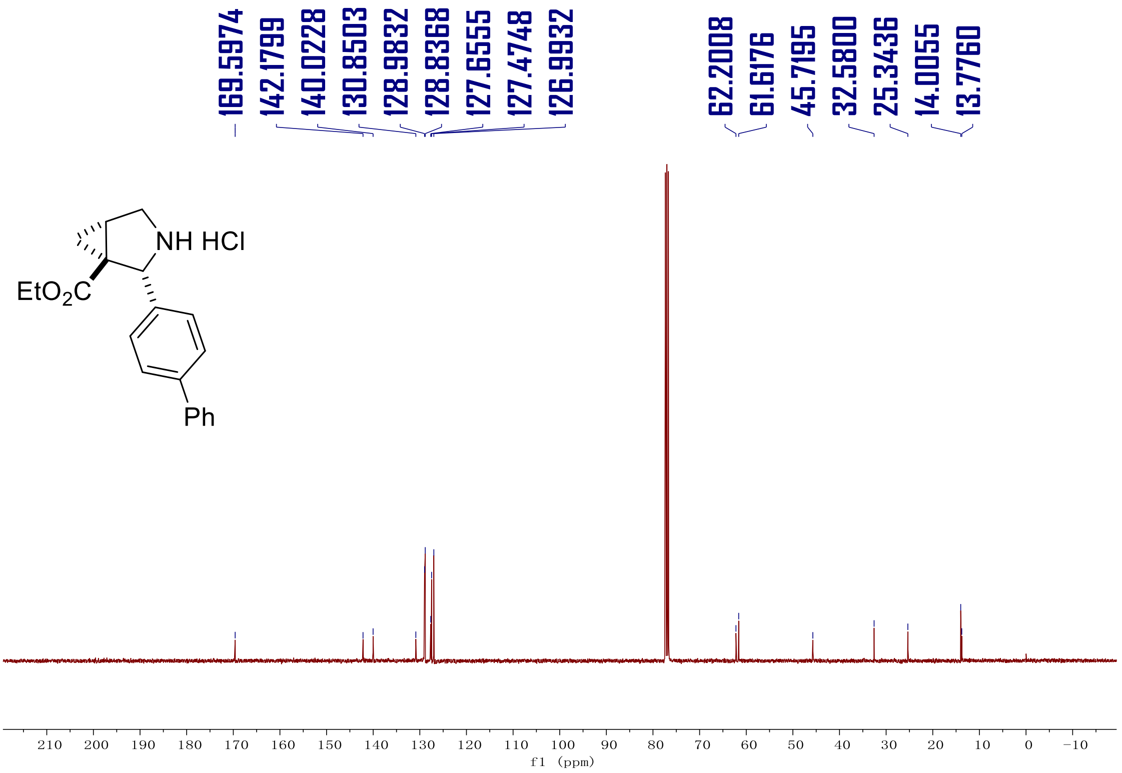

Supplementary Figure 24. ^{13}C NMR (101 MHz, $CDCl_3$) of 6a

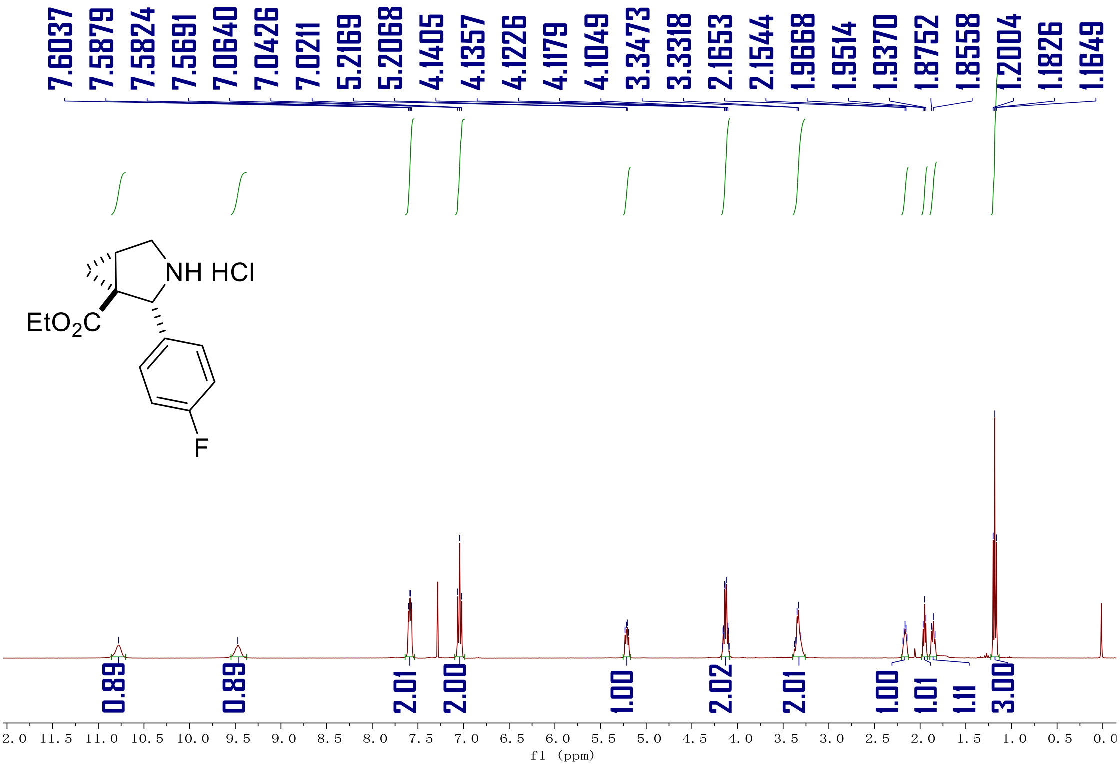

Supplementary Figure 25. ^{1}**H NMR (400 MHz,** $CDCl_3$**) of 7a**

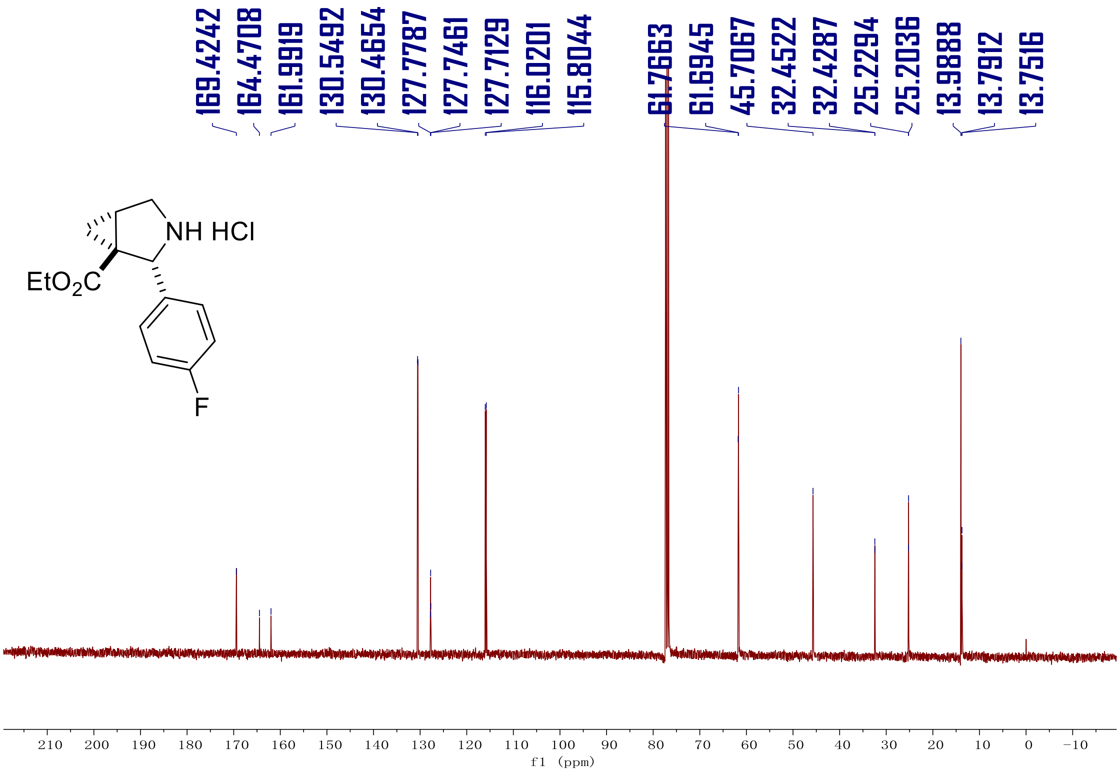

Supplementary Figure 26. ^{13}**C NMR (101 MHz,** $CDCl_3$**) of 7a**

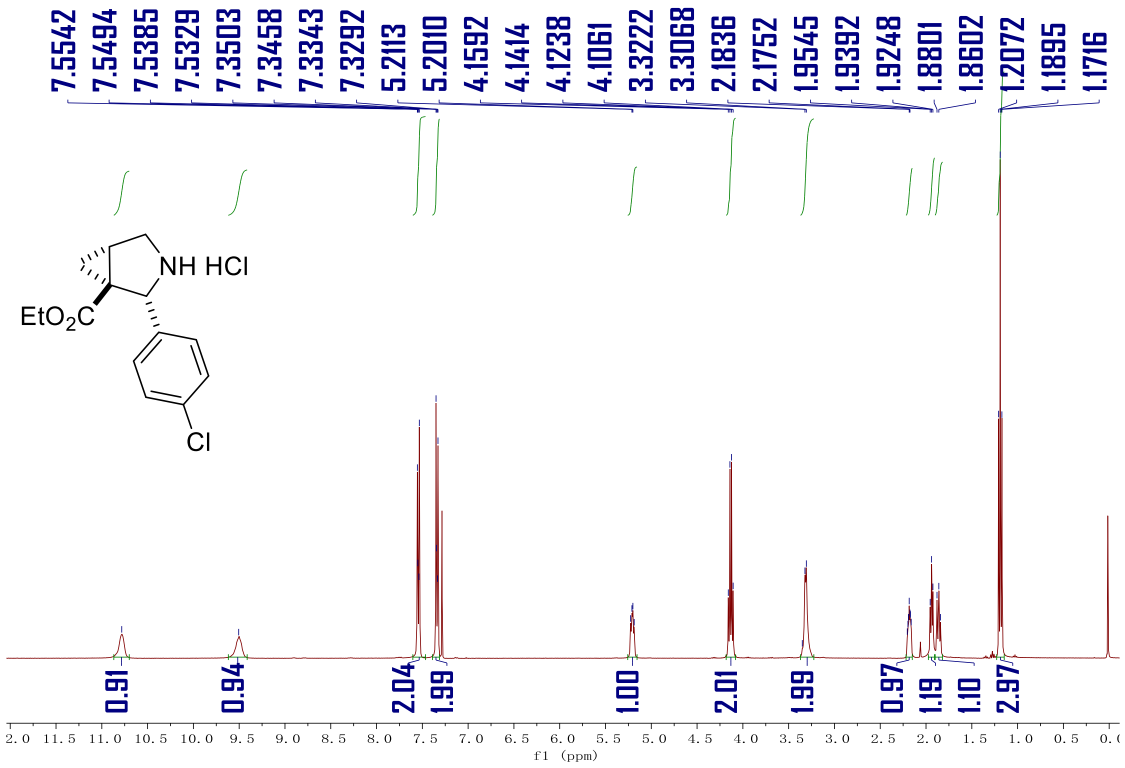

Supplementary Figure 27. ^{1}H NMR (400 MHz, $CDCl_3$) of 8a

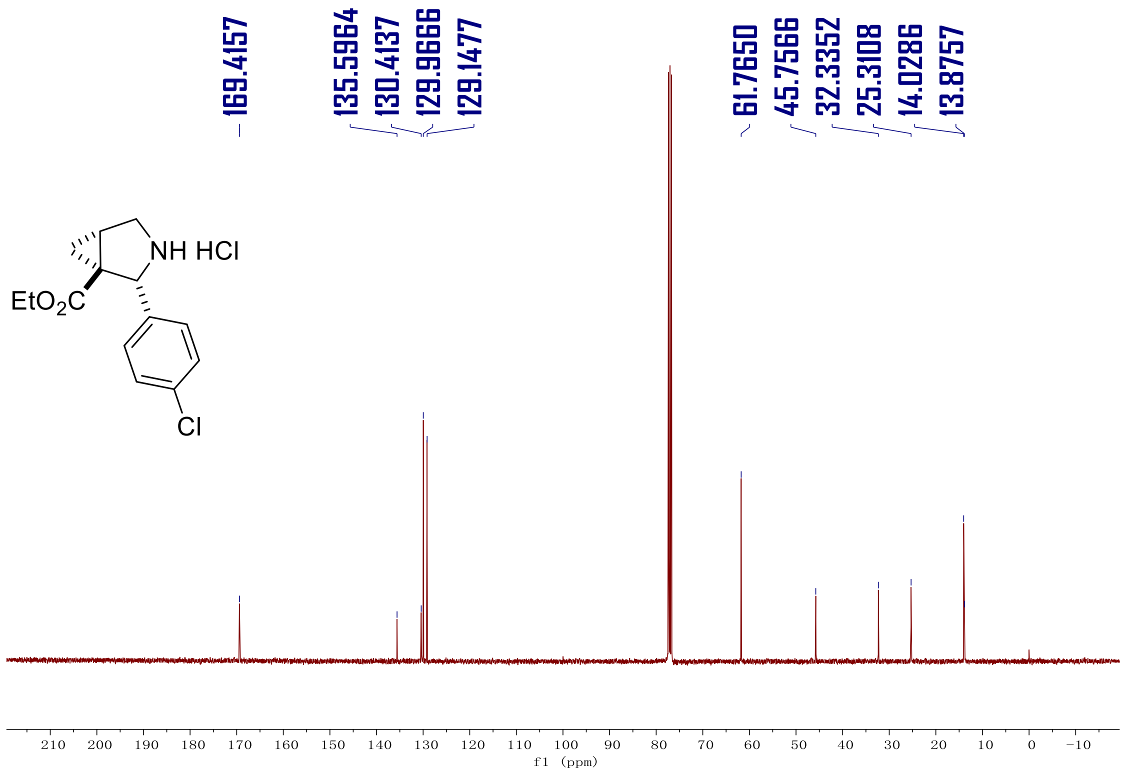

Supplementary Figure 28. ^{13}C NMR (101 MHz, $CDCl_3$) of 8a

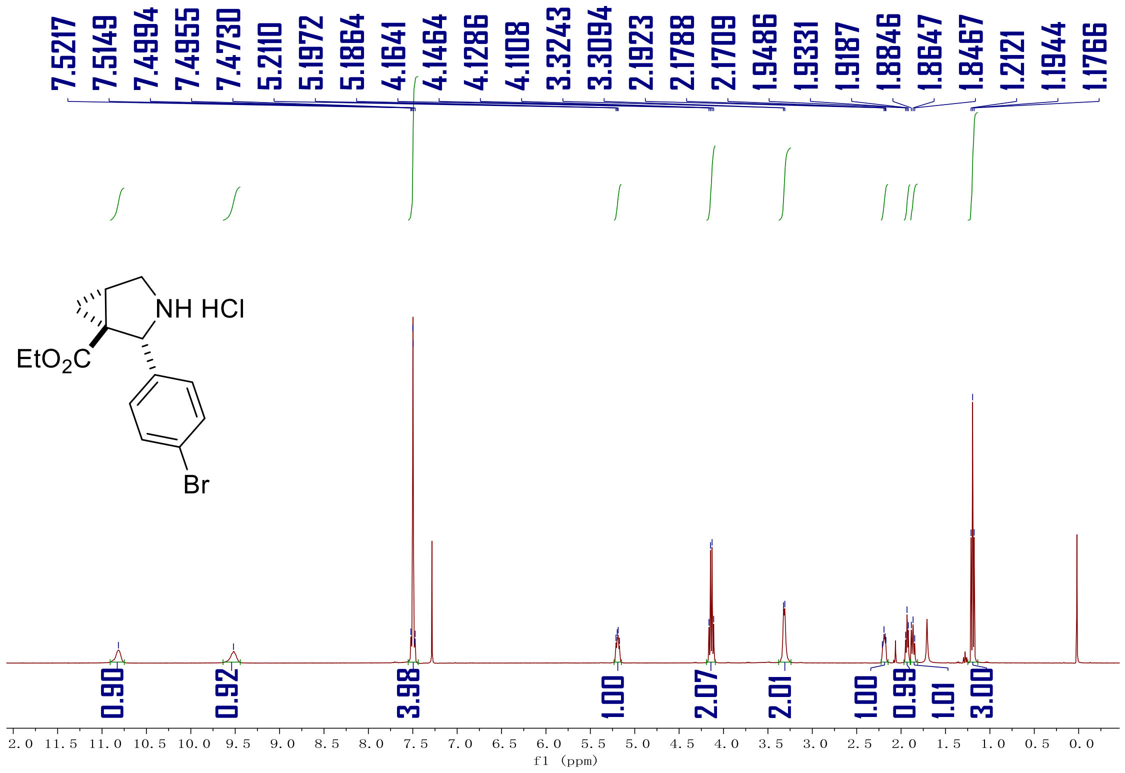

Supplementary Figure 29. ^{1}H NMR (400 MHz, $CDCl_3$) of 9a

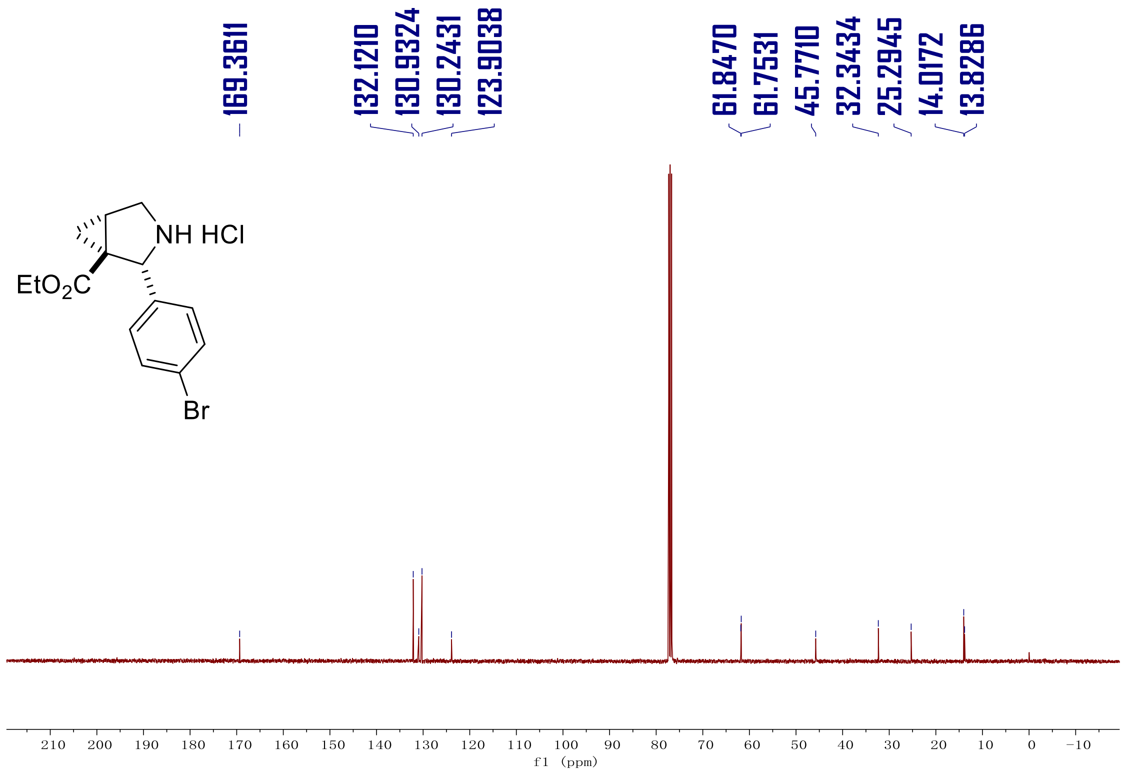

Supplementary Figure 30. ^{13}C NMR (101 MHz, $CDCl_3$) of 9a

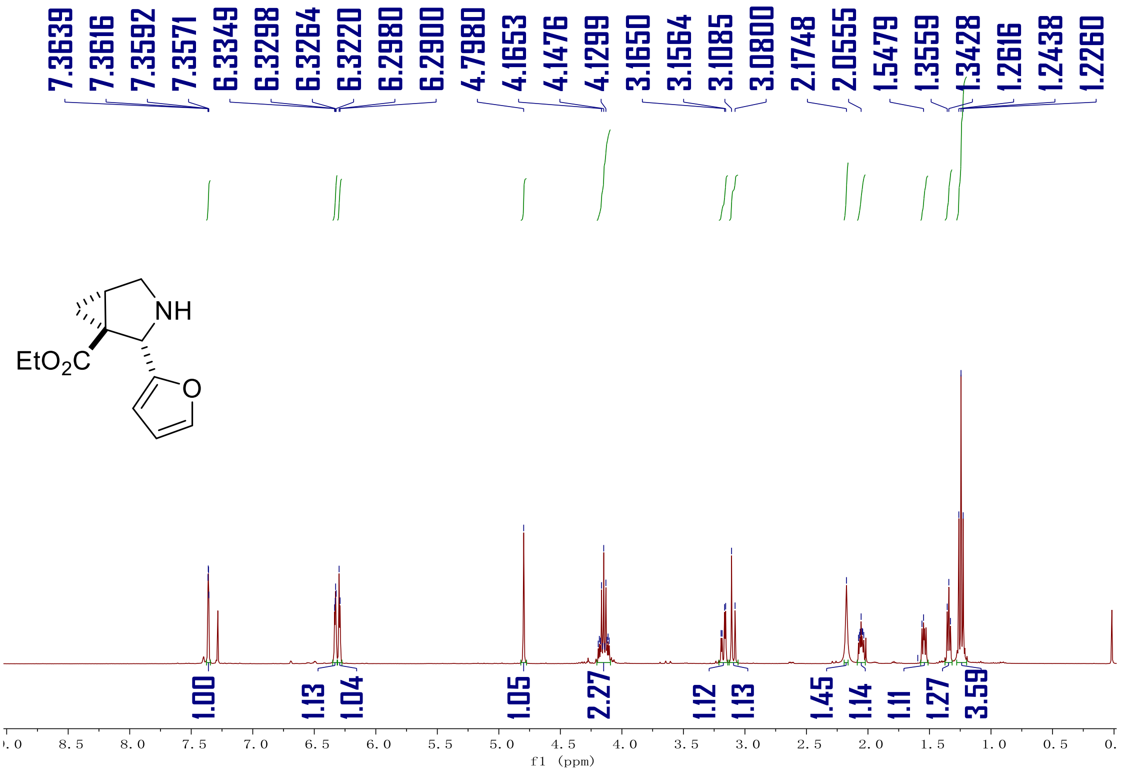

Supplementary Figure 31. ^{1}**H NMR (400 MHz, CDCl**$_3$**) of 10a**

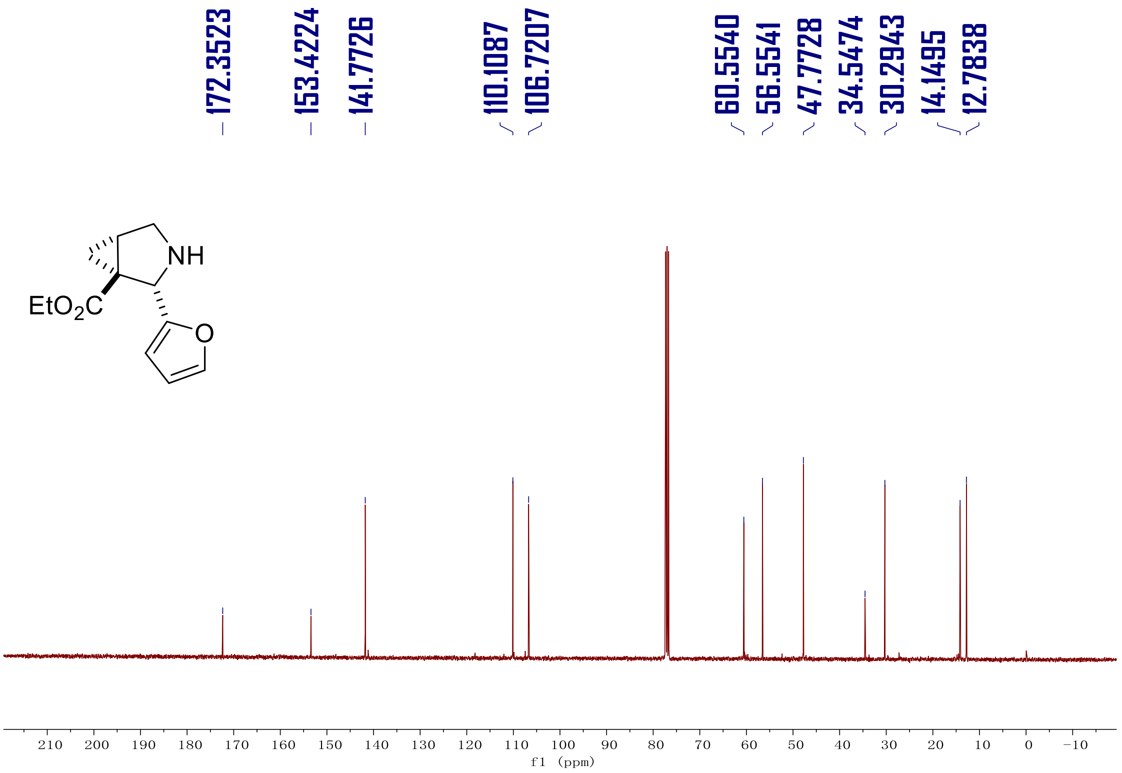

Supplementary Figure 32. ^{13}**C NMR (101 MHz, CDCl**$_3$**) of 10a**

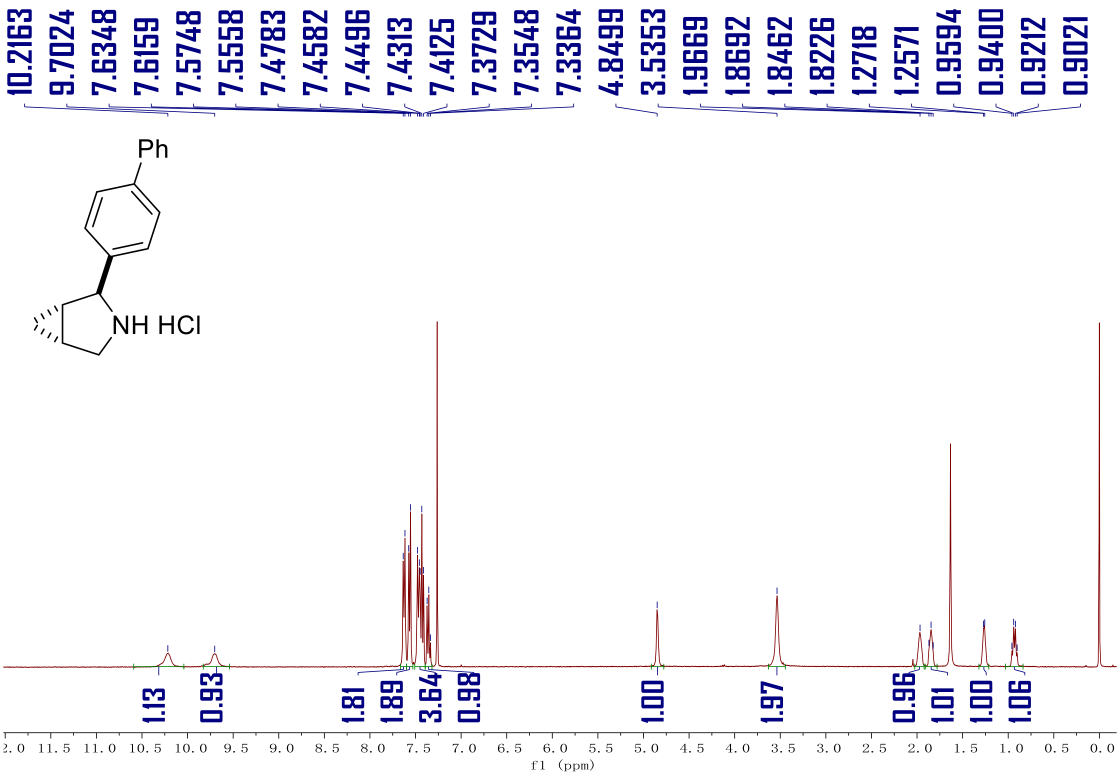

Supplementary Figure 33. ^{1}H NMR (400 MHz, $CDCl_3$) of 12a

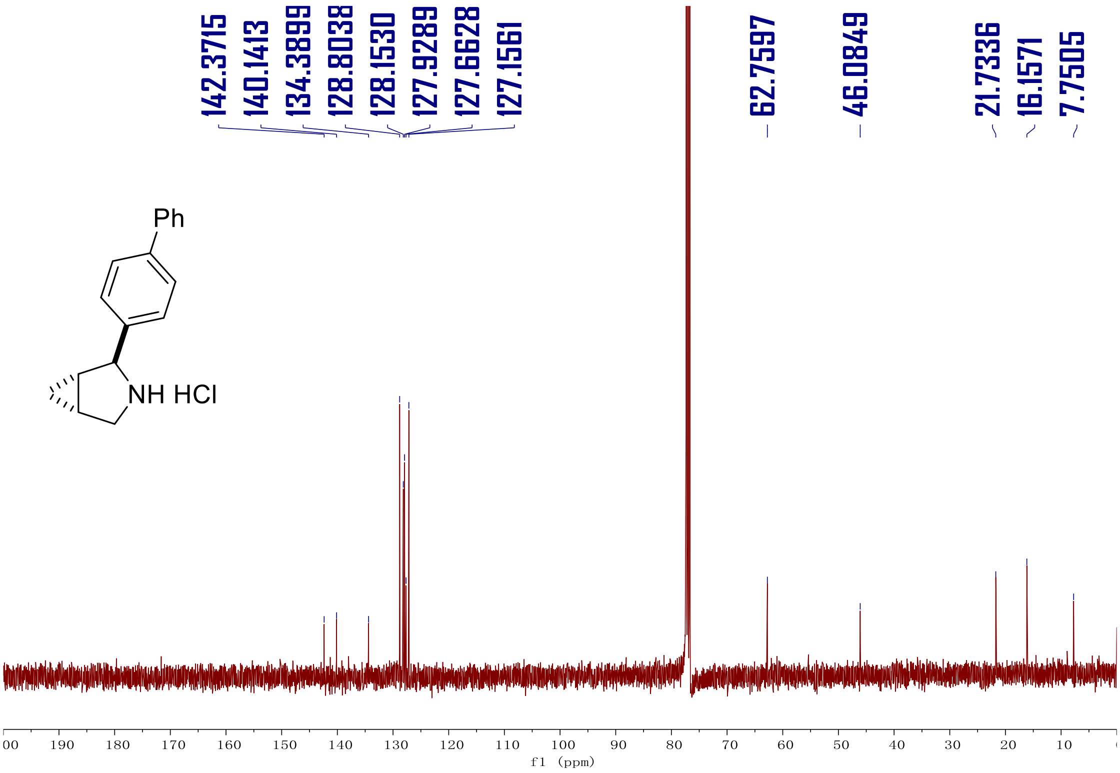

Supplementary Figure 34. ^{13}C NMR (101 MHz, $CDCl_3$) of 12a

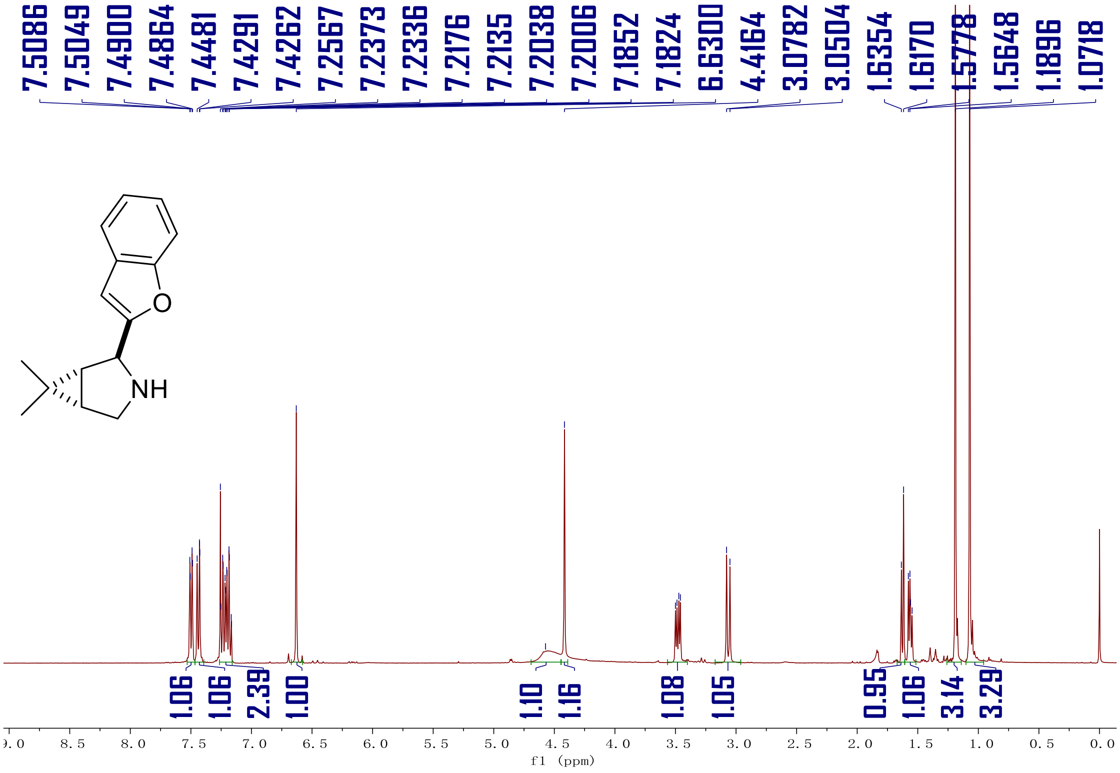

Supplementary Figure 35. ^{1}H NMR (400 MHz, $CDCl_3$) of 13a

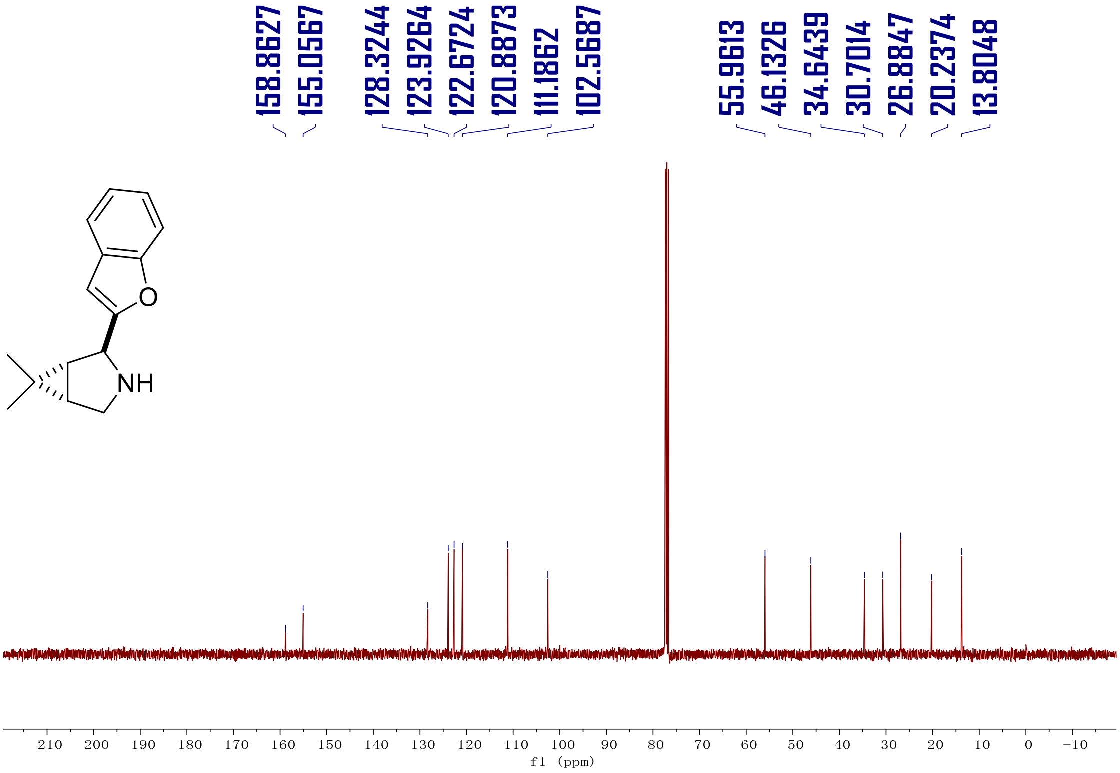

Supplementary Figure 36. ^{13}C NMR (101 MHz, $CDCl_3$) of 13a

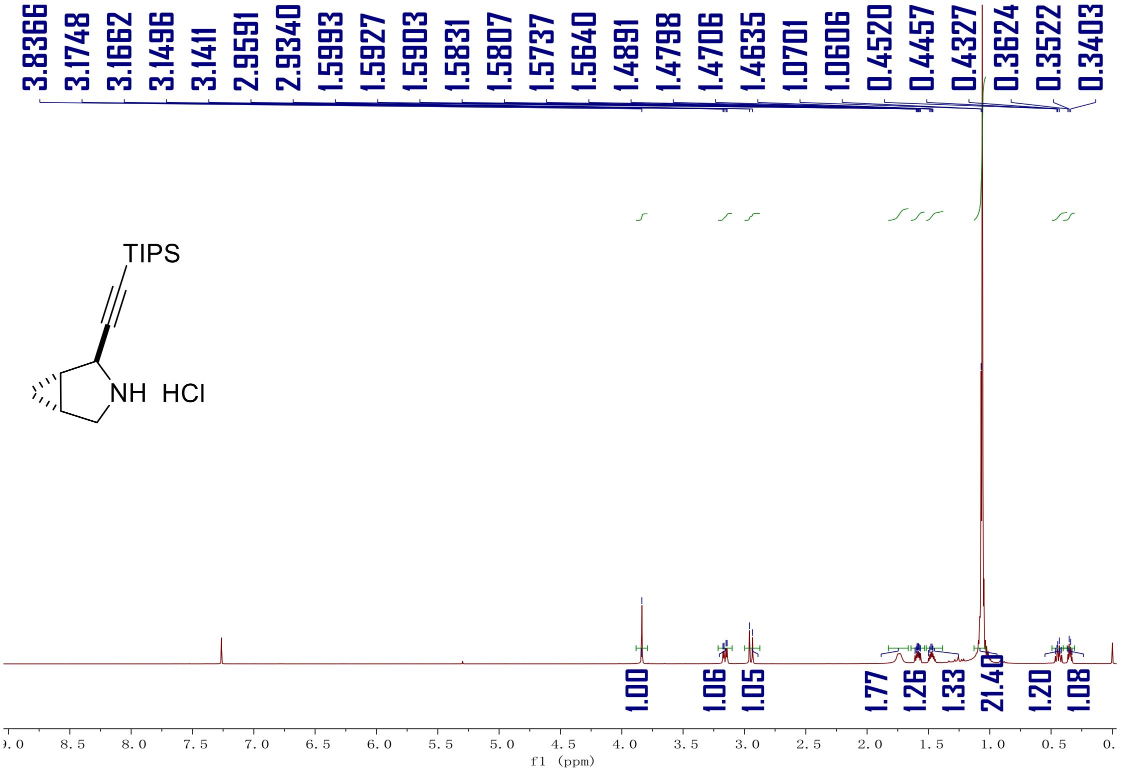

Supplementary Figure 37. ^{1}H NMR (400 MHz, $CDCl_3$) of 14a

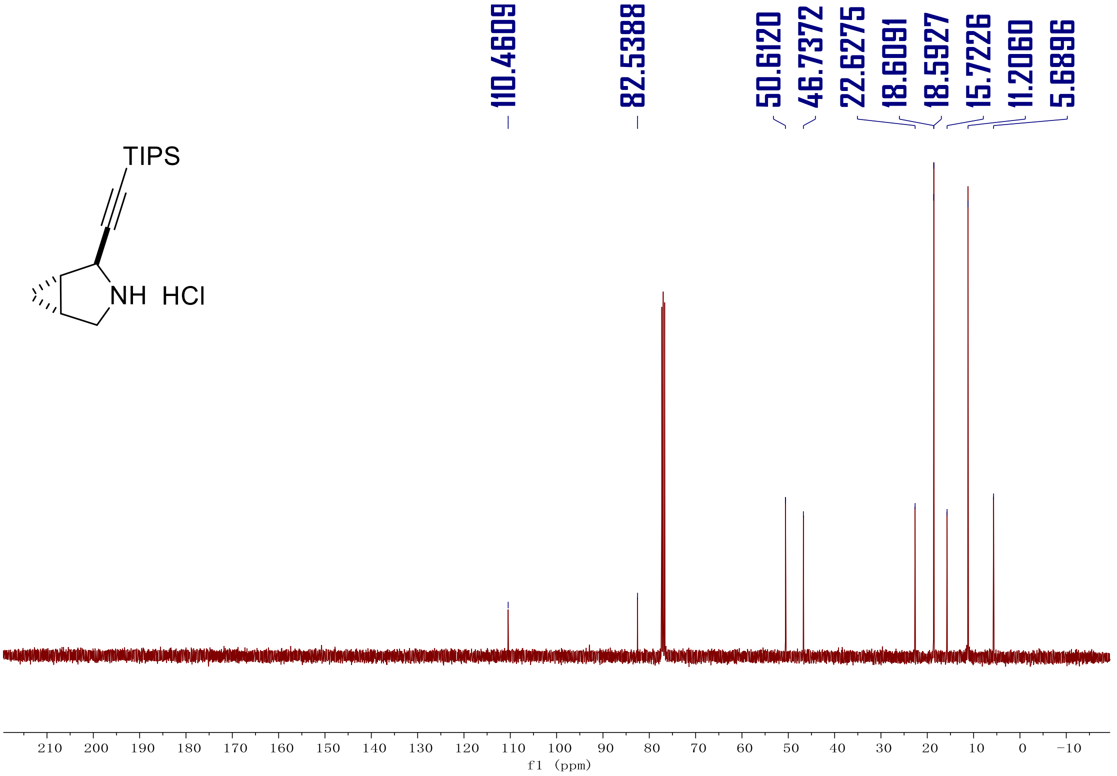

Supplementary Figure 38. ^{13}C NMR (101 MHz, $CDCl_3$) of 14a

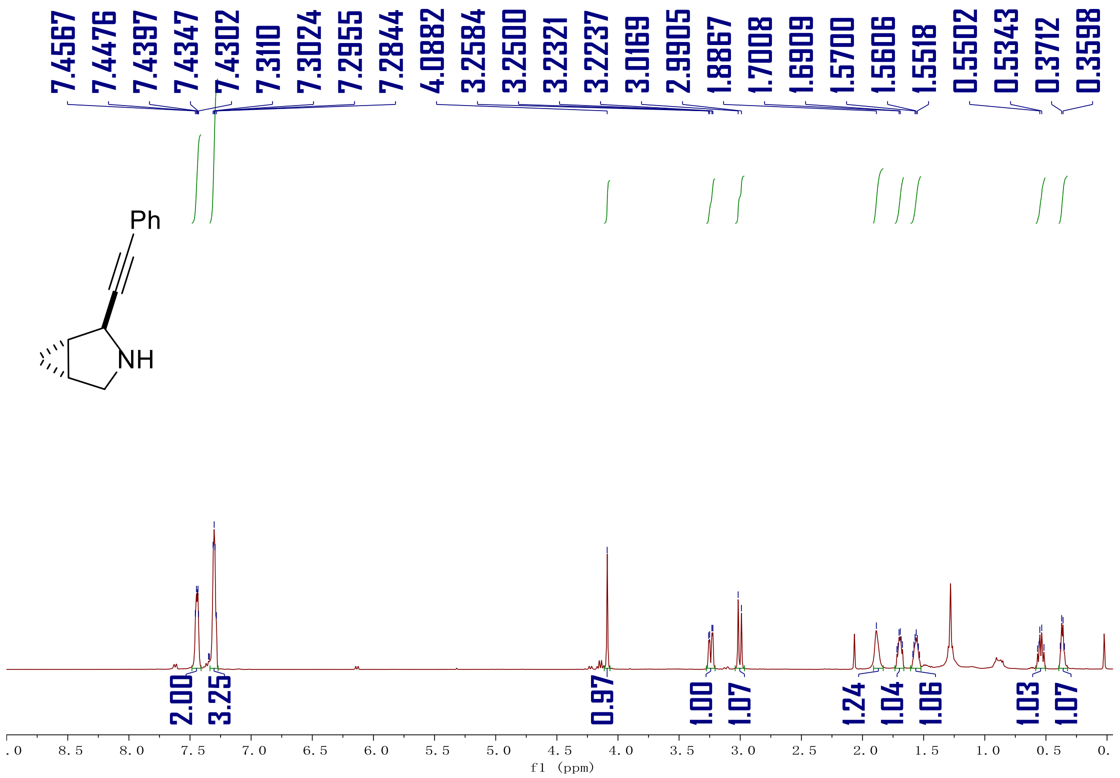

Supplementary Figure 39. ^{1}H NMR (400 MHz, $CDCl_3$) of 15a

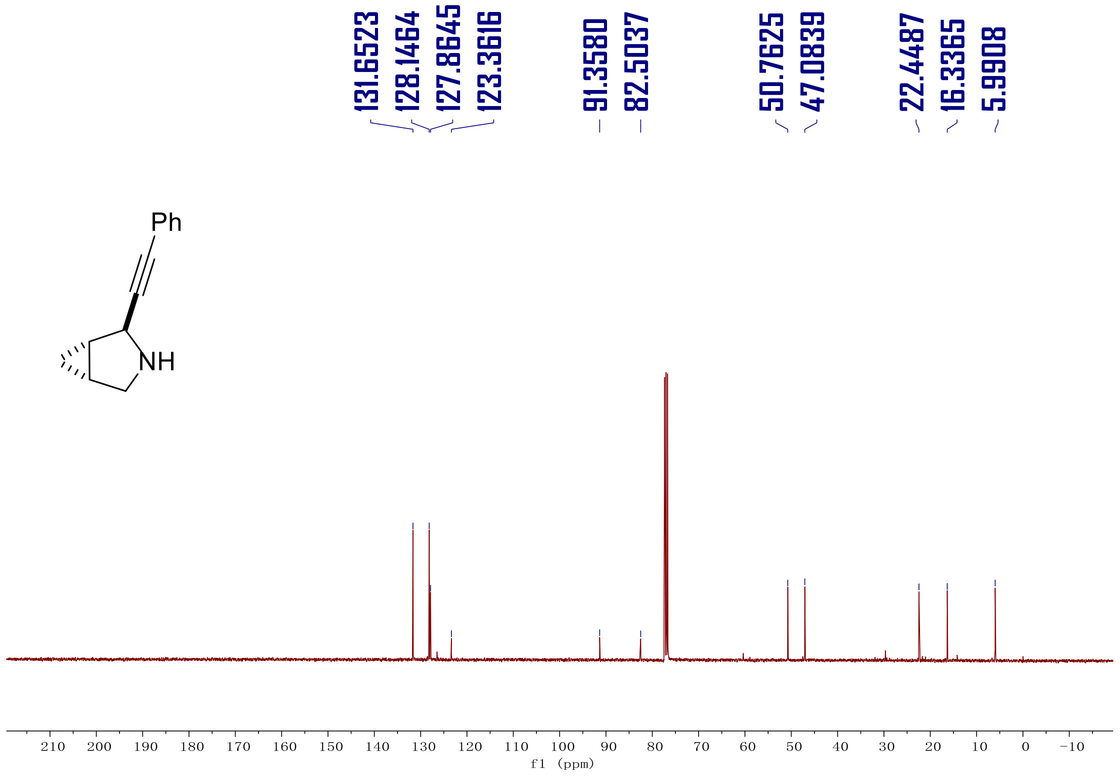

Supplementary Figure 40. ^{13}C NMR (101 MHz, $CDCl_3$) of 15a

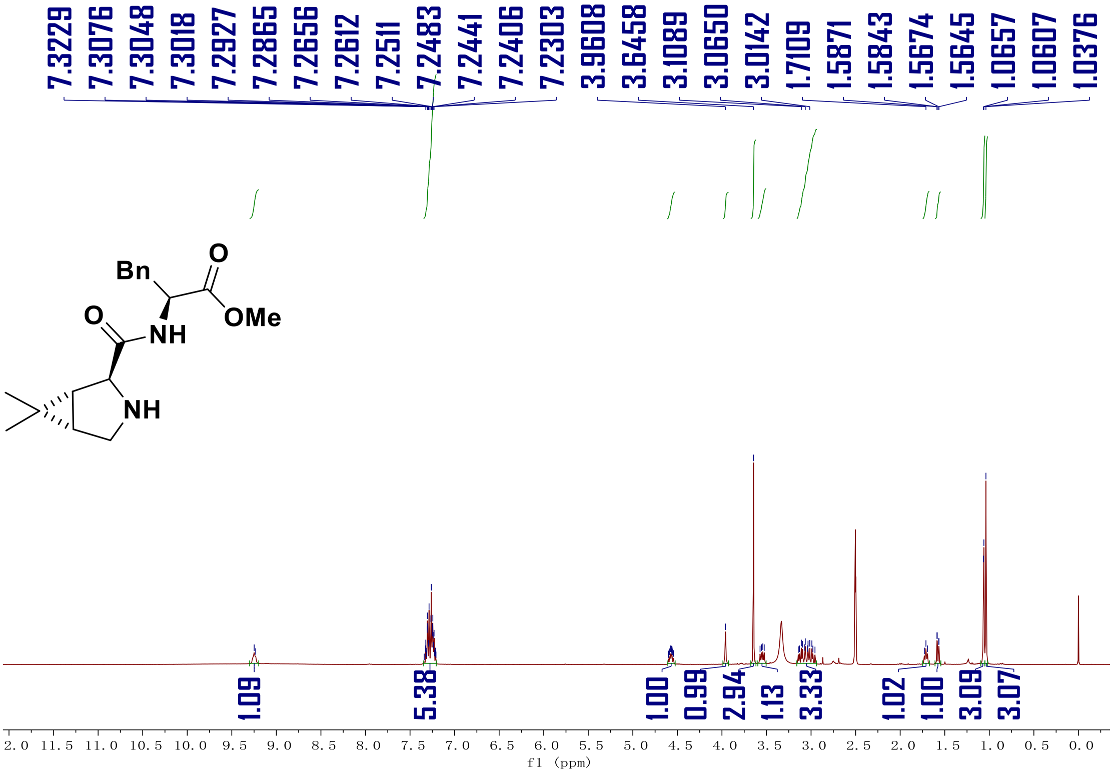

Supplementary Figure 41. ^{1}**H NMR (400 MHz, DMSO-*d*$_6$) of 17a**

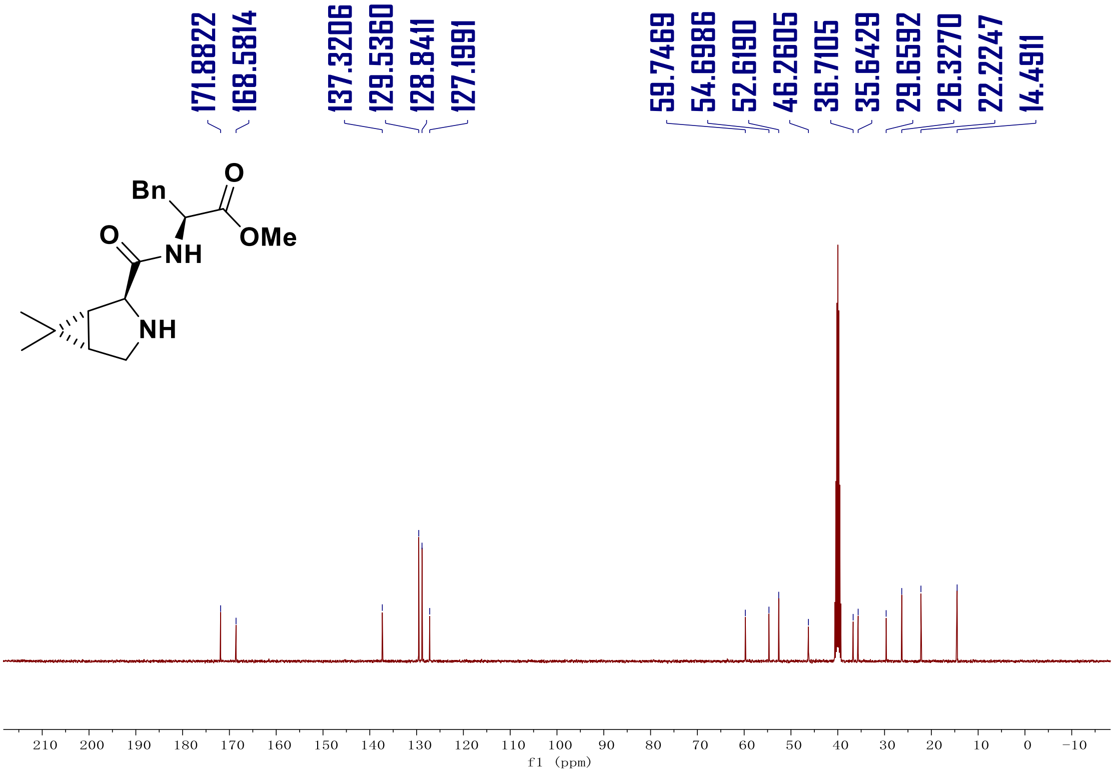

Supplementary Figure 42. ^{13}**C NMR (101 MHz, DMSO-*d*$_6$) of 17a**

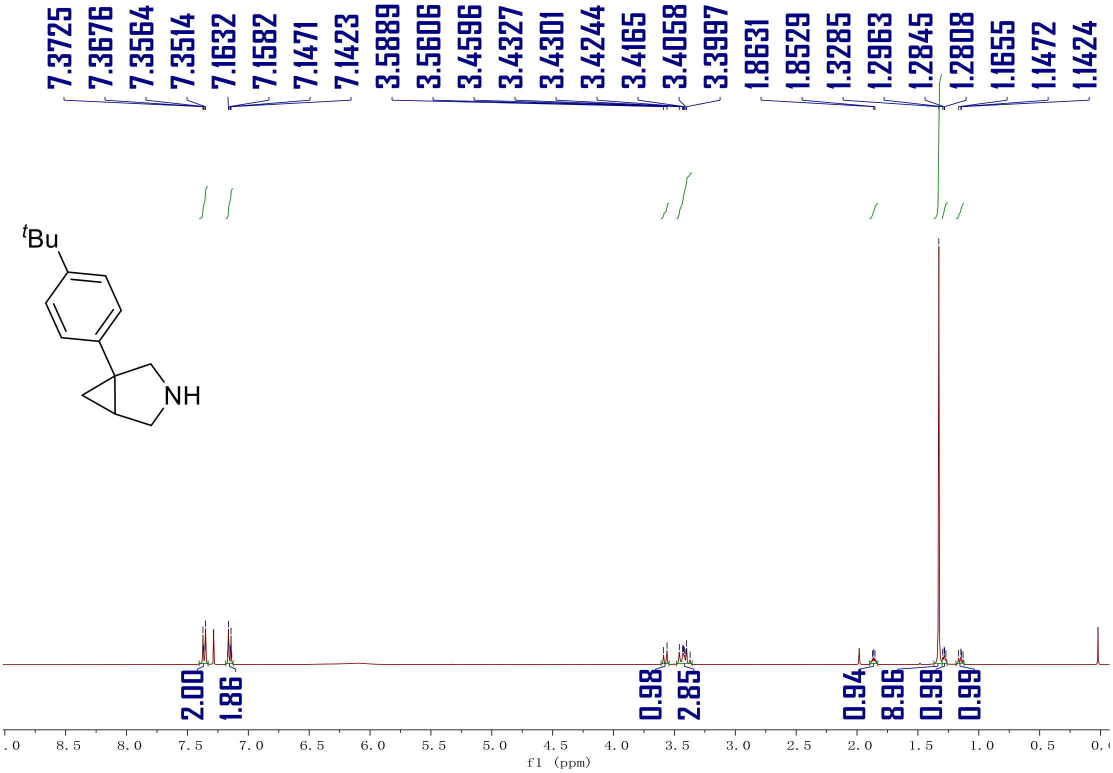

Supplementary Figure 43. ^{1}H NMR (400 MHz, $CDCl_3$) of 18a

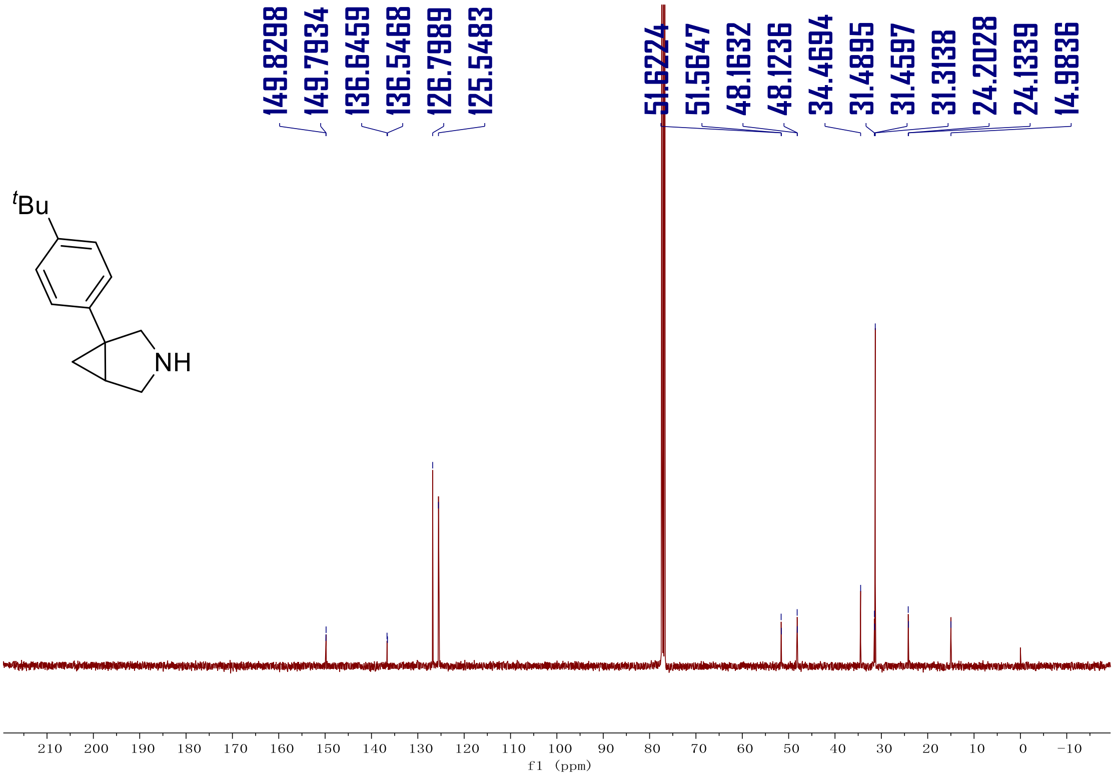

Supplementary Figure 44. ^{13}C NMR (101 MHz, $CDCl_3$) of 18a

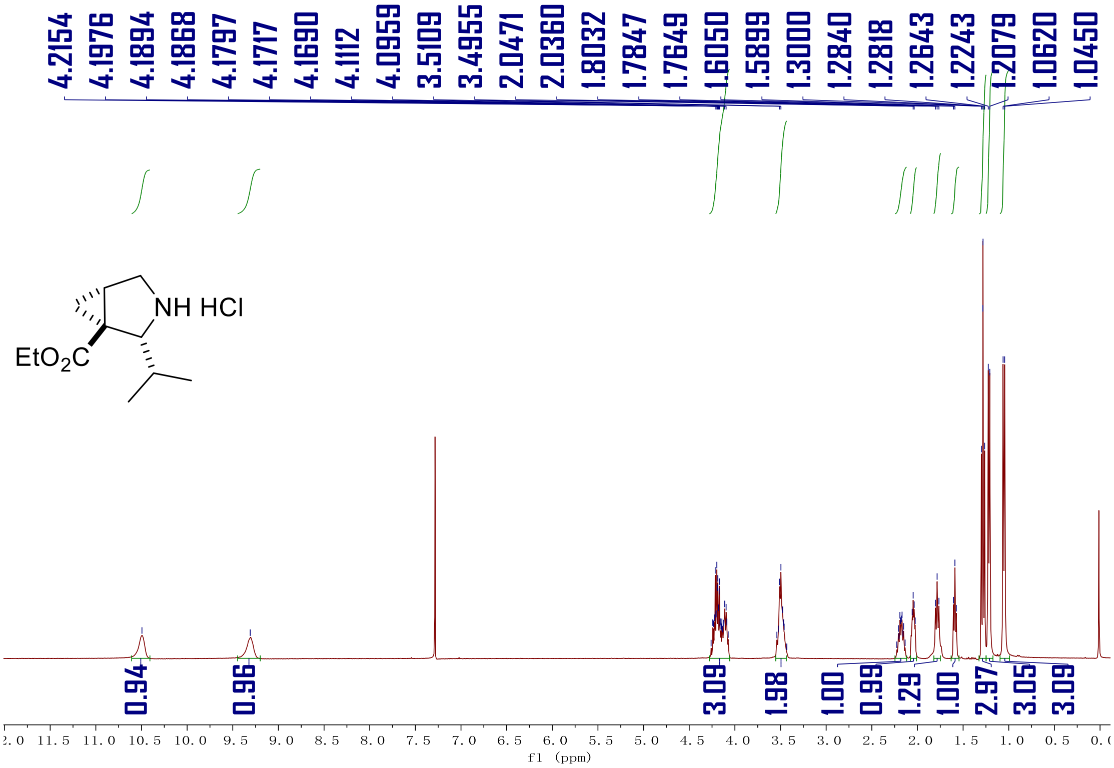

Supplementary Figure 45. ^{1}H NMR (400 MHz, $CDCl_3$) of 20a

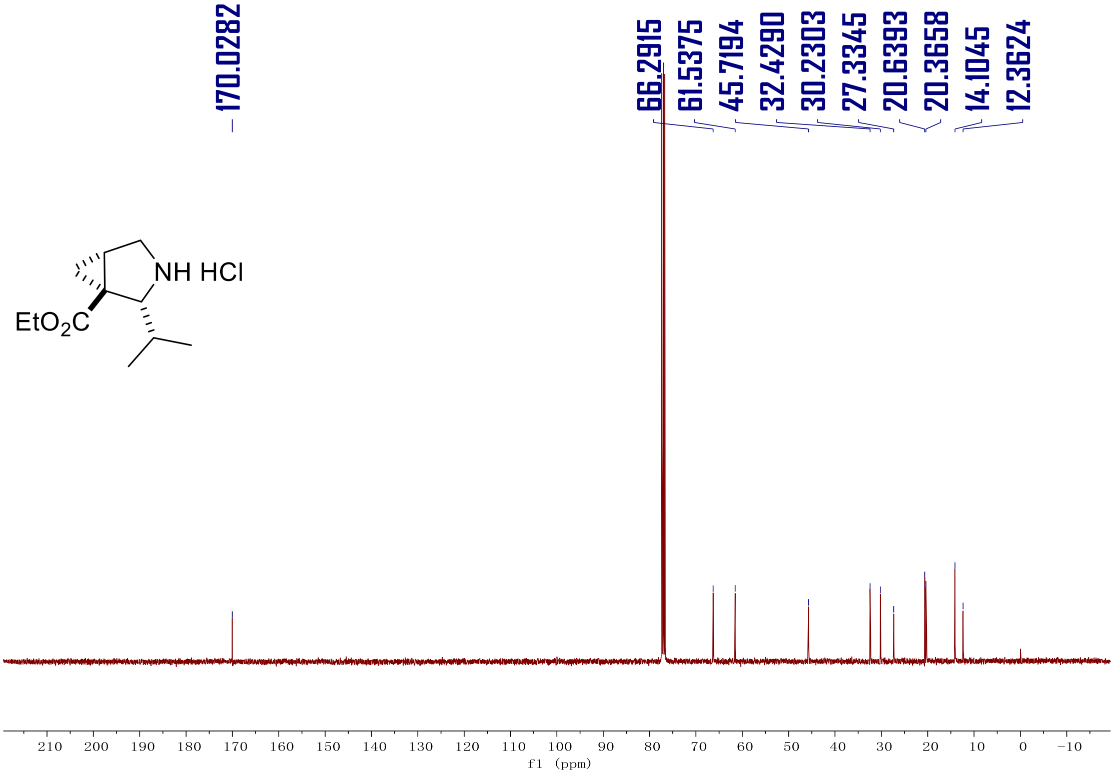

Supplementary Figure 46. ^{13}C NMR (101 MHz, $CDCl_3$) of 20a

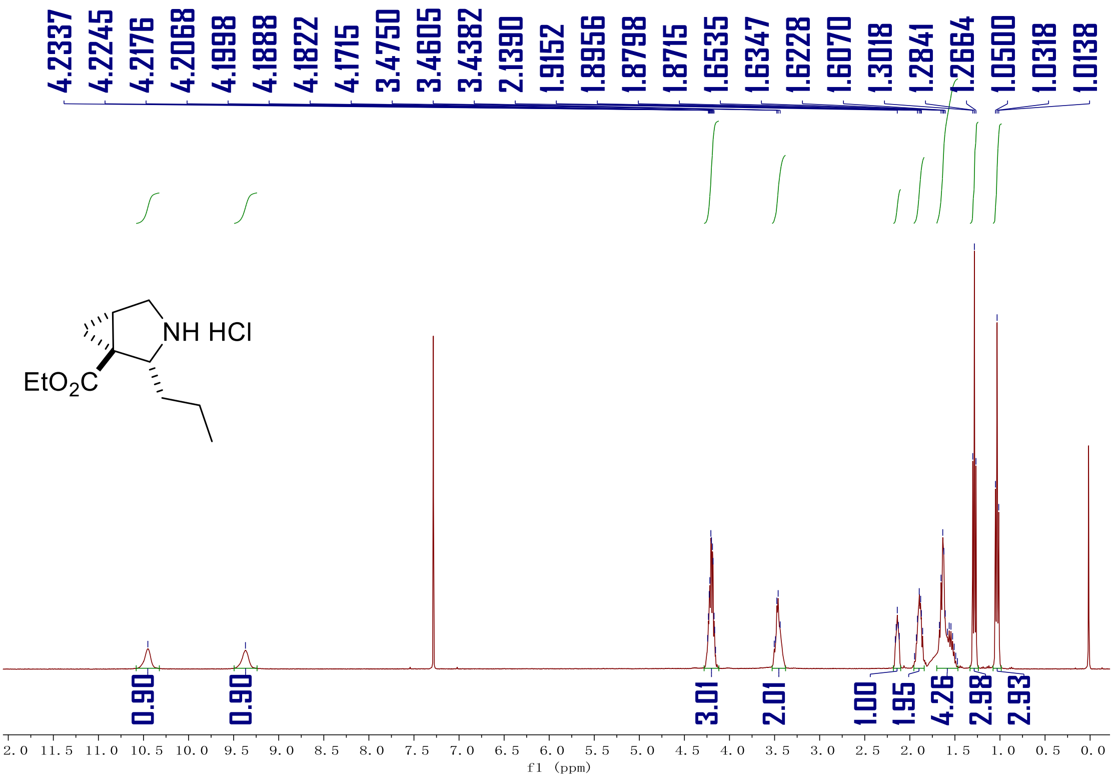

Supplementary Figure 47. ^{1}H NMR (400 MHz, $CDCl_3$) of 21a

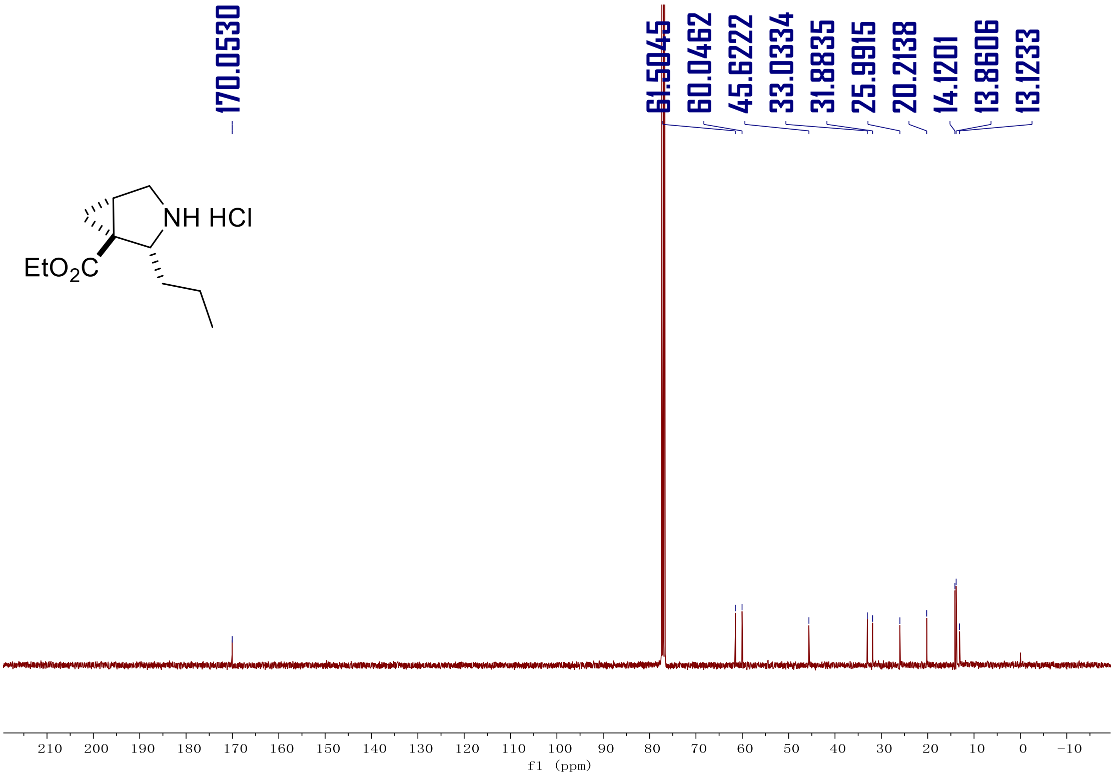

Supplementary Figure 48. ^{13}C NMR (101 MHz, $CDCl_3$) of 21a

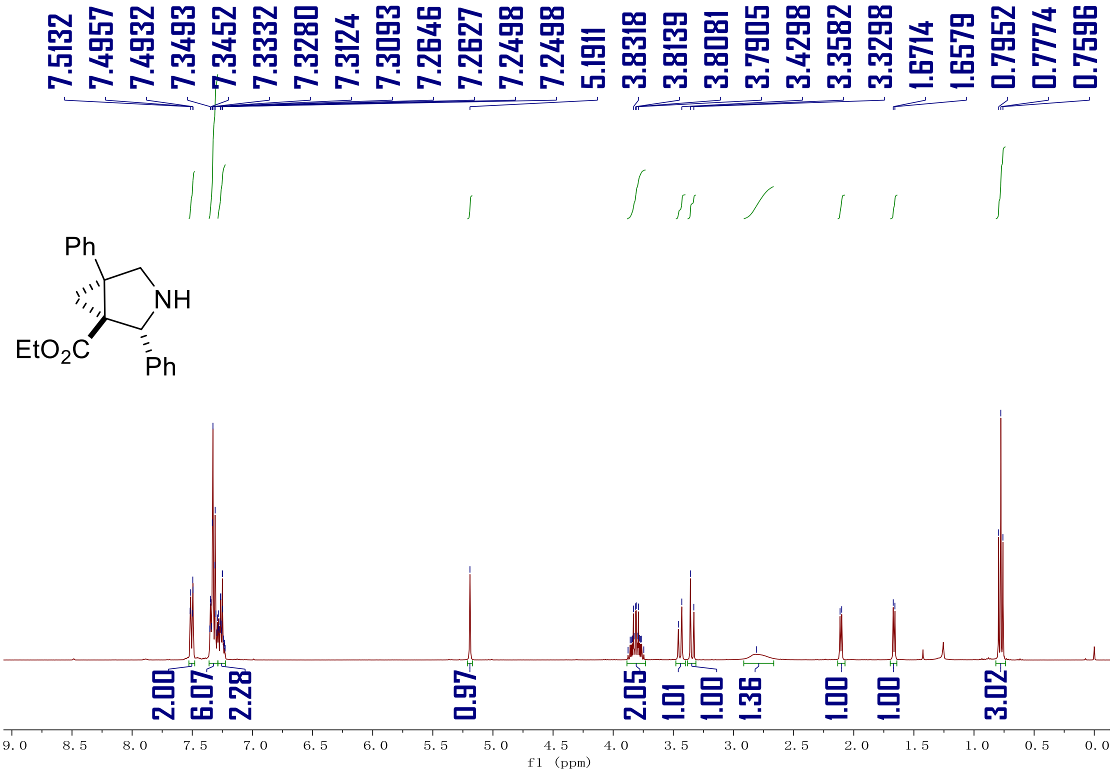

Supplementary Figure 49. ^{1}**H NMR (400 MHz, CDCl**$_{3}$**) of 22a**

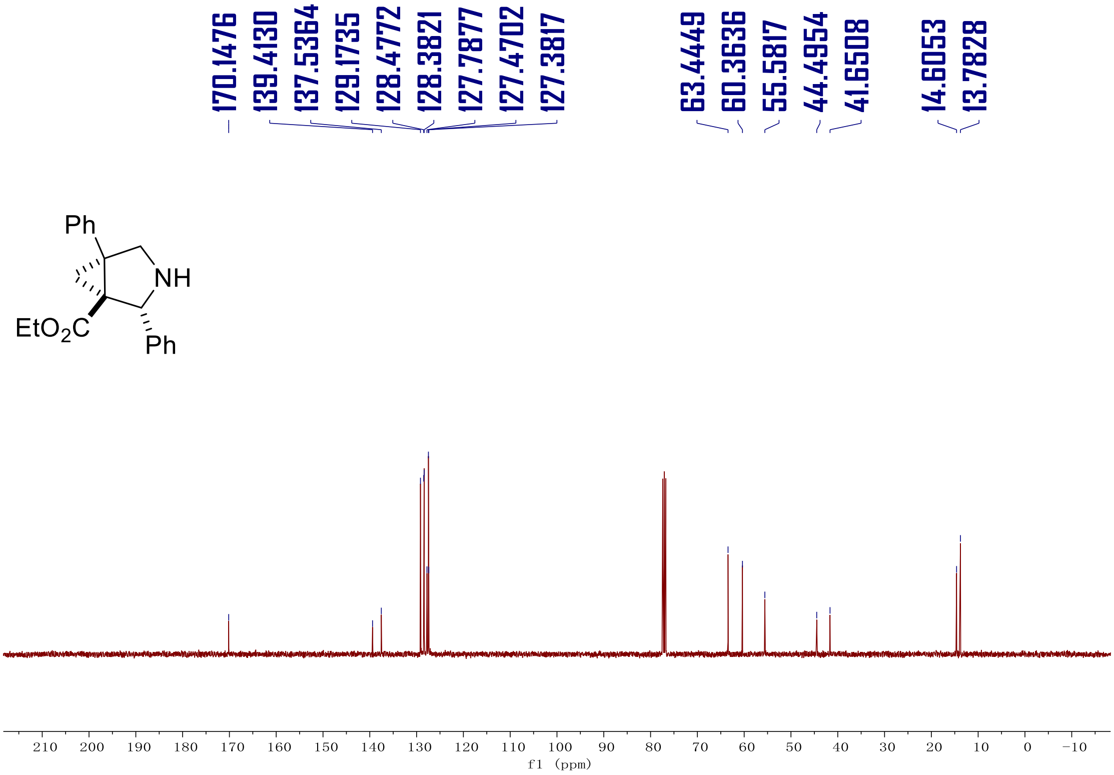

Supplementary Figure 50. ^{13}**C NMR (101 MHz, CDCl**$_{3}$**) of 22a**

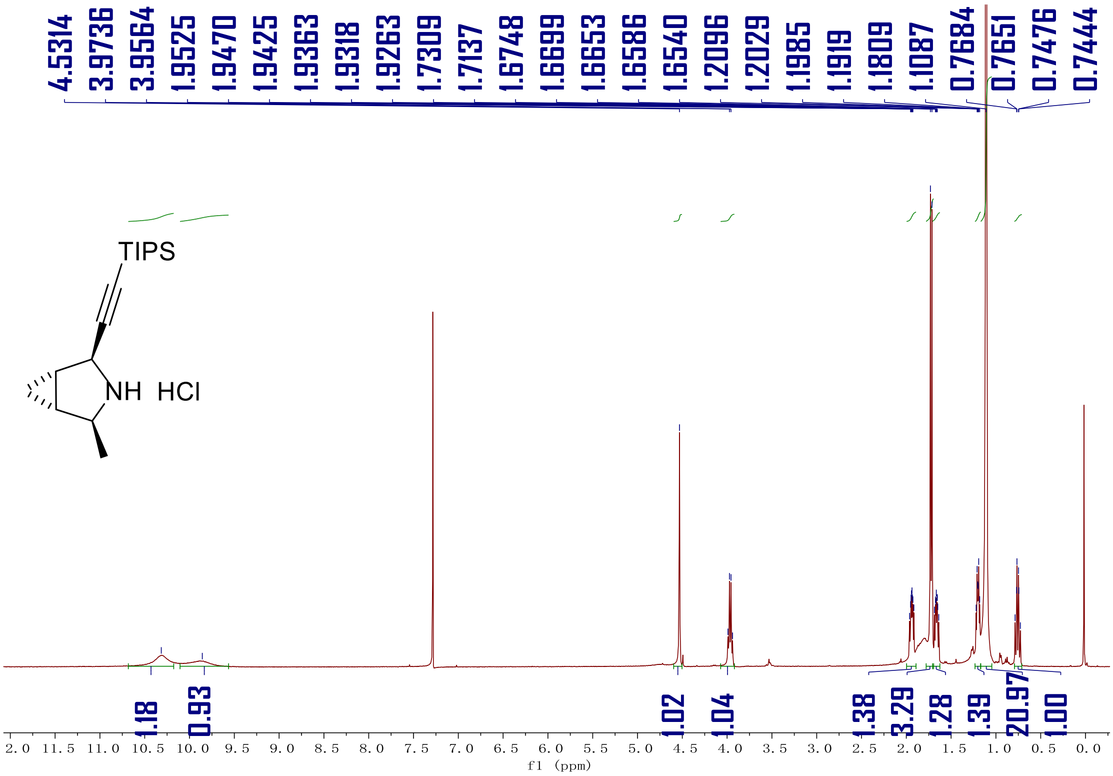

Supplementary Figure 51. ^{1}H NMR (400 MHz, $CDCl_3$) of 24a

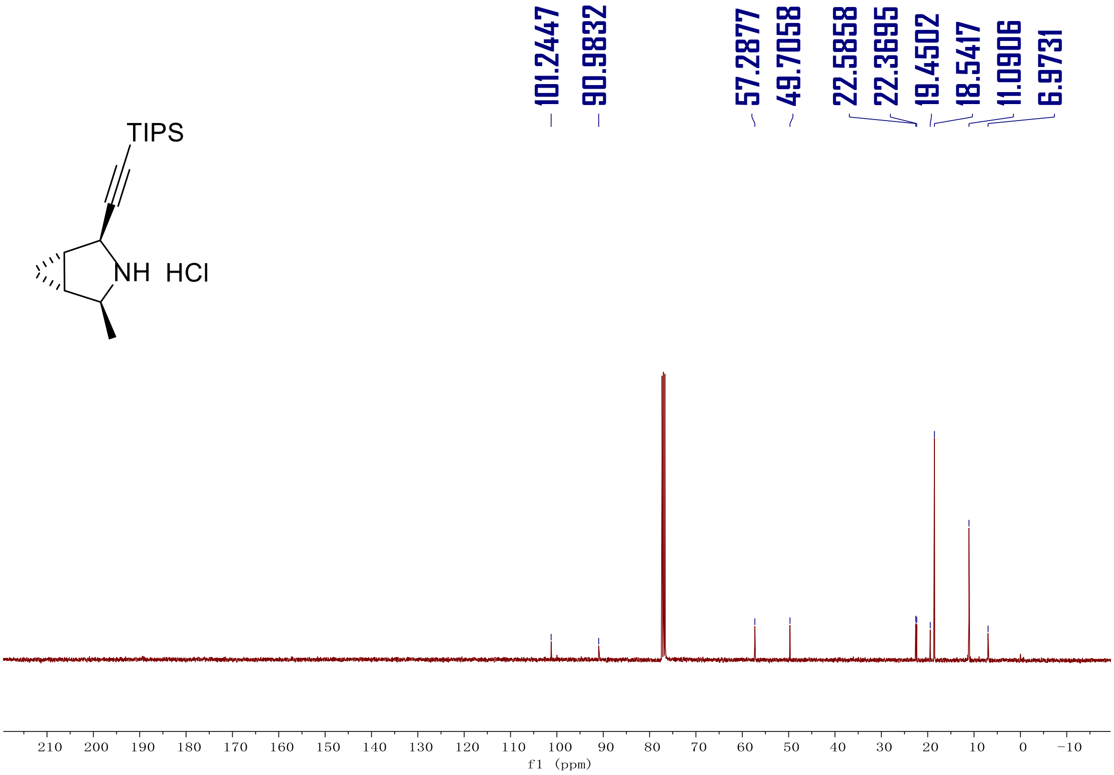

Supplementary Figure 52. ^{13}C NMR (101 MHz, $CDCl_3$) of 24a

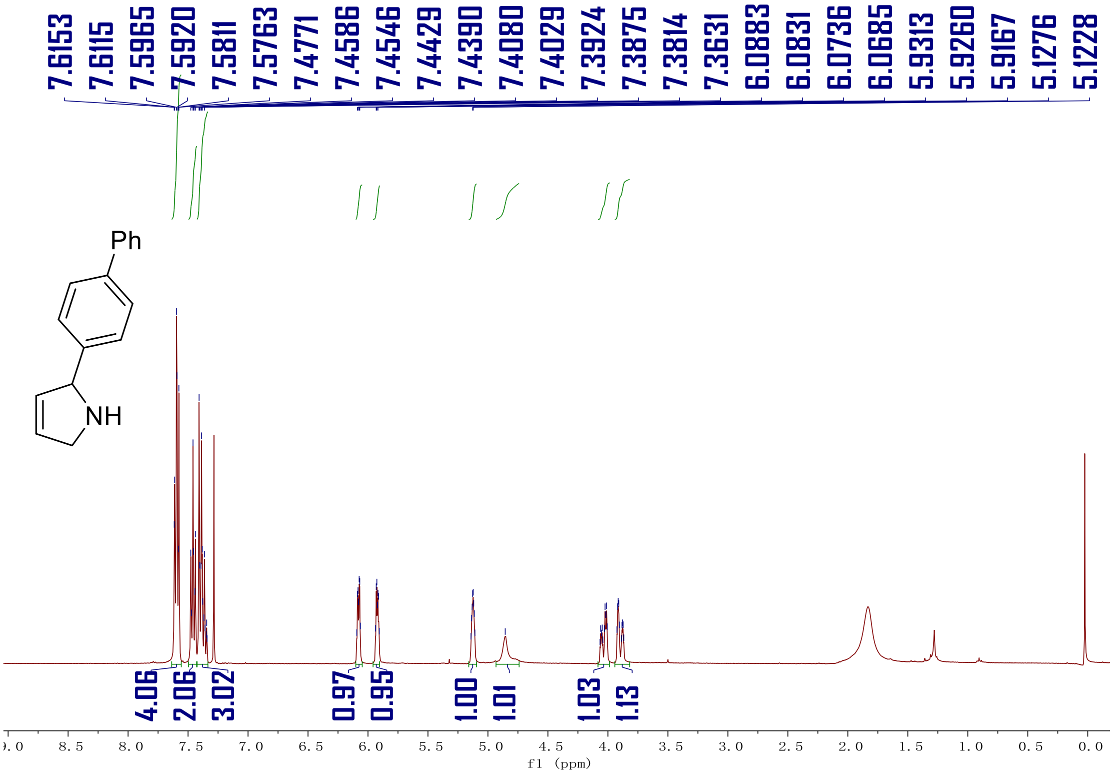

Supplementary Figure 53. ^{1}H NMR (400 MHz, $CDCl_3$) of 25a

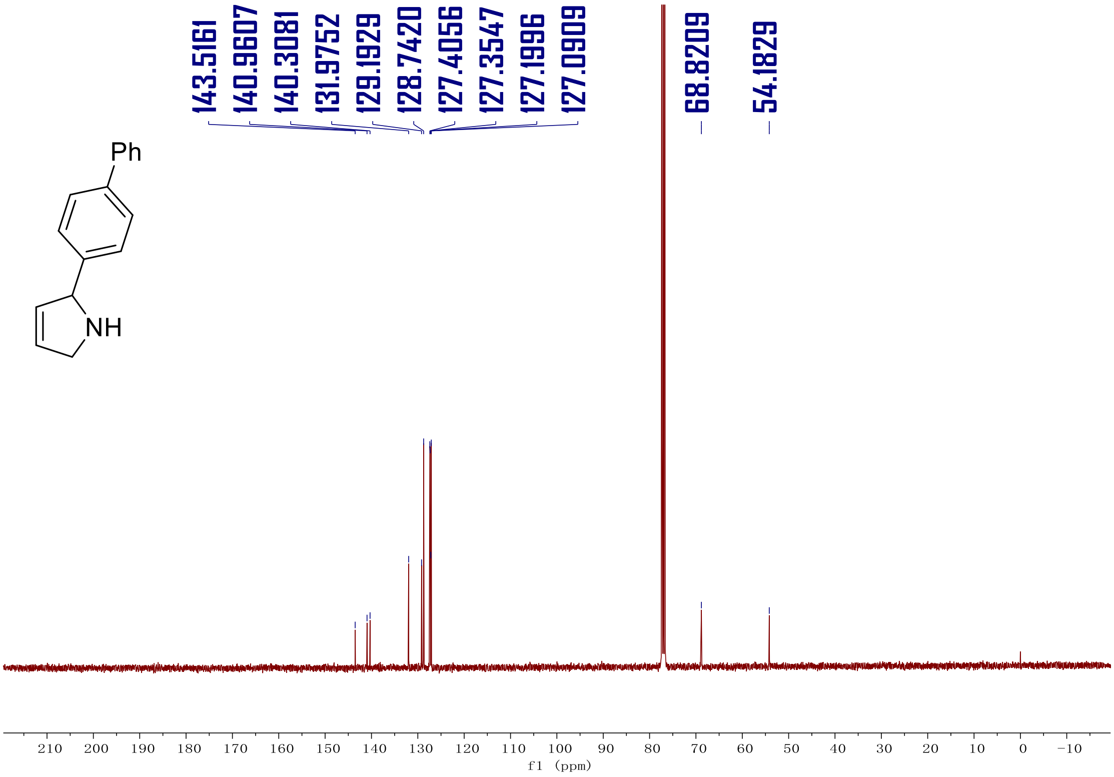

Supplementary Figure 54. ^{13}C NMR (101 MHz, $CDCl_3$) of 25a

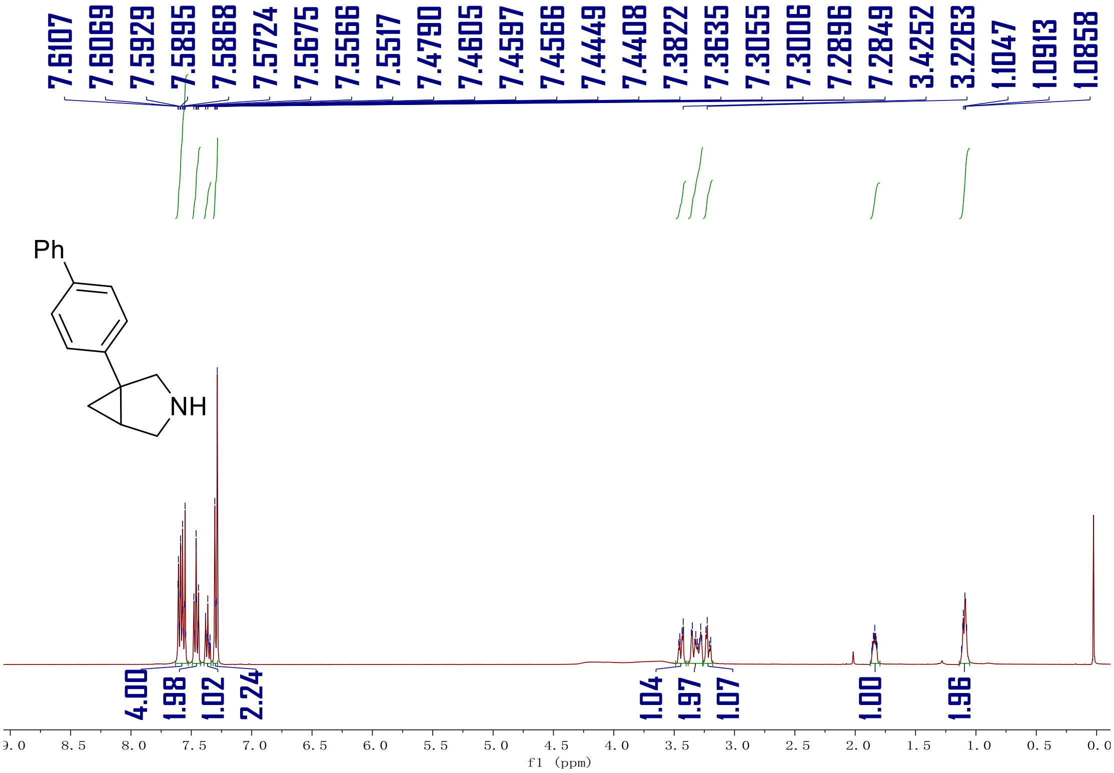

Supplementary Figure 55. ^{1}**H NMR (400 MHz, CDCl$_3$) of 26a**

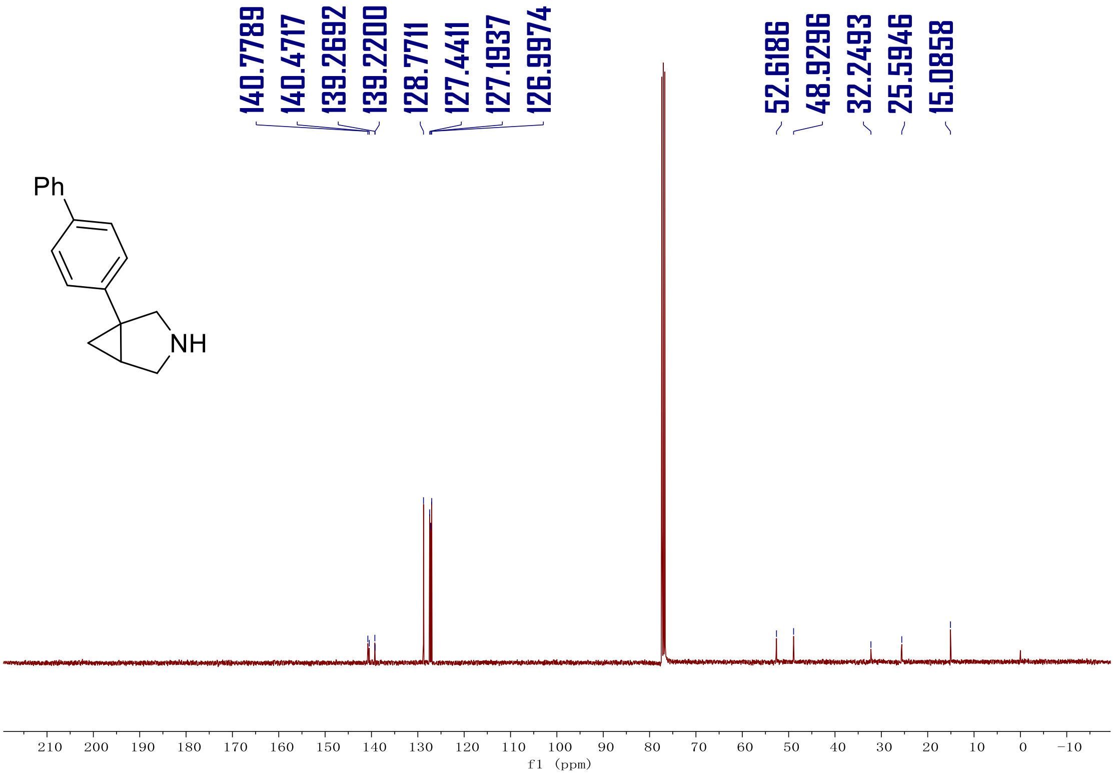

Supplementary Figure 56. ^{13}**C NMR (101 MHz, CDCl$_3$) of 26a**

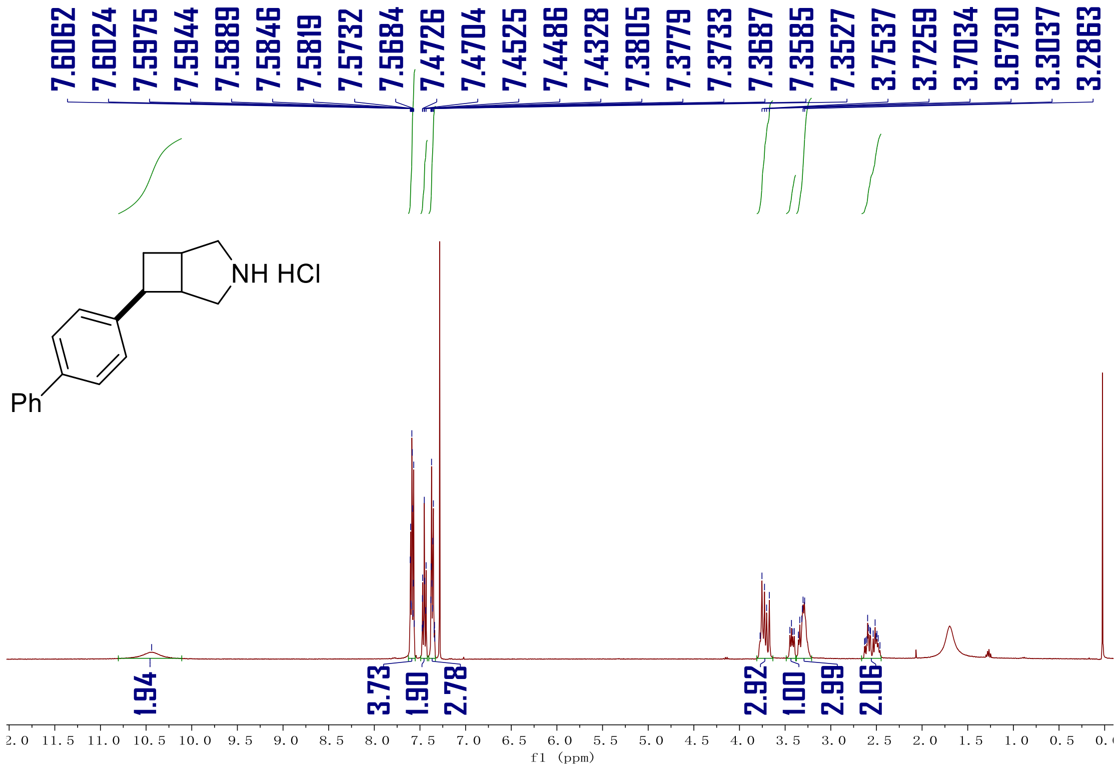

Supplementary Figure 57. ^{1}H NMR (400 MHz, CDCl$_3$) of 27a

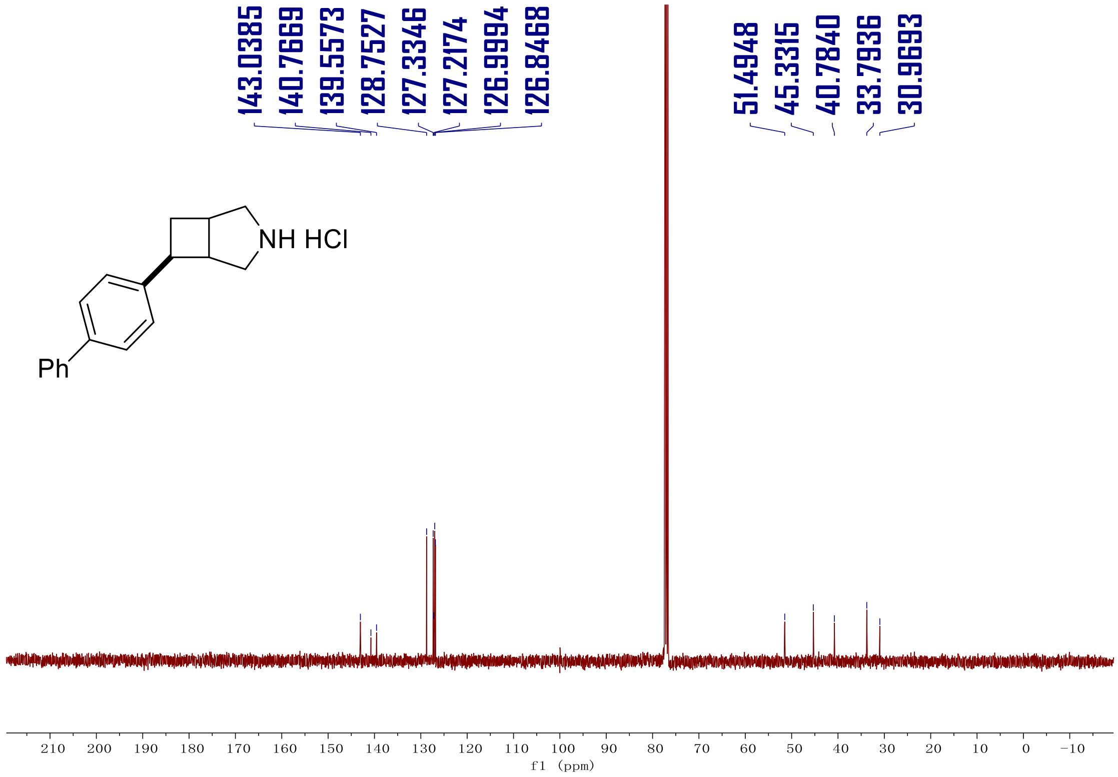

Supplementary Figure 58. ^{13}C NMR (101 MHz, CDCl$_3$) of 27a

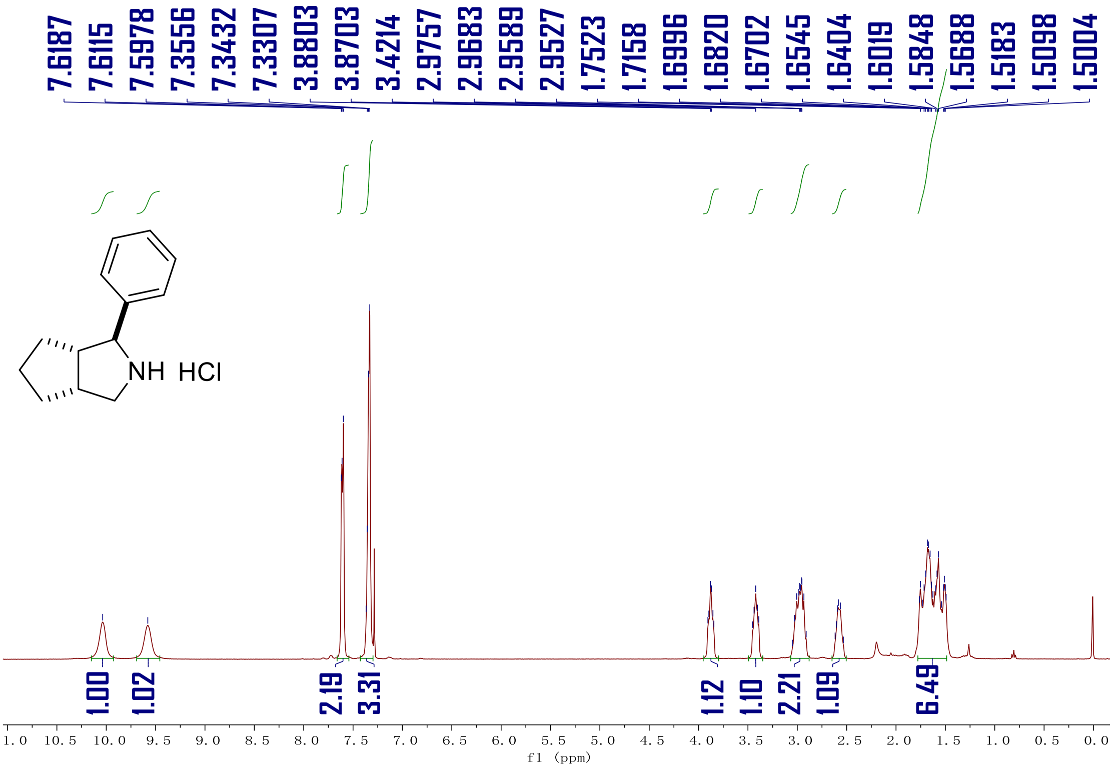

Supplementary Figure 59. ^{1}**H NMR (400 MHz,** $CDCl_3$**) of 28a**

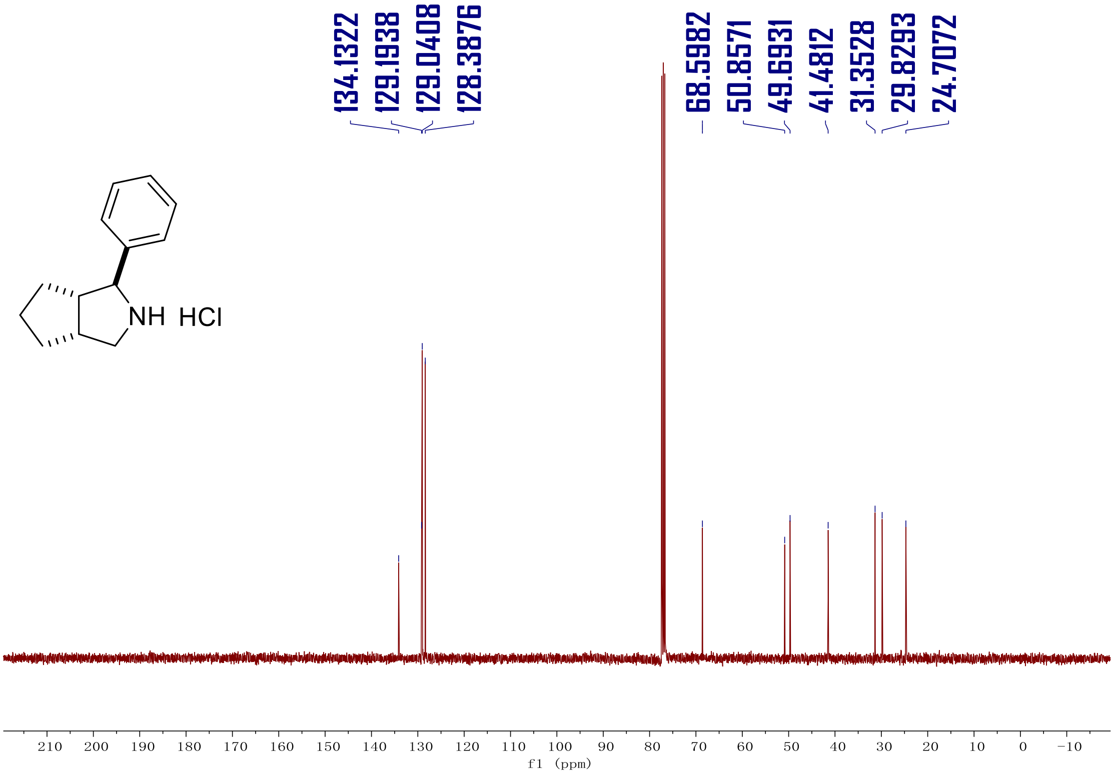

Supplementary Figure 60. ^{13}**C NMR (101 MHz,** $CDCl_3$**) of 28a**

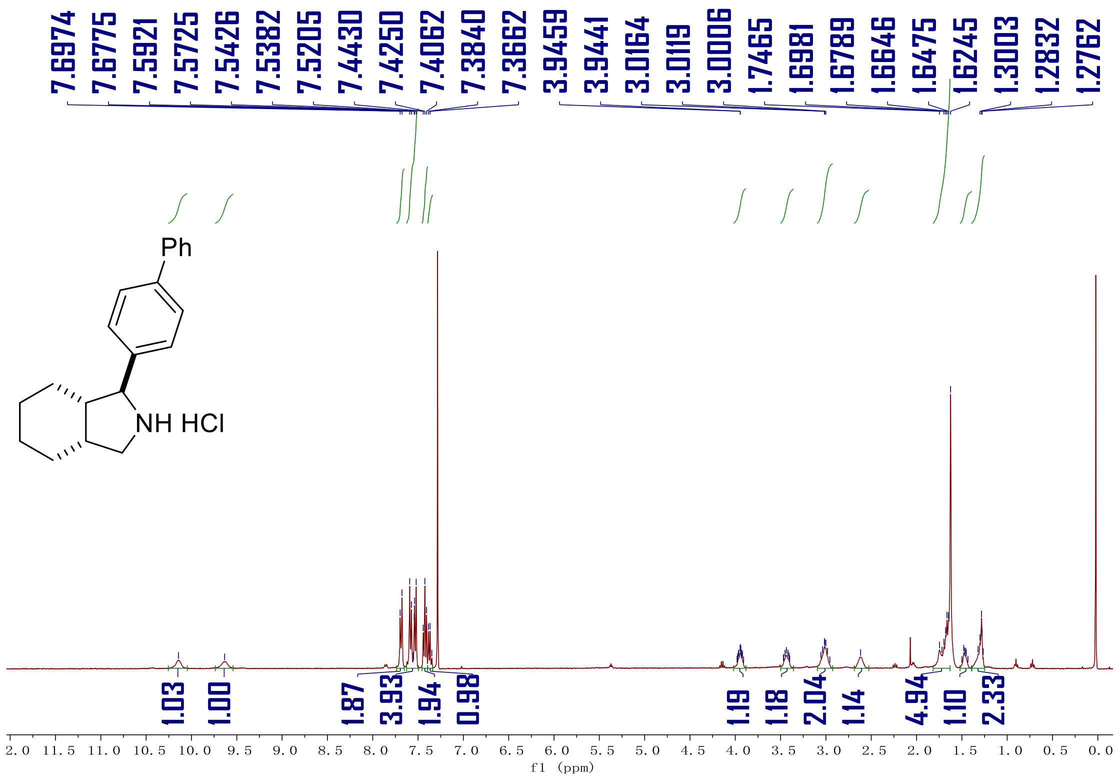

Supplementary Figure 61. ^{1}H NMR (400 MHz, CDCl$_3$) of 29a

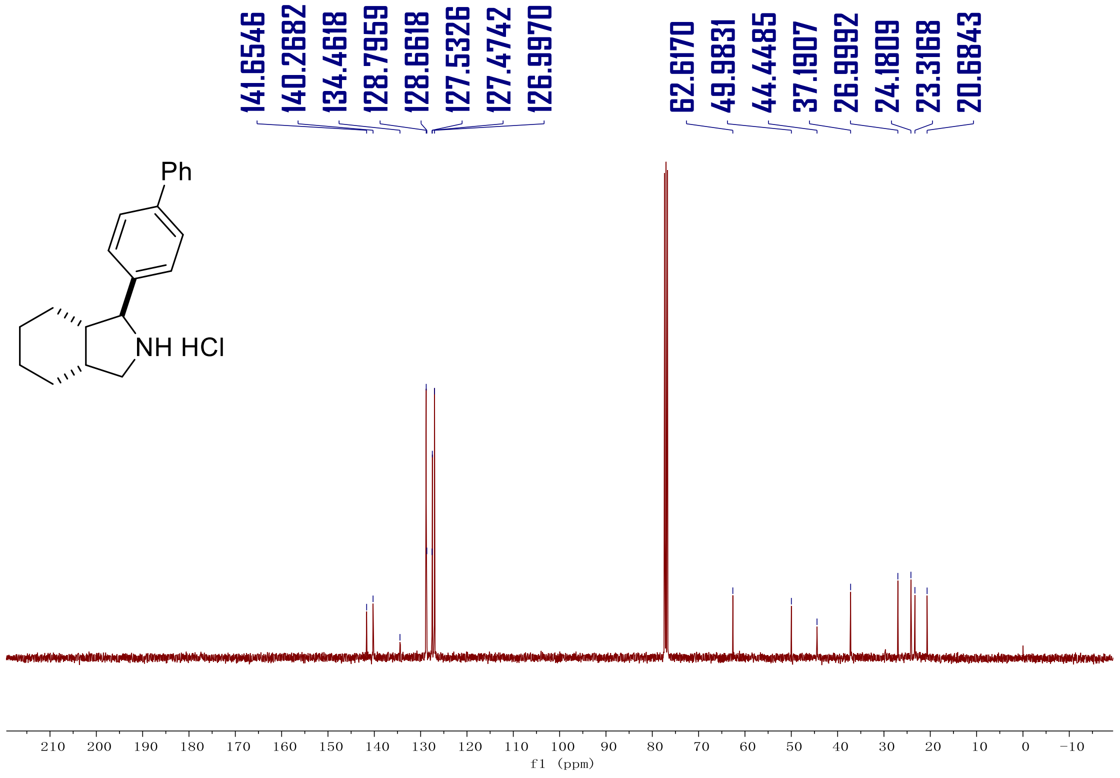

Supplementary Figure 62. ^{13}C NMR (101 MHz, CDCl$_3$) of 29a

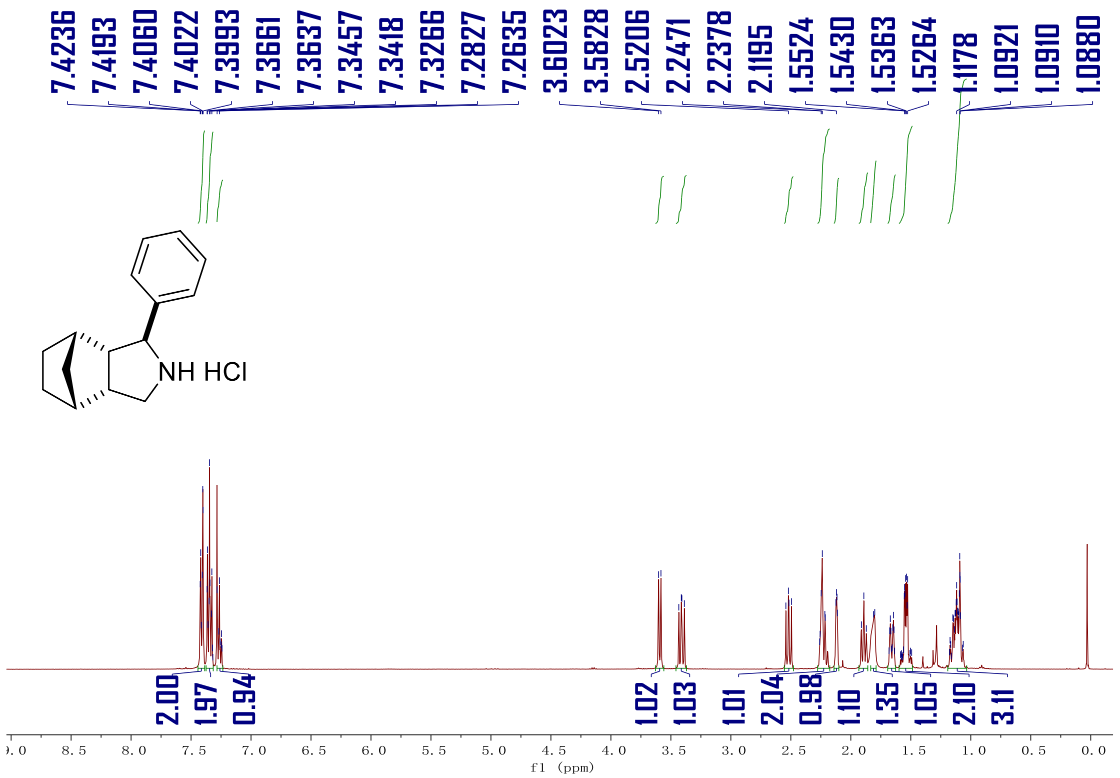

Supplementary Figure 63. ^{1}H NMR (400 MHz, $CDCl_3$) of 30a

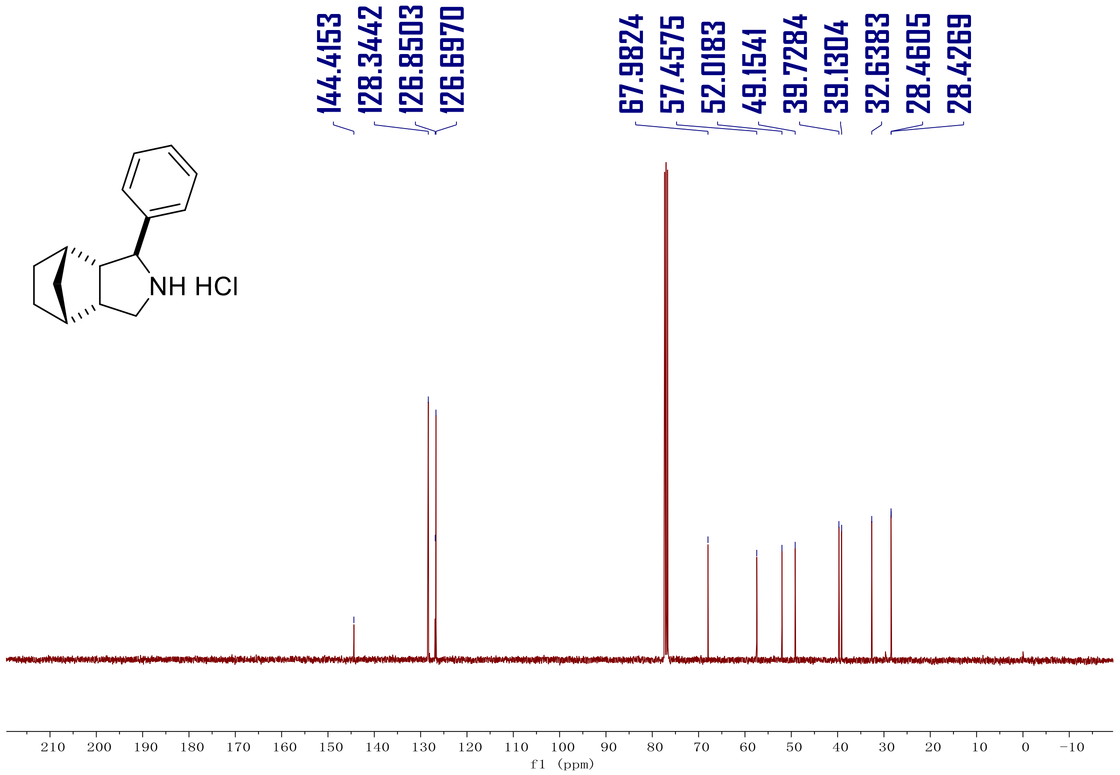

Supplementary Figure 64. ^{13}C NMR (101 MHz, $CDCl_3$) of 30a

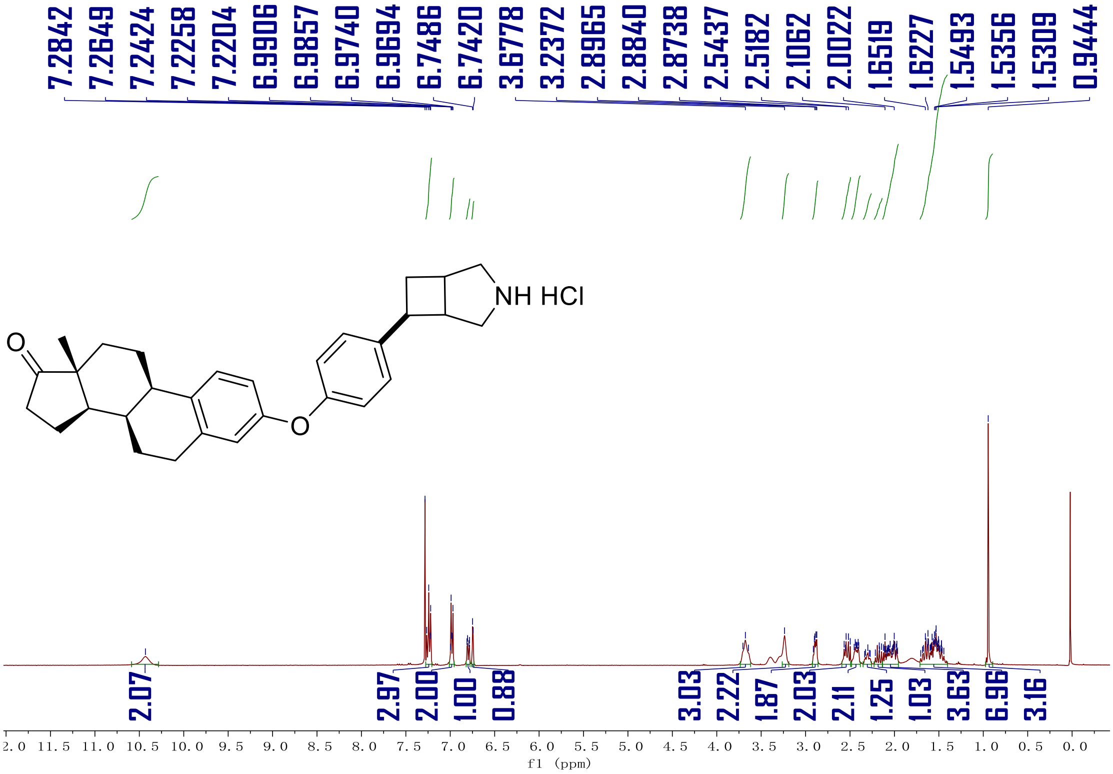

Supplementary Figure 65. ^{1}H NMR (400 MHz, CDCl$_3$) of 31a

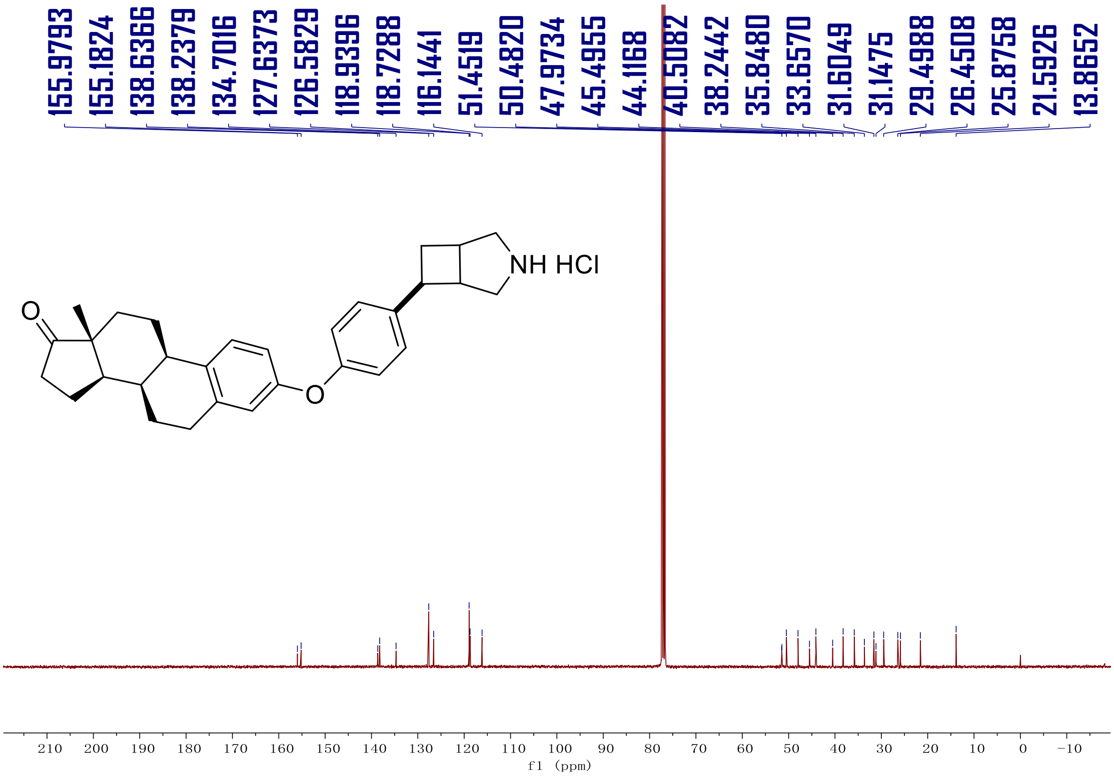

Supplementary Figure 66. ^{13}C NMR (101 MHz, CDCl$_3$) of 31a

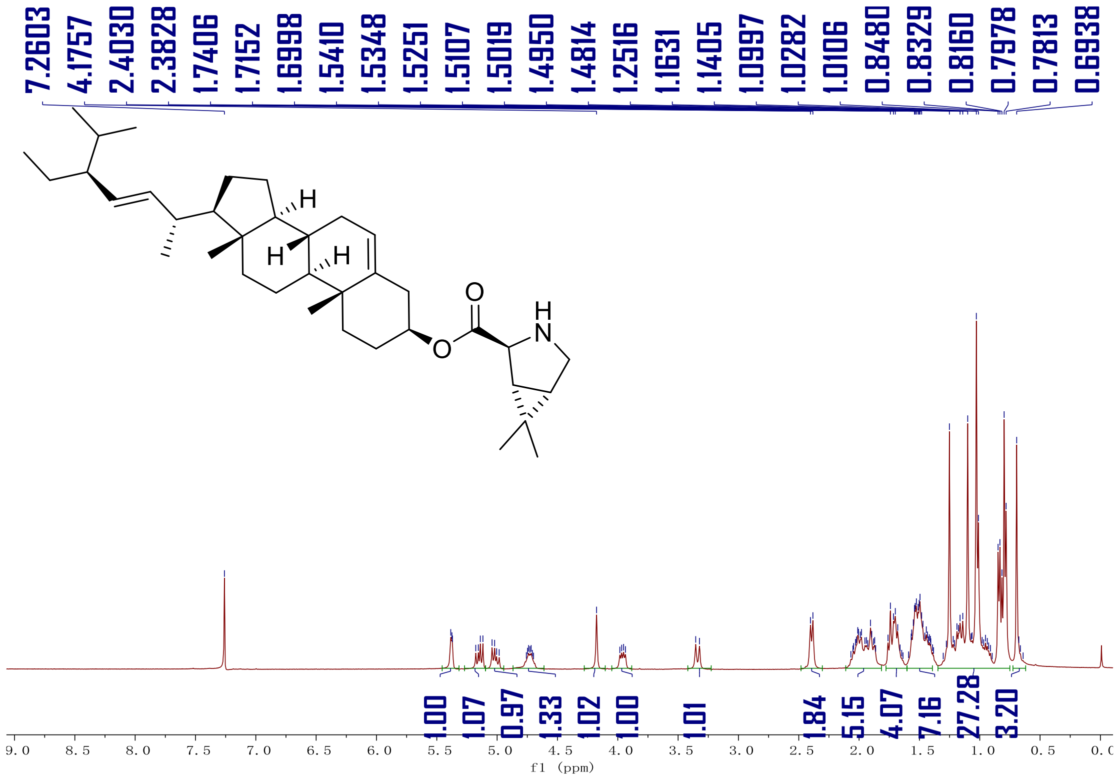

Supplementary Figure 67. ^{1}H NMR (400 MHz, $CDCl_3$) of 32a

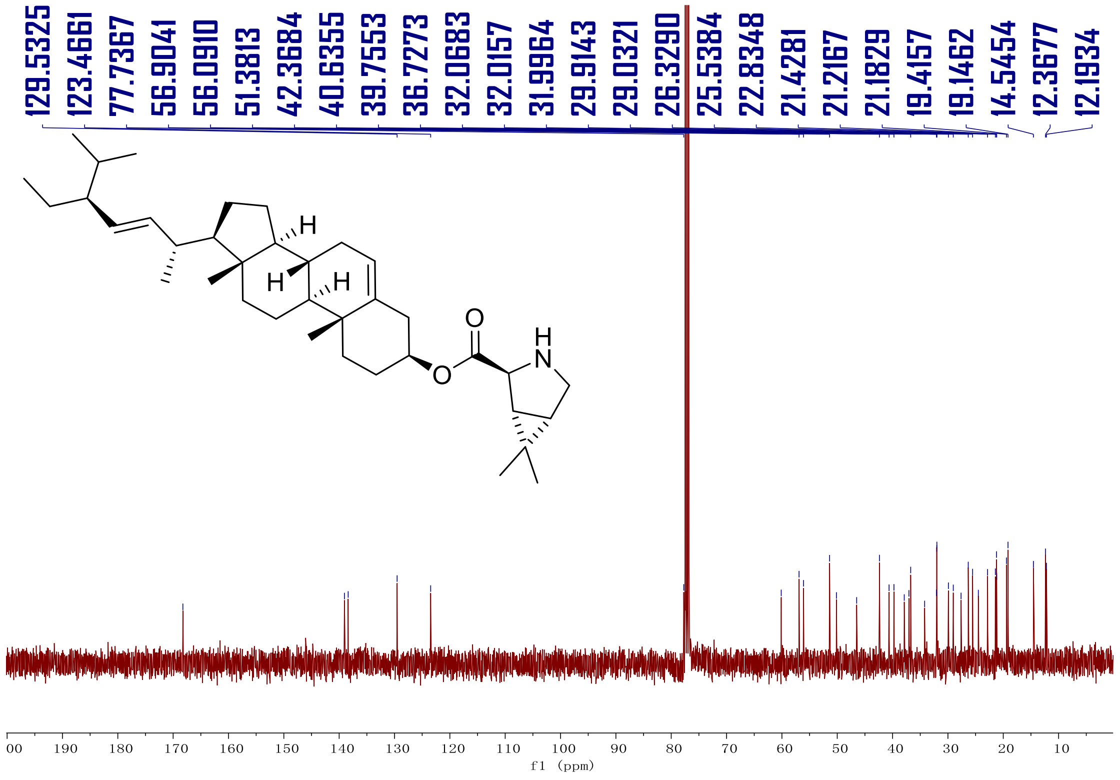

Supplementary Figure 68. ^{13}C NMR (101 MHz, $CDCl_3$) of 32a

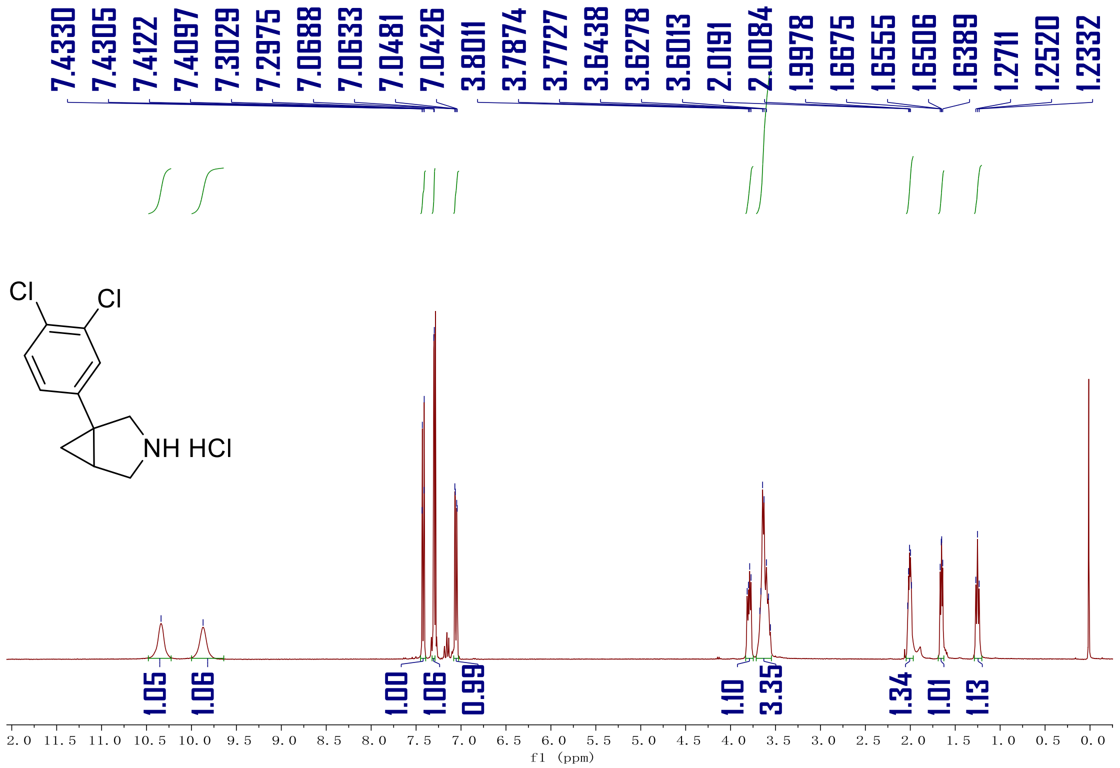

Supplementary Figure 69. ^{1}H NMR (400 MHz, $CDCl_3$) of 34a

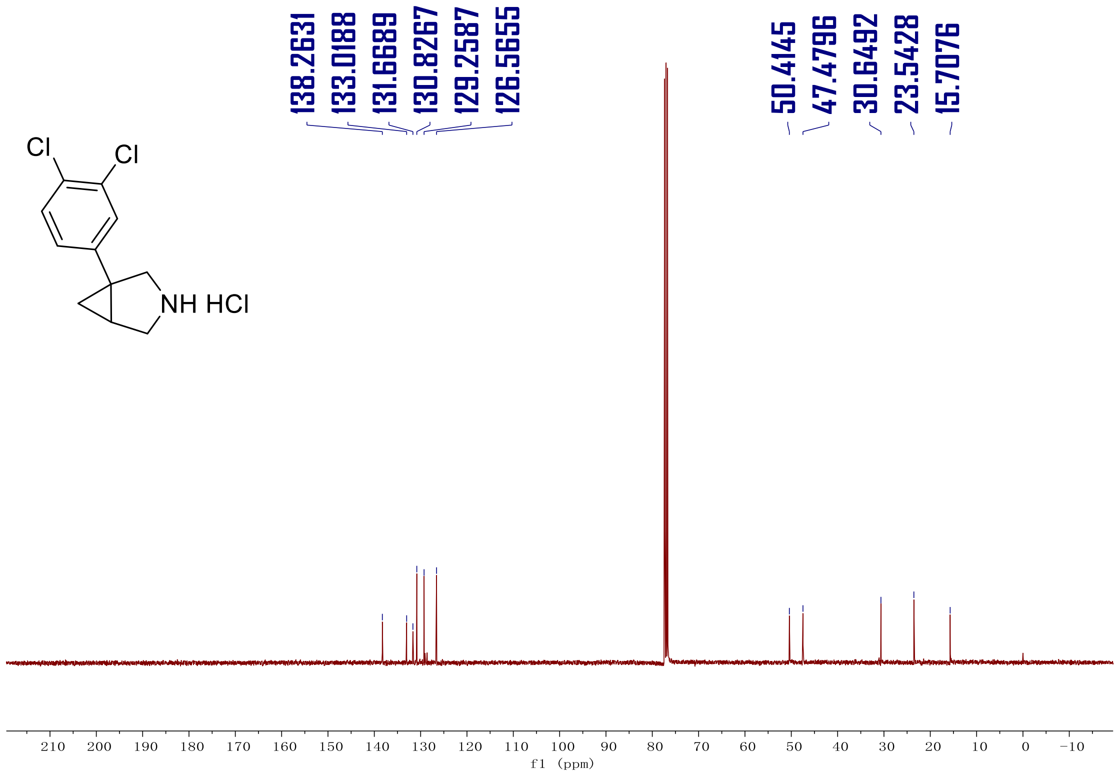

Supplementary Figure 70. ^{13}C NMR (101 MHz, $CDCl_3$) of 34a

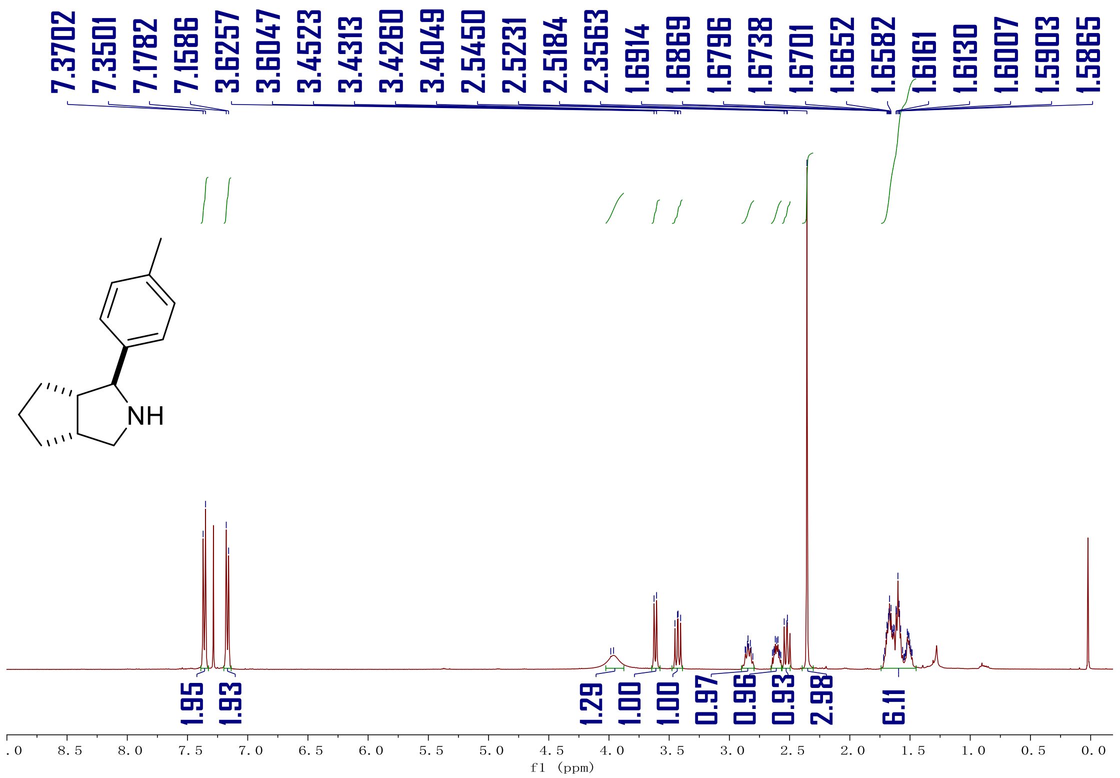

Supplementary Figure 71. ^{1}H NMR (400 MHz, $CDCl_3$) of 36a

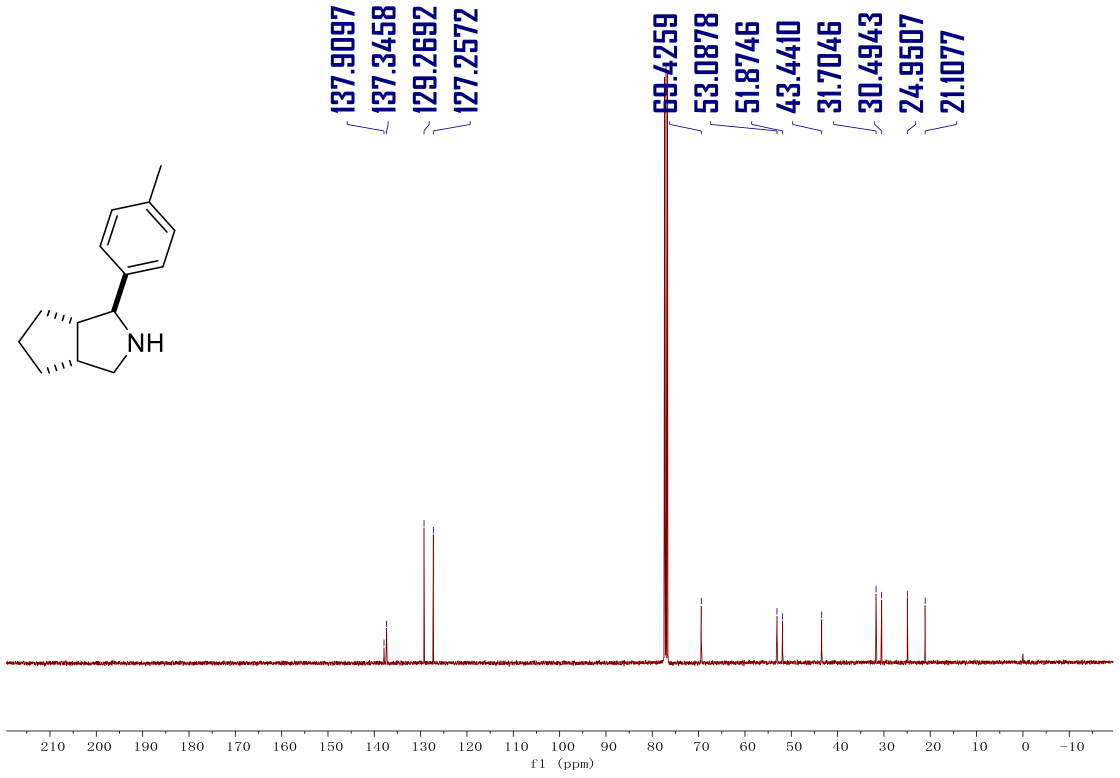

Supplementary Figure 72. ^{13}C NMR (101 MHz, $CDCl_3$) of 36a

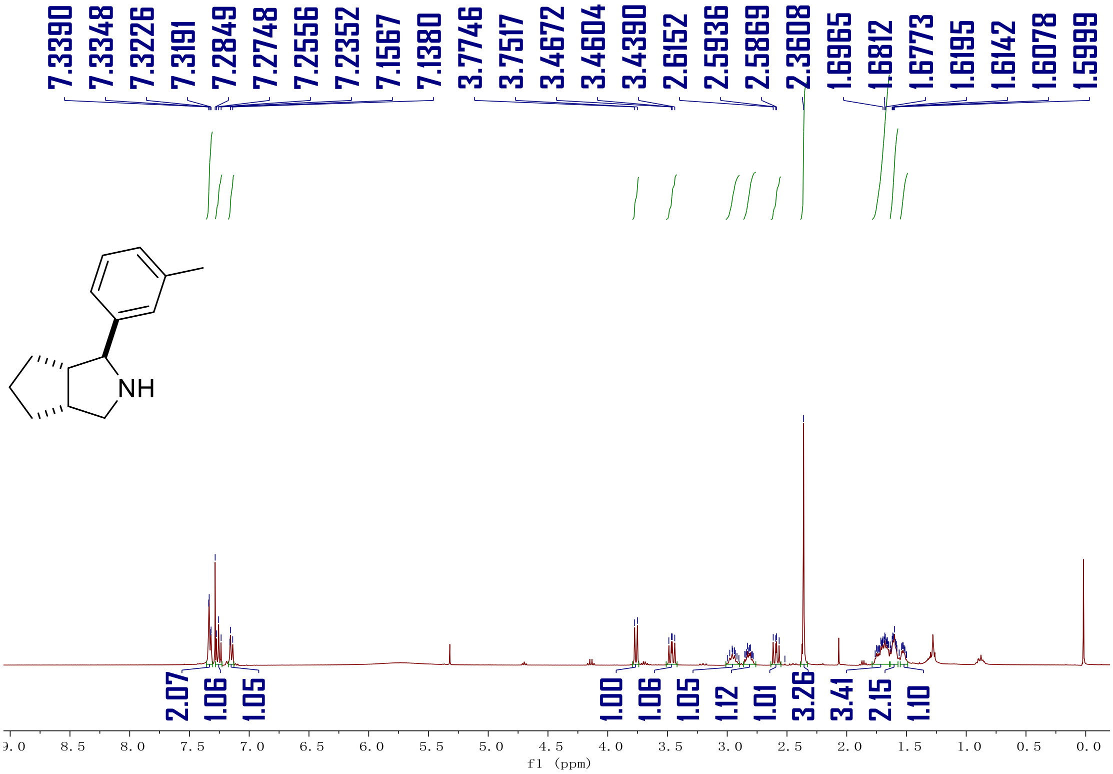

Supplementary Figure 73. ^{1}H NMR (400 MHz, CDCl$_3$) of 37a

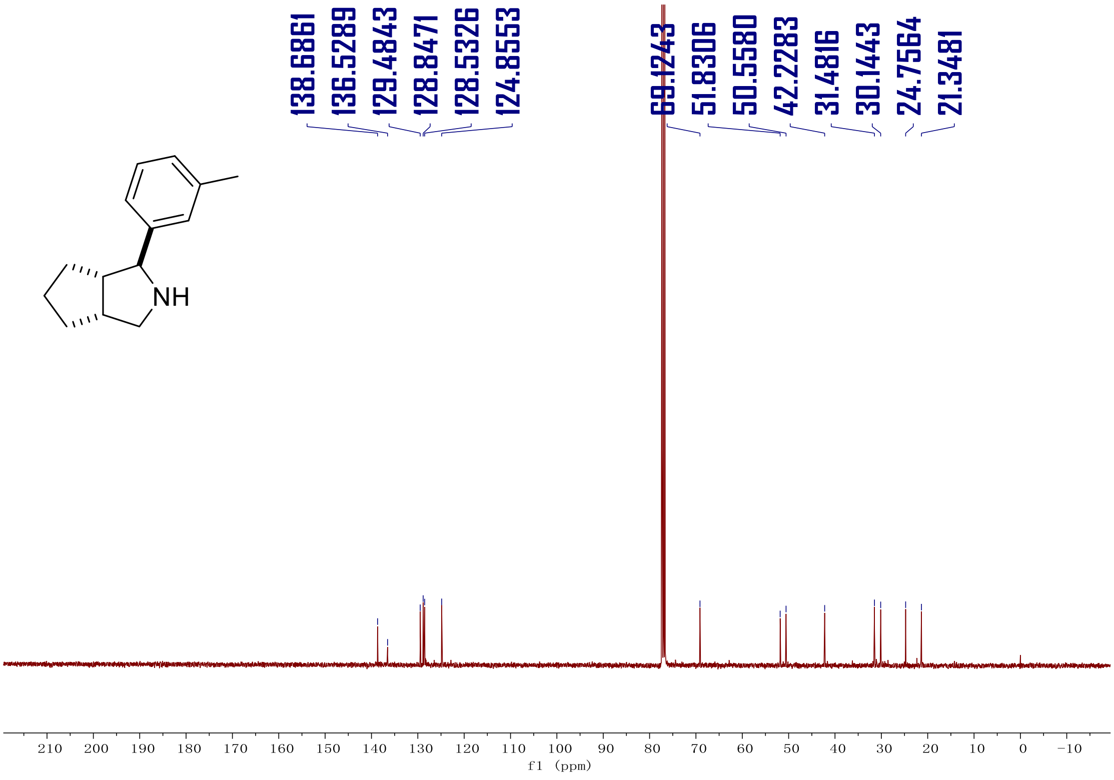

Supplementary Figure 74. ^{13}C NMR (101 MHz, CDCl$_3$) of 37a

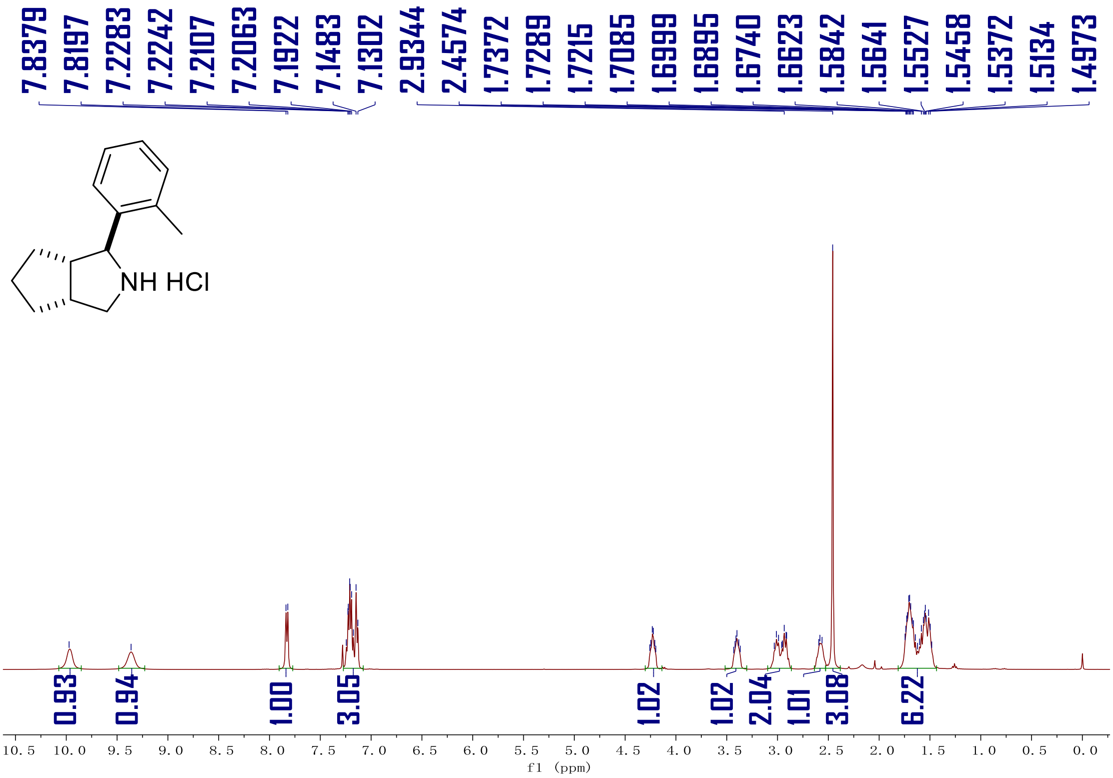

Supplementary Figure 75. ^{1}H NMR (400 MHz, $CDCl_3$) of 38a

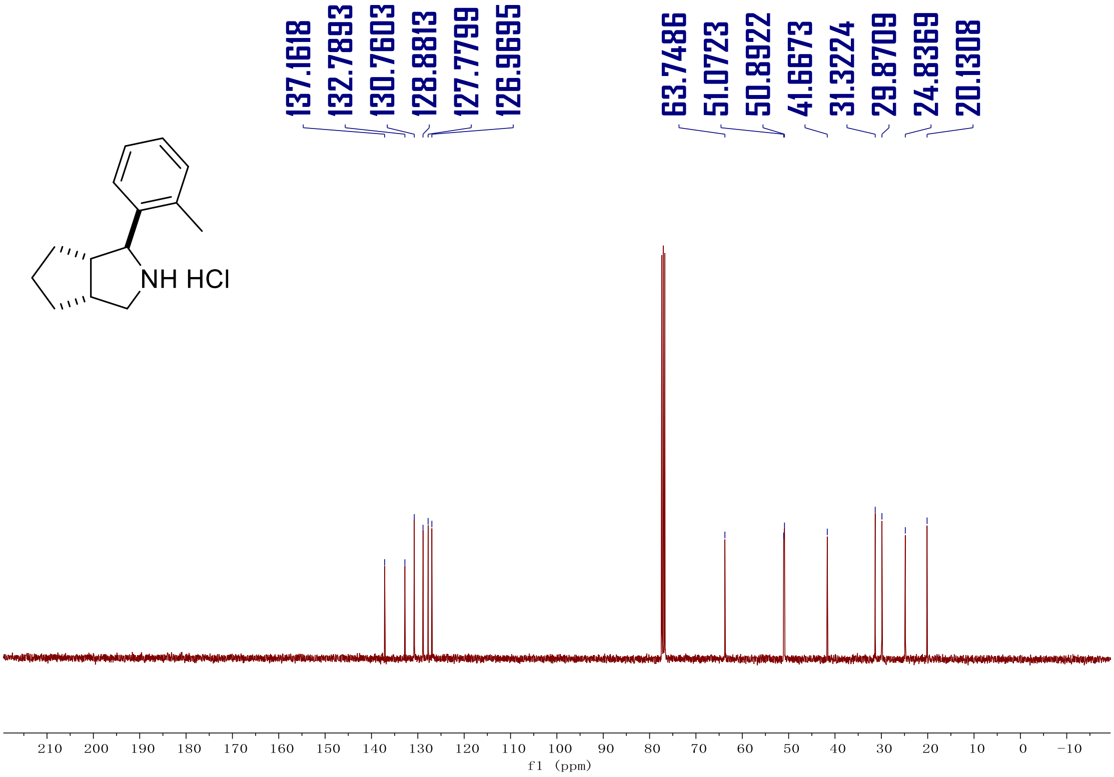

Supplementary Figure 76. ^{13}C NMR (101 MHz, $CDCl_3$) of 38a

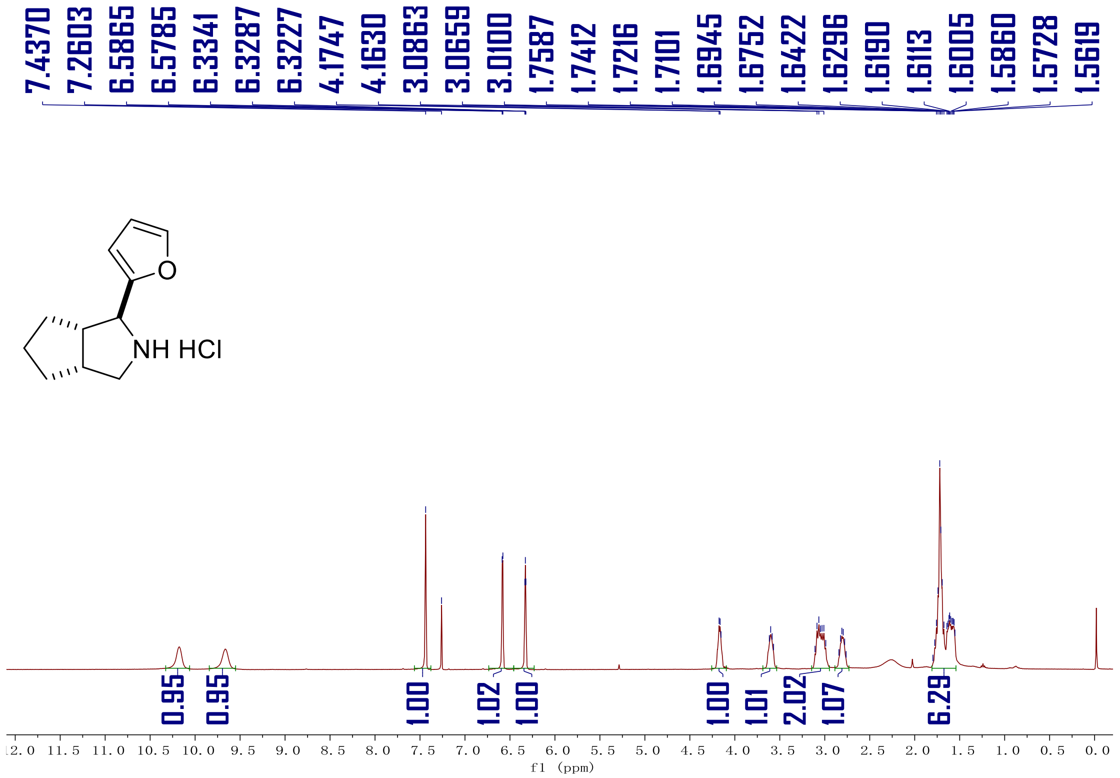

Supplementary Figure 77. ^{1}H NMR (400 MHz, $CDCl_3$) of 39a

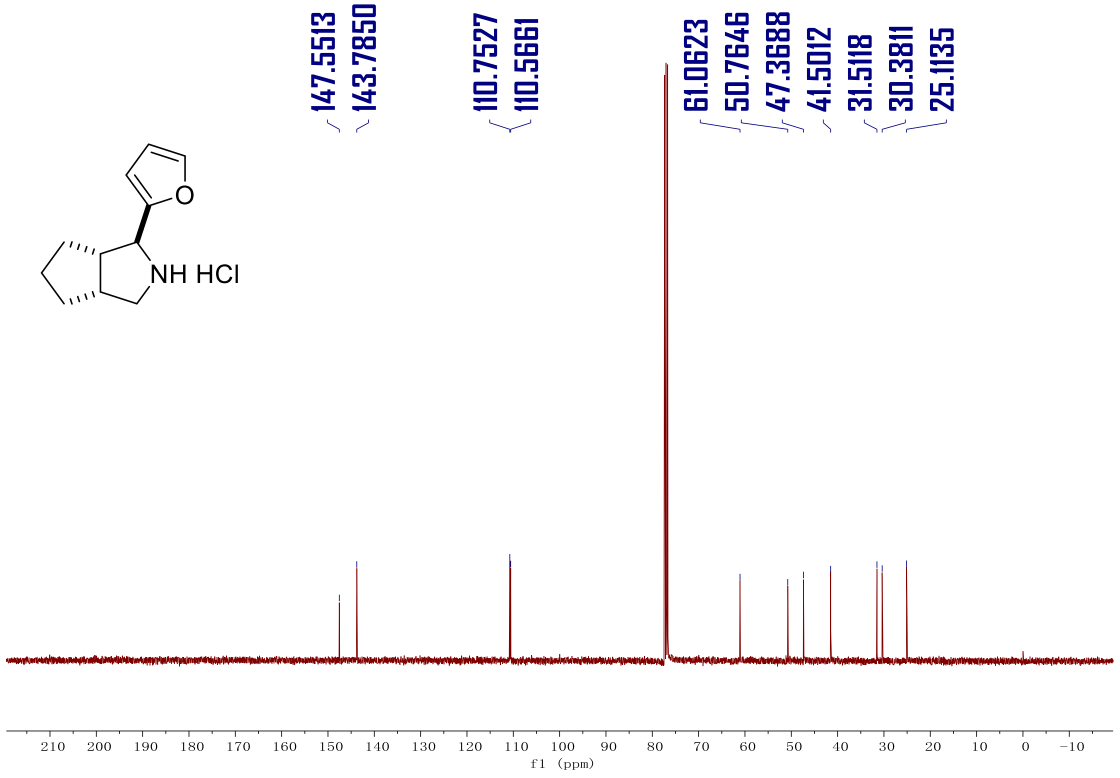

Supplementary Figure 78. ^{13}C NMR (101 MHz, $CDCl_3$) of 39a

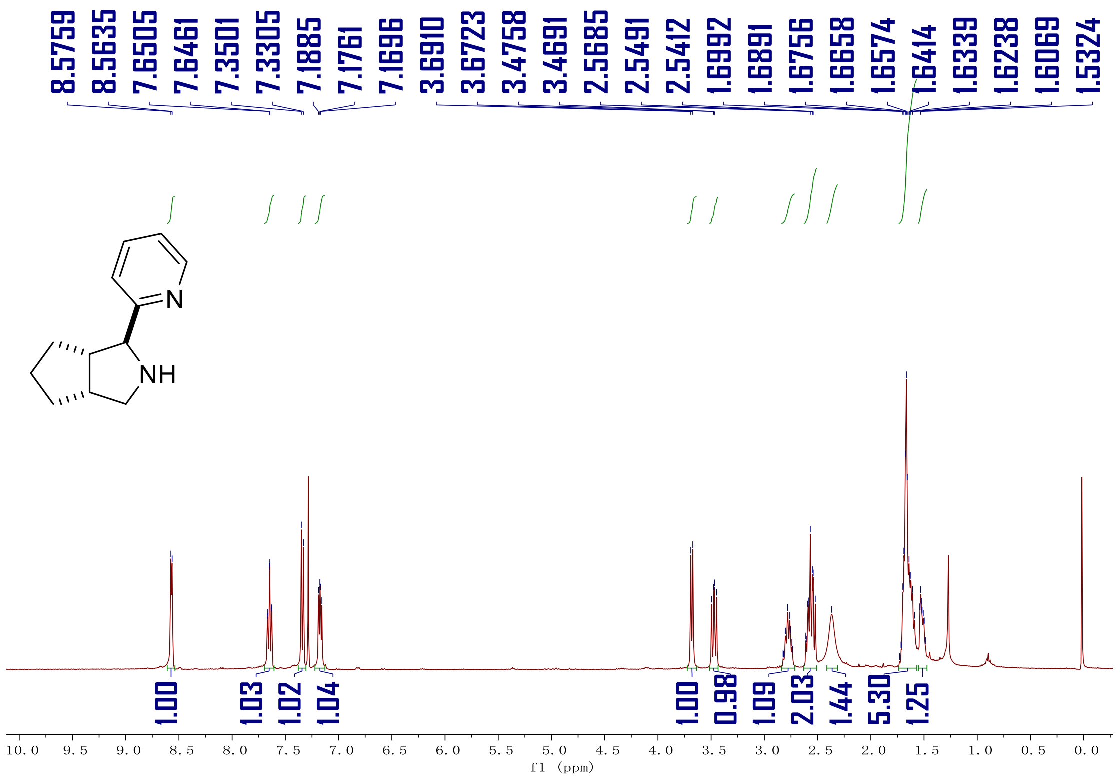

Supplementary Figure 79. ^{1}H NMR (400 MHz, CDCl$_3$) of 40a

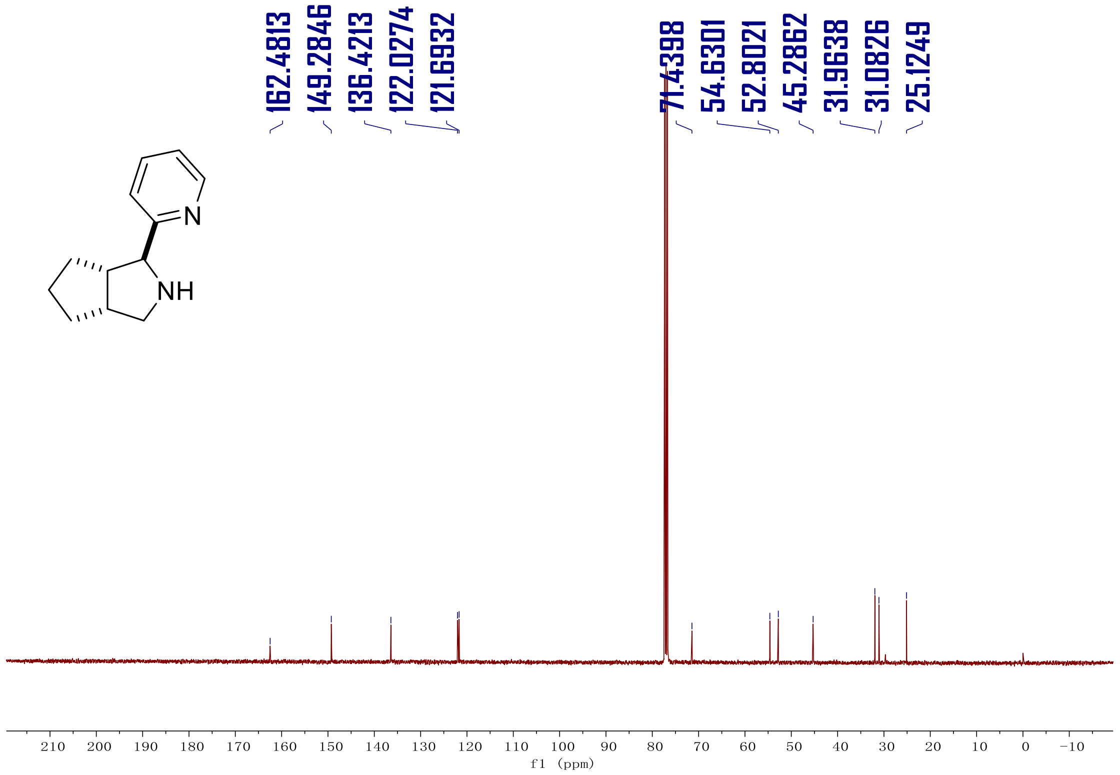

Supplementary Figure 80. ^{13}C NMR (101 MHz, CDCl$_3$) of 40a

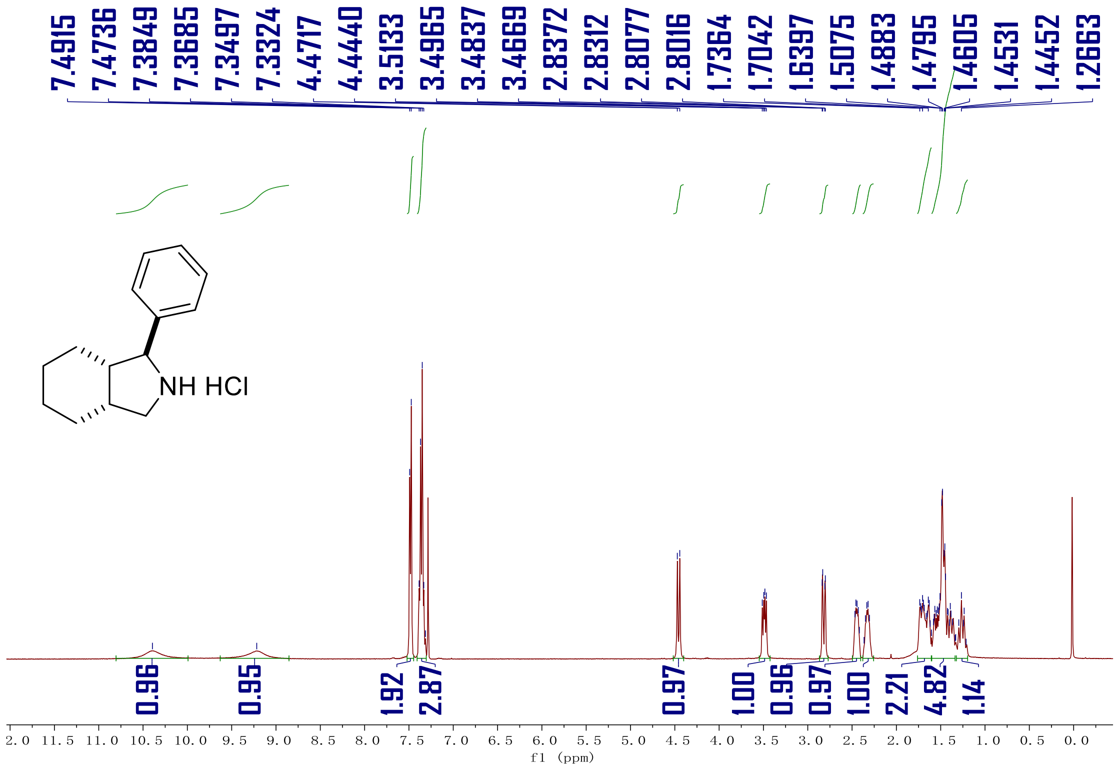

Supplementary Figure 81. ^{1}**H NMR (400 MHz, CDCl**$_3$**) of 41a**

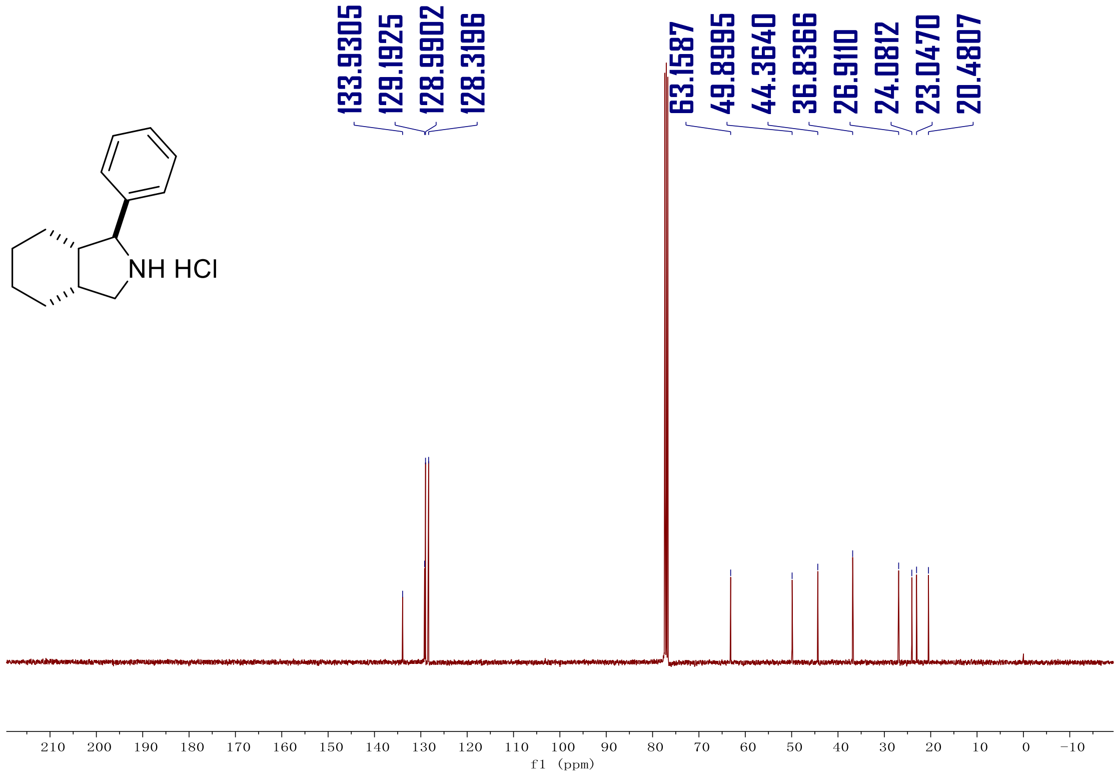

Supplementary Figure 82. ^{13}**C NMR (101 MHz, CDCl**$_3$**) of 41a**

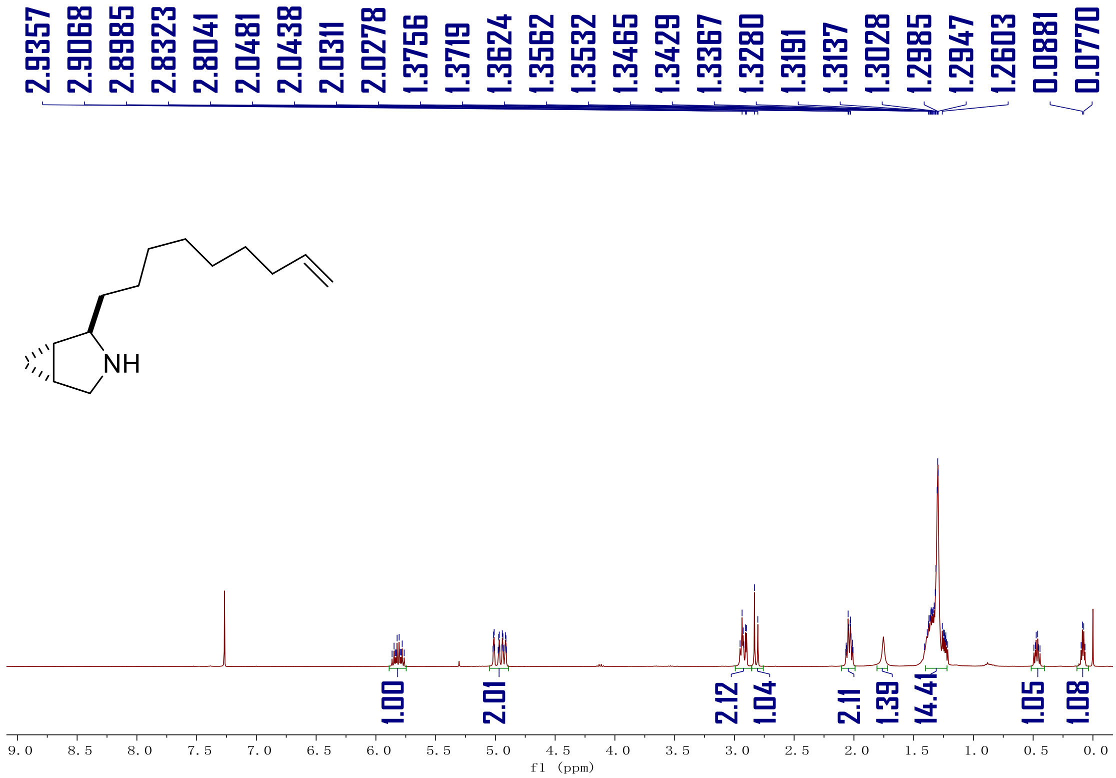

Supplementary Figure 83. ^{1}H NMR (400 MHz, $CDCl_3$) of 42a

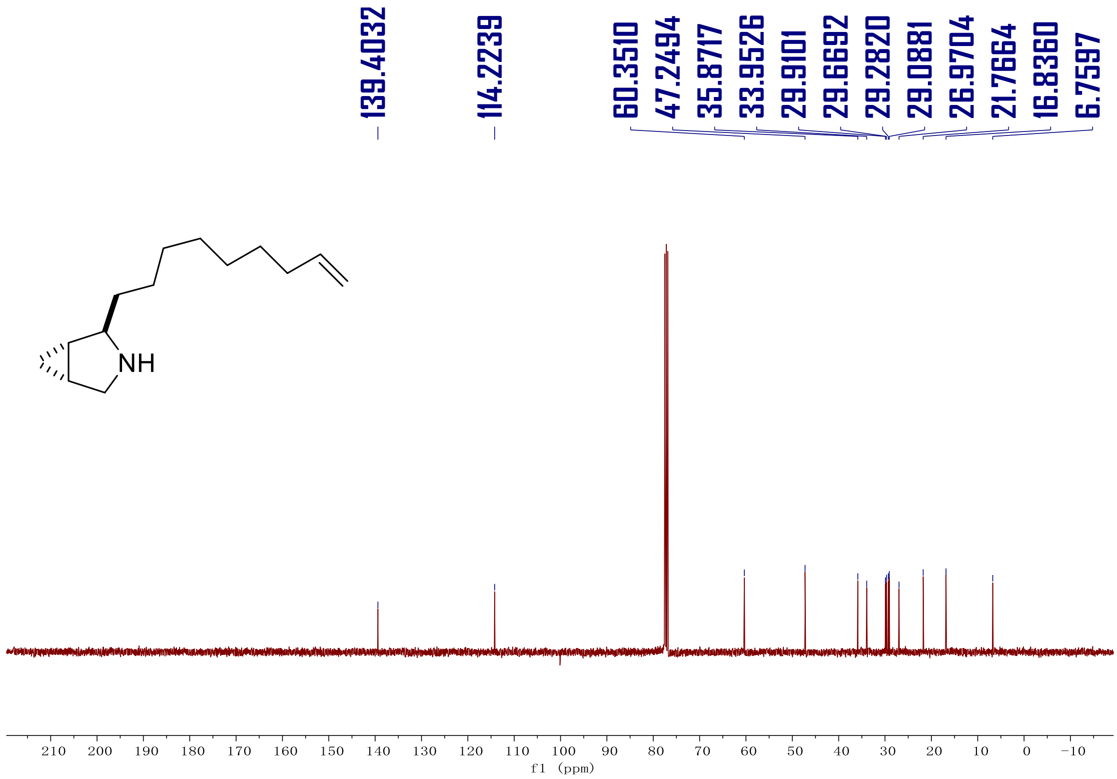

Supplementary Figure 84. ^{13}C NMR (101 MHz, $CDCl_3$) of 42a

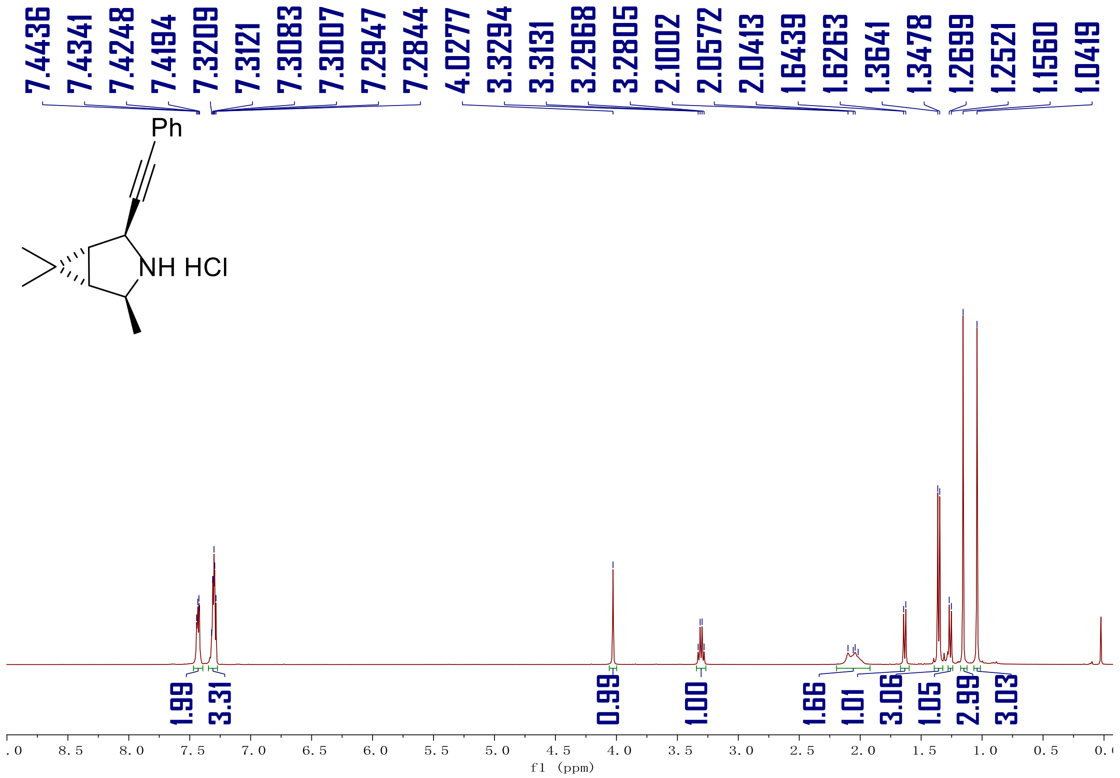

Supplementary Figure 85. ^{1}**H NMR (400 MHz,** $CDCl_3$**) of 43a**

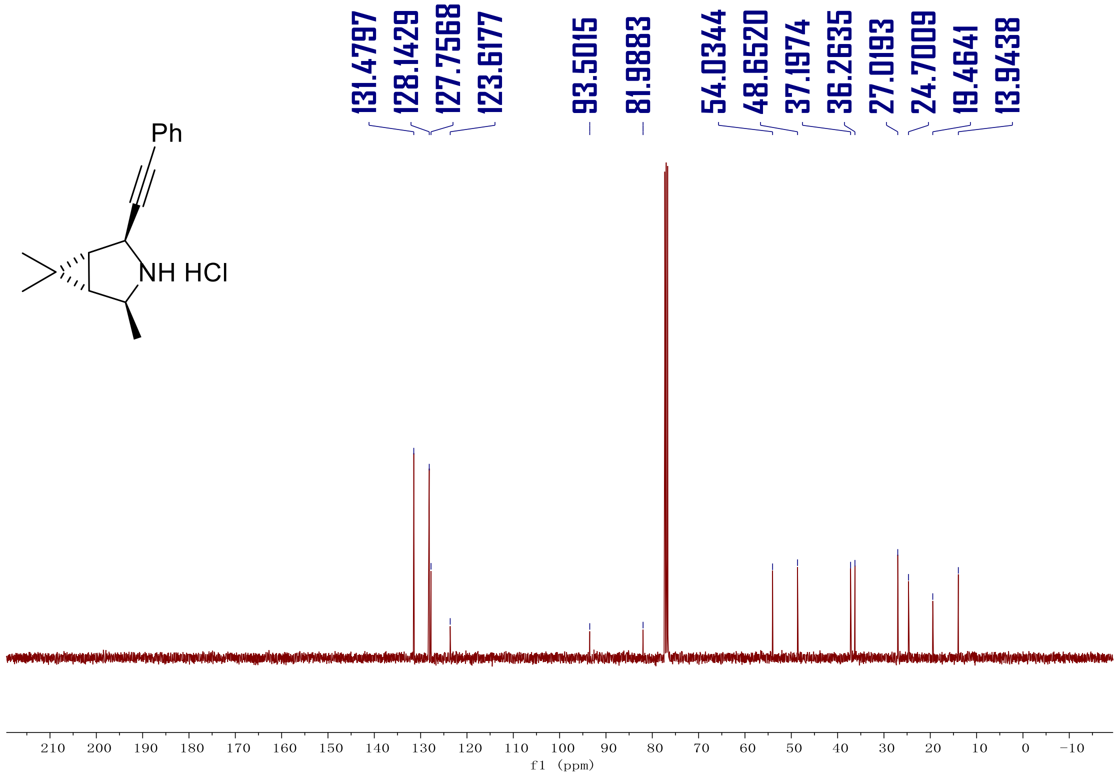

Supplementary Figure 86. ^{13}**C NMR (101 MHz,** $CDCl_3$**) of 43a**

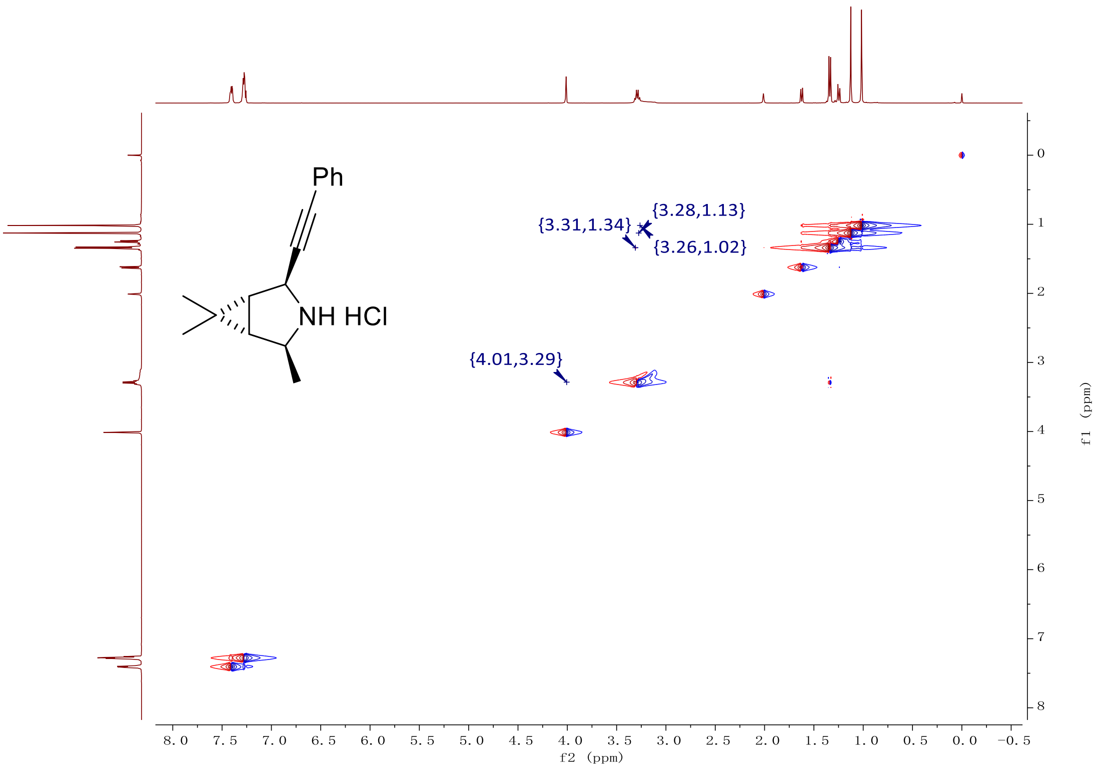

Supplementary Figure 87. NOESY (400 MHz, $CDCl_3$) of 43a

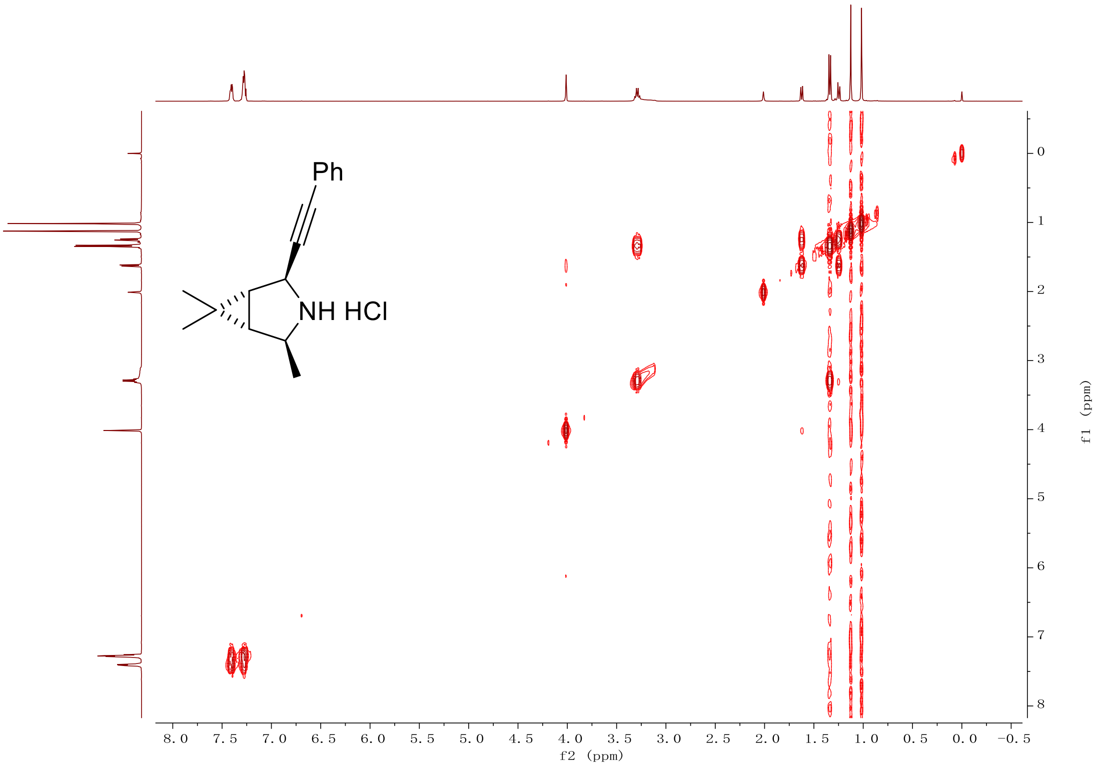

Supplementary Figure 88. COSY (400 MHz, $CDCl_3$) of 43a

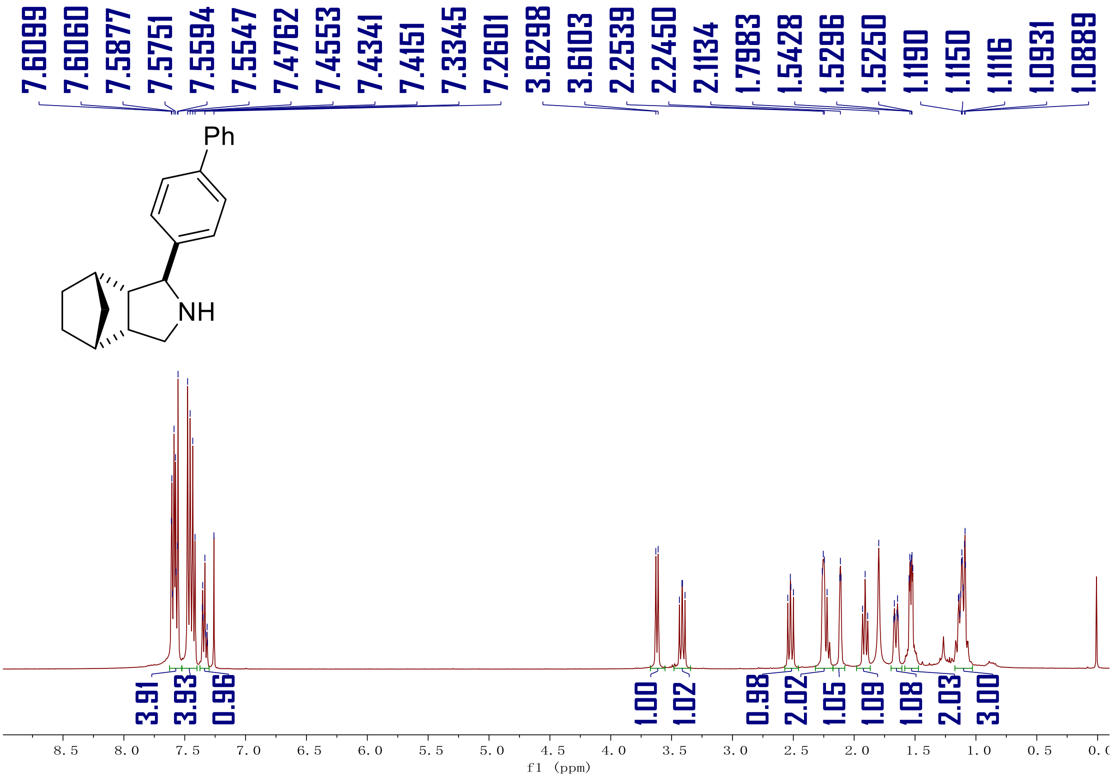

Supplementary Figure 89. ^{1}**H NMR (400 MHz, CDCl$_3$) of 44a**

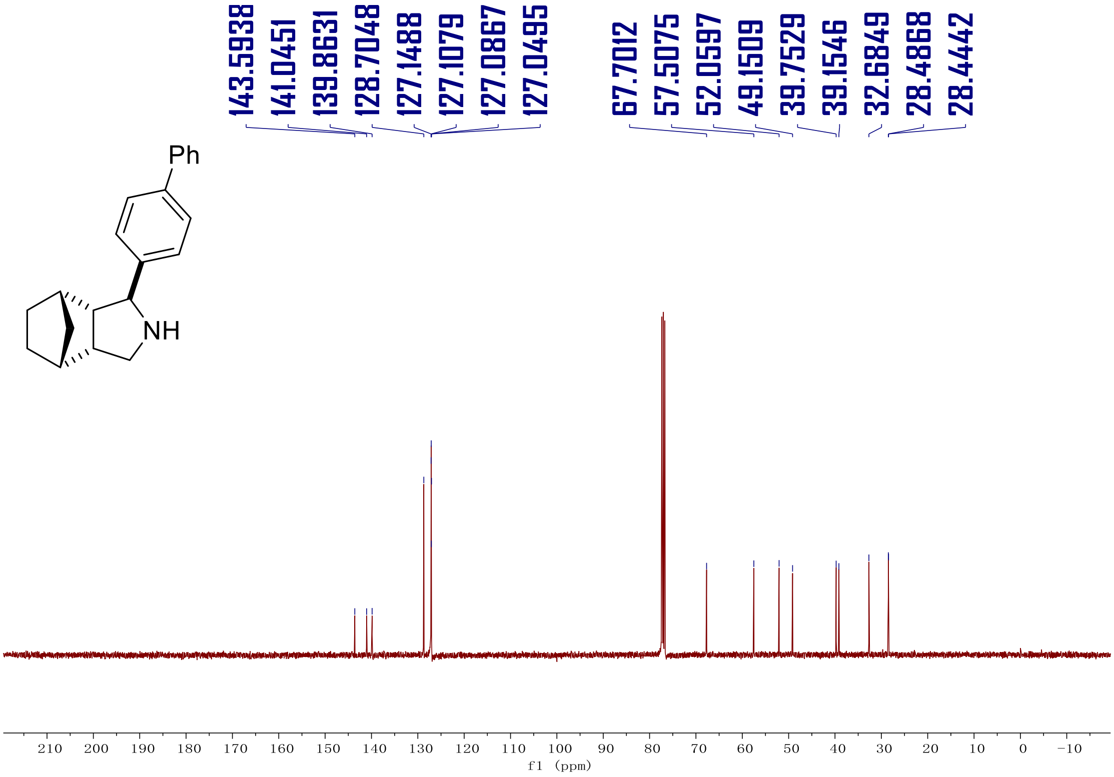

Supplementary Figure 90. ^{13}**C NMR (101 MHz, CDCl$_3$) of 44a**

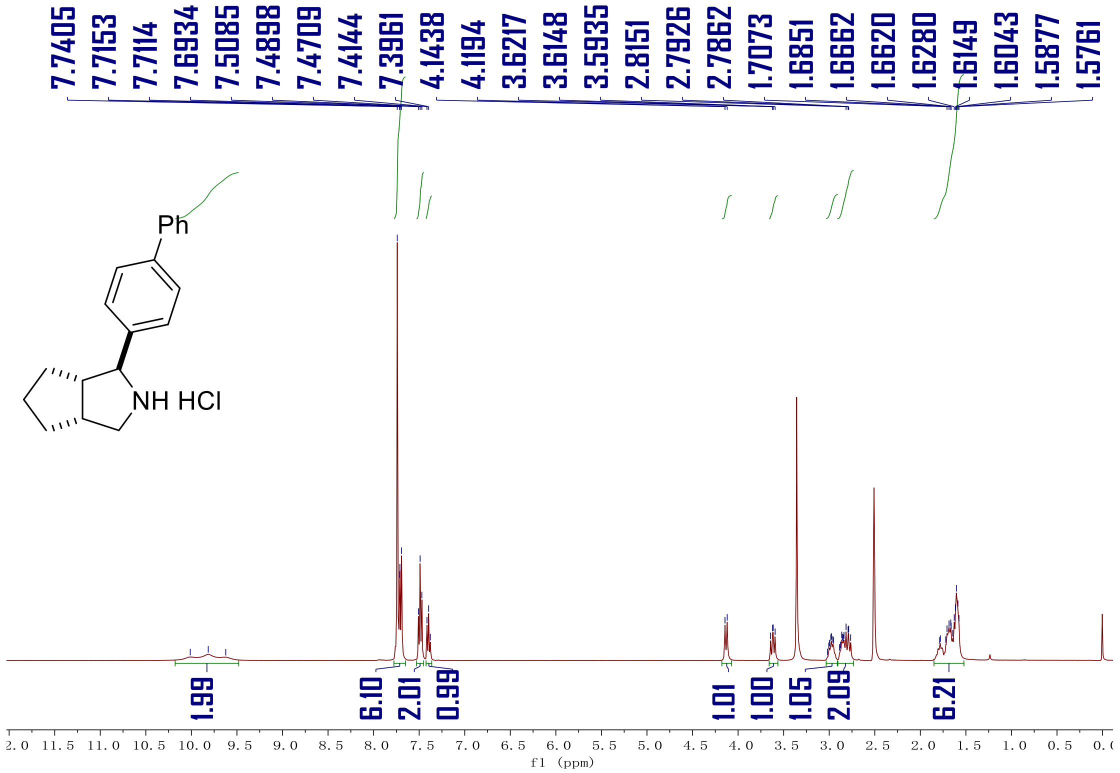

Supplementary Figure 91. ^{1}**H NMR (400 MHz, DMSO-*d*$_6$) of 45a**

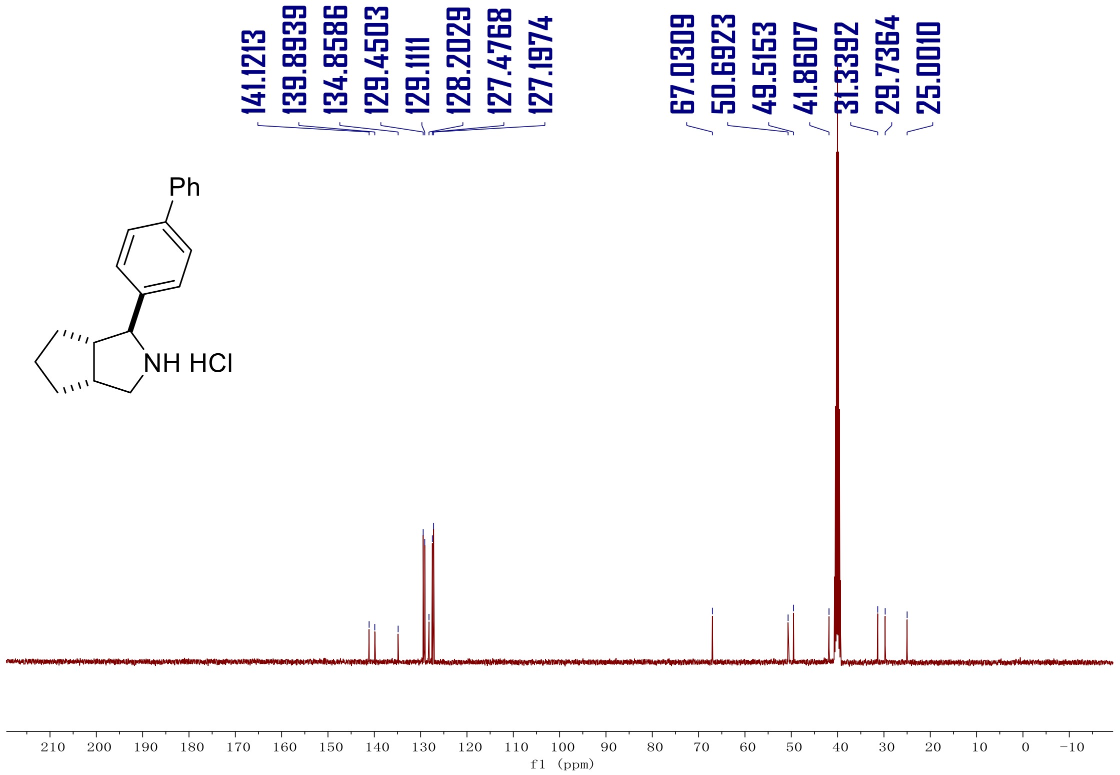

Supplementary Figure 92. ^{13}**C NMR (101 MHz, DMSO-*d*$_6$) of 45a**

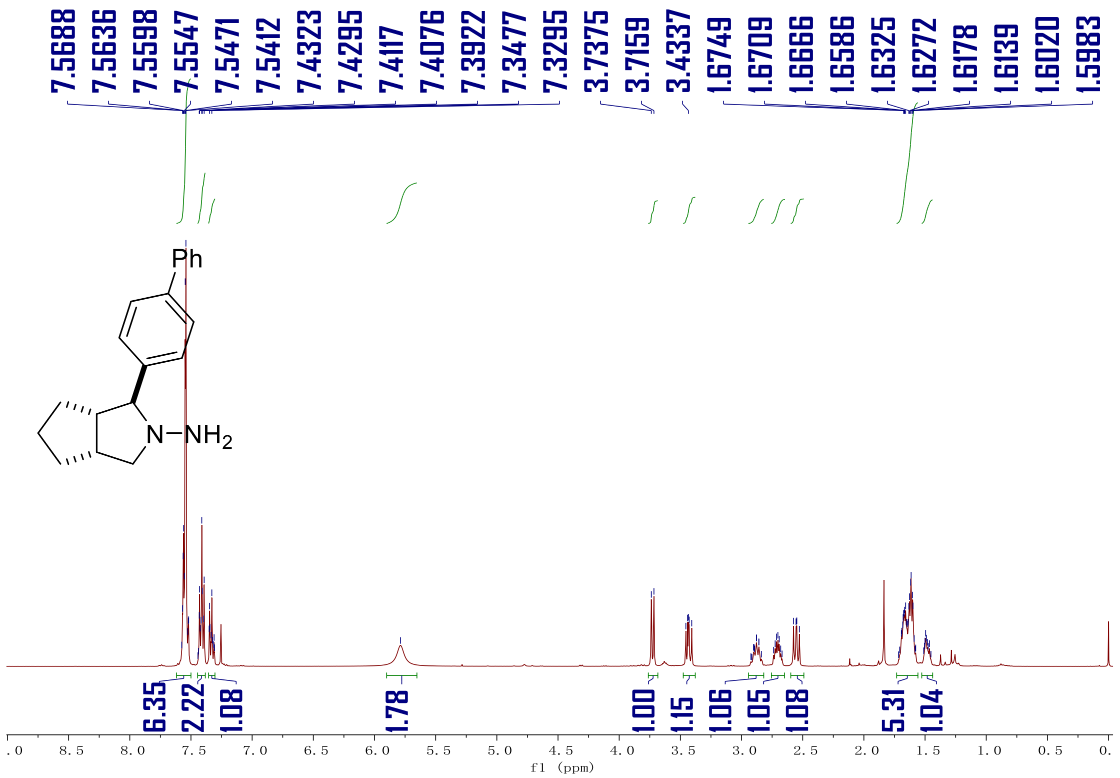

Supplementary Figure 93. ^{1}H NMR (400 MHz, CDCl$_3$) of 45d

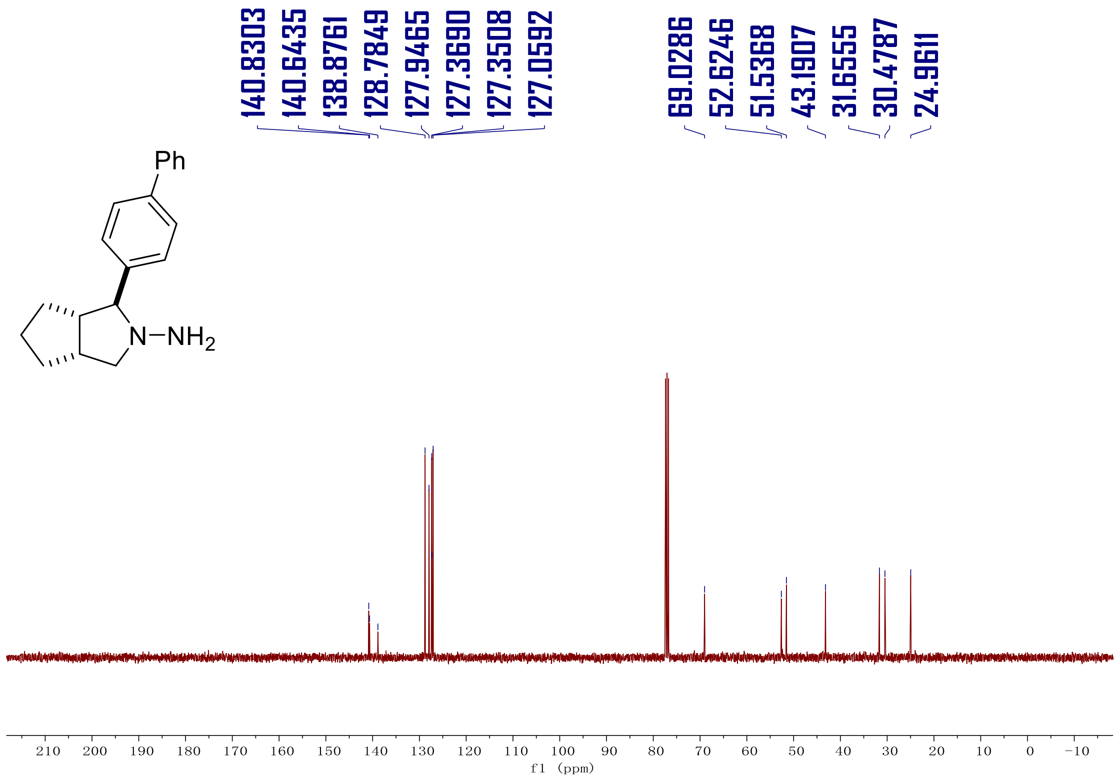

Supplementary Figure 94. ^{13}C NMR (101 MHz, CDCl$_3$) of 45d

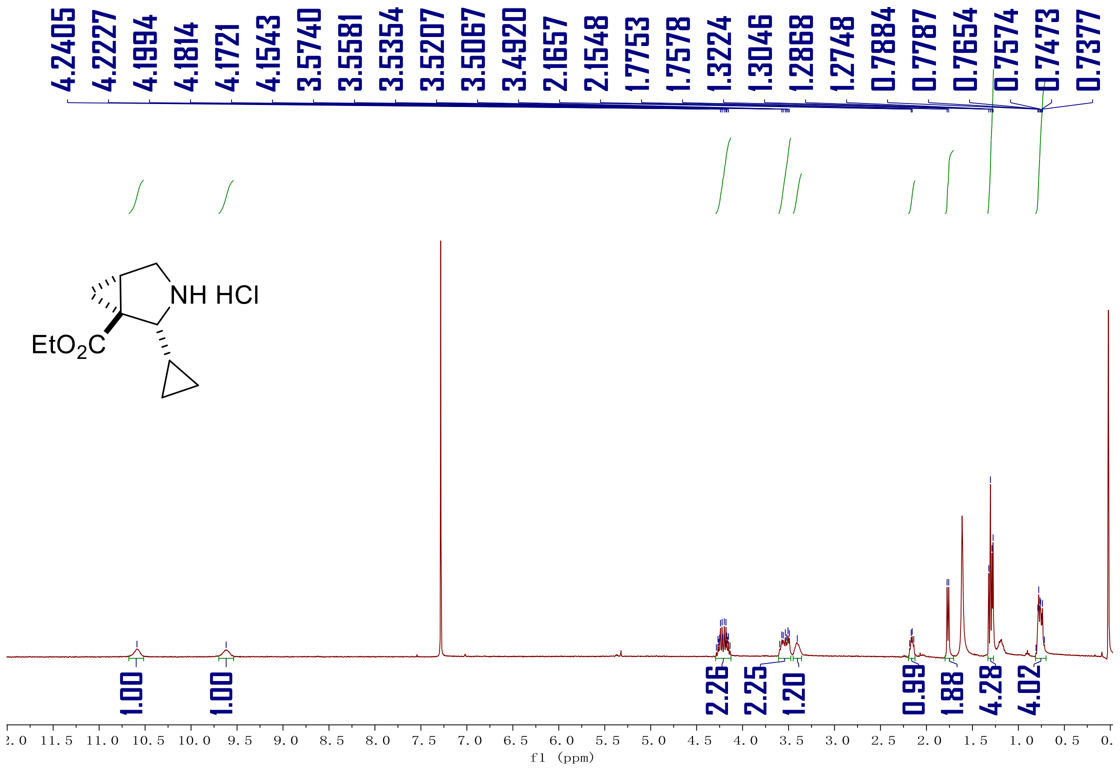

Supplementary Figure 95. ^{1}H NMR (400 MHz, $CDCl_3$) of 46a

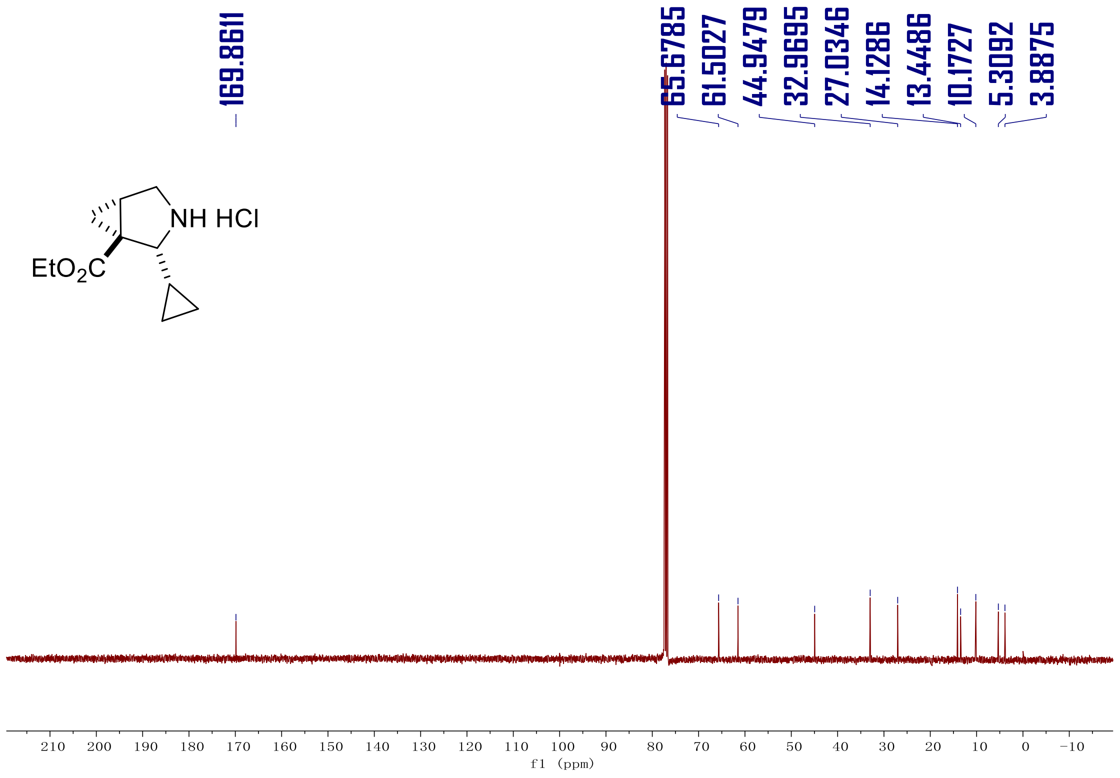

Supplementary Figure 96. ^{13}C NMR (101 MHz, $CDCl_3$) of 46a

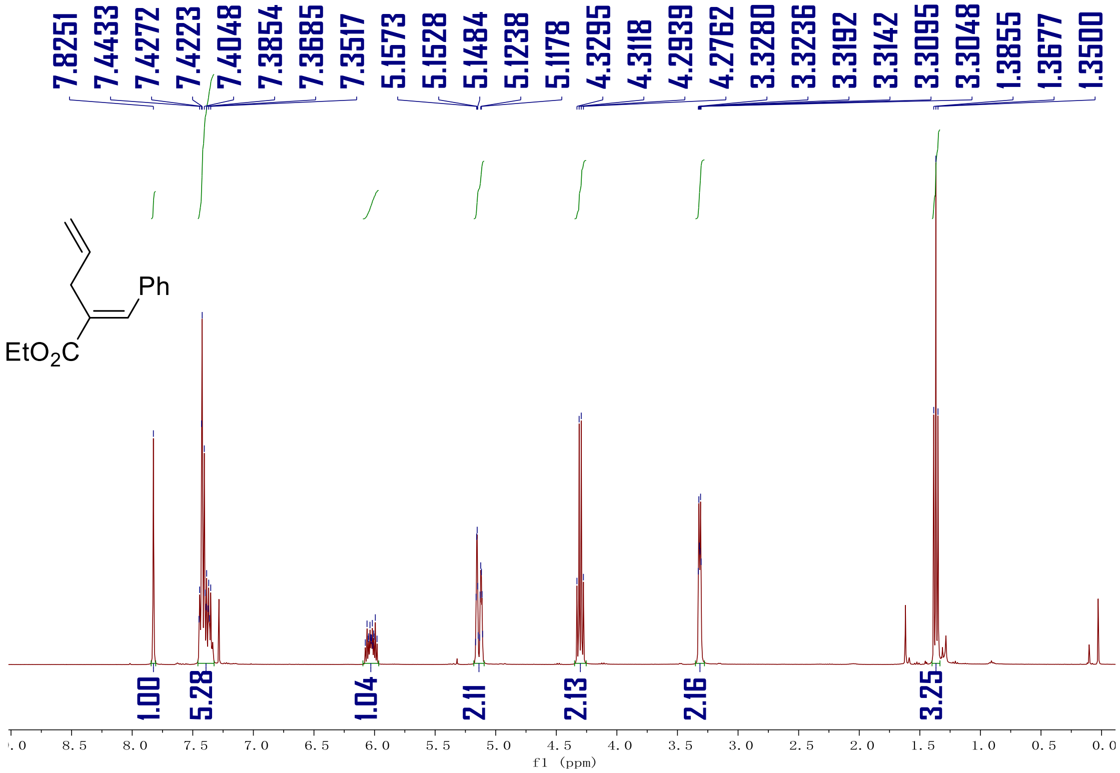

Supplementary Figure 97. ^{1}H NMR (400 MHz, $CDCl_3$) of 1b

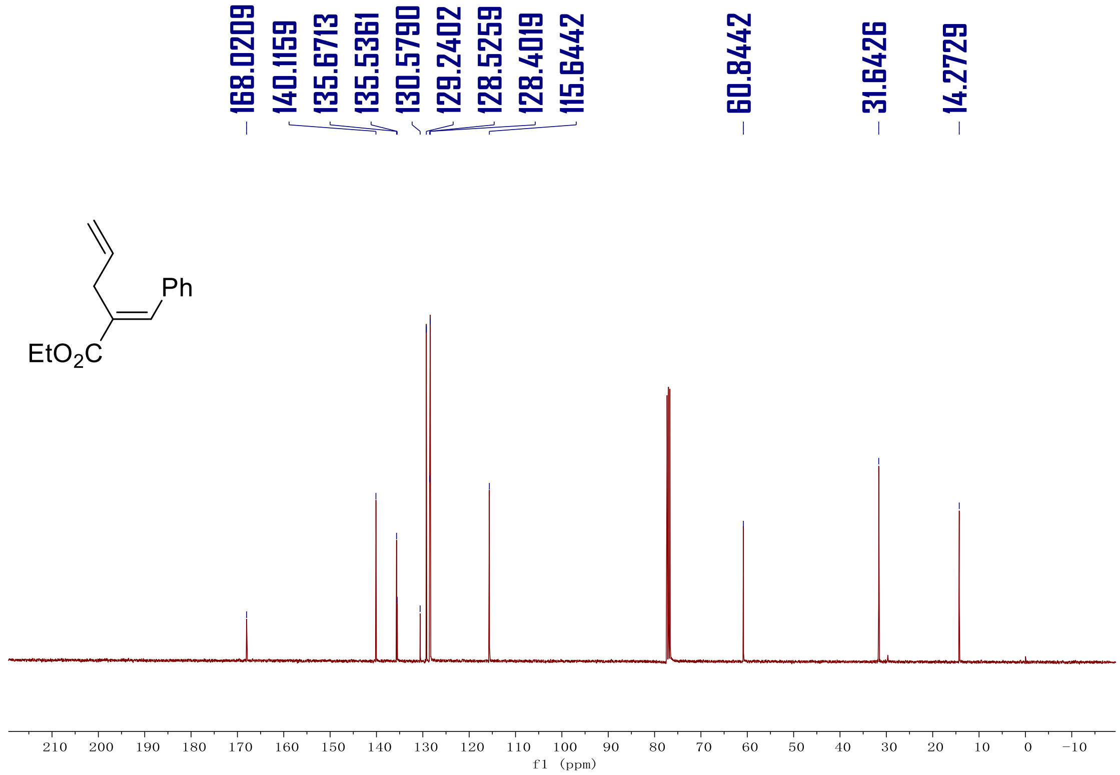

Supplementary Figure 98. ^{13}C NMR (101 MHz, $CDCl_3$) of 1b

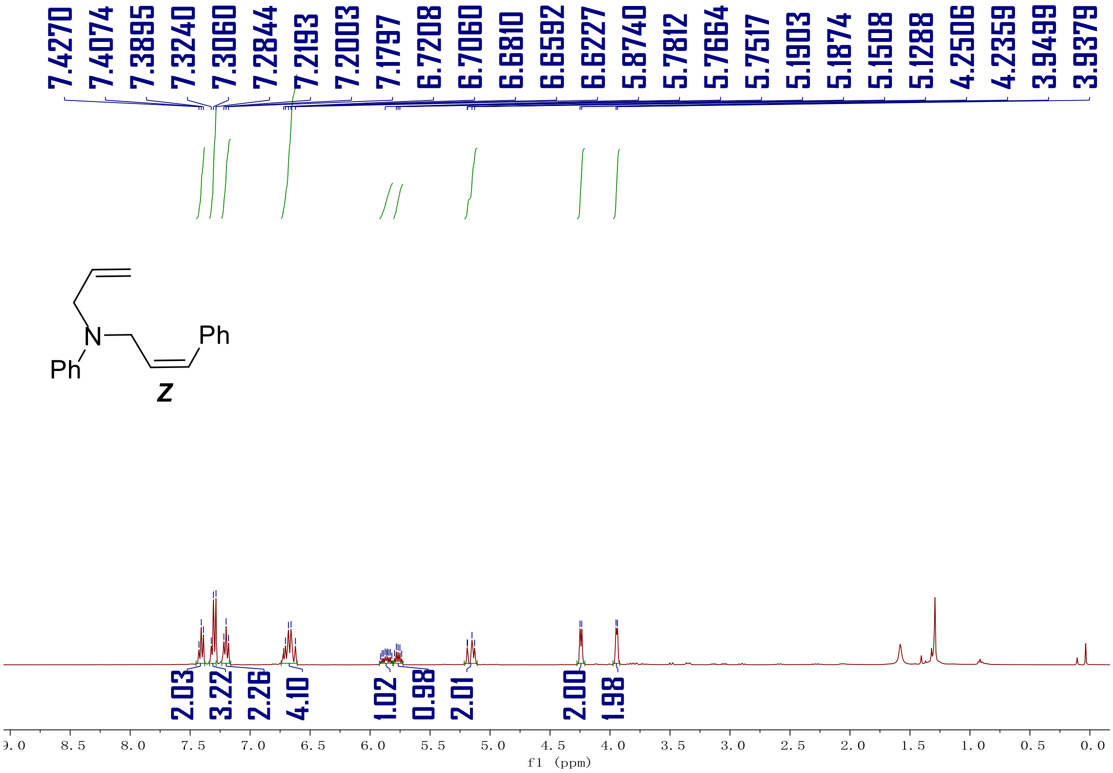

Supplementary Figure 99. ^{1}H NMR (400 MHz, $CDCl_3$) of 2b

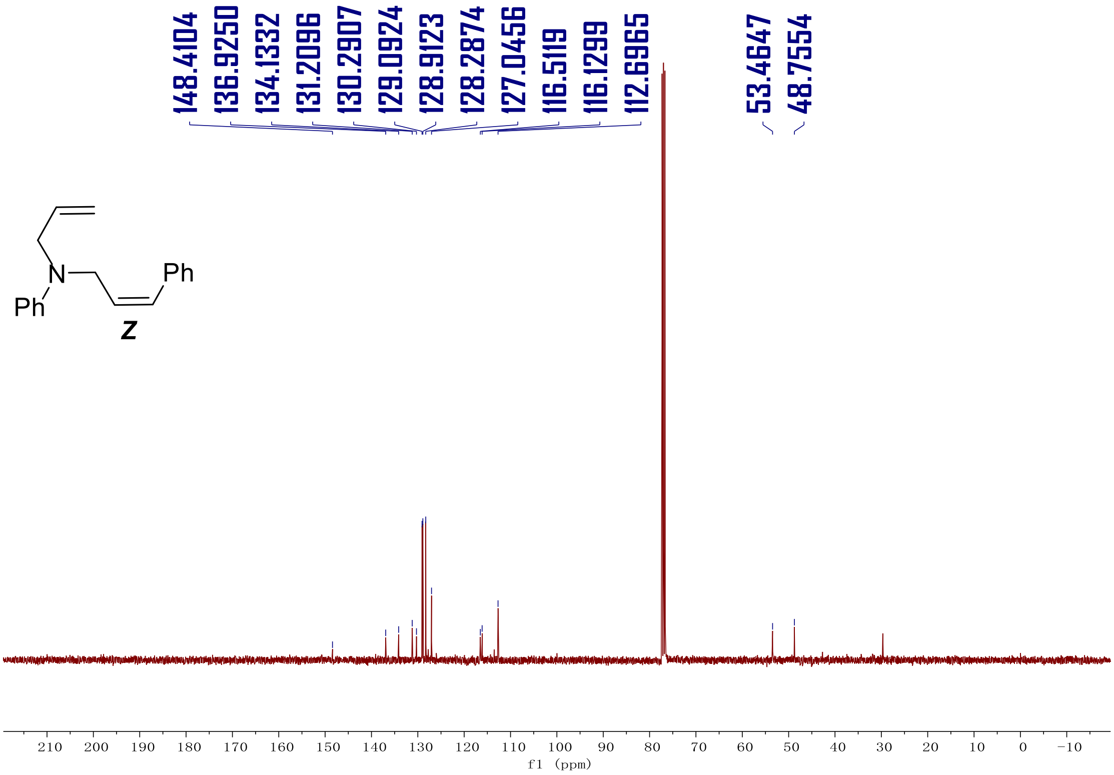

Supplementary Figure 100. ^{13}C NMR (101 MHz, $CDCl_3$) of 2b

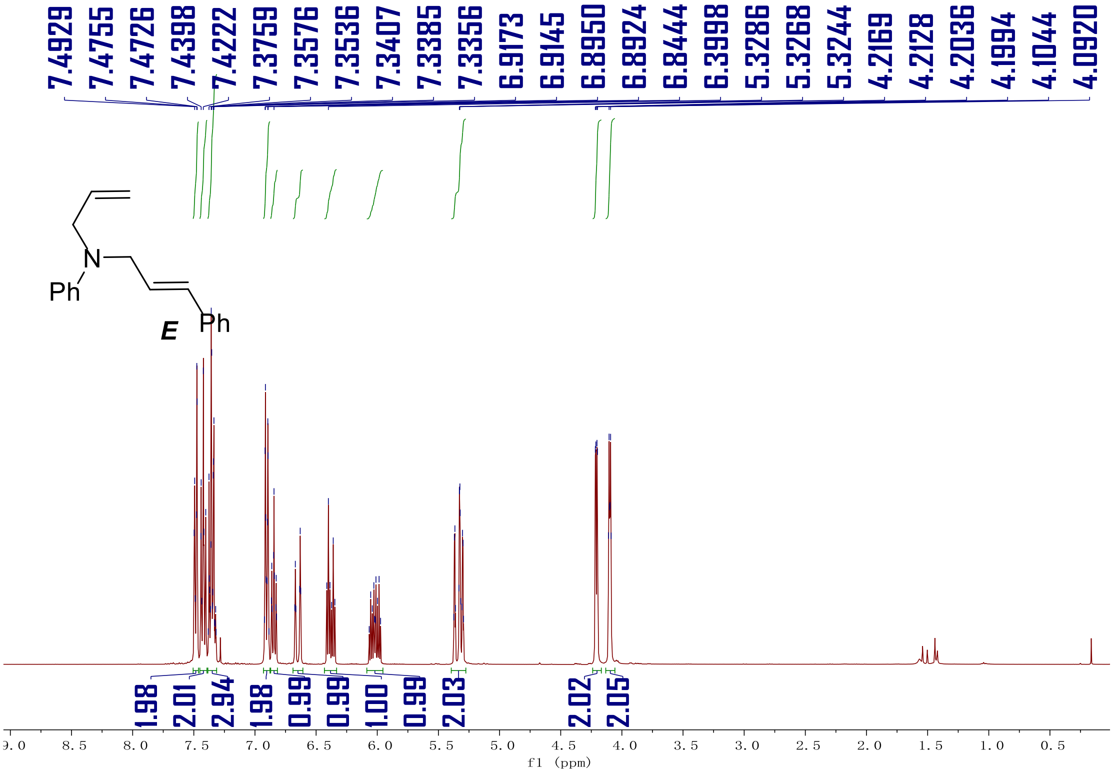

Supplementary Figure 101. ^{1}**H NMR (400 MHz, CDCl**$_3$**) of 3b**

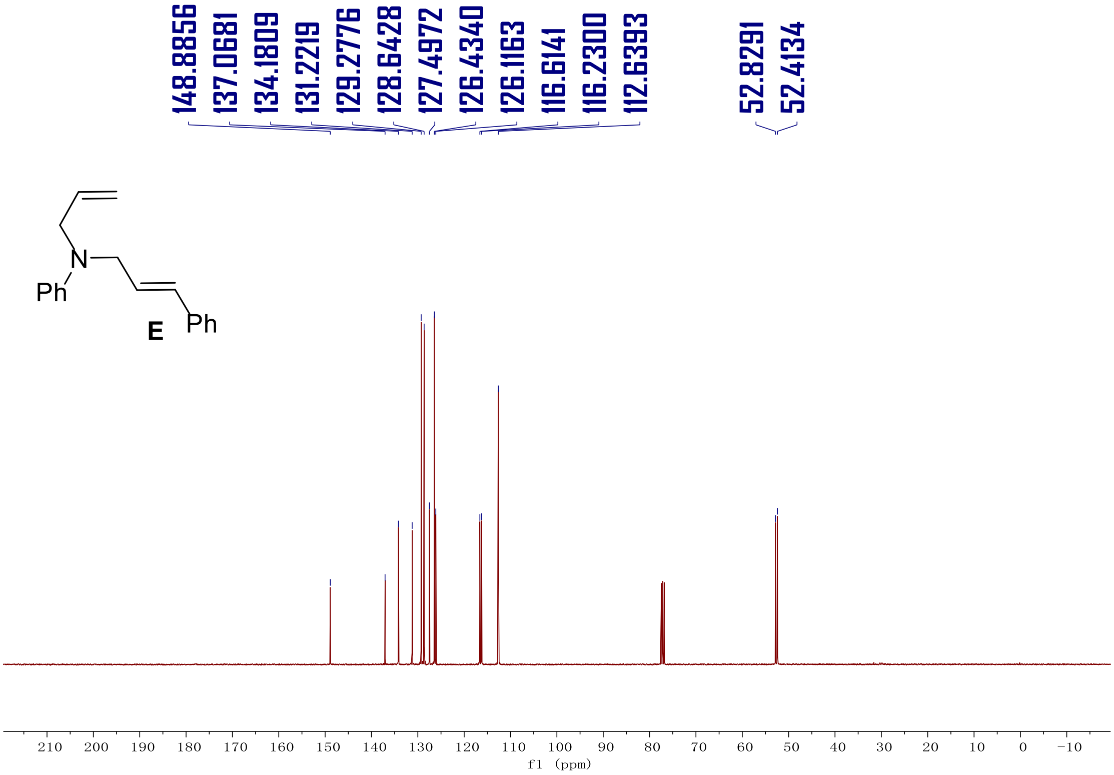

Supplementary Figure 102. ^{13}**C NMR (101 MHz, CDCl**$_3$**) of 3b**

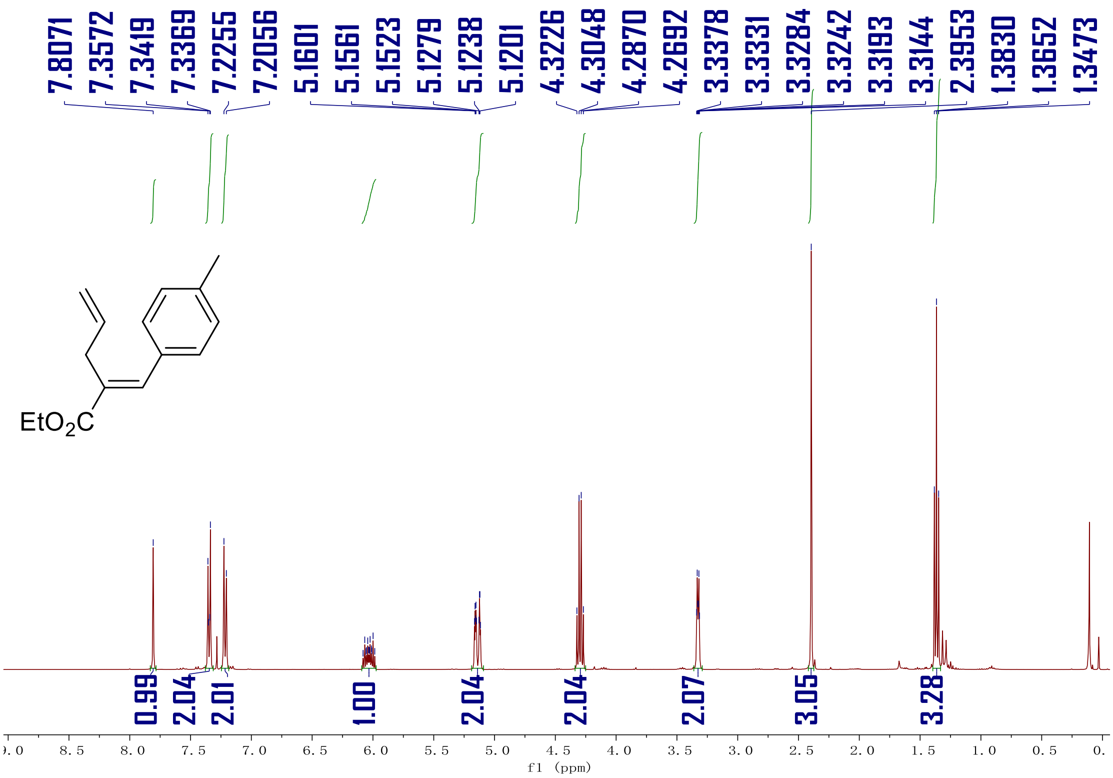

Supplementary Figure 103. ^{1}**H NMR (400 MHz, CDCl3) of 4b**

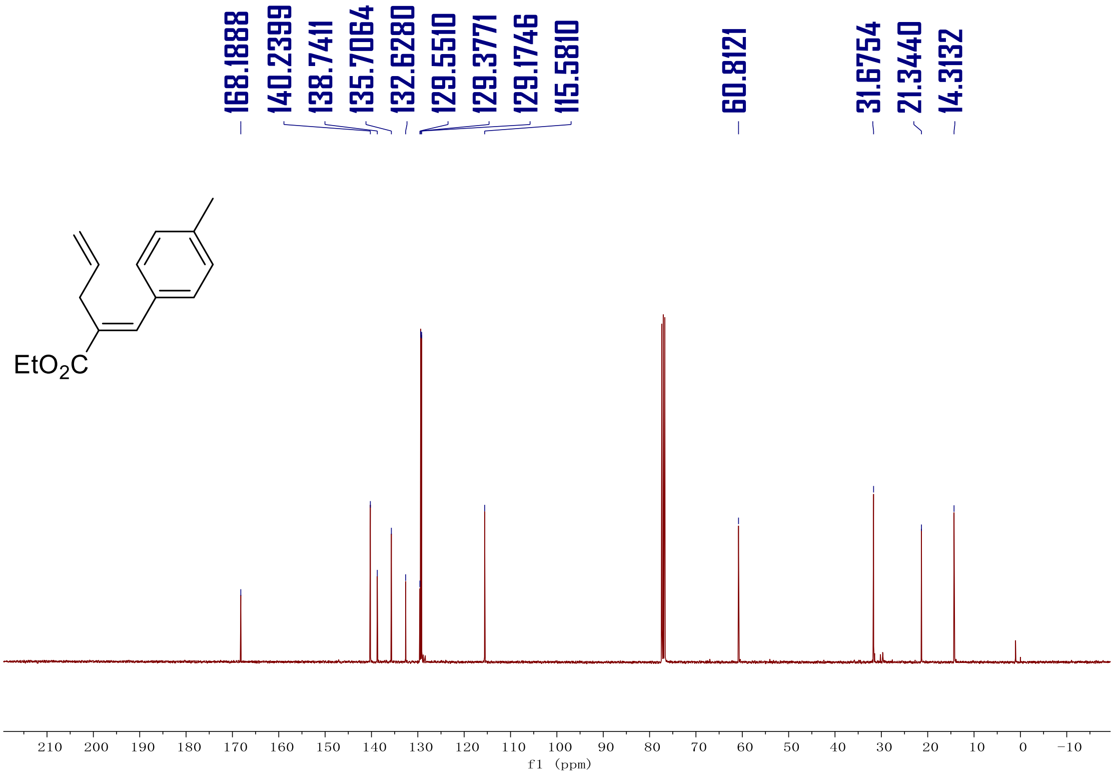

Supplementary Figure 104. ^{13}**C NMR (101 MHz, CDCl3) of 4b**

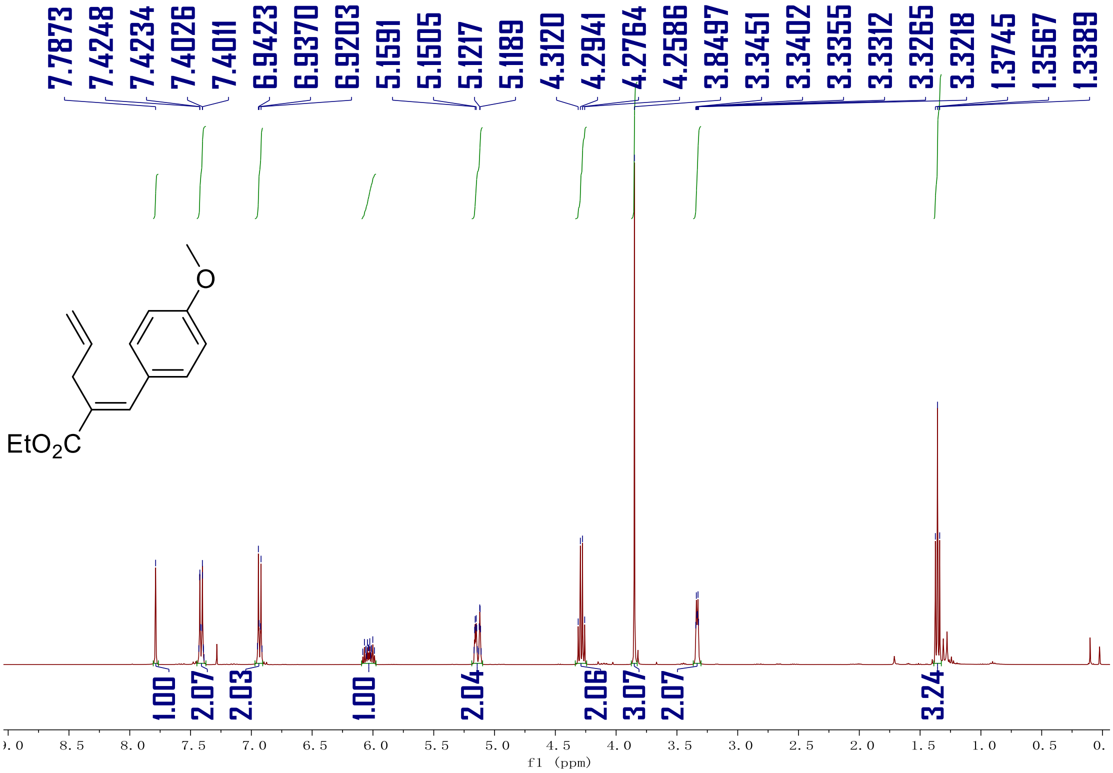

Supplementary Figure 105. ^{1}**H NMR (400 MHz, CDCl**$_3$**) of 5b**

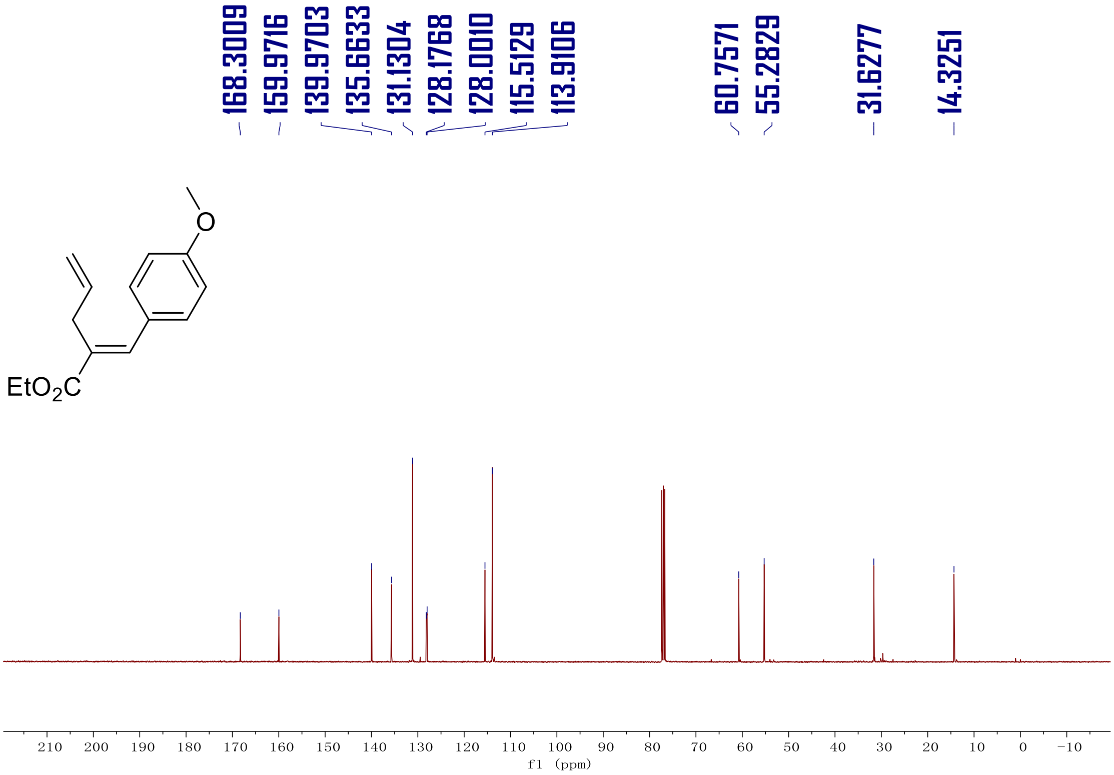

Supplementary Figure 106. ^{13}**C NMR (101 MHz, CDCl**$_3$**) of 5b**

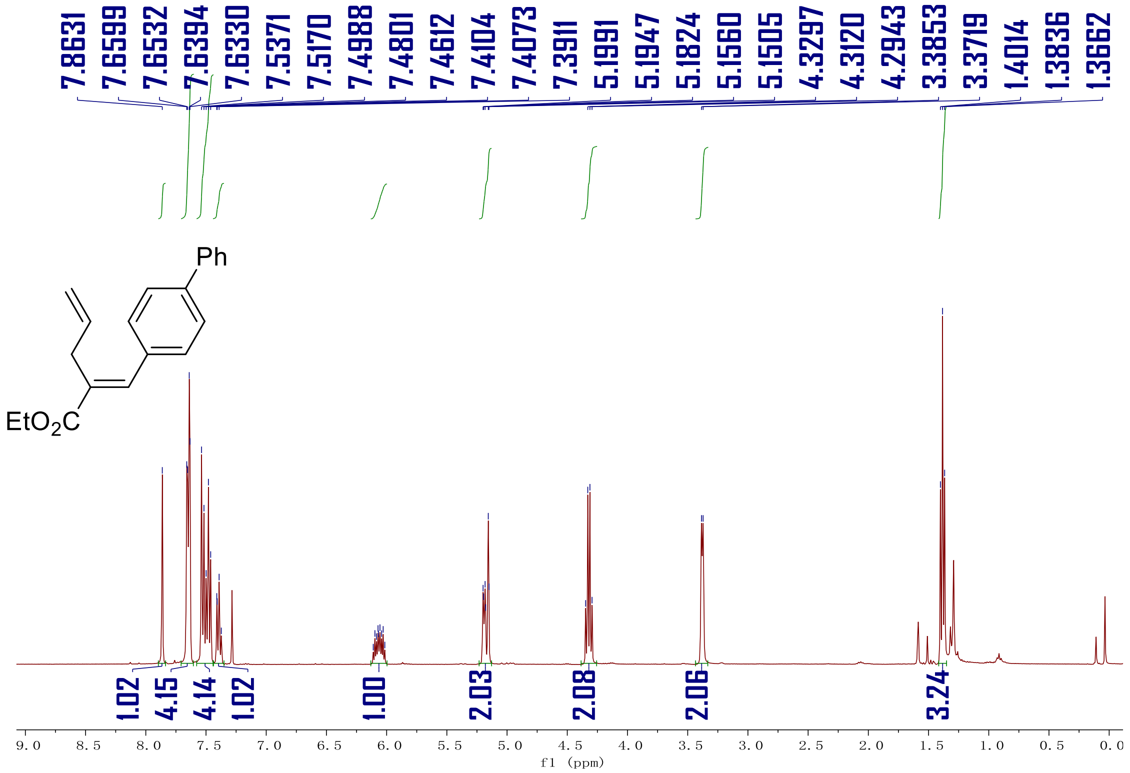

Supplementary Figure 107. ^{1}**H NMR (400 MHz, CDCl**$_{3}$**) of 6b**

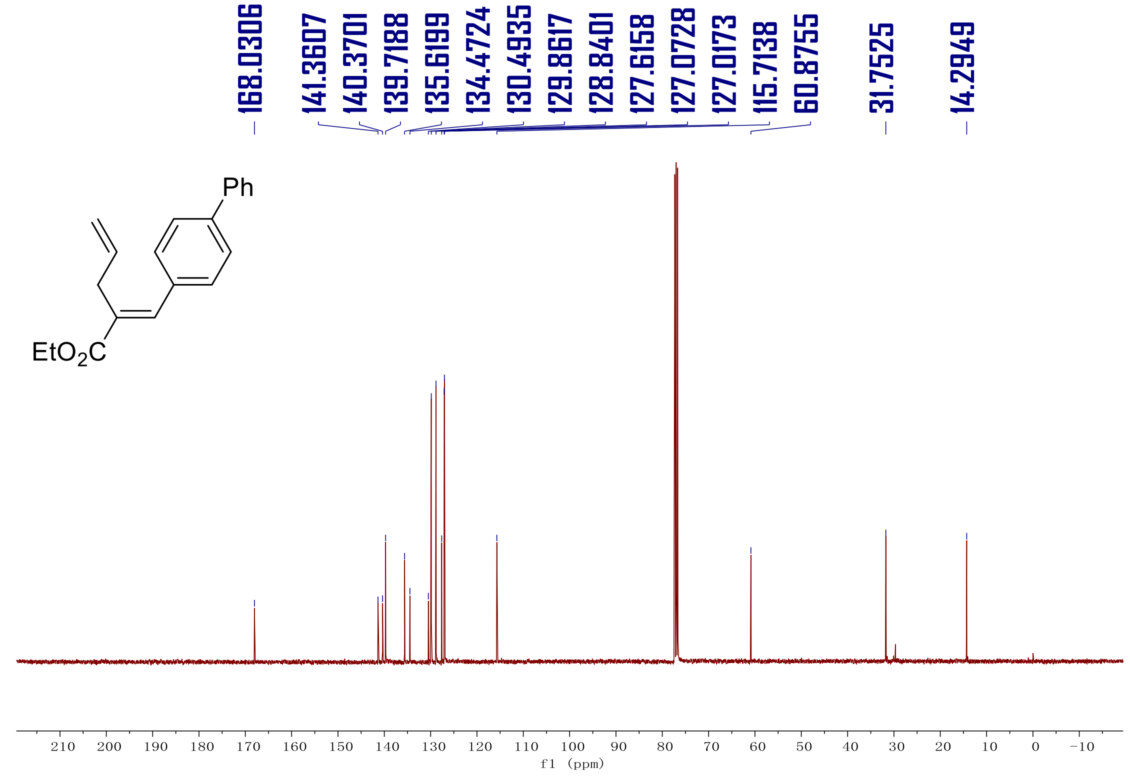

Supplementary Figure 108. ^{13}**C NMR (101 MHz, CDCl**$_{3}$**) of 6b**

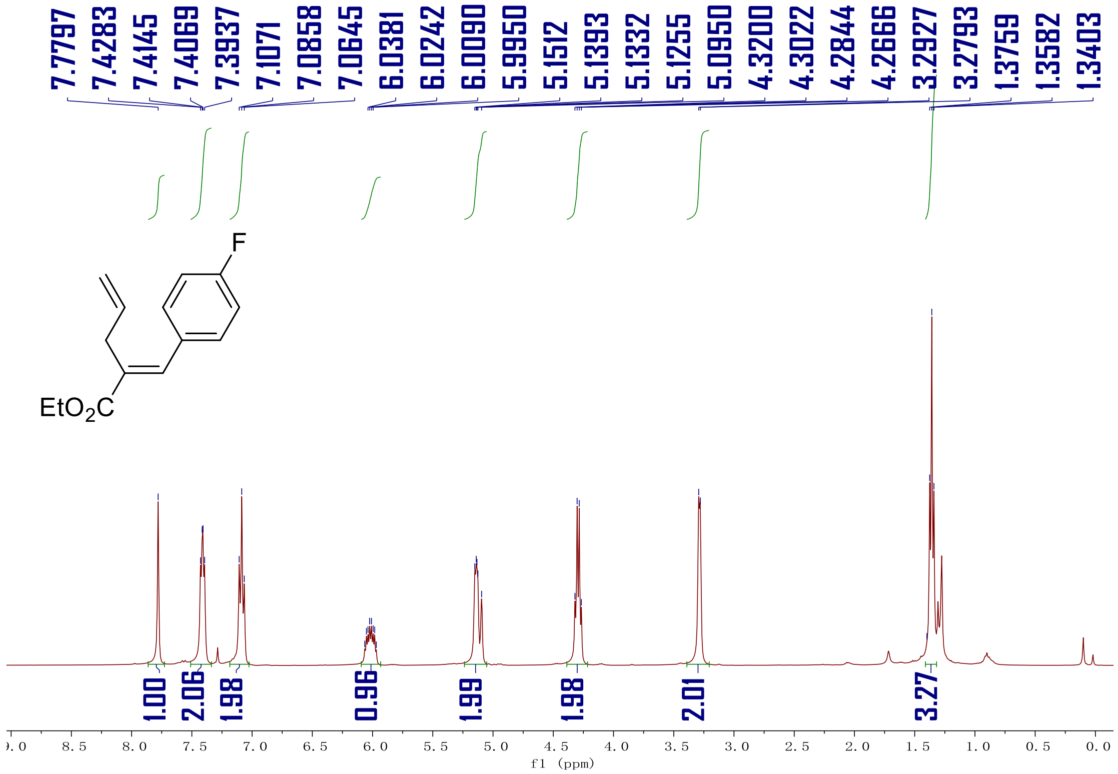

Supplementary Figure 109. ^{1}H NMR (400 MHz, $CDCl_3$) of 7b

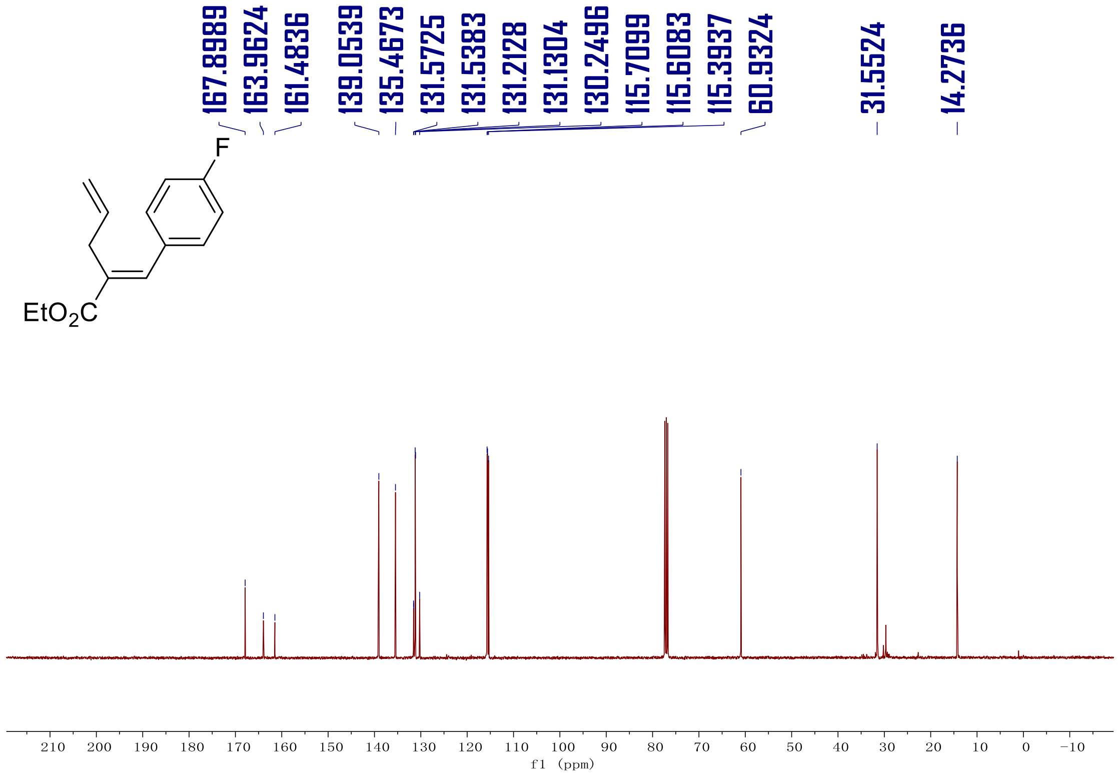

Supplementary Figure 110. ^{13}C NMR (101 MHz, $CDCl_3$) of 7b

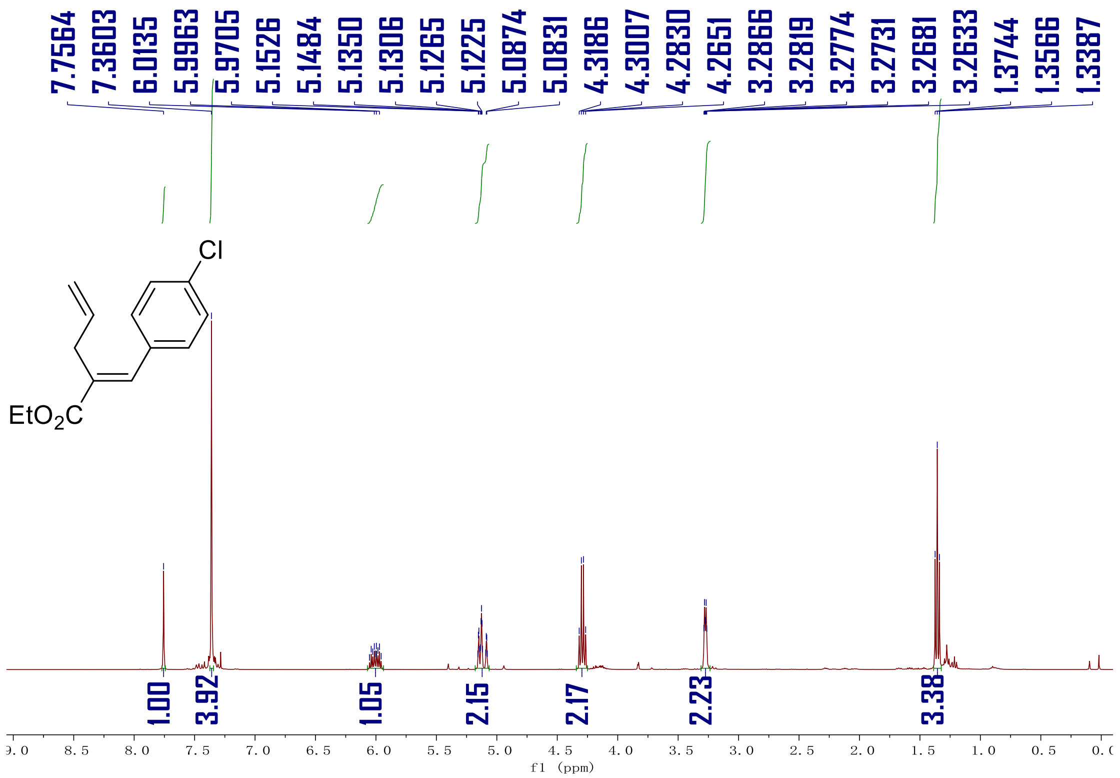

Supplementary Figure 111. ^{1}H NMR (400 MHz, CDCl3) of 8b

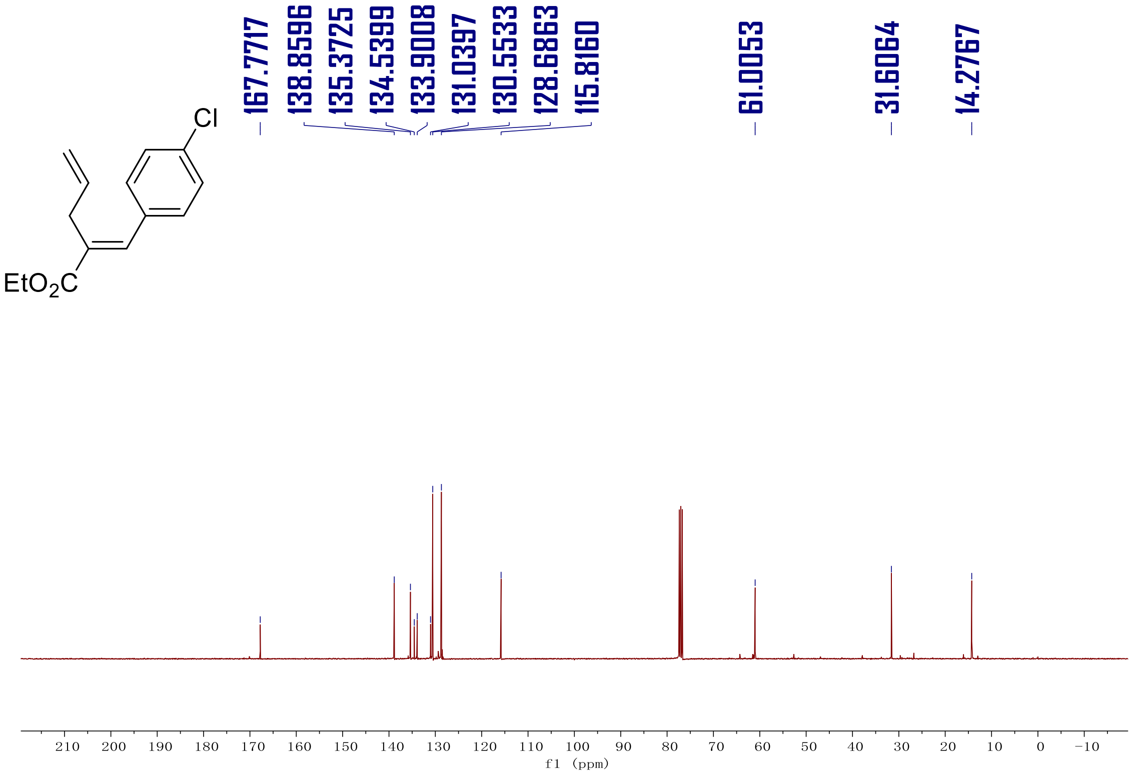

Supplementary Figure 112. ^{13}C NMR (101 MHz, CDCl3) of 8b

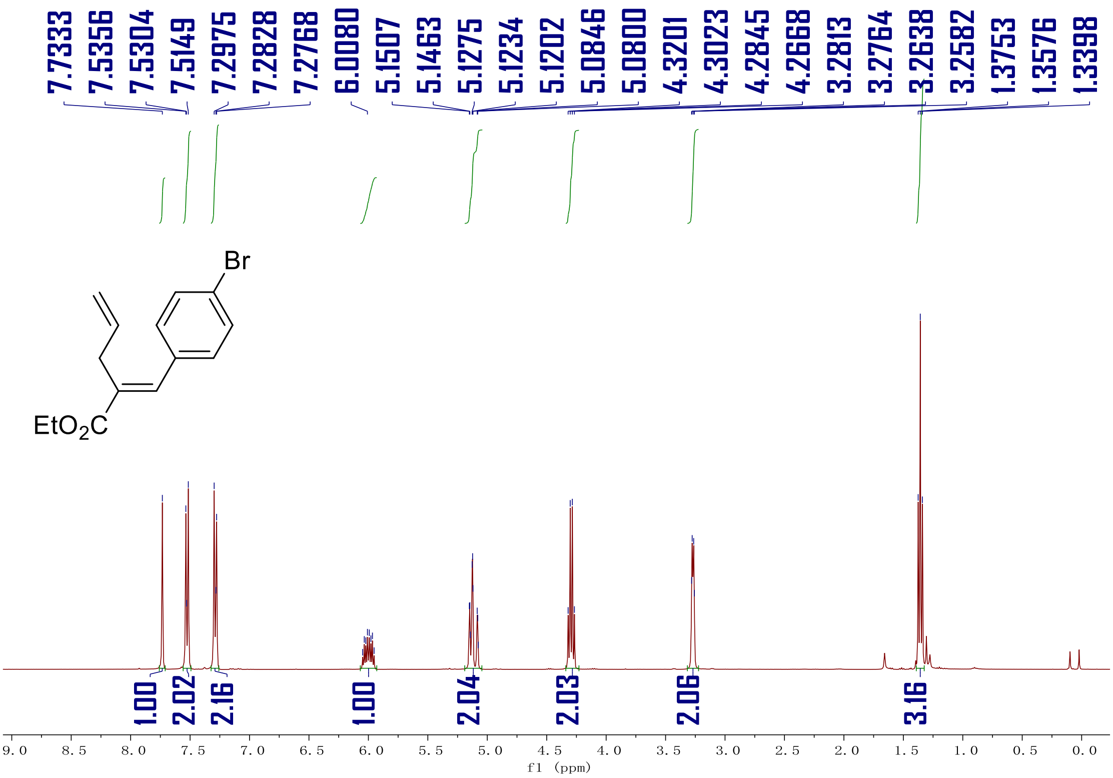

Supplementary Figure 113. **^{1}H NMR (400 MHz, $CDCl_3$) of 9b**

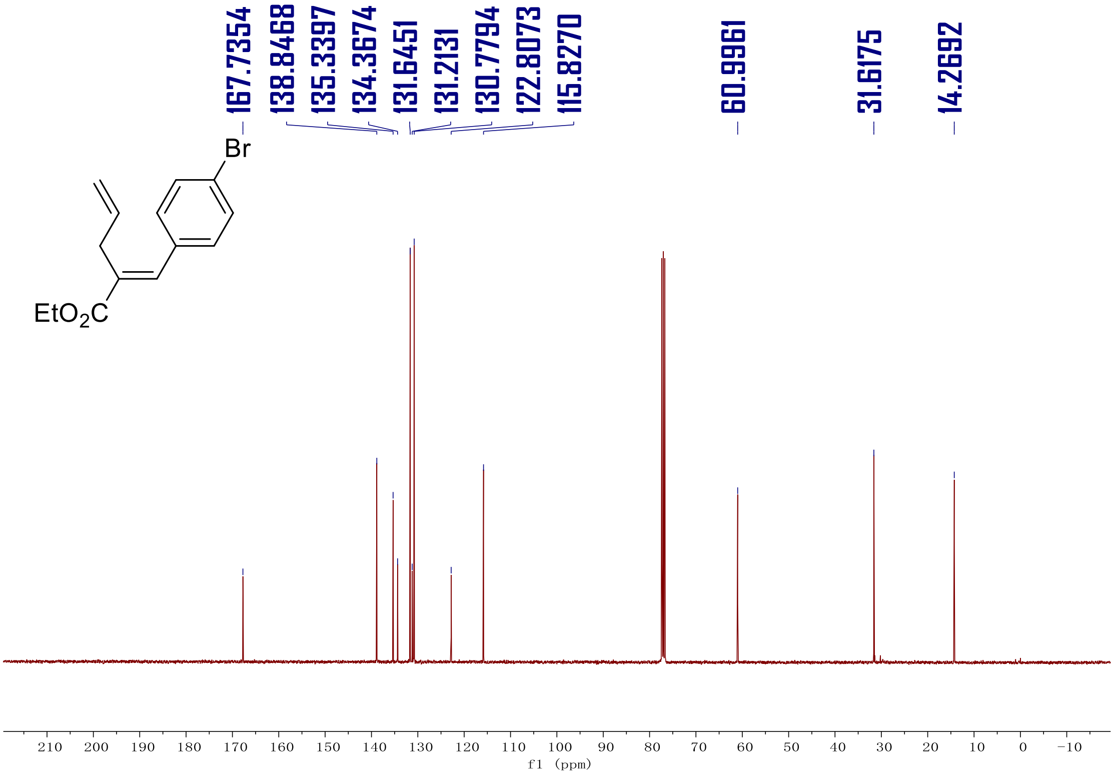

Supplementary Figure 114. **^{13}C NMR (101 MHz, $CDCl_3$) of 9b**

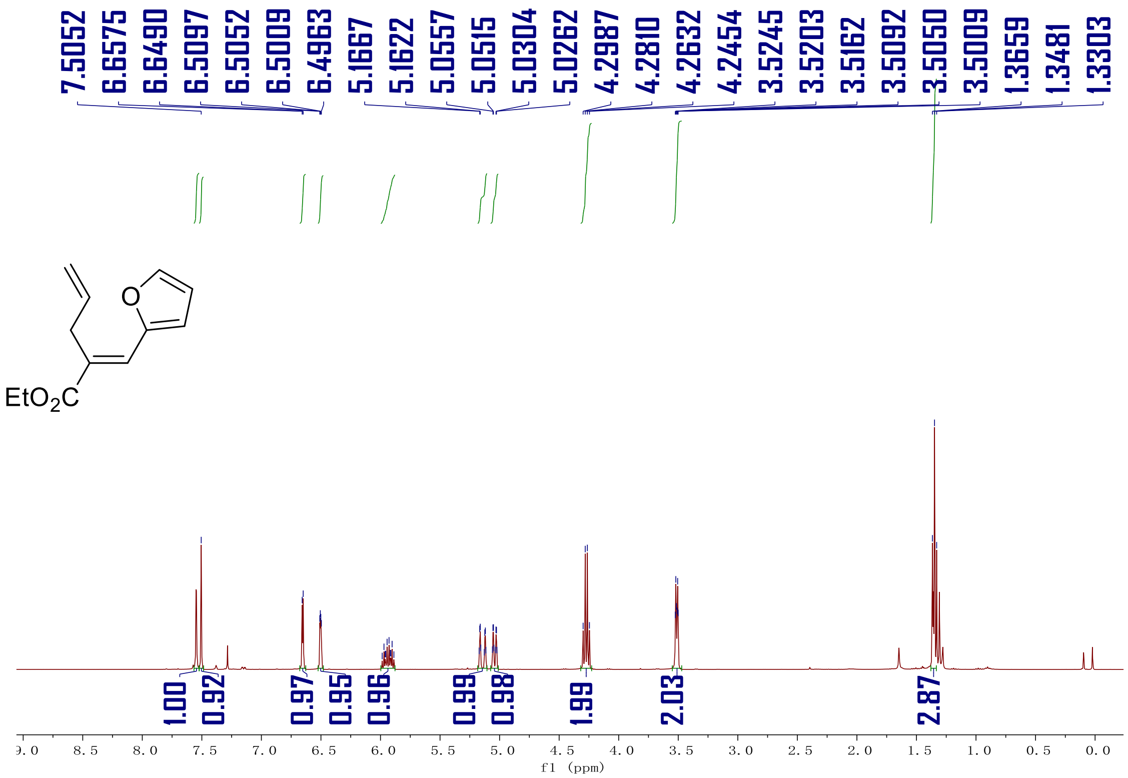

Supplementary Figure 115. ^{1}**H NMR (400 MHz, CDCl**$_3$**) of 10b**

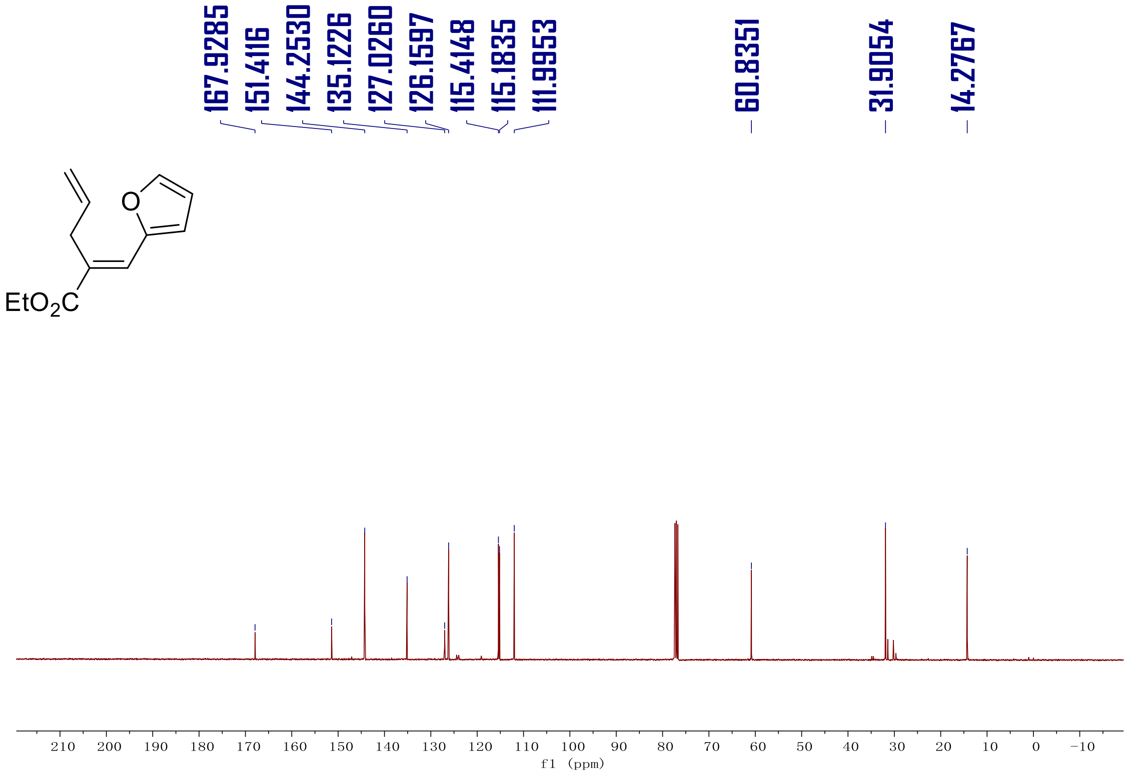

Supplementary Figure 116. ^{13}**C NMR (101 MHz, CDCl**$_3$**) of 10b**

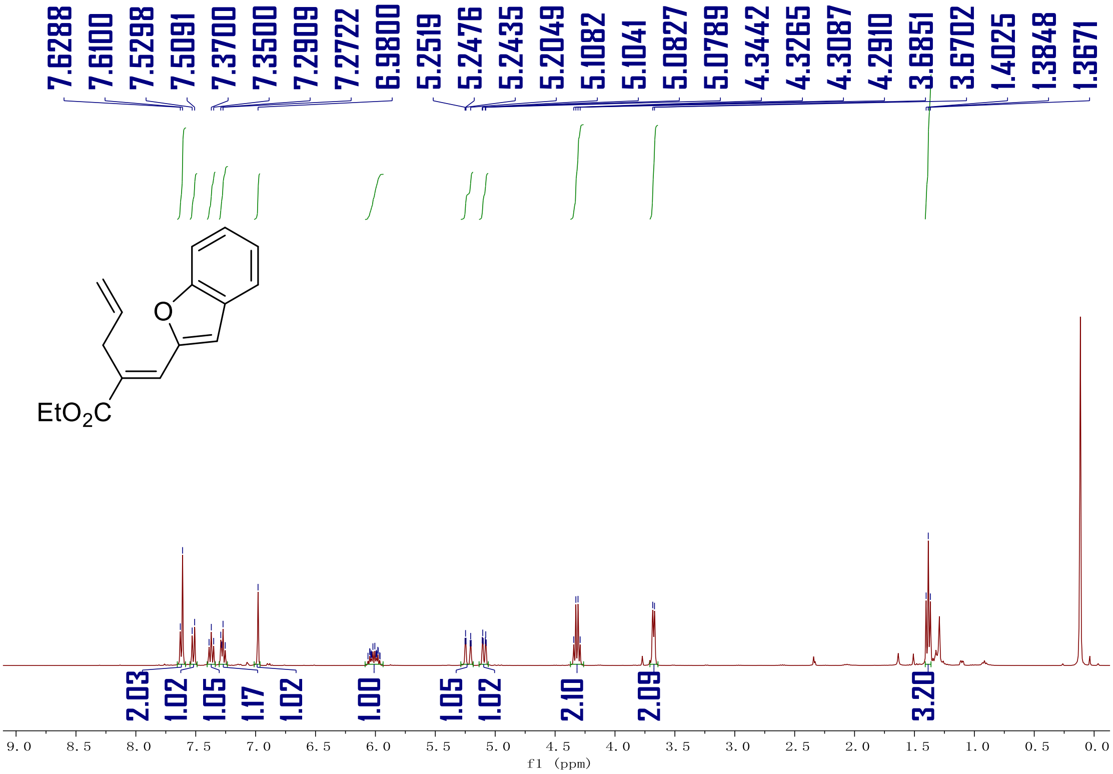

Supplementary Figure 117. ^{1}**H NMR (400 MHz,** $CDCl_3$**) of 11b**

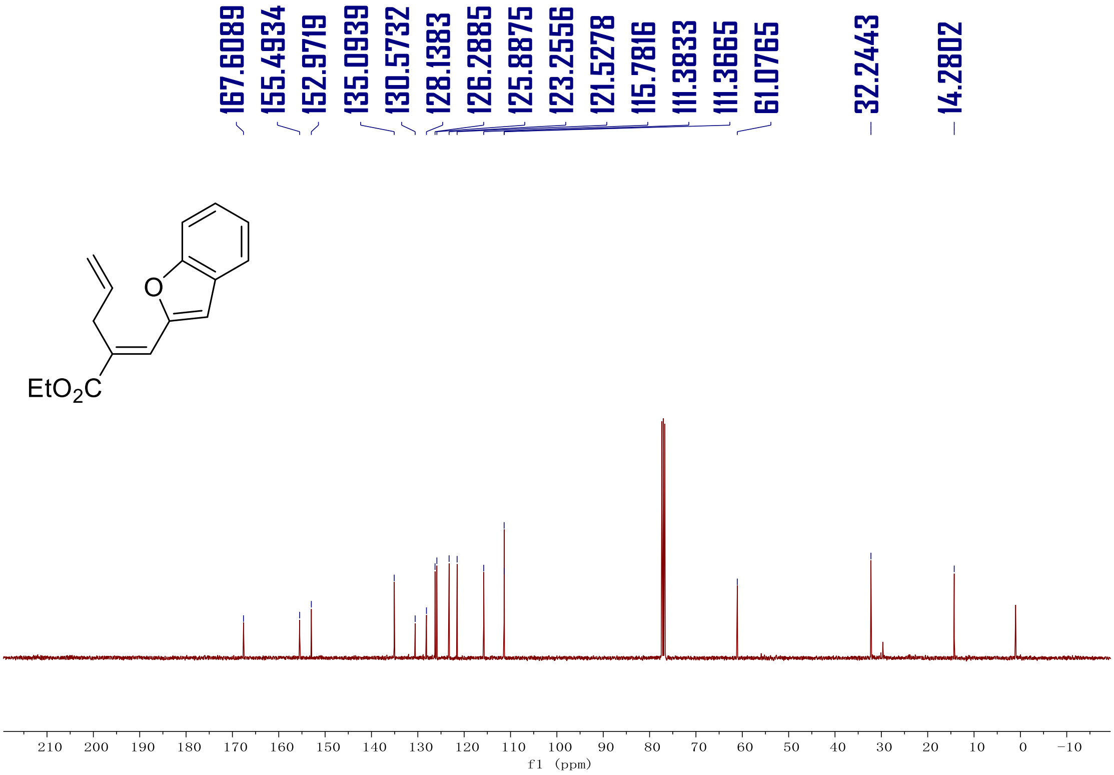

Supplementary Figure 118. ^{13}**C NMR (101 MHz,** $CDCl_3$**) of 11b**

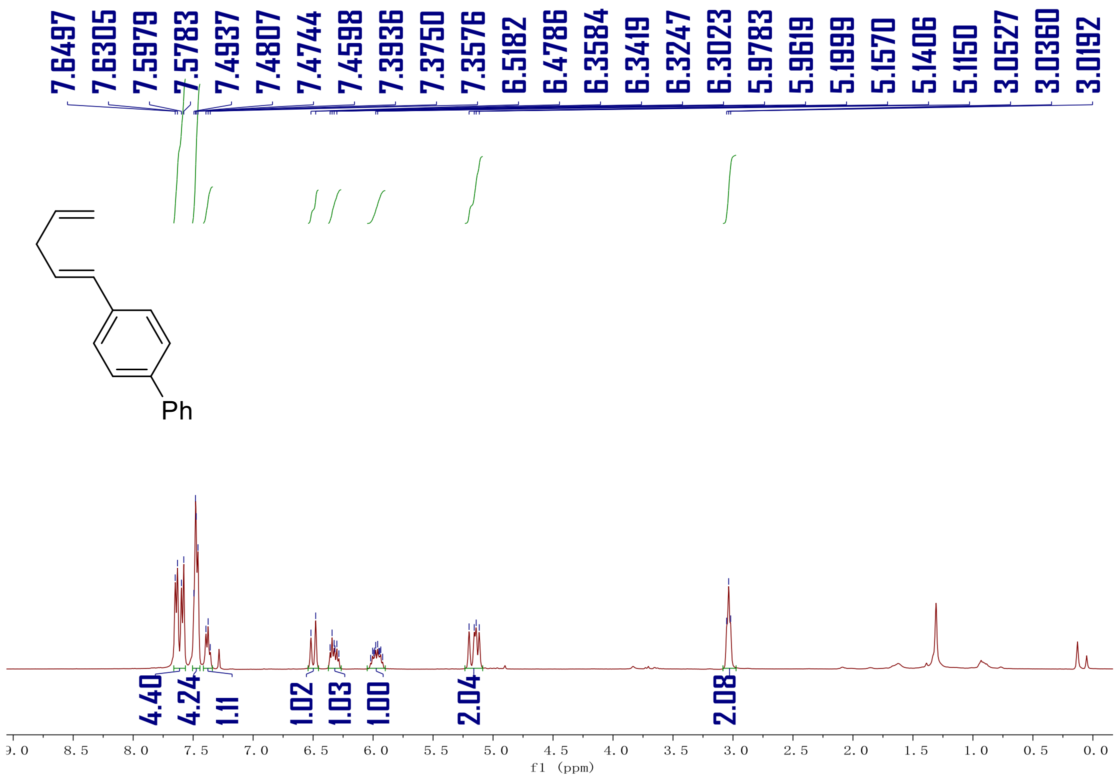

Supplementary Figure 119. ^{1}**H NMR (400 MHz, CDCl**$_3$**) of 12b**

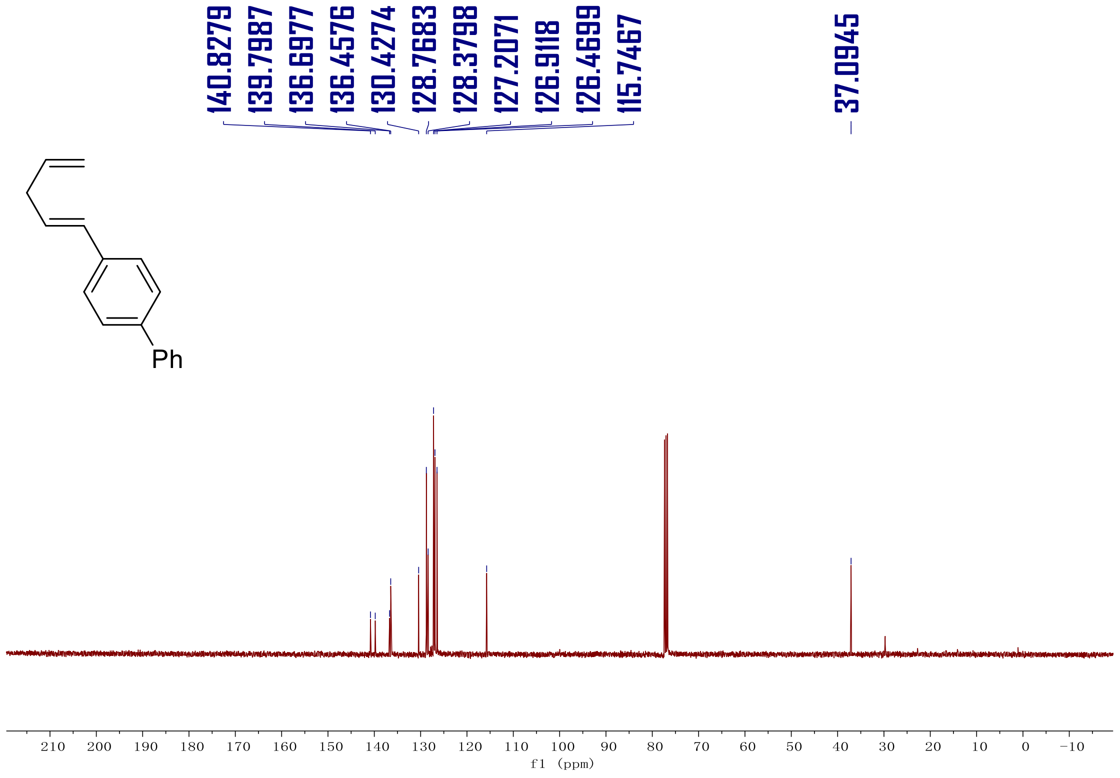

Supplementary Figure 120. ^{13}**C NMR (101 MHz, CDCl**$_3$**) of 12b**

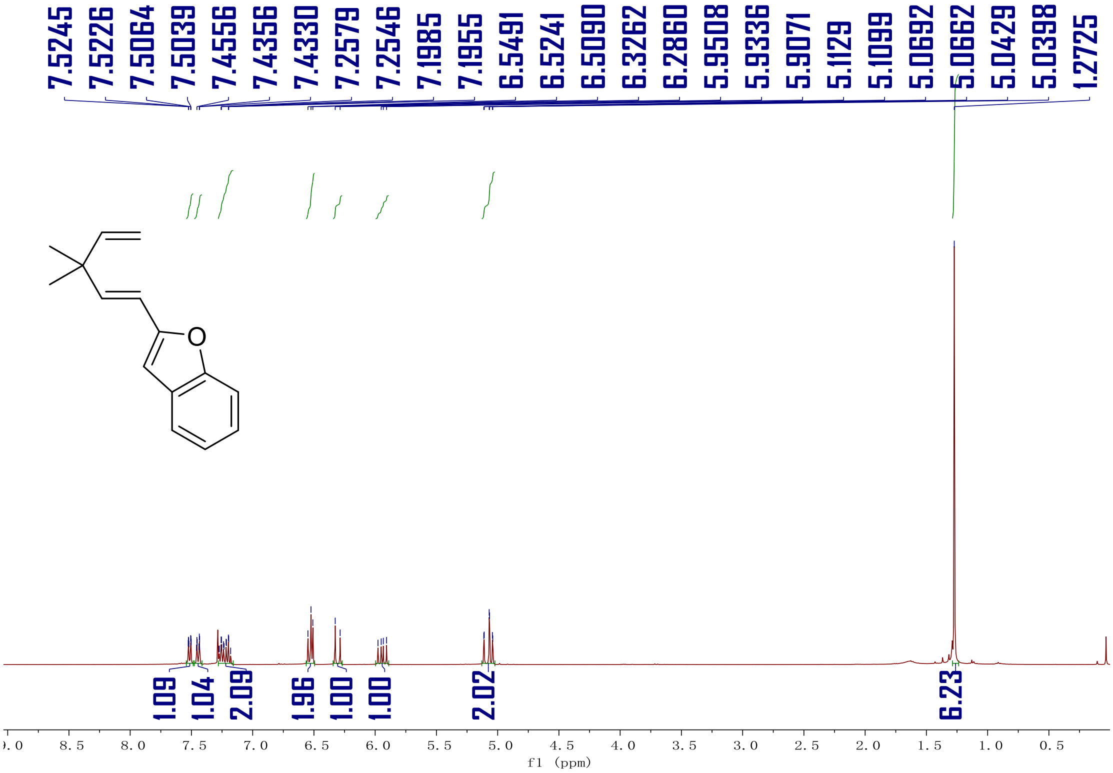

Supplementary Figure 121. ^{1}**H NMR (400 MHz, CDCl₃) of 13b**

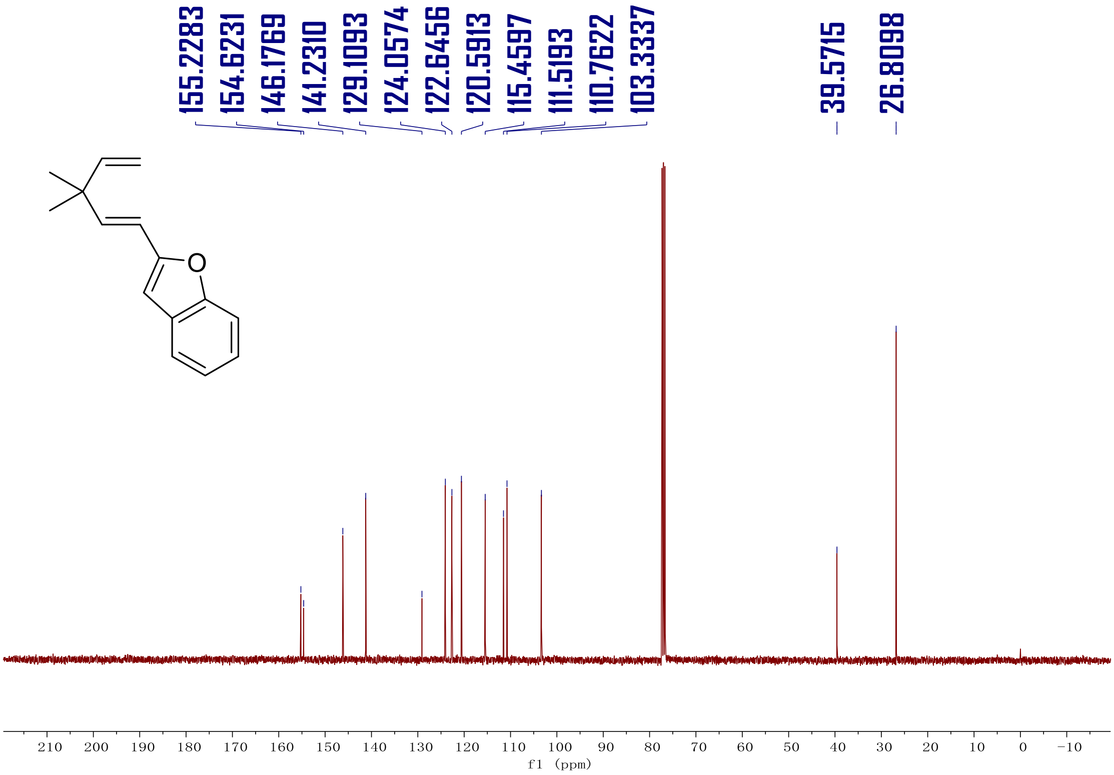

Supplementary Figure 122. ^{13}**C NMR (101 MHz, CDCl₃) of 13b**

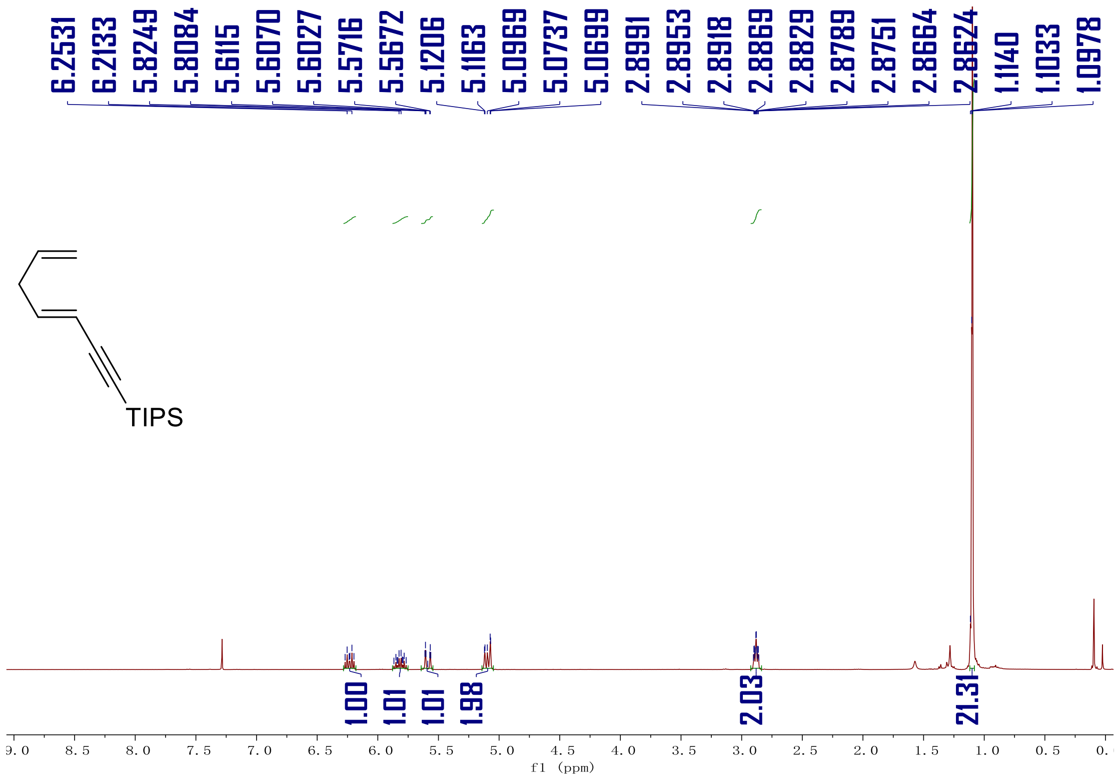

Supplementary Figure 123. ^{1}H NMR (400 MHz, CDCl$_3$) of 14b

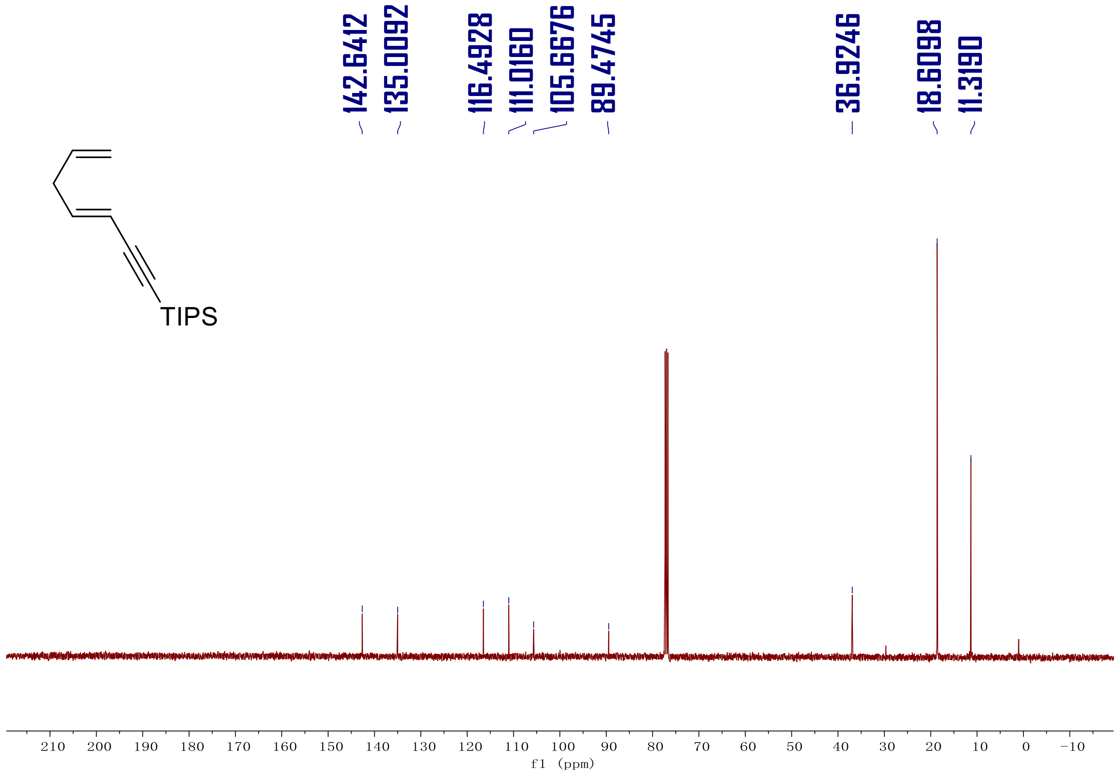

Supplementary Figure 124. ^{13}C NMR (101 MHz, CDCl$_3$) of 14b

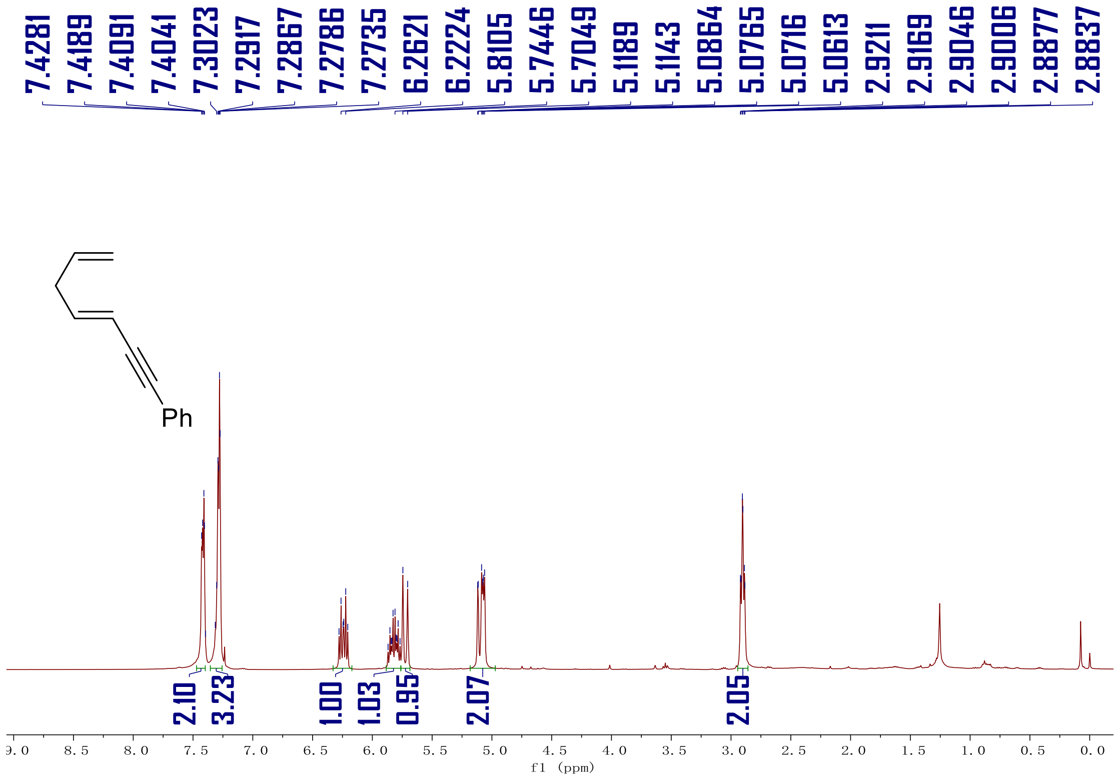

Supplementary Figure 125. ^{1}H NMR (400 MHz, $CDCl_3$) of 15b

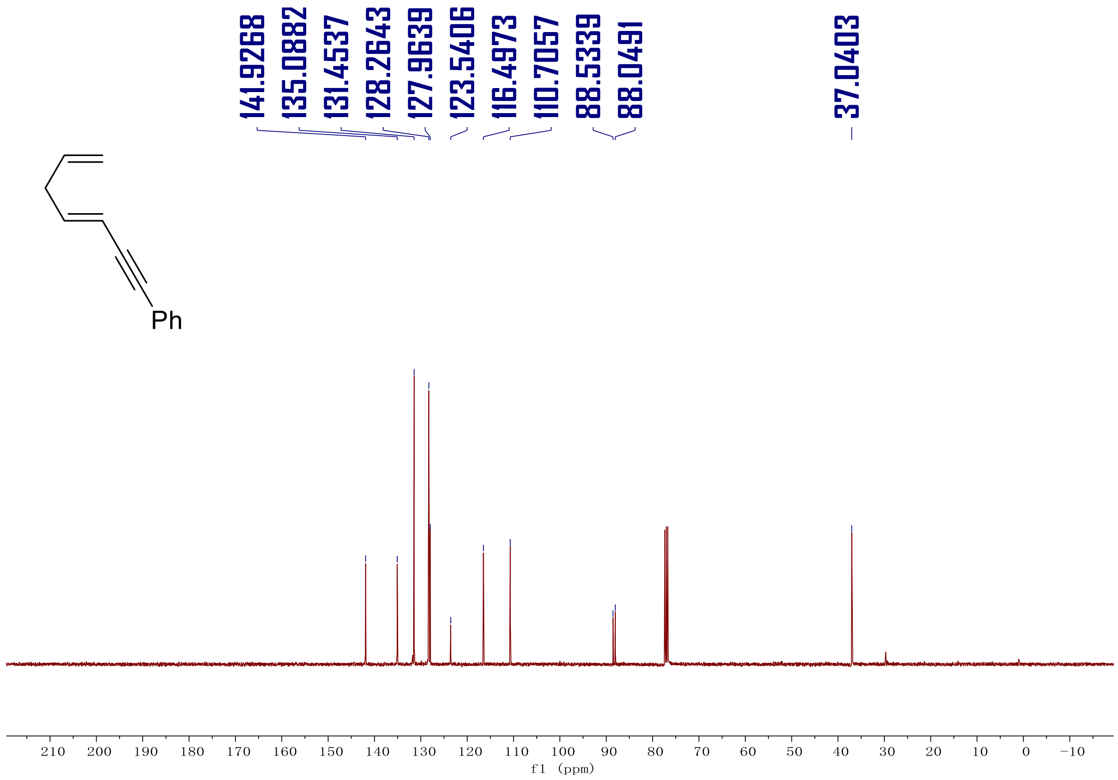

Supplementary Figure 126. ^{13}C NMR (101 MHz, $CDCl_3$) of 15b

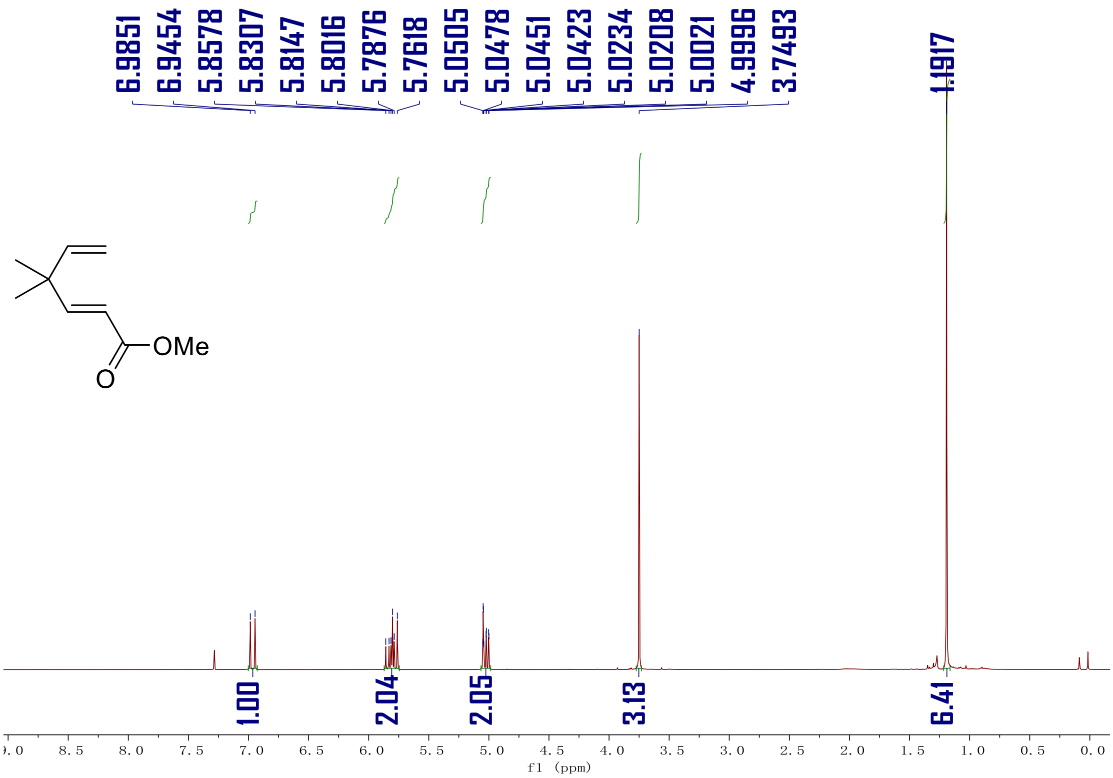

Supplementary Figure 127. ^{1}**H NMR (400 MHz, CDCl$_3$) of 16b**

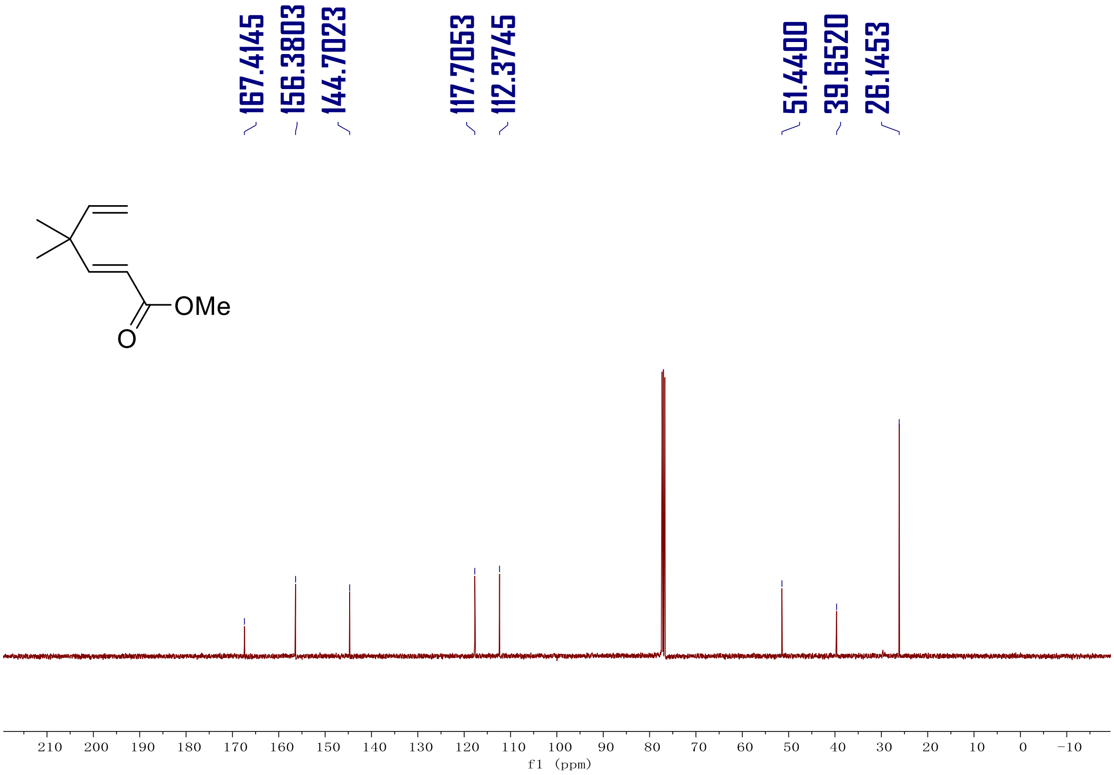

Supplementary Figure 128. ^{13}**C NMR (101 MHz, CDCl$_3$) of 16b**

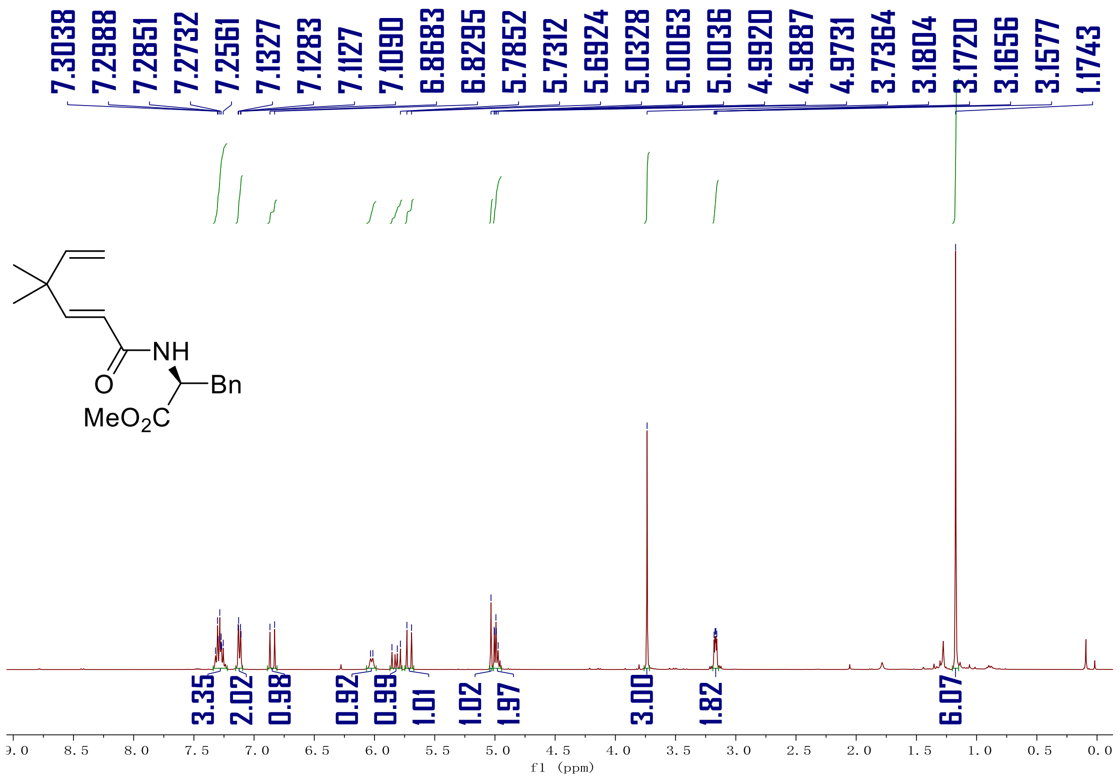

Supplementary Figure 129. **^{1}H NMR (400 MHz, $CDCl_3$) of 17b**

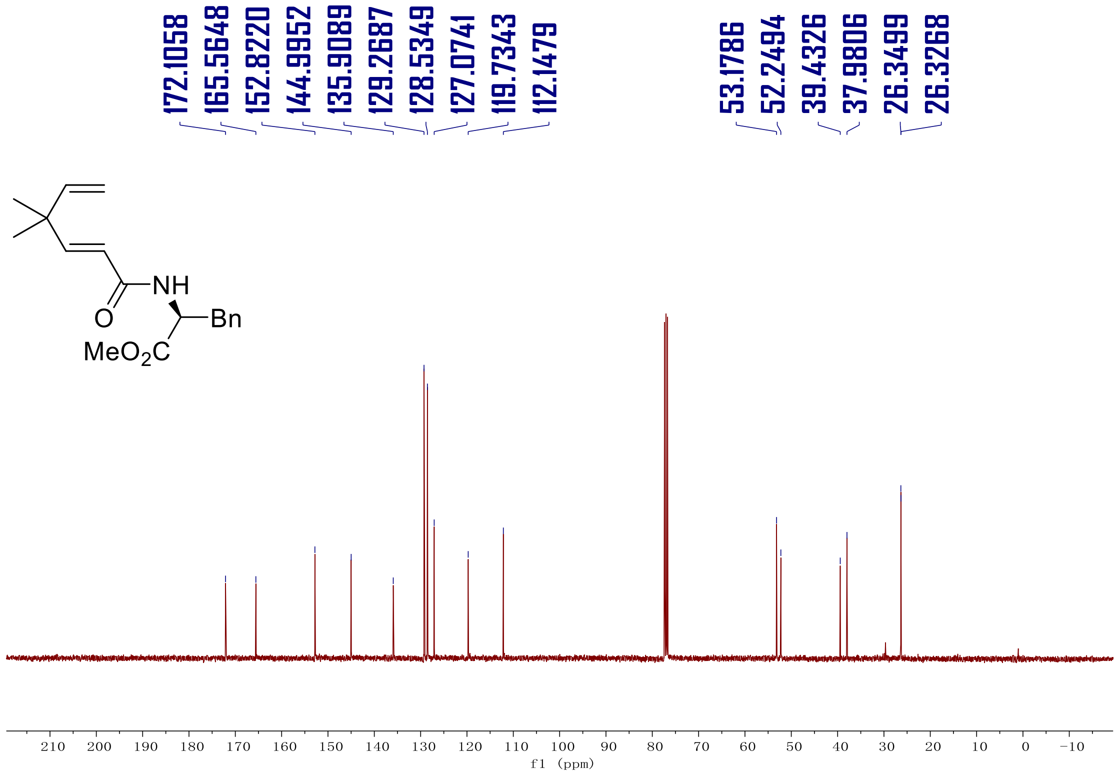

Supplementary Figure 130. **^{13}C NMR (101 MHz, $CDCl_3$) of 17b**

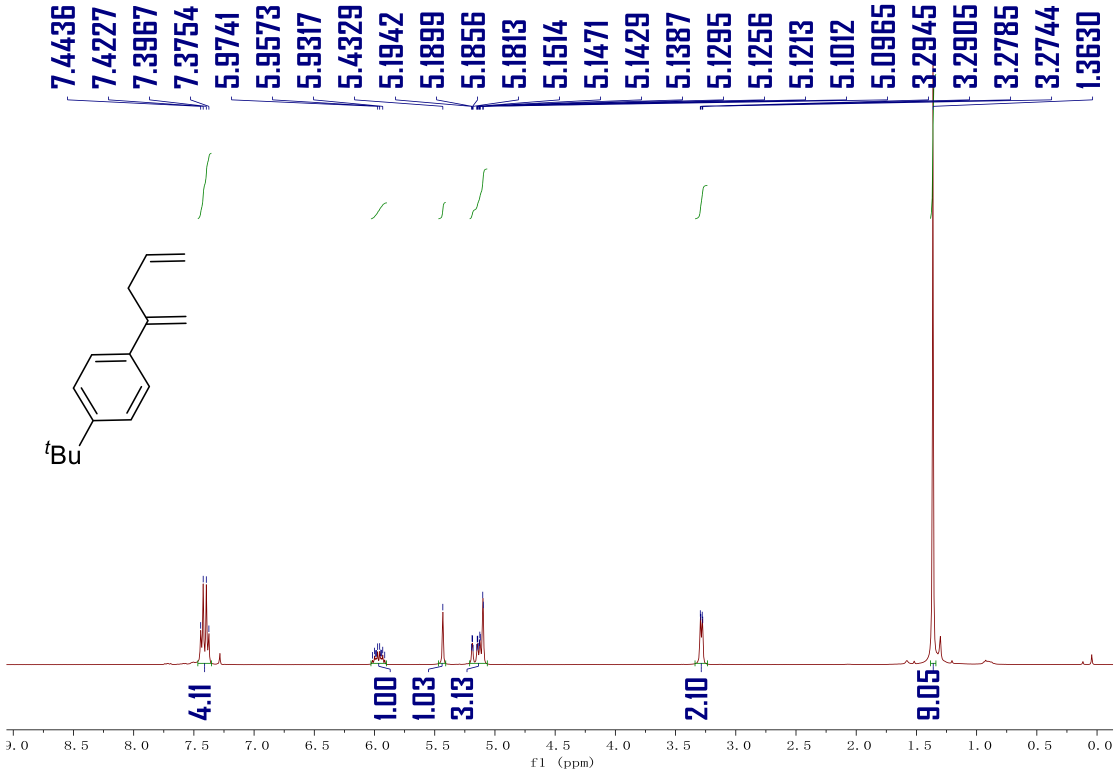

Supplementary Figure 131. ^{1}**H NMR (400 MHz, CDCl$_3$) of 18b**

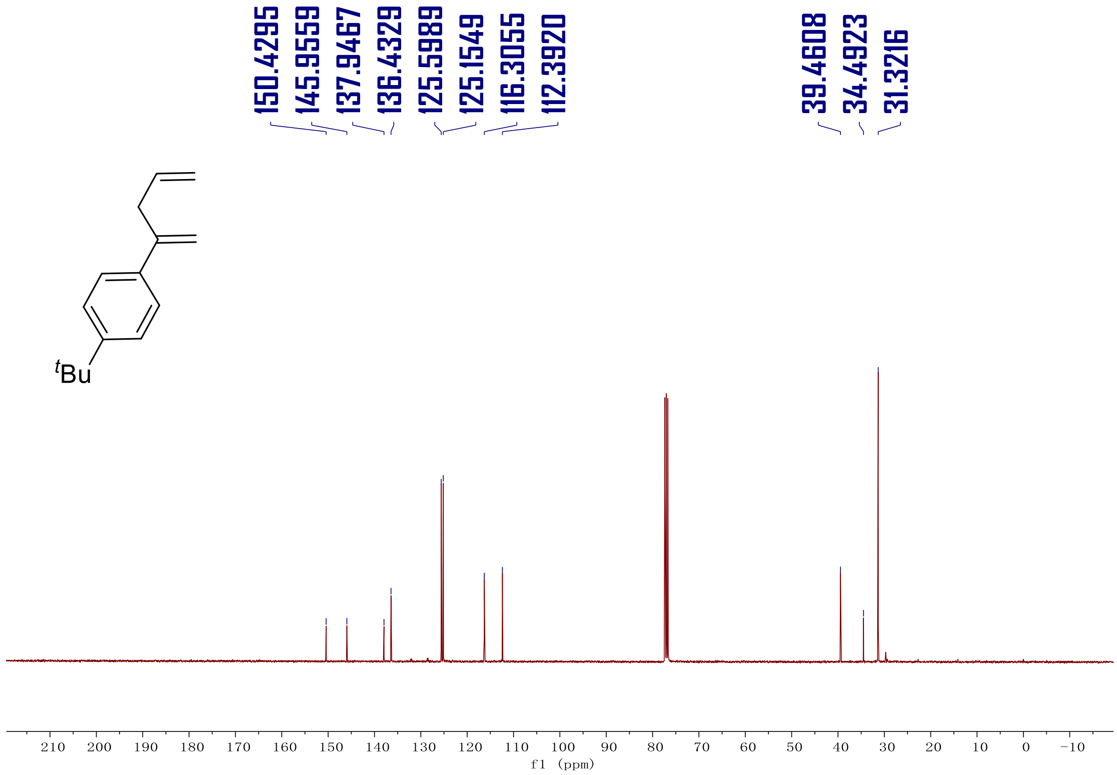

Supplementary Figure 132. ^{13}**C NMR (101 MHz, CDCl$_3$) of 18b**

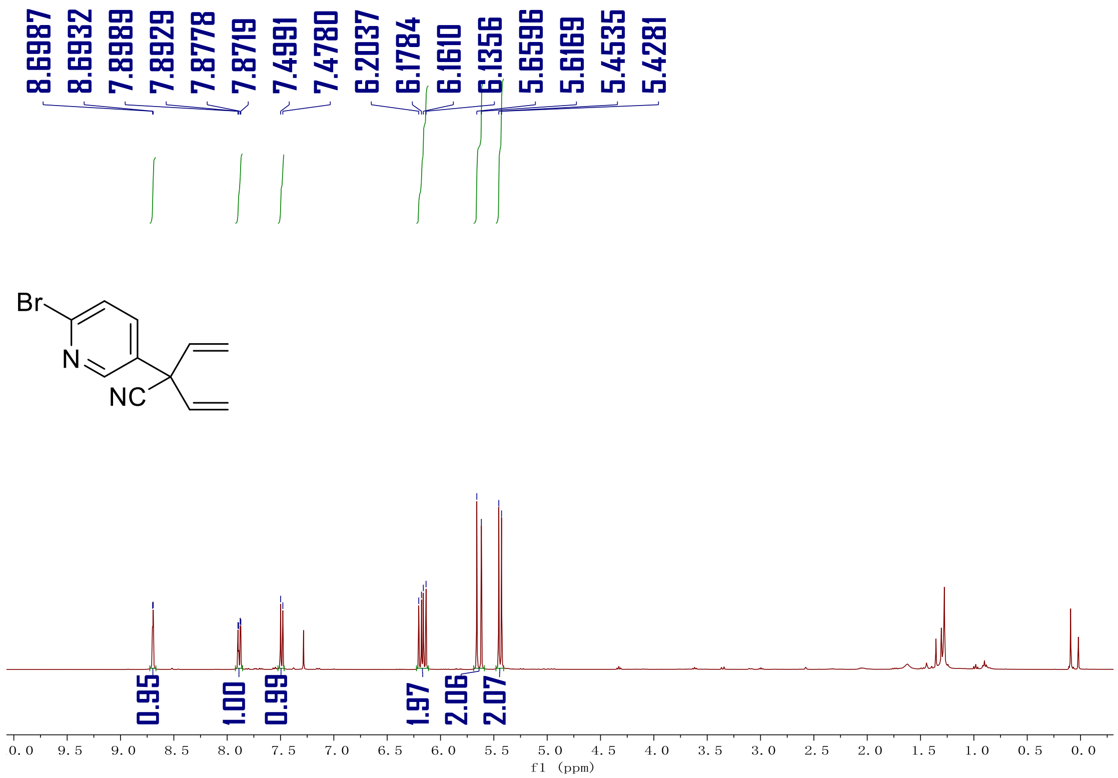

Supplementary Figure 133. ^{1}H NMR (400 MHz, $CDCl_3$) of 19b

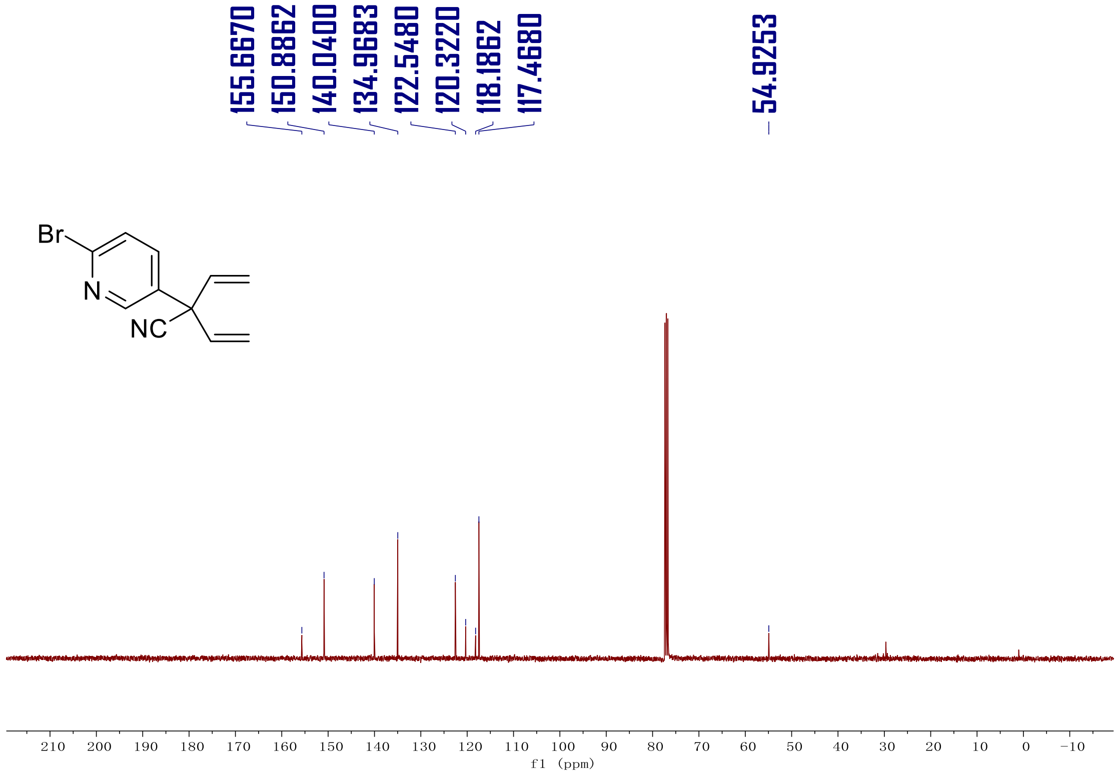

Supplementary Figure 134. ^{13}C NMR (101 MHz, $CDCl_3$) of 19b

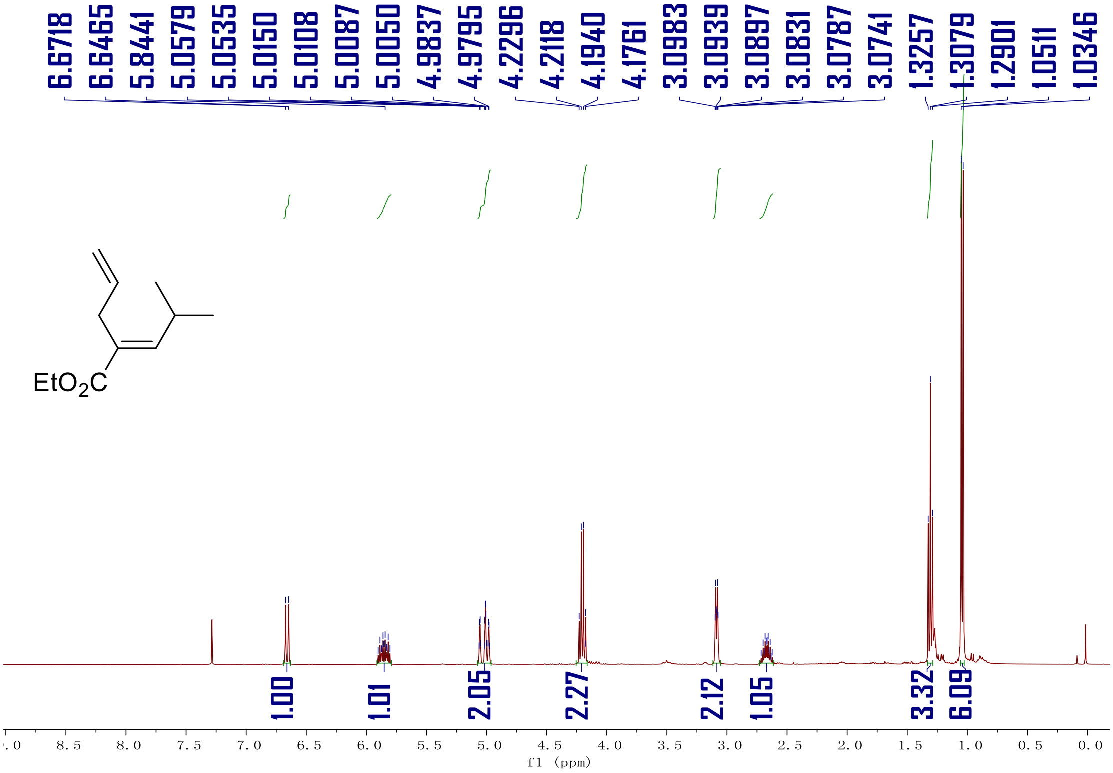

Supplementary Figure 135. ^{1}**H NMR (400 MHz, CDCl**$_3$**) of 20b**

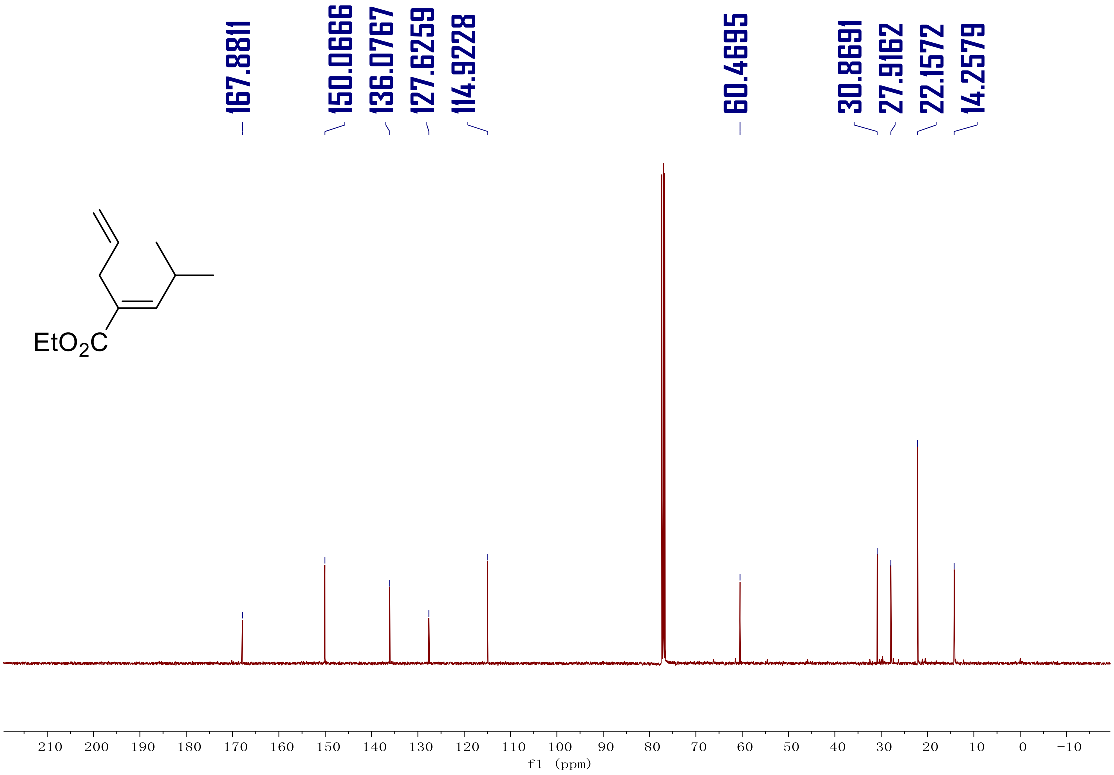

Supplementary Figure 136. ^{13}**C NMR (101 MHz, CDCl**$_3$**) of 20b**

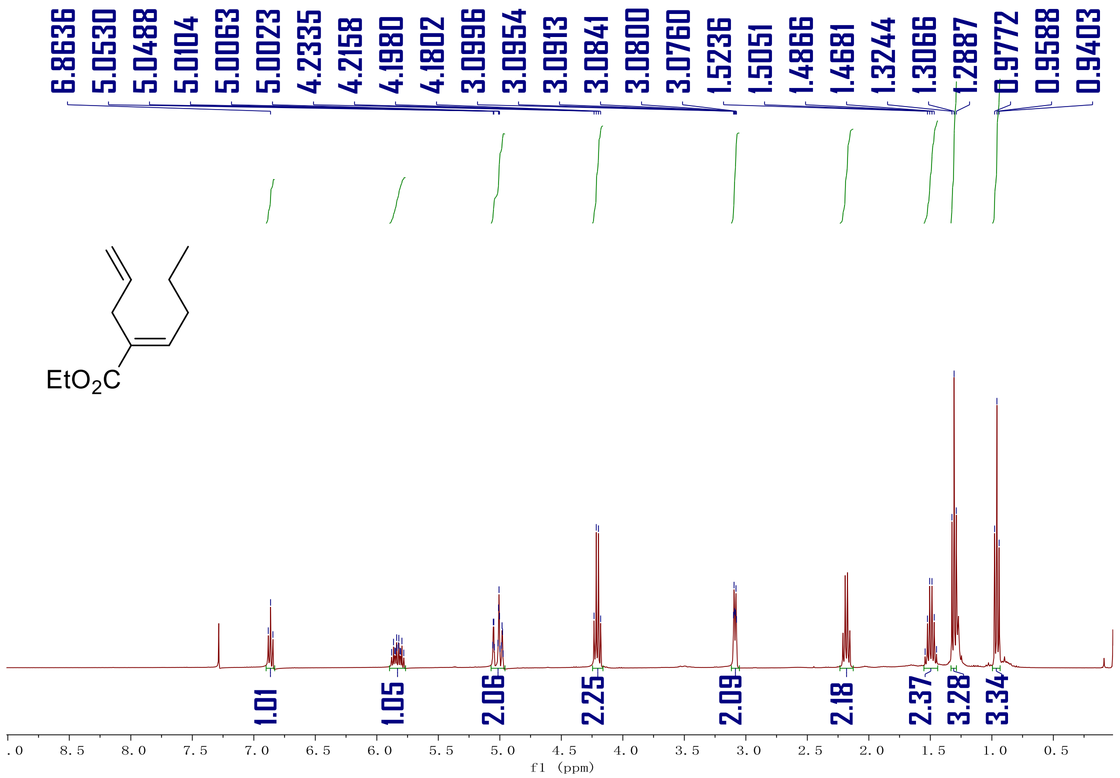

Supplementary Figure 137. ^{1}H NMR (400 MHz, CDCl$_3$) of 21b

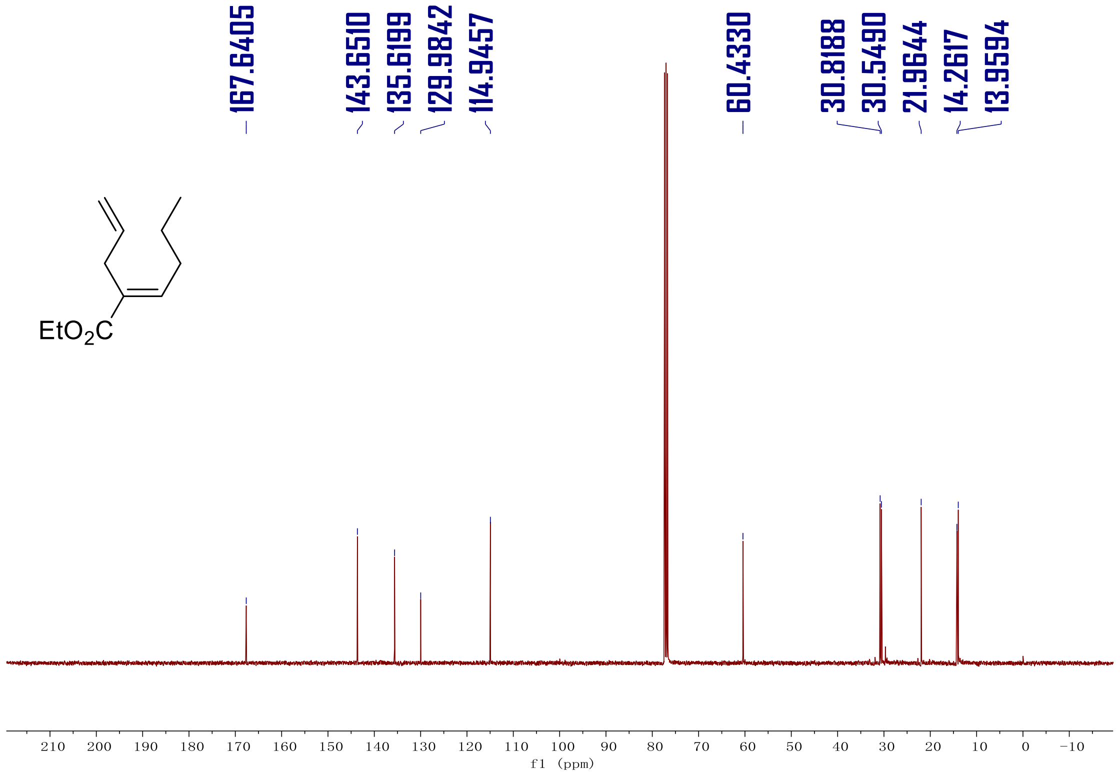

Supplementary Figure 138. ^{13}C NMR (101 MHz, CDCl$_3$) of 21b

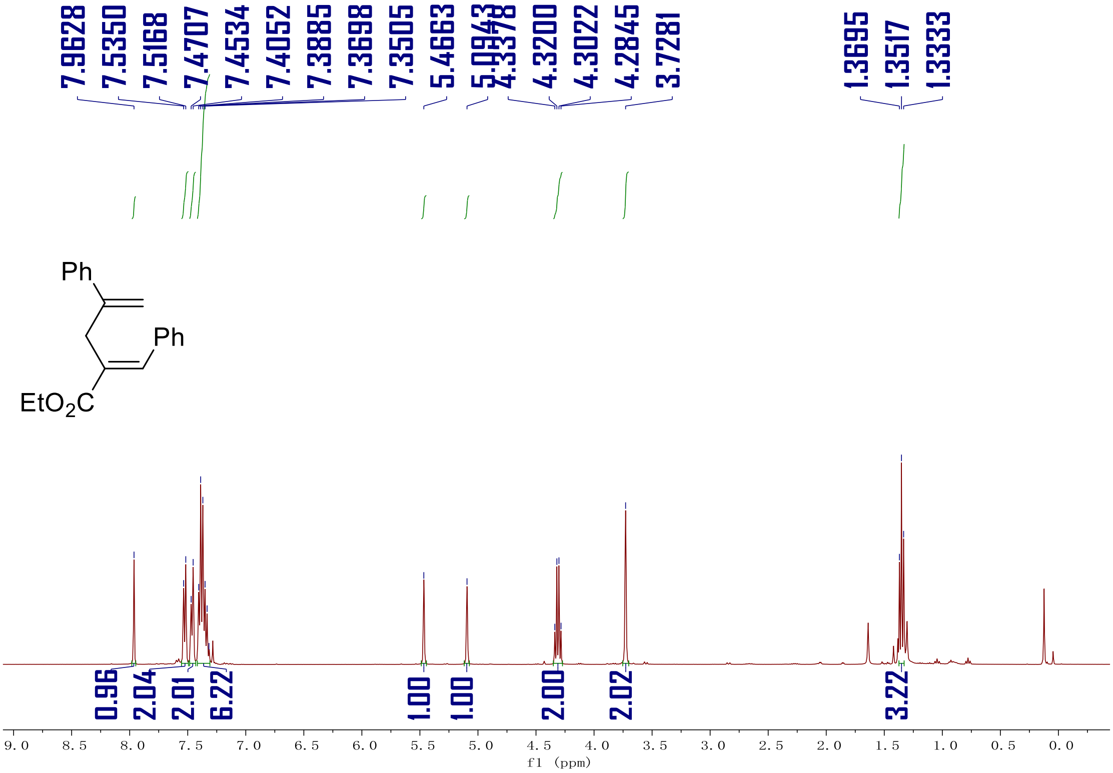

Supplementary Figure 139. ^{1}H NMR (400 MHz, $CDCl_3$) of 22b

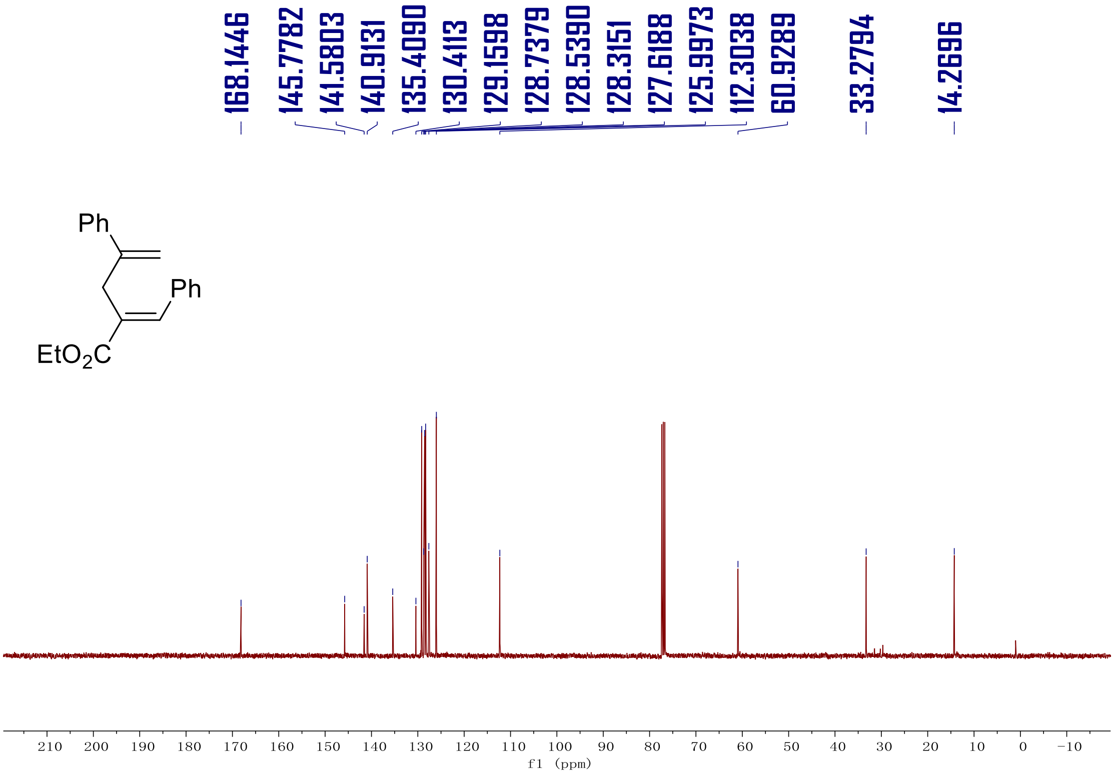

Supplementary Figure 140. ^{13}C NMR (101 MHz, $CDCl_3$) of 22b

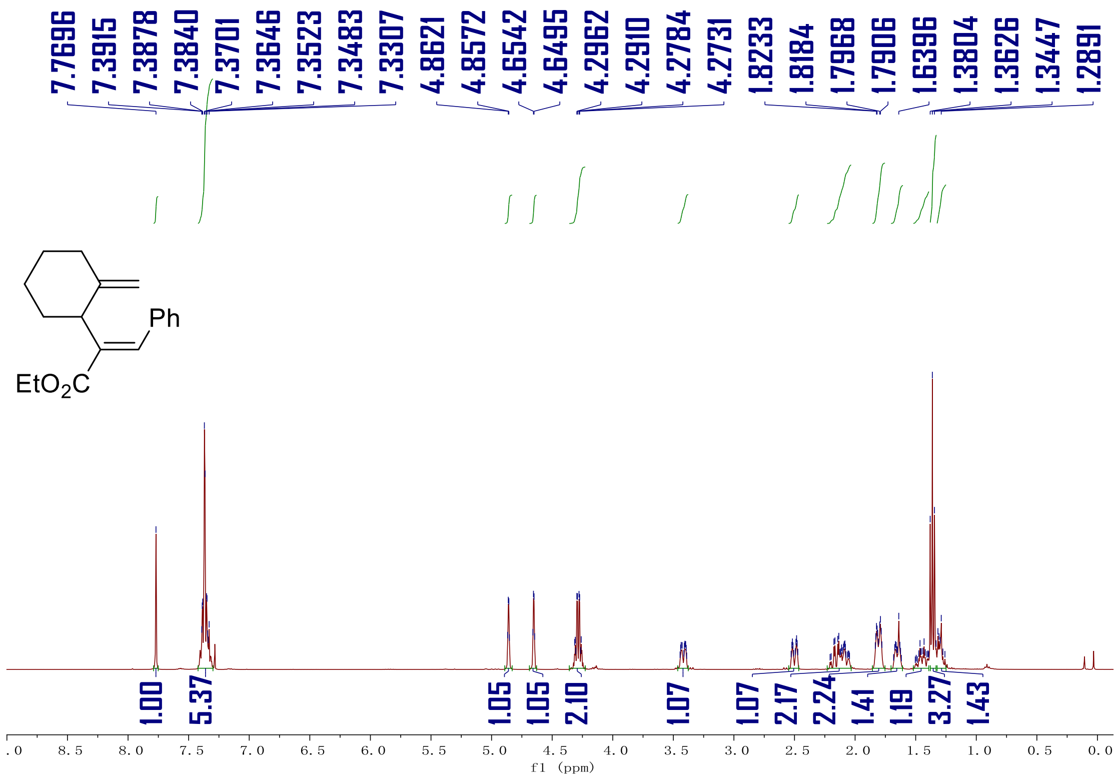

Supplementary Figure 141. ^{1}H NMR (400 MHz, CDCl₃) of 23b

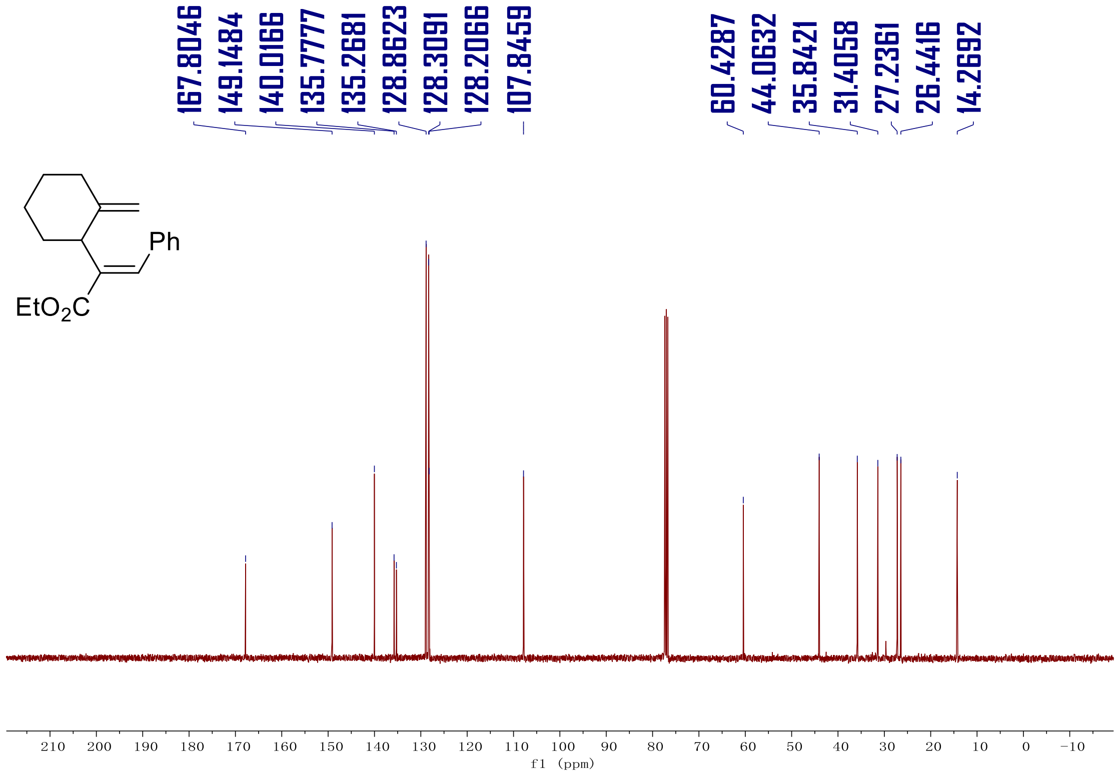

Supplementary Figure 142. ^{13}C NMR (101 MHz, CDCl₃) of 23b

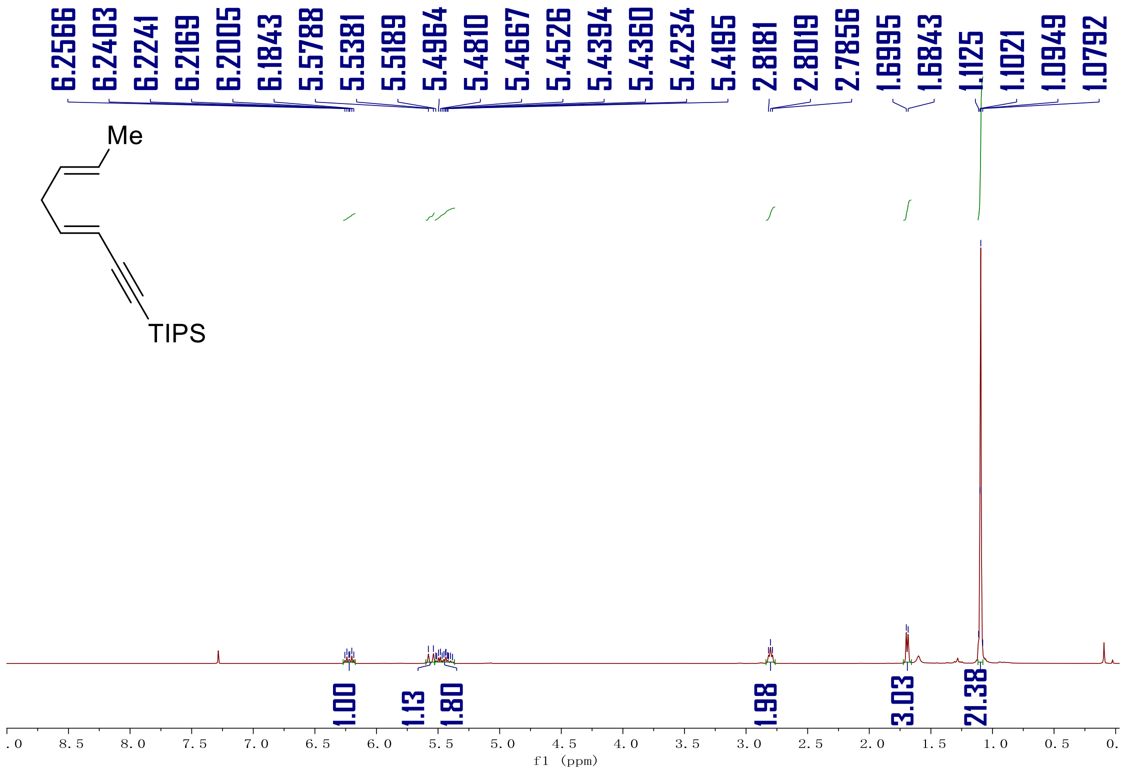

Supplementary Figure 143. ^{1}H NMR (400 MHz, CDCl3) of 24b

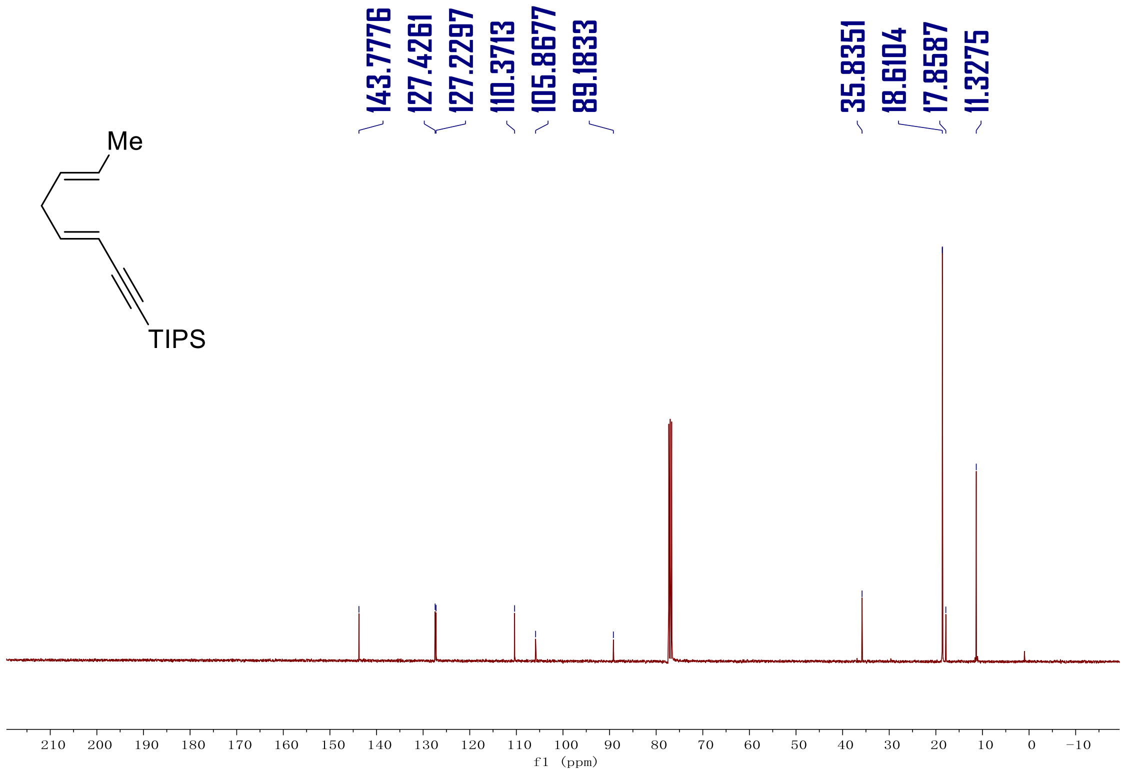

Supplementary Figure 144. ^{13}C NMR (101 MHz, CDCl3) of 24b

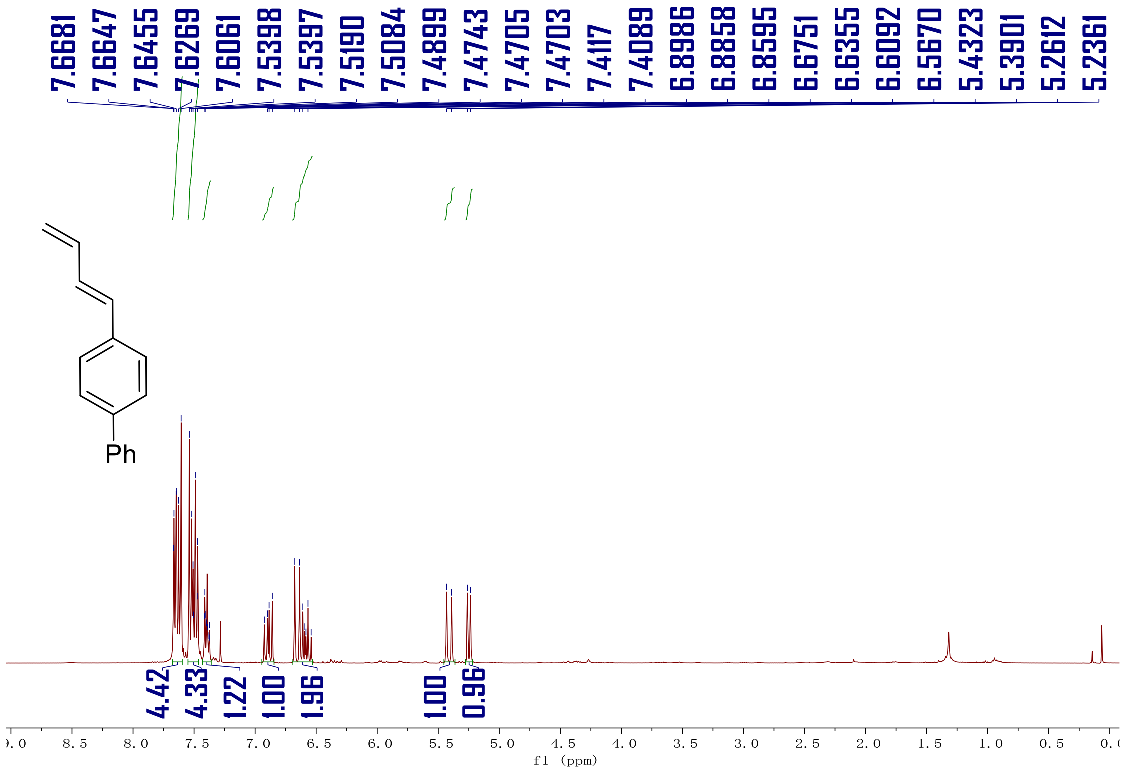

Supplementary Figure 145. ^{1}H NMR (400 MHz, CDCl$_3$) of 25b

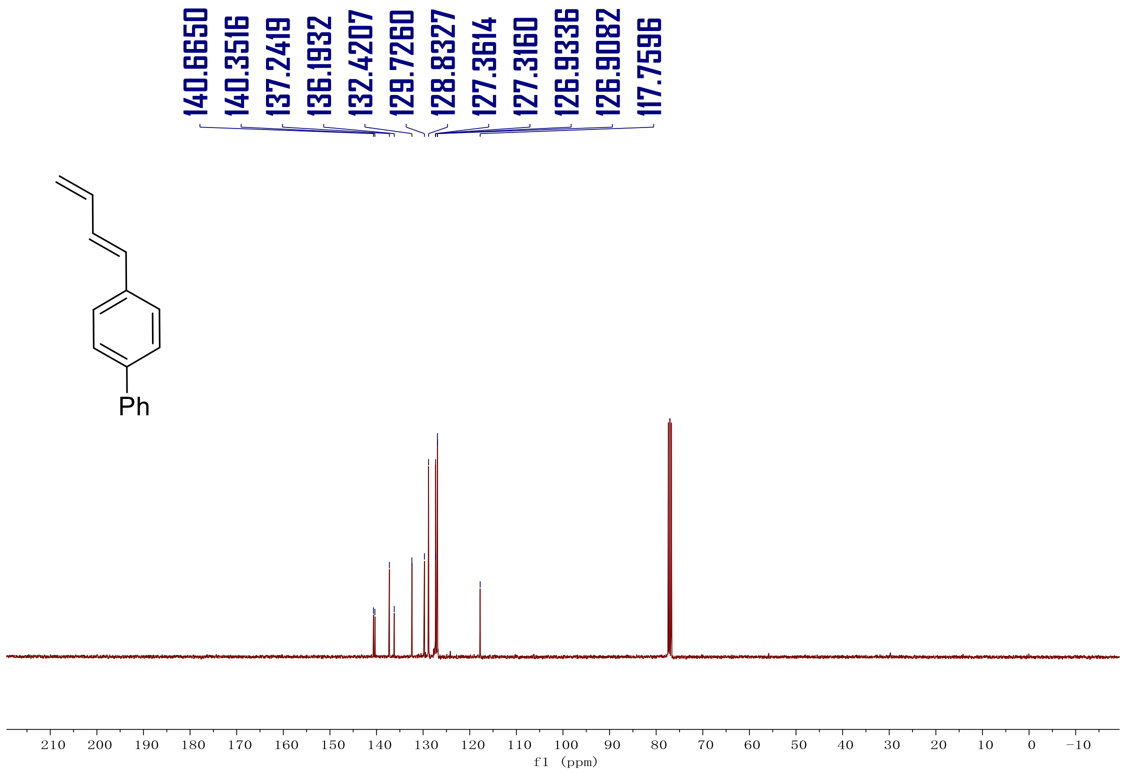

Supplementary Figure 146. ^{13}C NMR (101 MHz, CDCl$_3$) of 25b

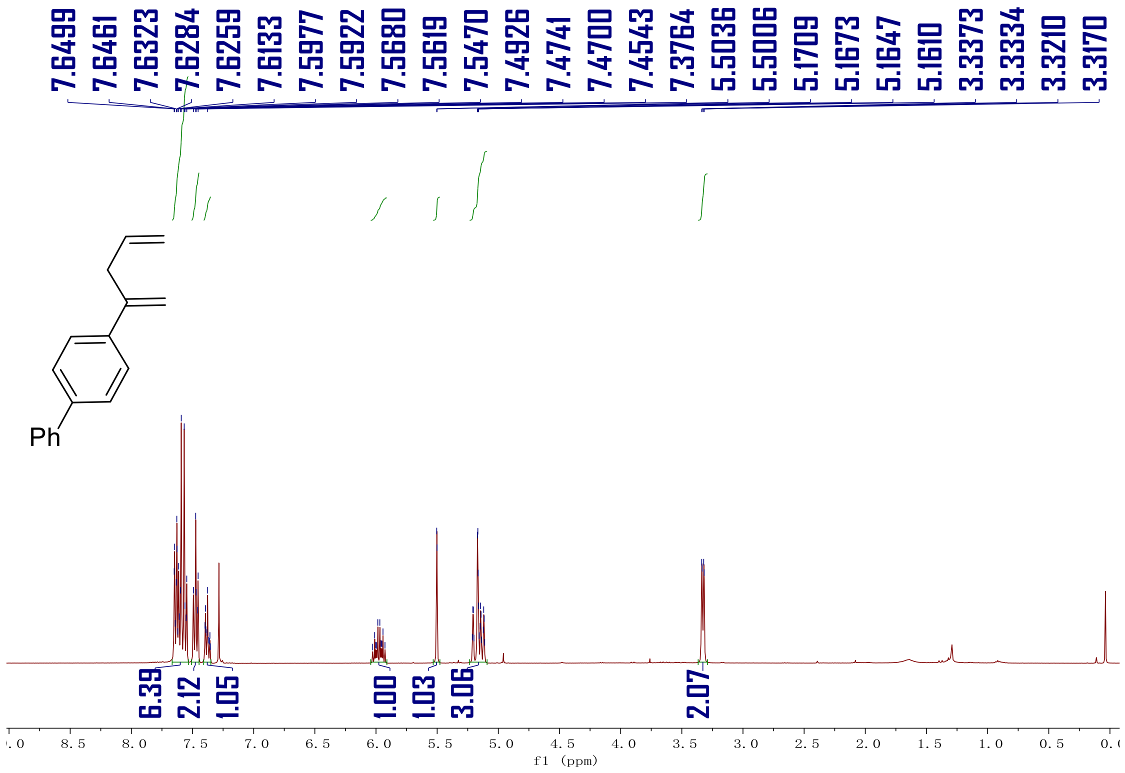

Supplementary Figure 147. ^{1}H NMR (400 MHz, CDCl$_3$) of 26b

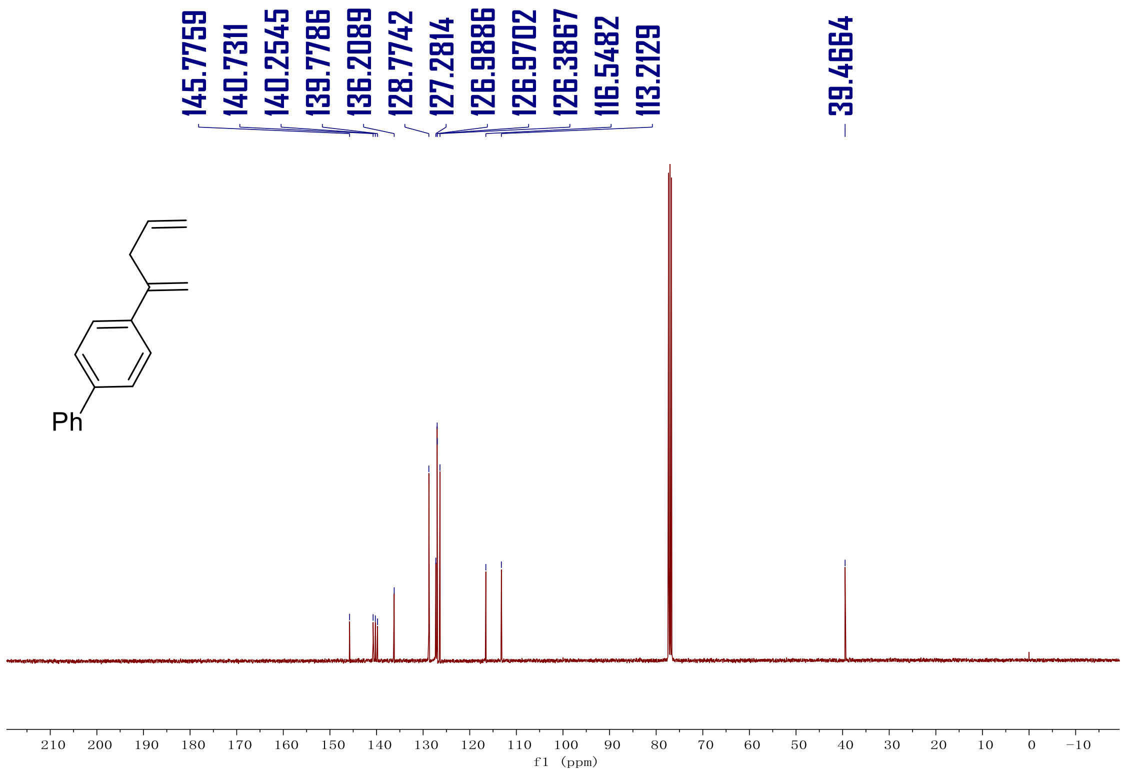

Supplementary Figure 148. ^{13}C NMR (101 MHz, CDCl$_3$) of 26b

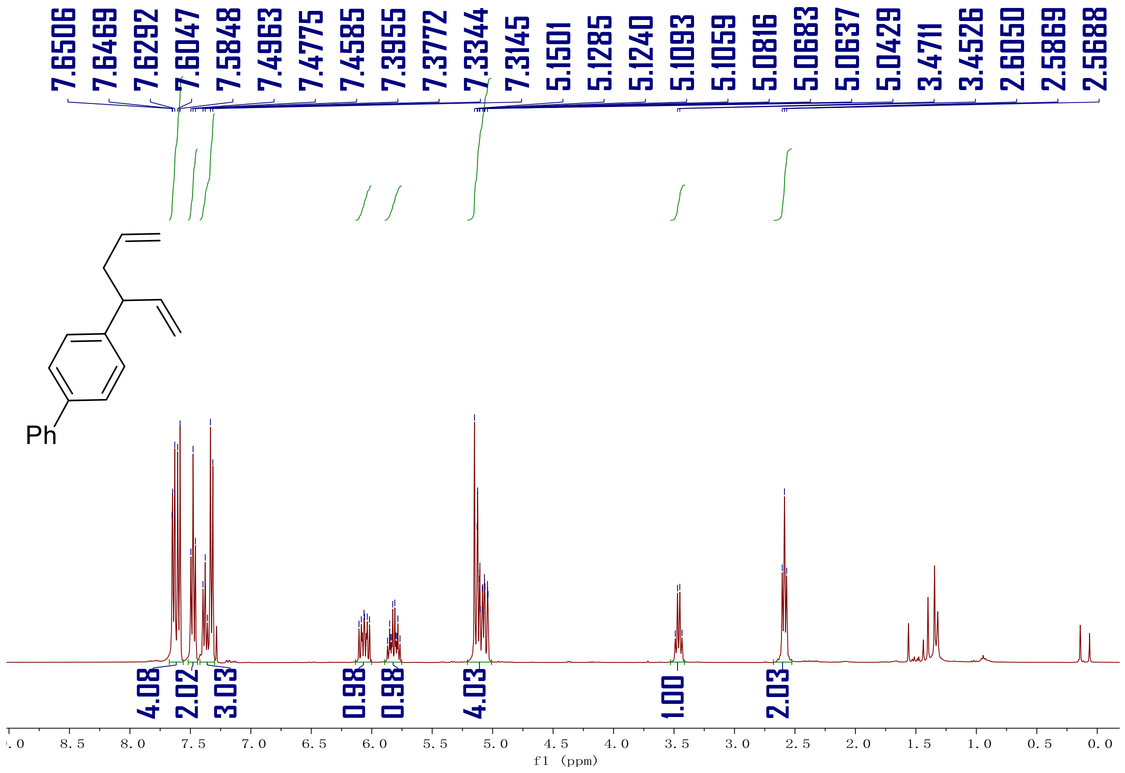

Supplementary Figure 149. ^{1}H NMR (400 MHz, $CDCl_3$) of 27b

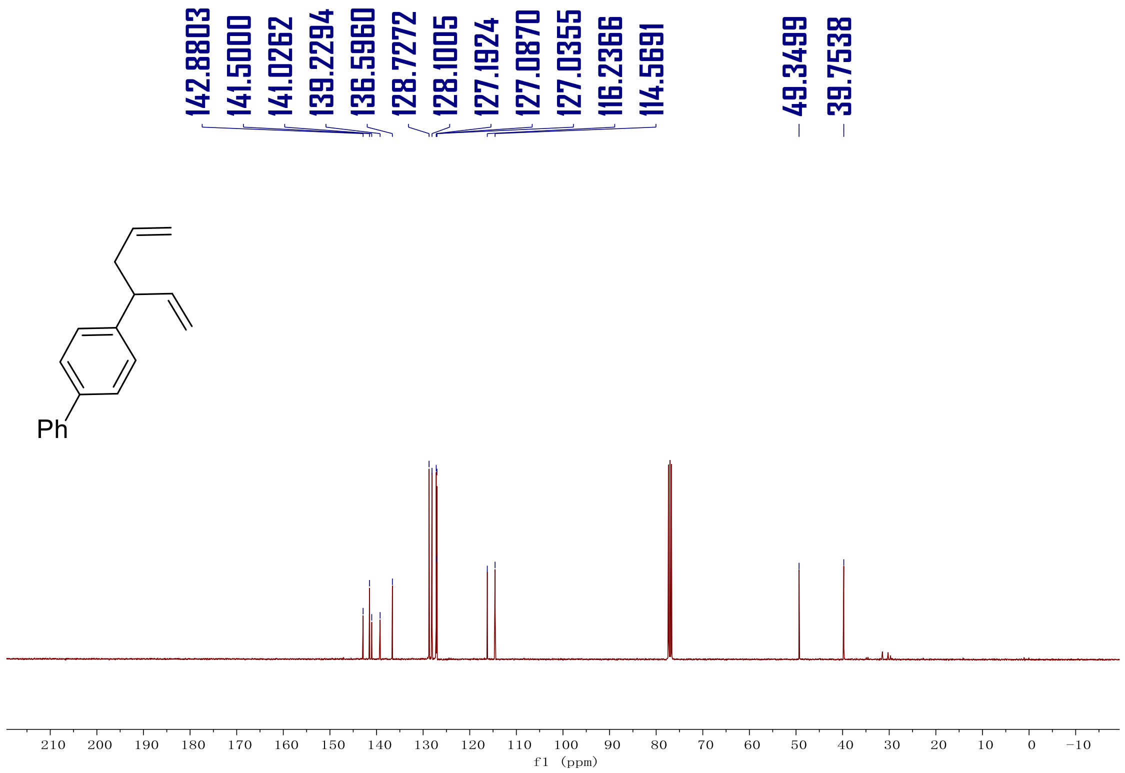

Supplementary Figure 150. ^{13}C NMR (101 MHz, $CDCl_3$) of 27b

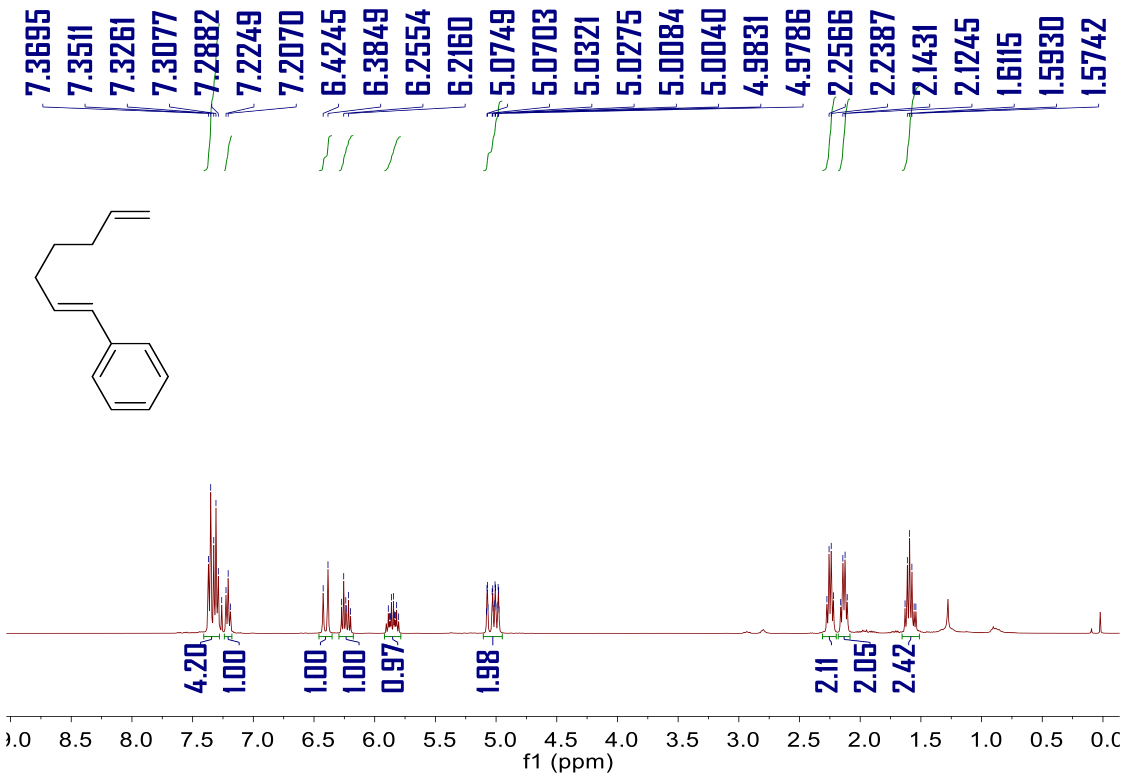

Supplementary Figure 151. ^{1}H NMR (400 MHz, $CDCl_3$) of 28b

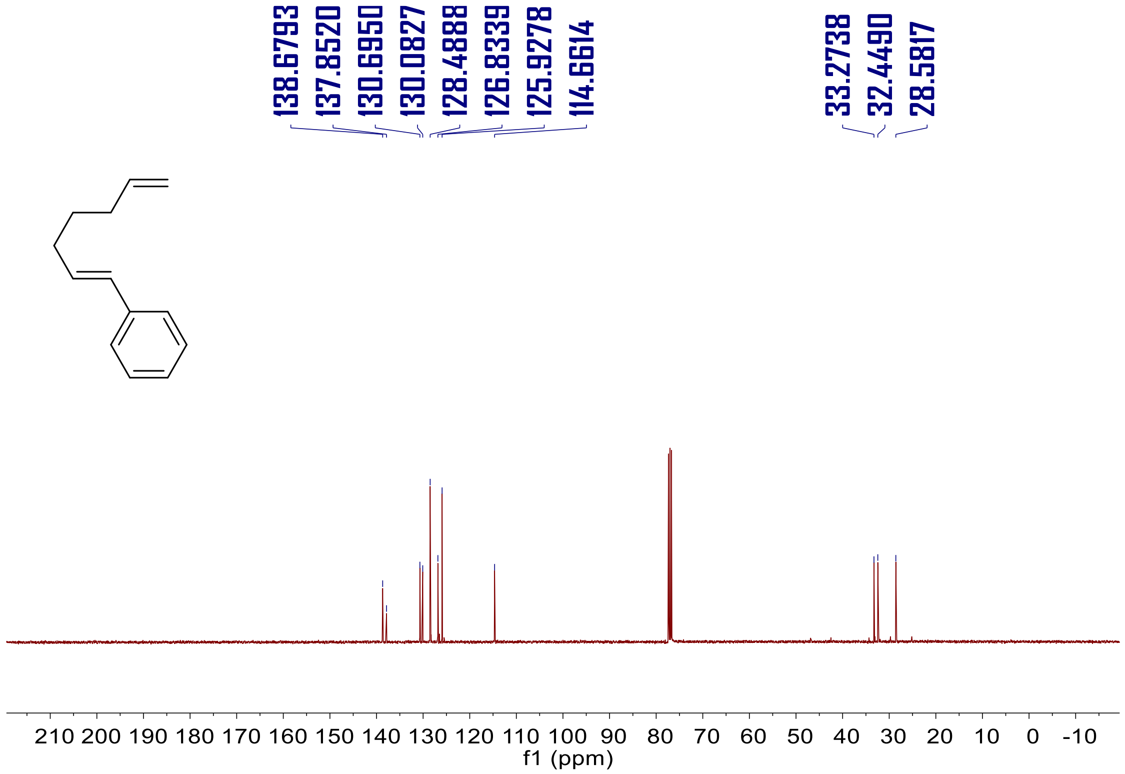

Supplementary Figure 152. ^{13}C NMR (101 MHz, $CDCl_3$) of 28b

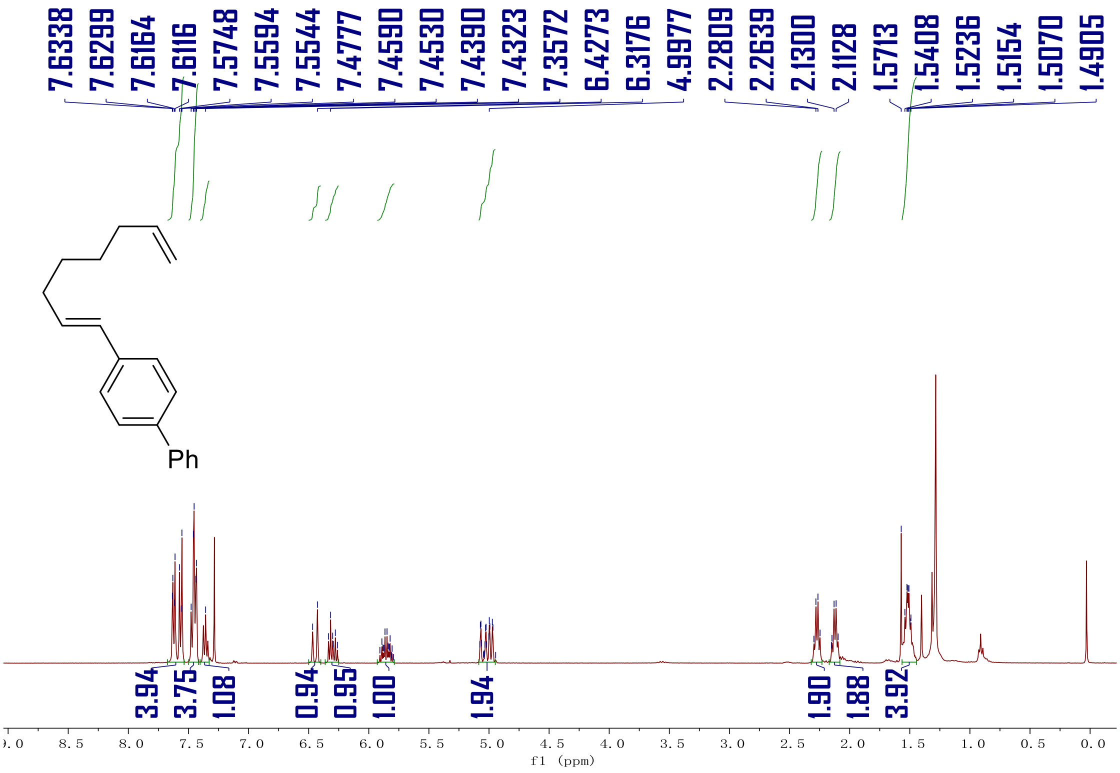

Supplementary Figure 153. ^{1}**H NMR (400 MHz, CDCl$_3$) of 29**

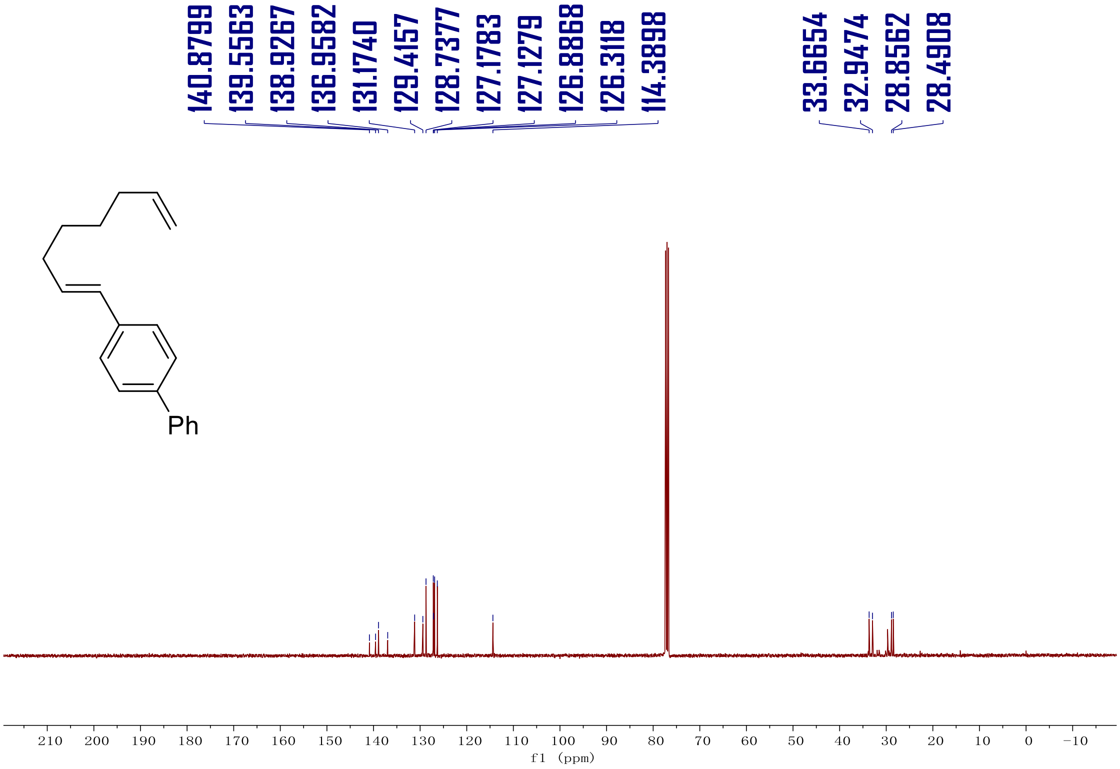

Supplementary Figure 154. ^{13}**C NMR (101 MHz, CDCl$_3$) of 29b**

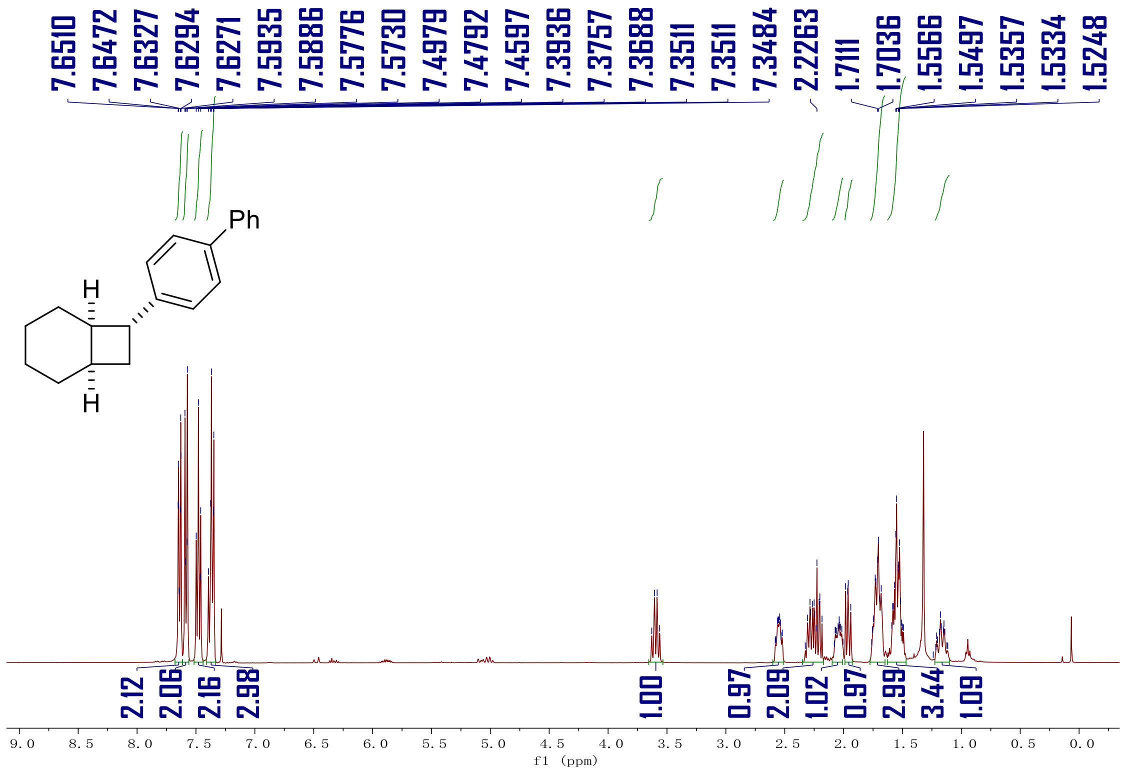

Supplementary Figure 155. ^{1}H NMR (400 MHz, $CDCl_3$) of 29c

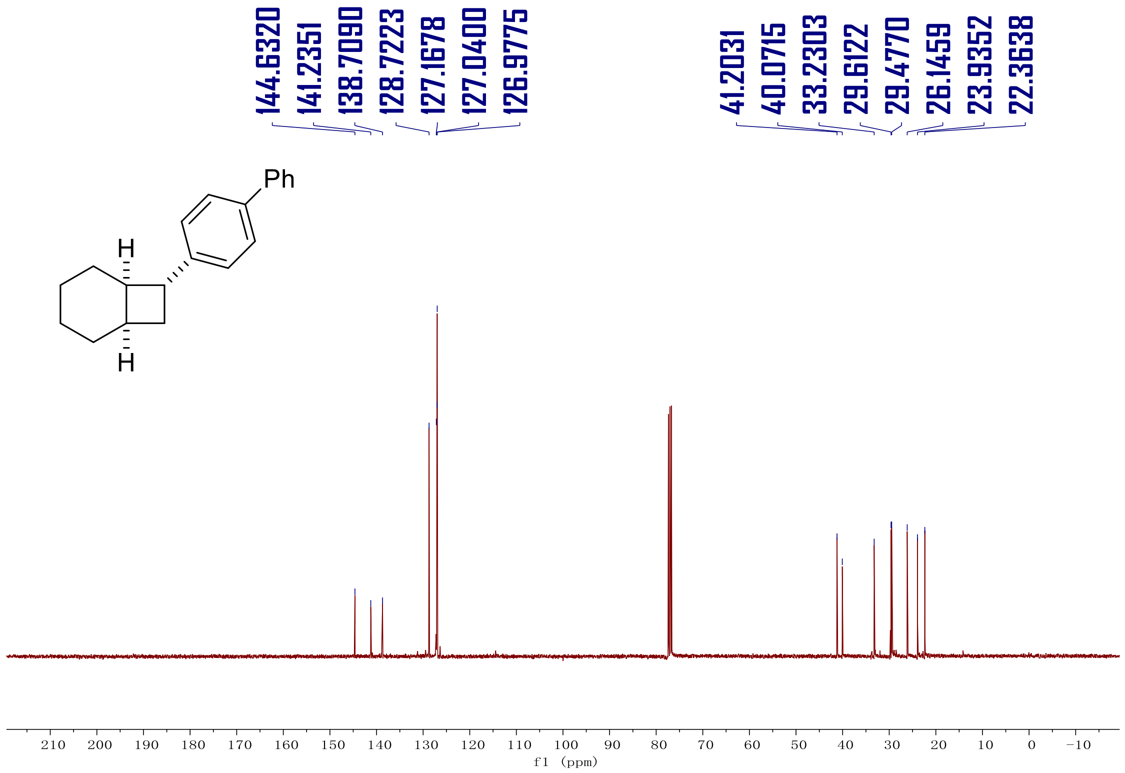

Supplementary Figure 156. ^{13}C NMR (101 MHz, $CDCl_3$) of 29c

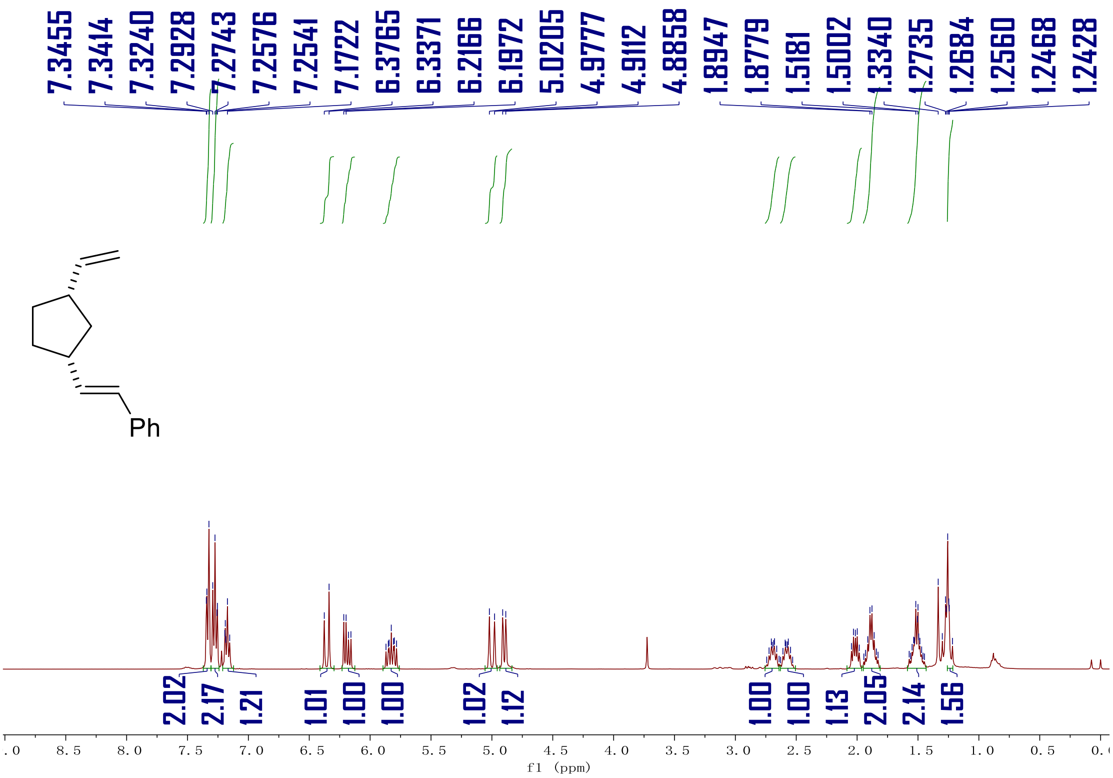

Supplementary Figure 157. ^{1}H NMR (400 MHz, $CDCl_3$) of 30b

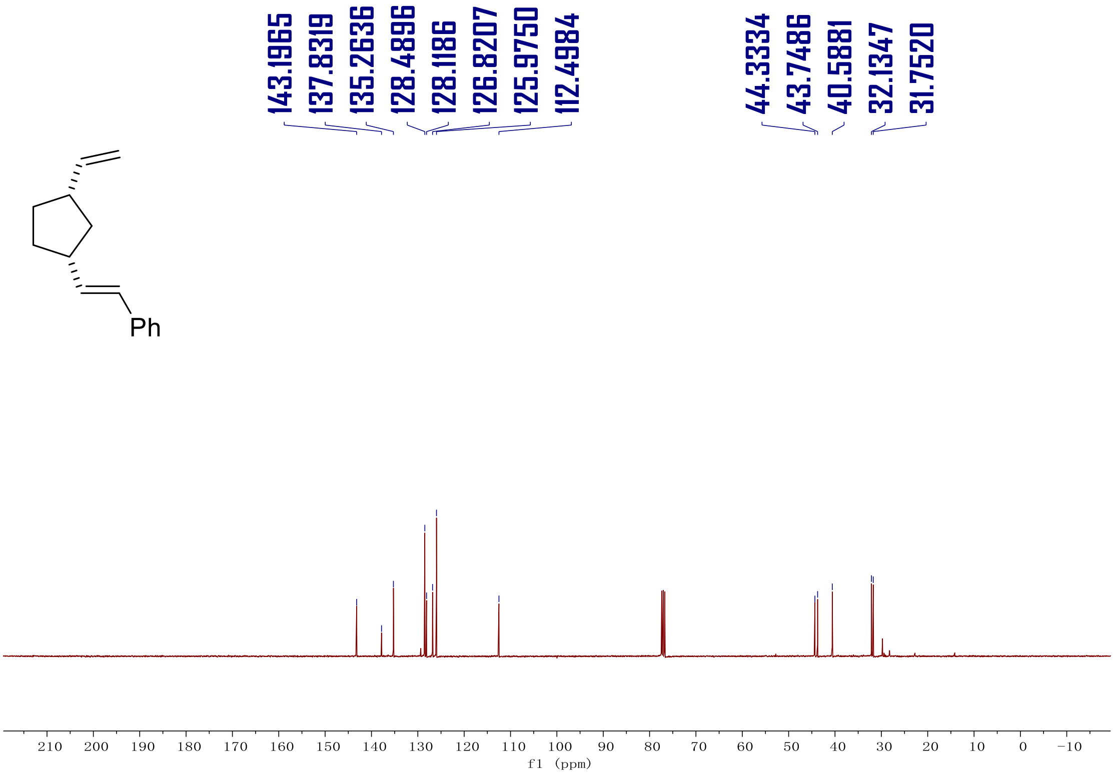

Supplementary Figure 158. ^{13}C NMR (101 MHz, $CDCl_3$) of 30b

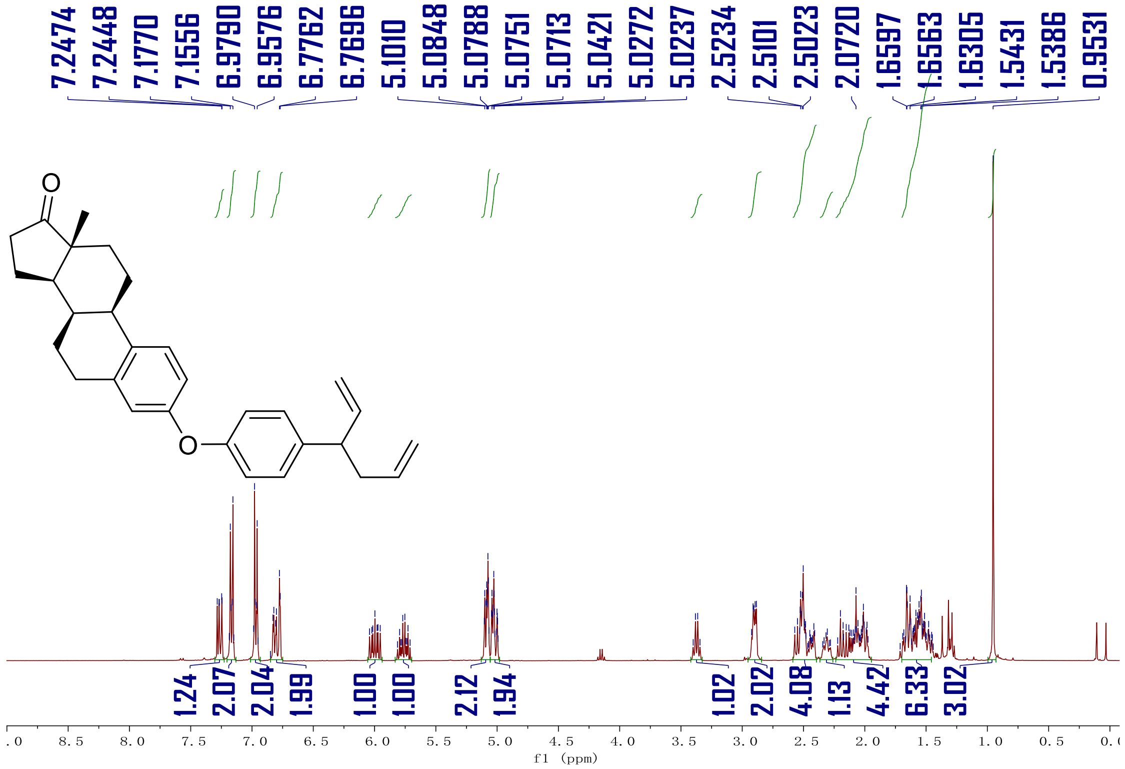

Supplementary Figure 159. ^{1}**H NMR (400 MHz, CDCl**$_3$**) of 31b**

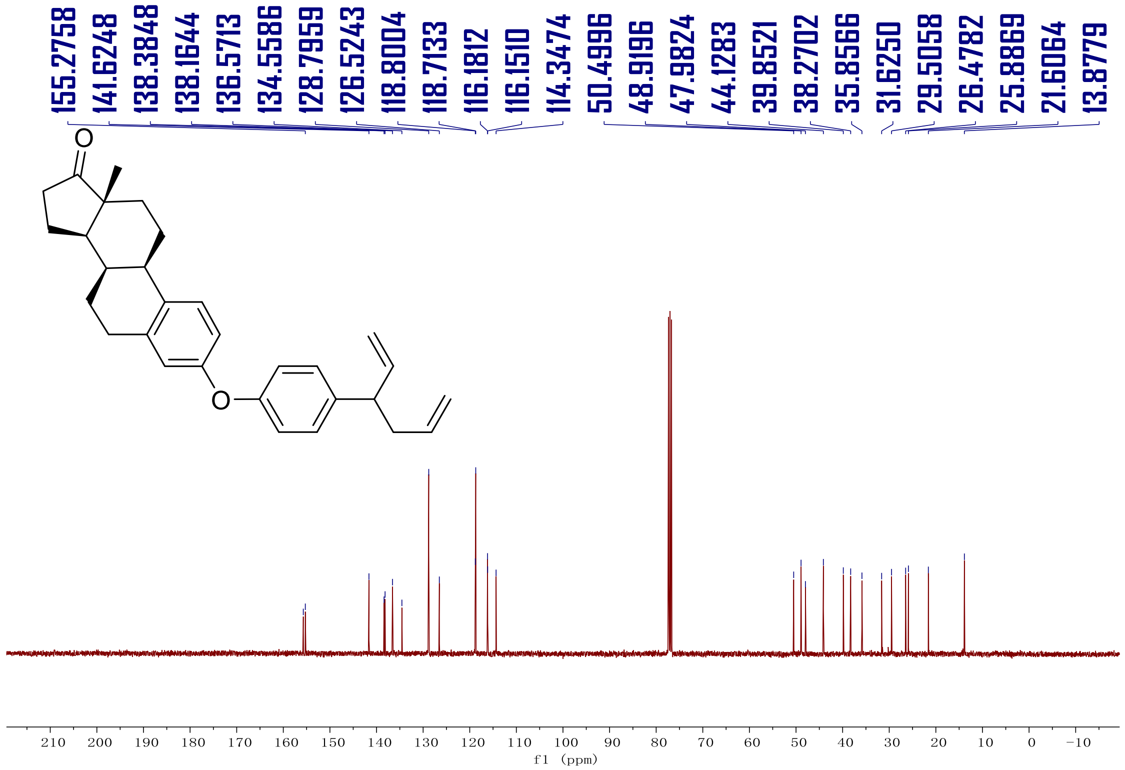

Supplementary Figure 160. ^{13}**C NMR (101 MHz, CDCl**$_3$**) of 31b**

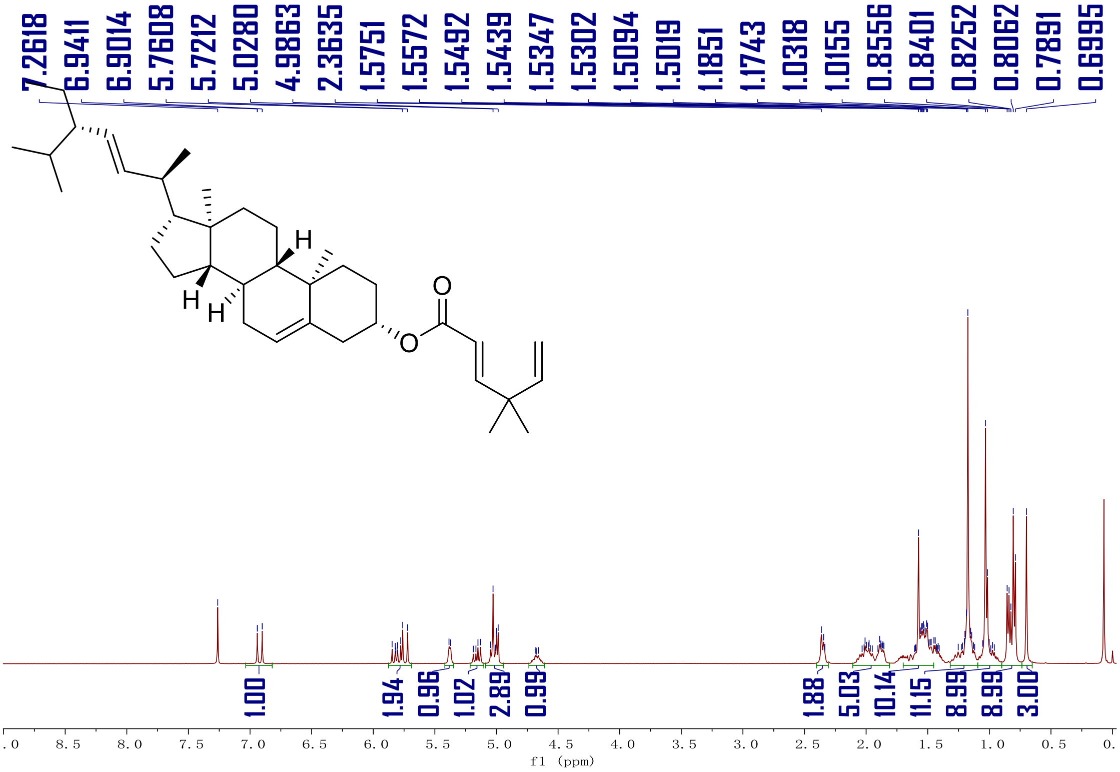

Supplementary Figure 161. ^{1}H NMR (400 MHz, CDCl$_3$) of 32b

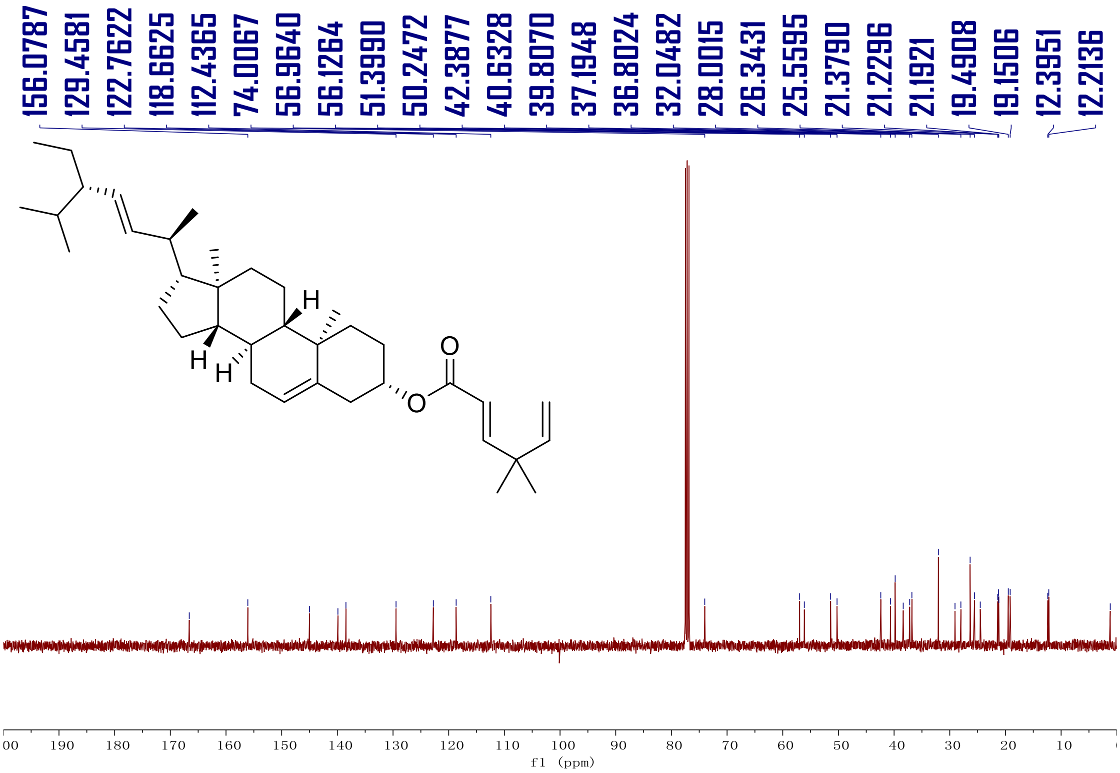

Supplementary Figure 162. ^{13}C NMR (101 MHz, CDCl$_3$) of 32b

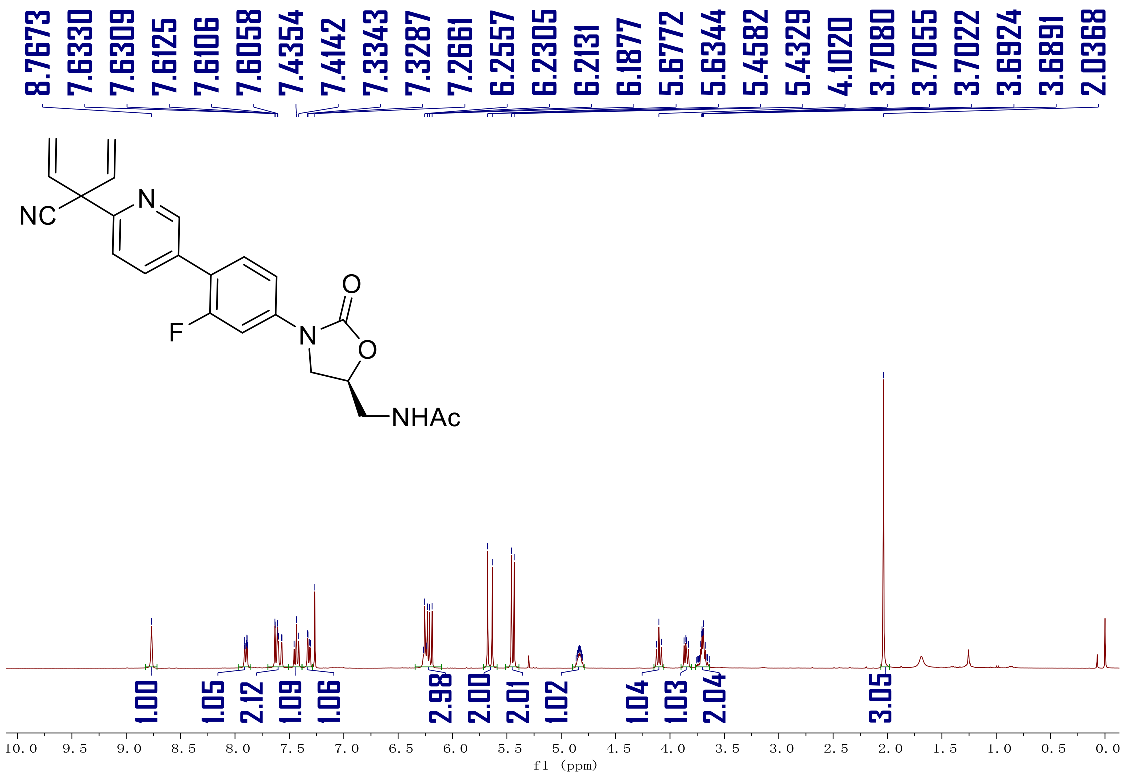

Supplementary Figure 163. ^{1}**H NMR (400 MHz,** $CDCl_3$**) of 33b**

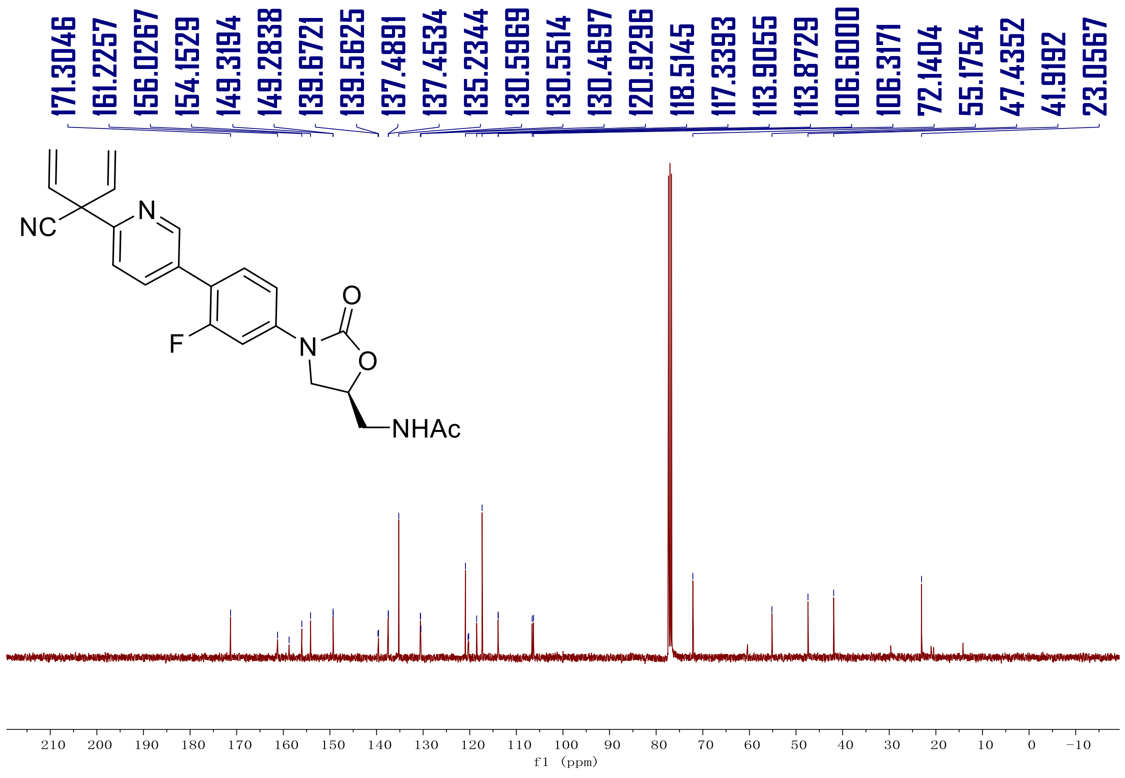

Supplementary Figure 164. ^{13}**C NMR (101 MHz,** $CDCl_3$**) of 33b**

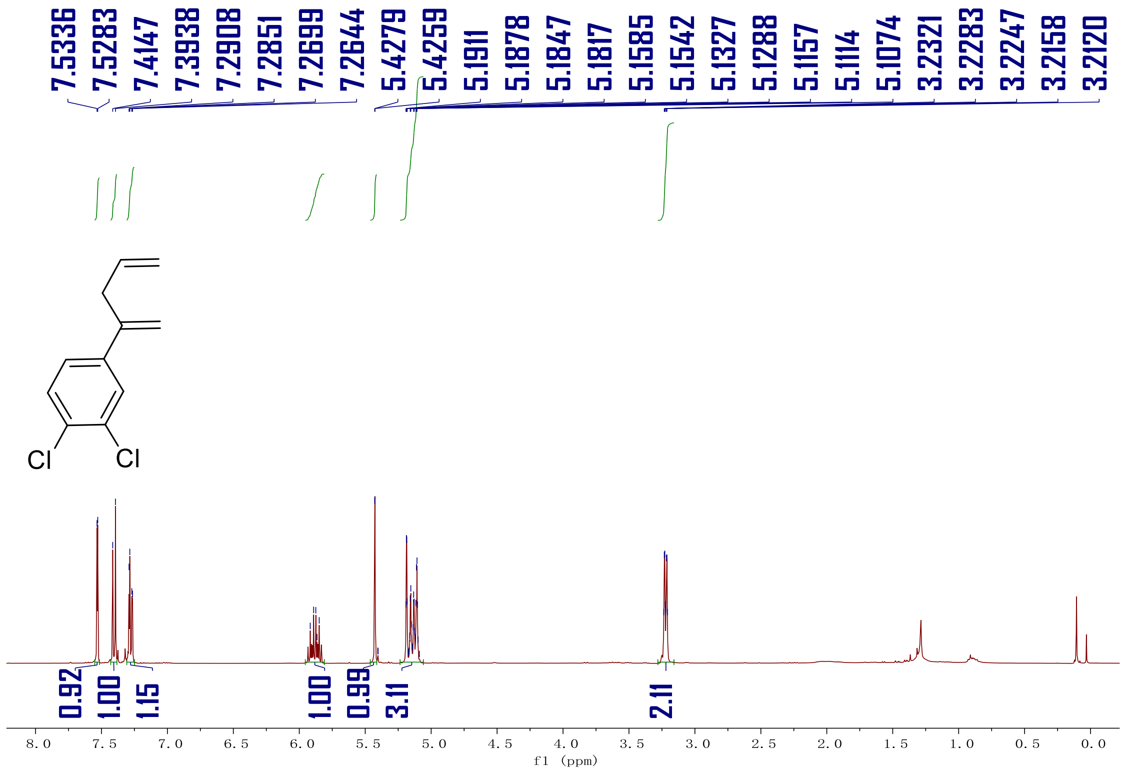

Supplementary Figure 165. ^{1}H NMR (400 MHz, $CDCl_3$) of 34b

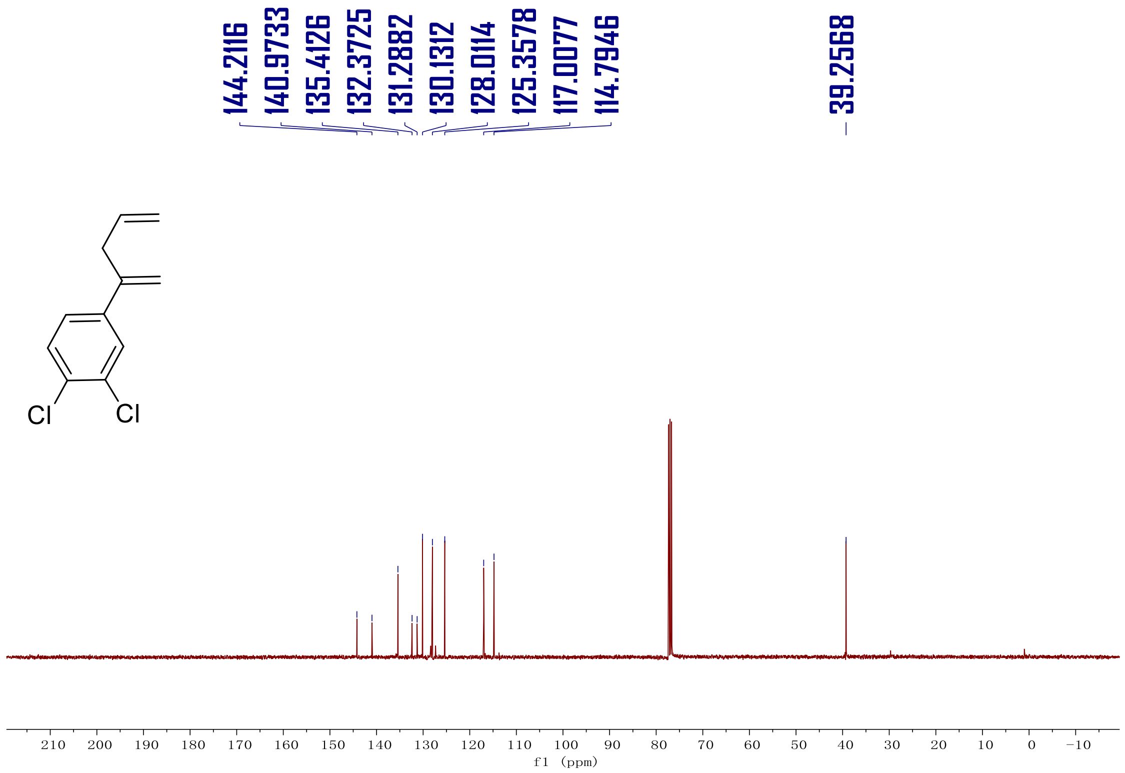

Supplementary Figure 166. ^{13}C NMR (101 MHz, $CDCl_3$) of 34b

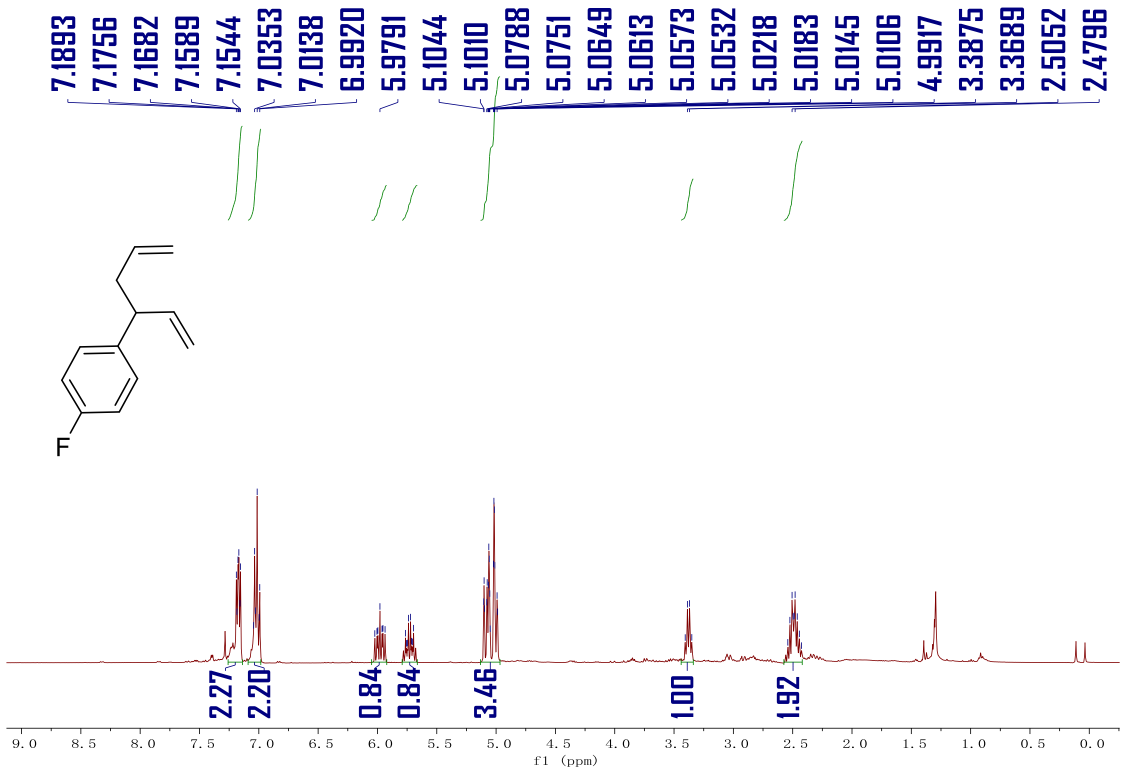

Supplementary Figure 167. ^{1}H NMR (400 MHz, $CDCl_3$) of 35b

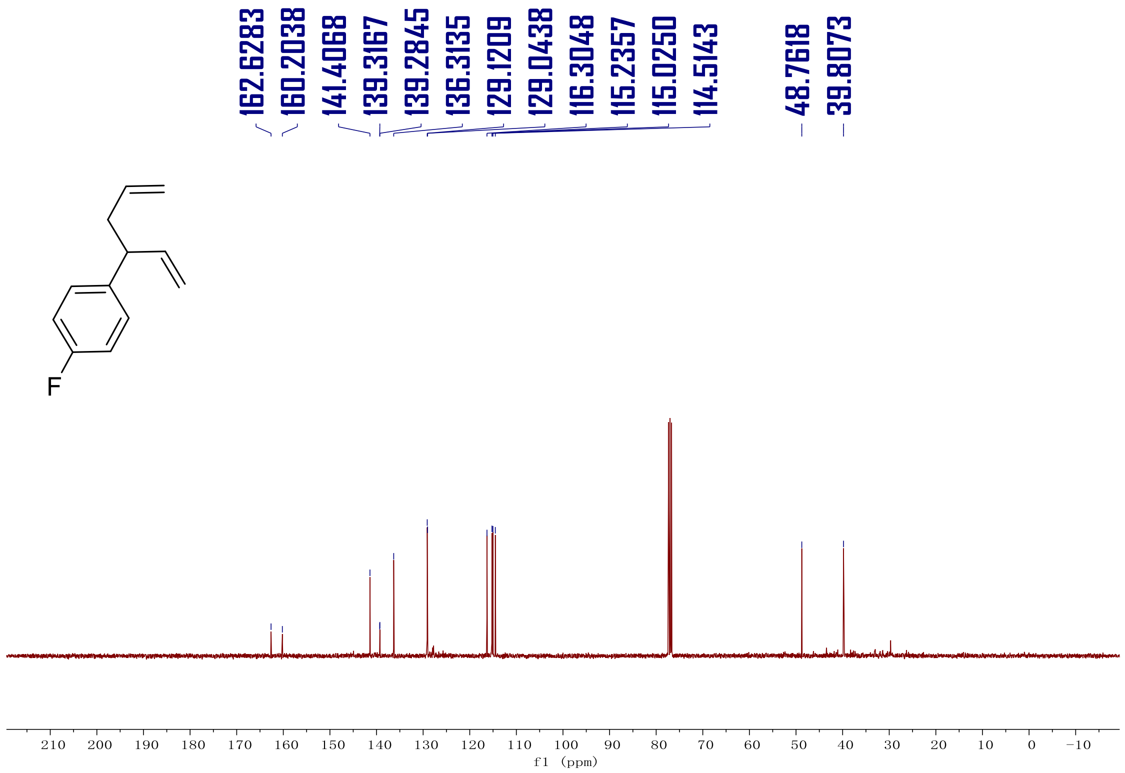

Supplementary Figure 168. ^{13}C NMR (101 MHz, $CDCl_3$) of 35b

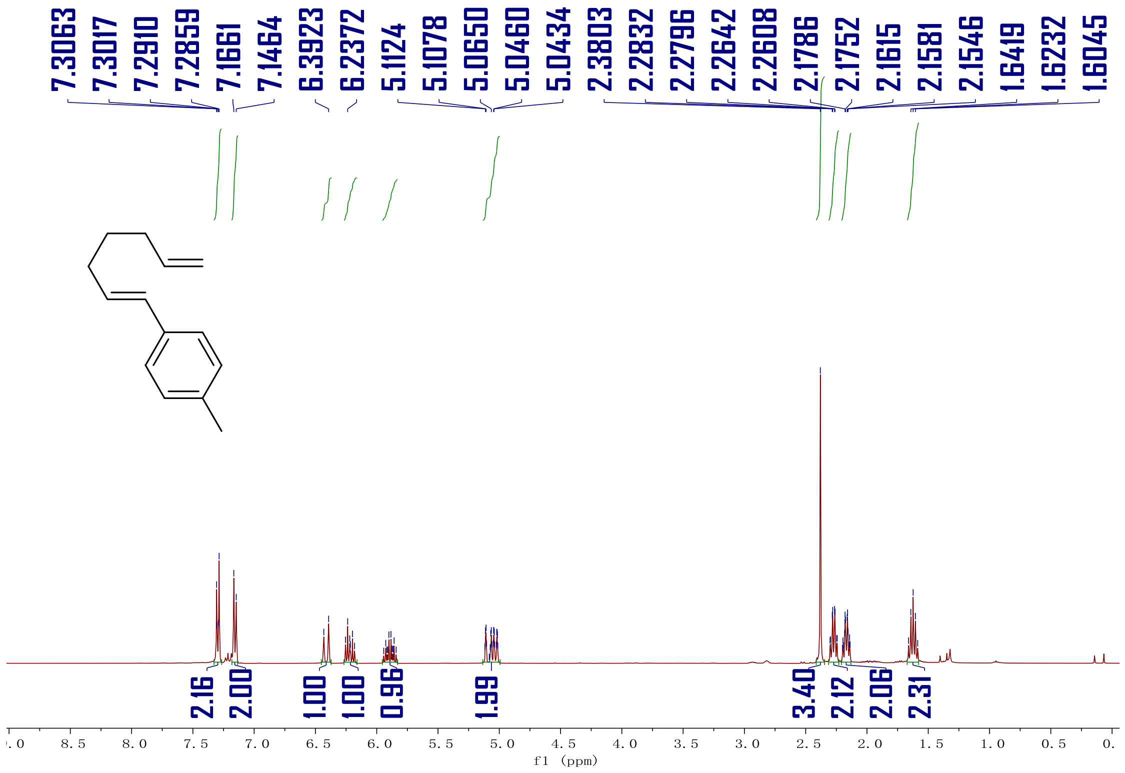

Supplementary Figure 169. ^{1}H NMR (400 MHz, $CDCl_3$) of 36b

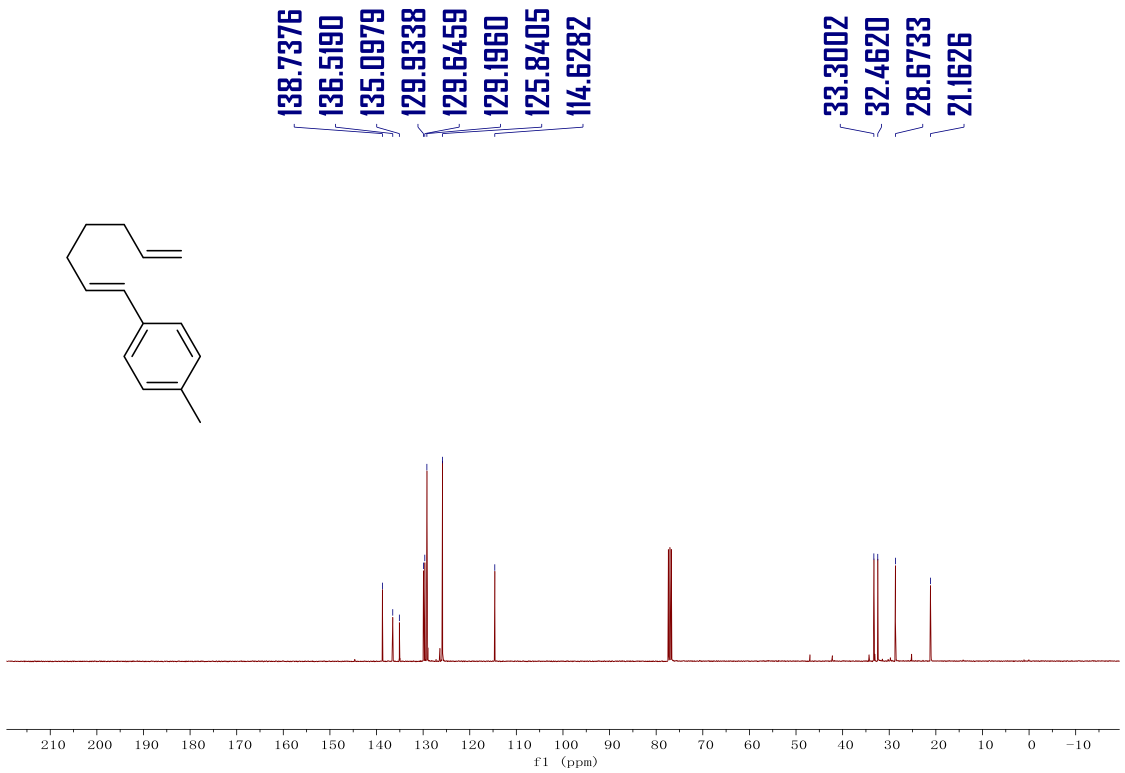

Supplementary Figure 170. ^{13}C NMR (101 MHz, $CDCl_3$) of 36b

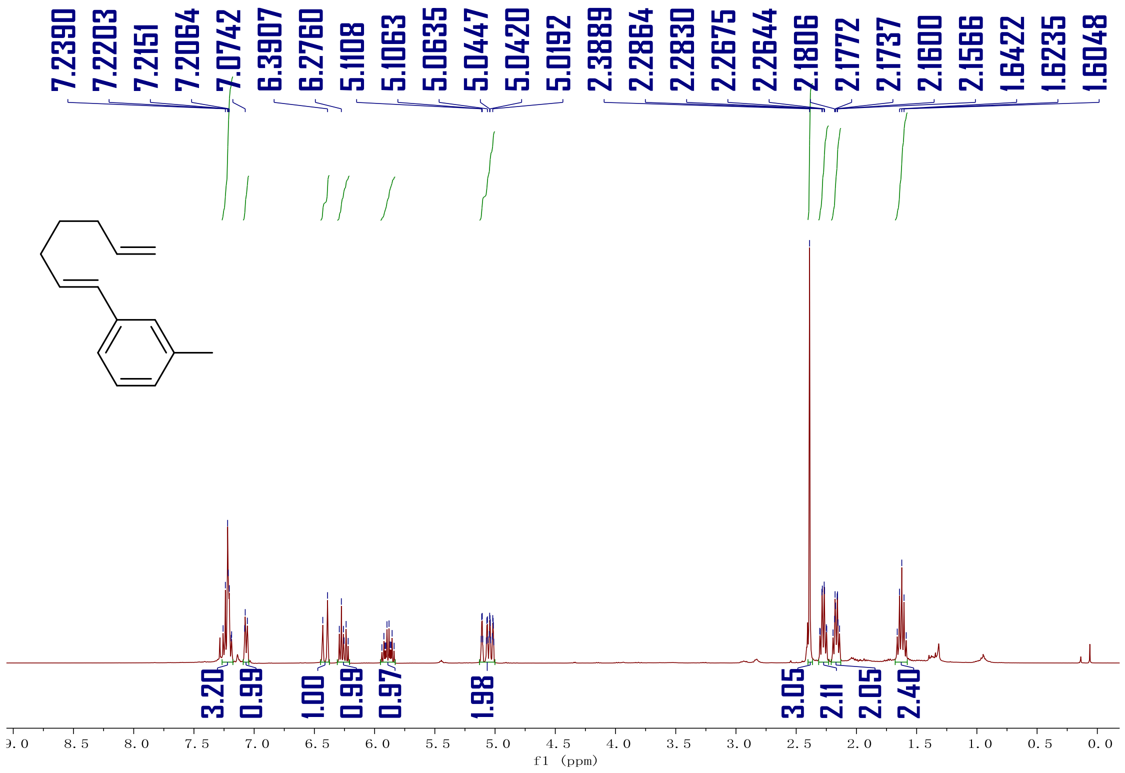

Supplementary Figure 171. ^{1}**H NMR (400 MHz, CDCl$_3$) of 37b**

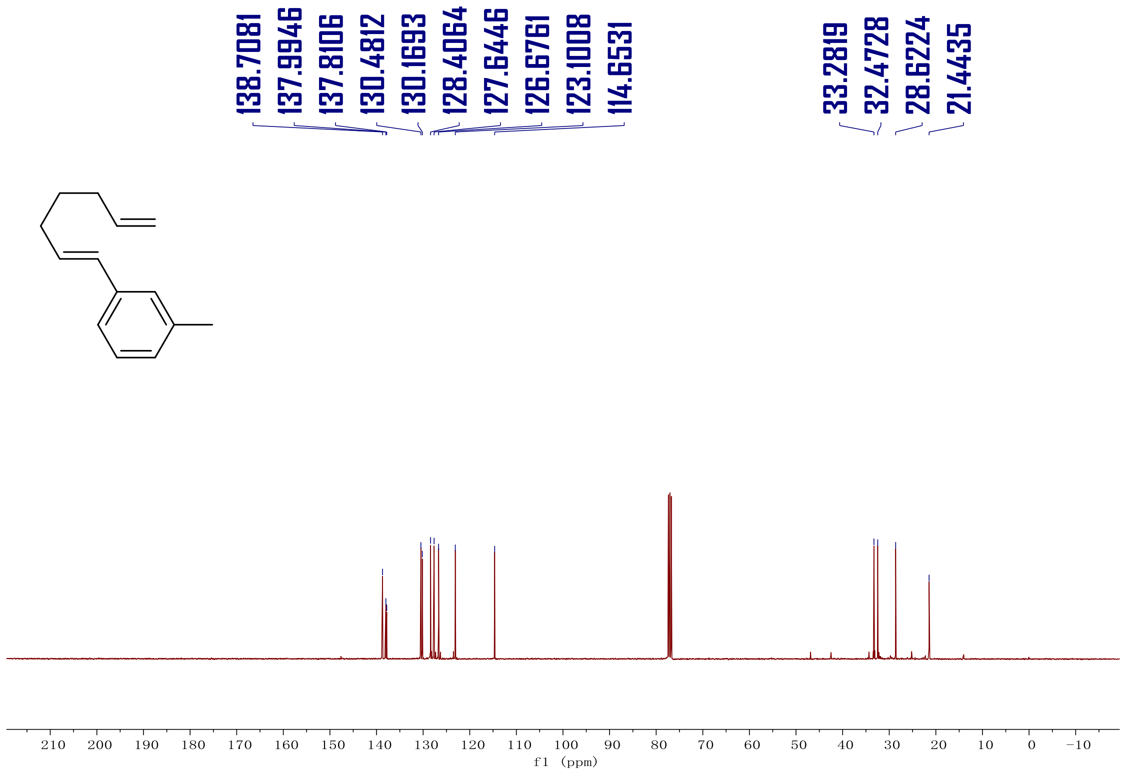

Supplementary Figure 172. ^{13}**C NMR (101 MHz, CDCl$_3$) of 37b**

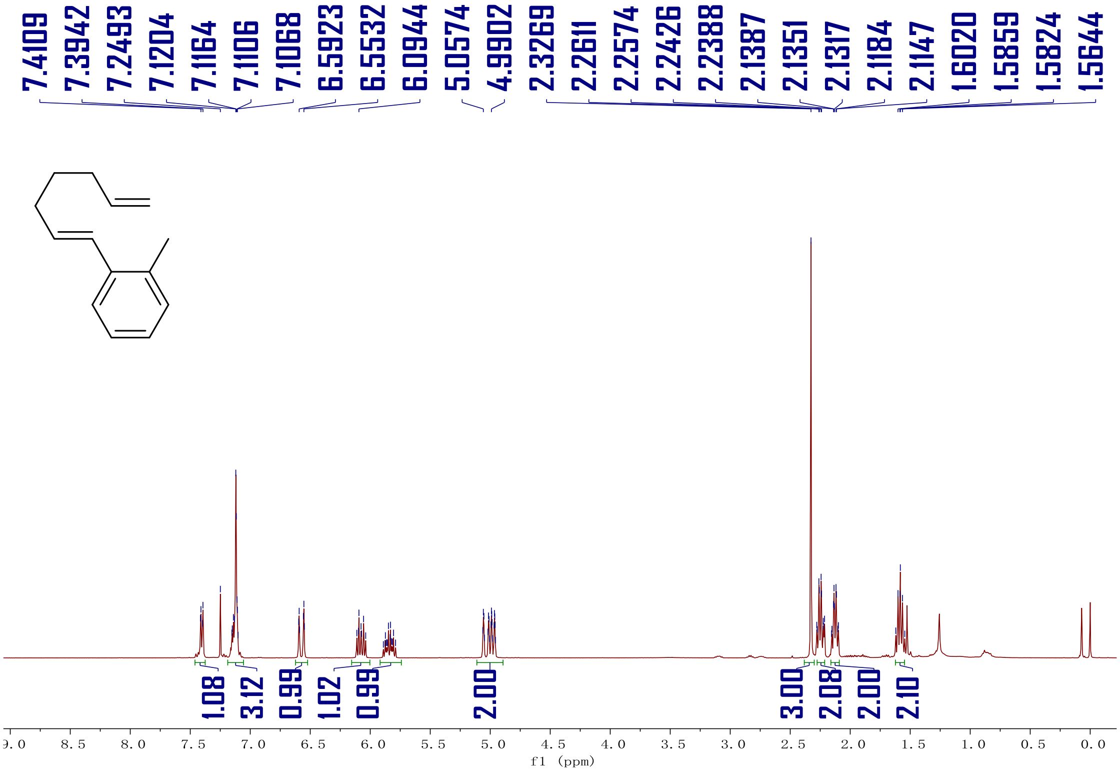

Supplementary Figure 173. ^{1}H NMR (400 MHz, $CDCl_3$) of 38b

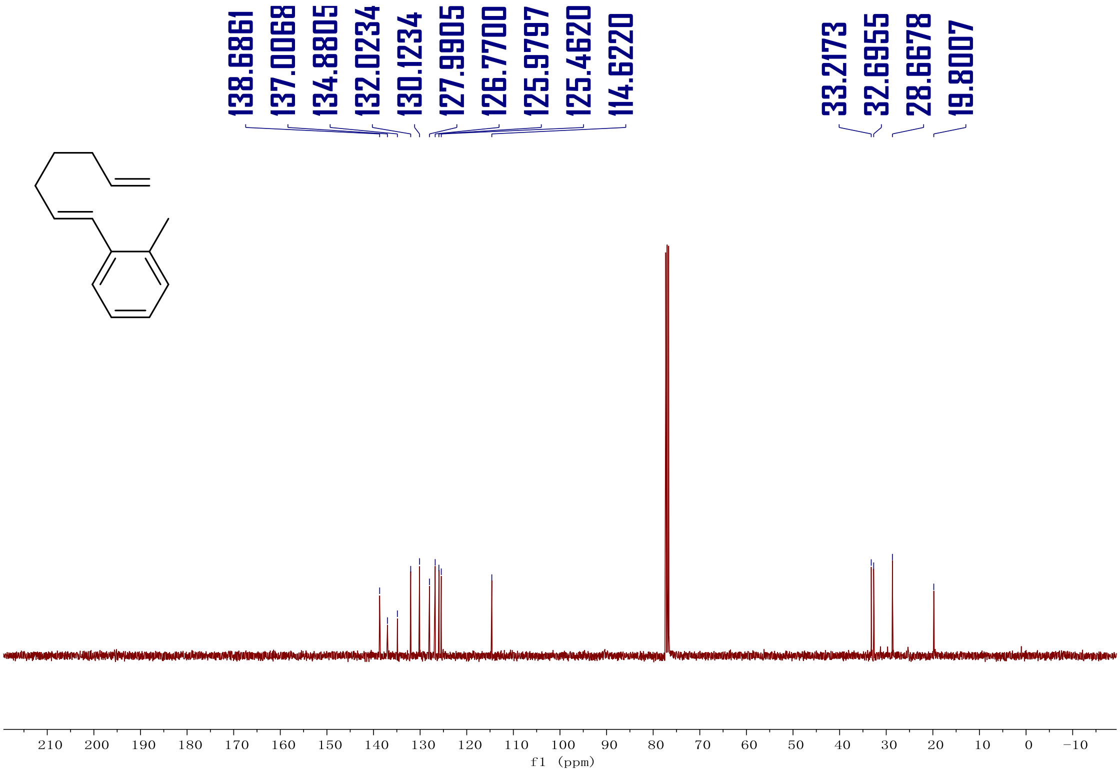

Supplementary Figure 174. ^{13}C NMR (101 MHz, $CDCl_3$) of 38b

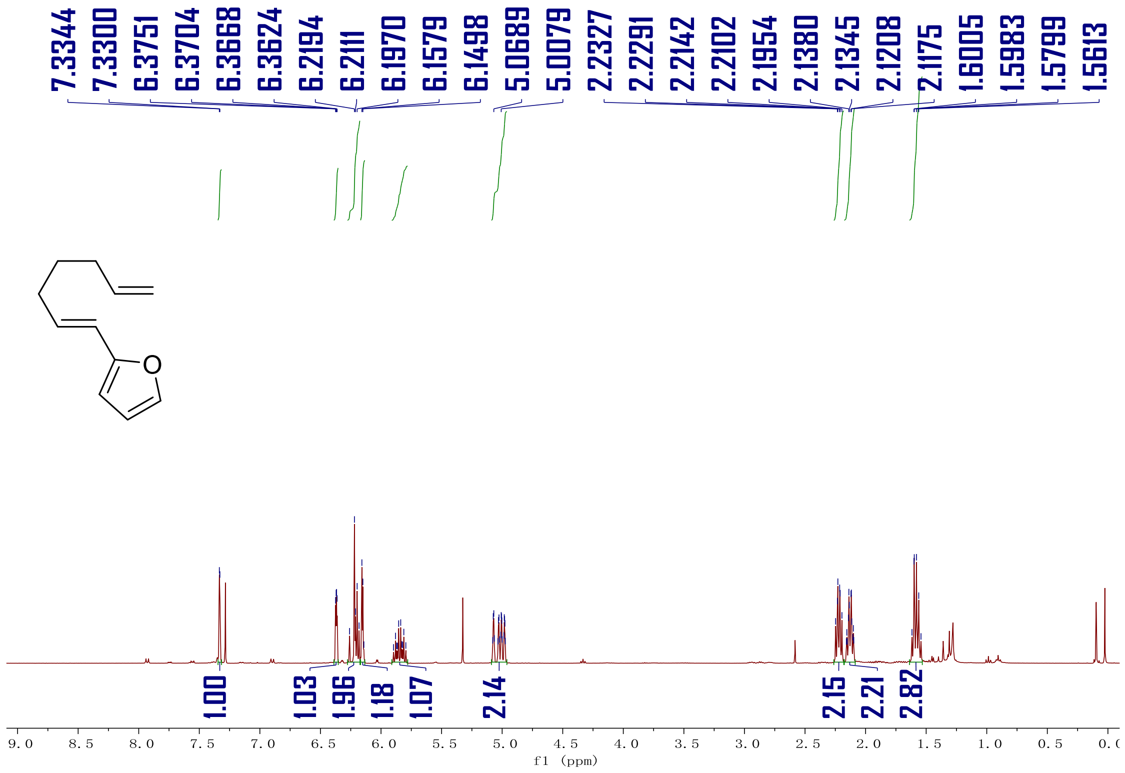

Supplementary Figure 175. ^{1}**H NMR (400 MHz,** $CDCl_3$**) of 39b**

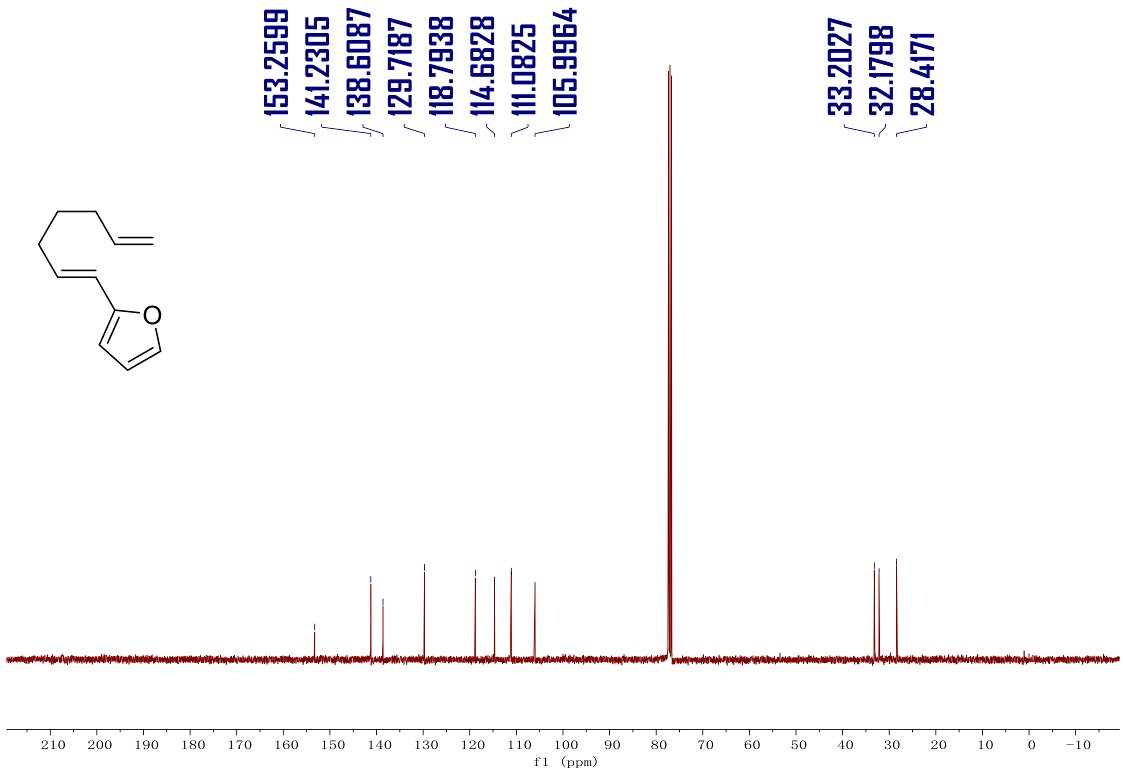

Supplementary Figure 176. ^{13}**C NMR (101 MHz,** $CDCl_3$**) of 39b**

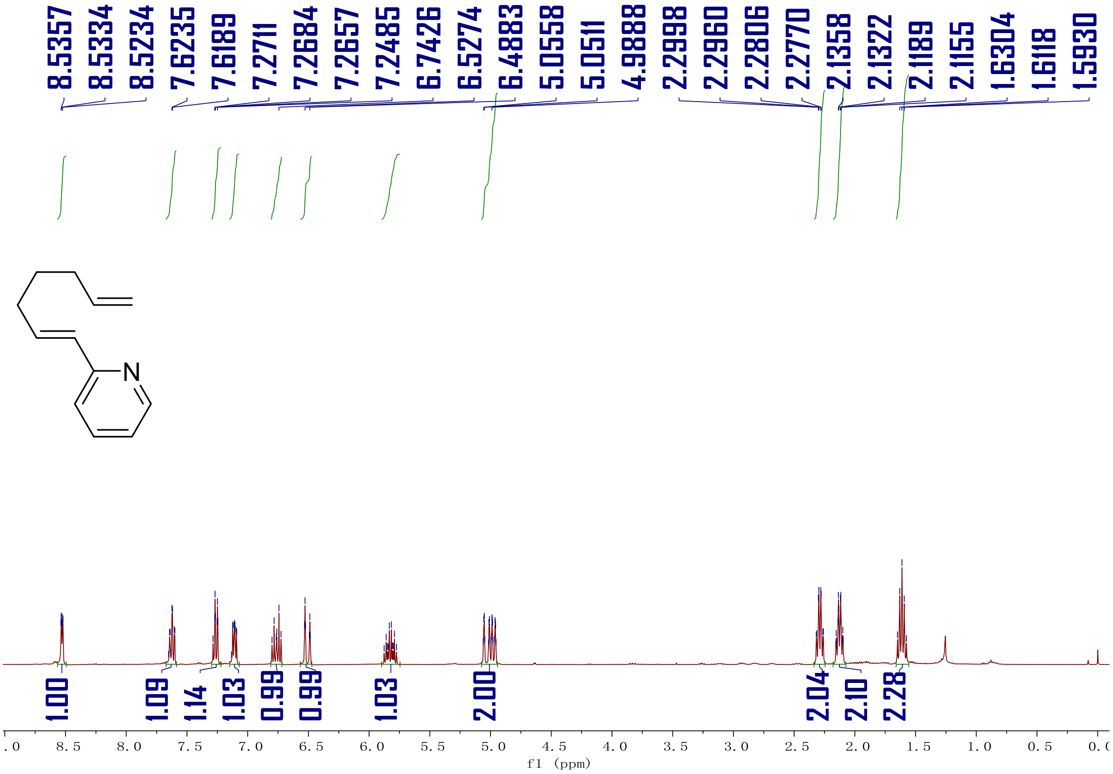

Supplementary Figure 177. ^{1}H NMR (400 MHz, CDCl$_3$) of 40b

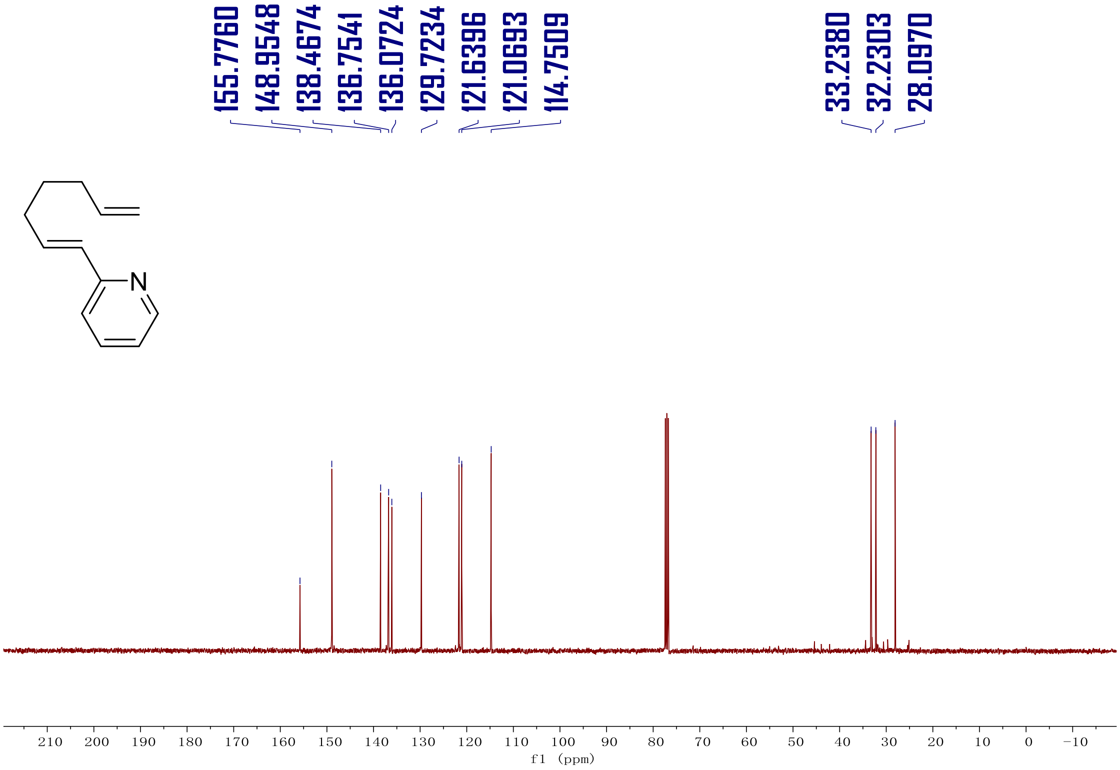

Supplementary Figure 178. ^{13}C NMR (101 MHz, CDCl$_3$) of 40b

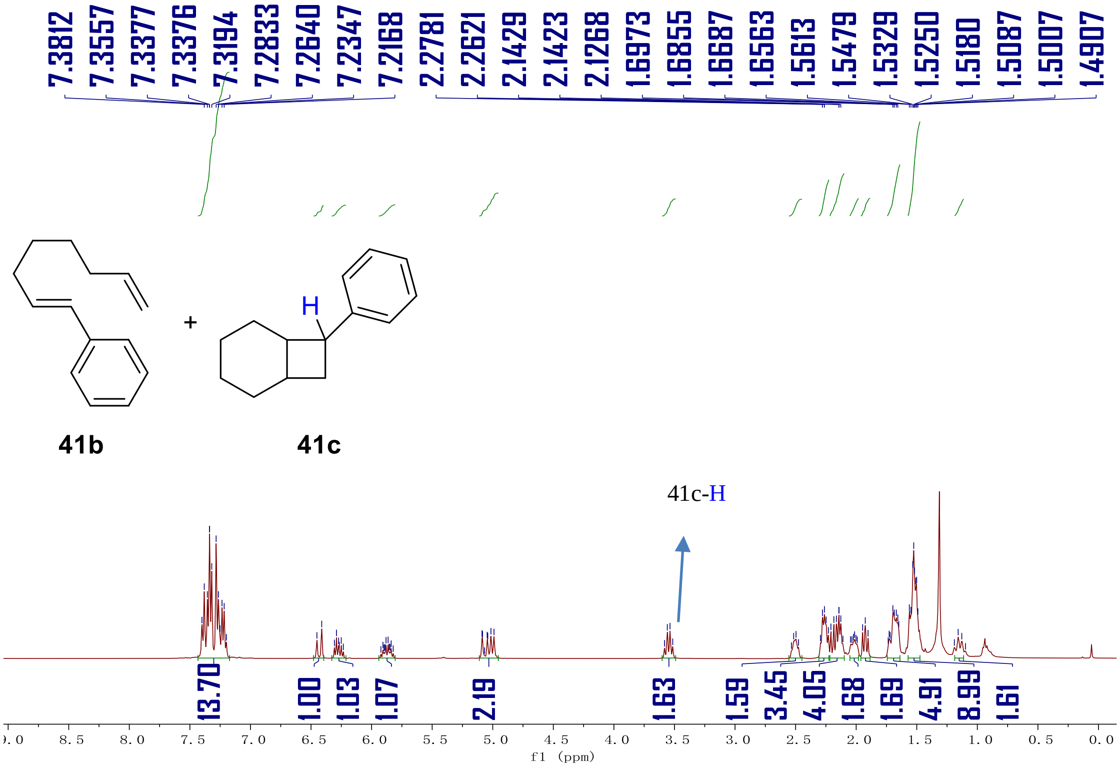

Supplementary Figure 179. ^{1}H NMR (400 MHz, $CDCl_3$) of 41b and 41c

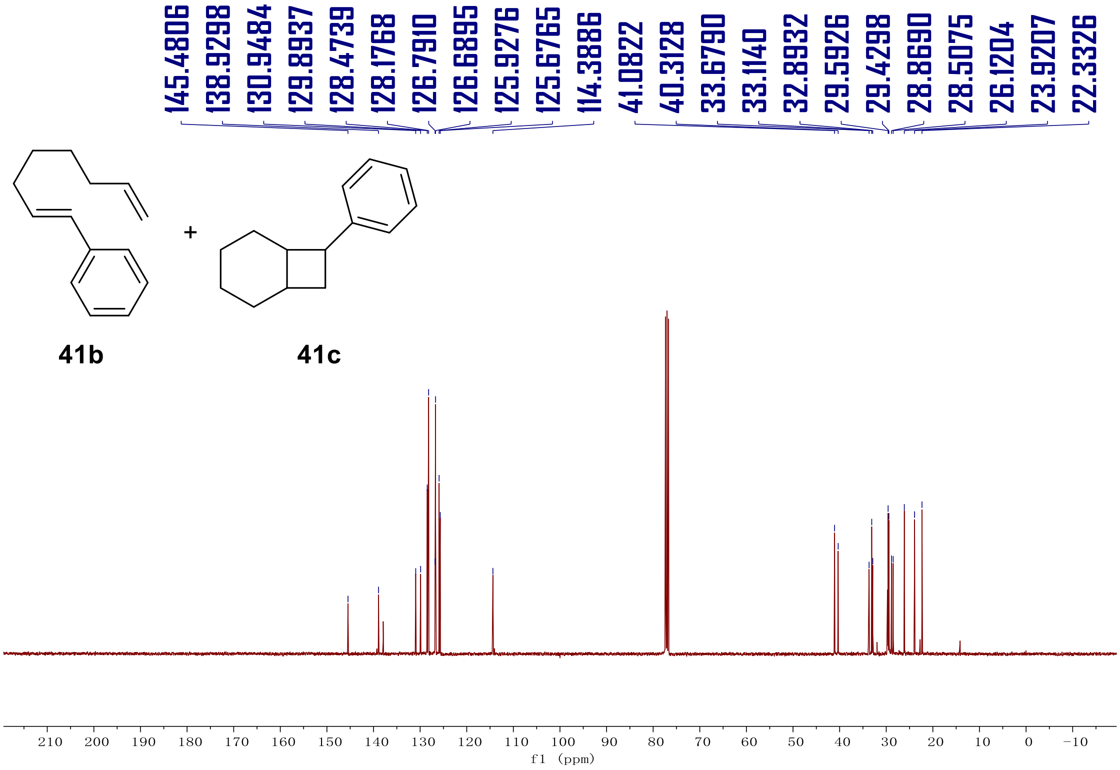

Supplementary Figure 180. ^{13}C NMR (101 MHz, $CDCl_3$) of 41b and 41c

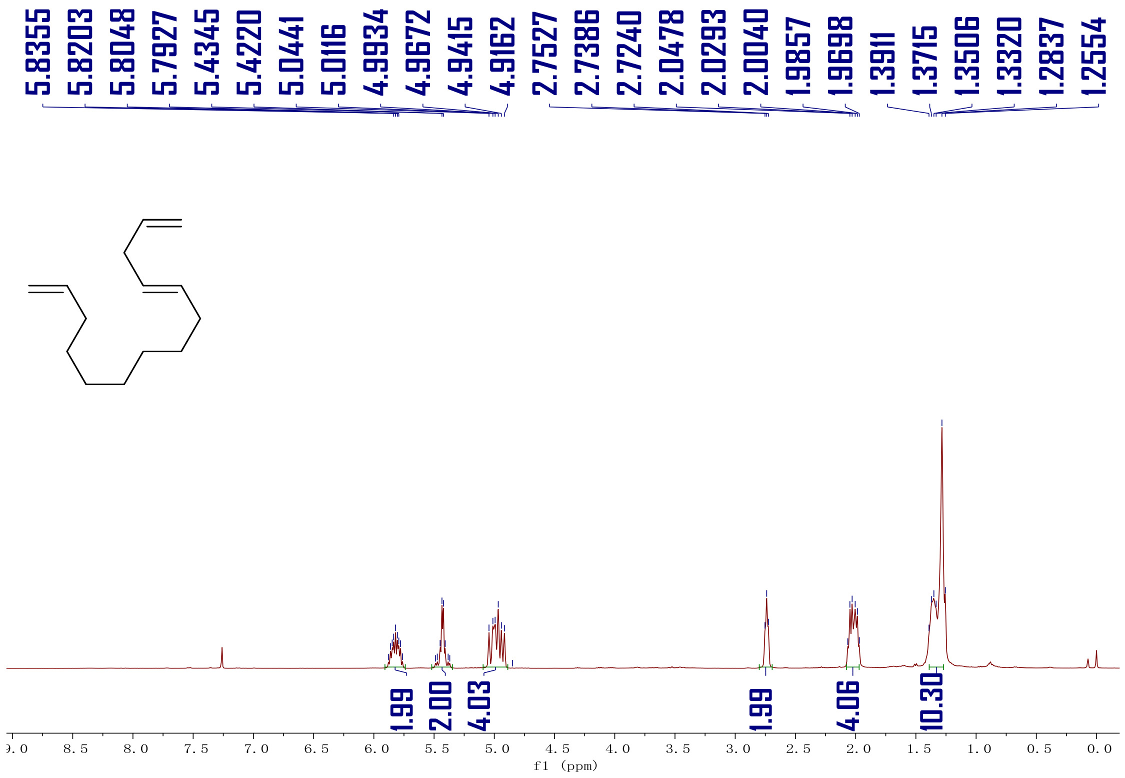

Supplementary Figure 181. ^{1}**H NMR (400 MHz, CDCl**$_3$**) of 42b**

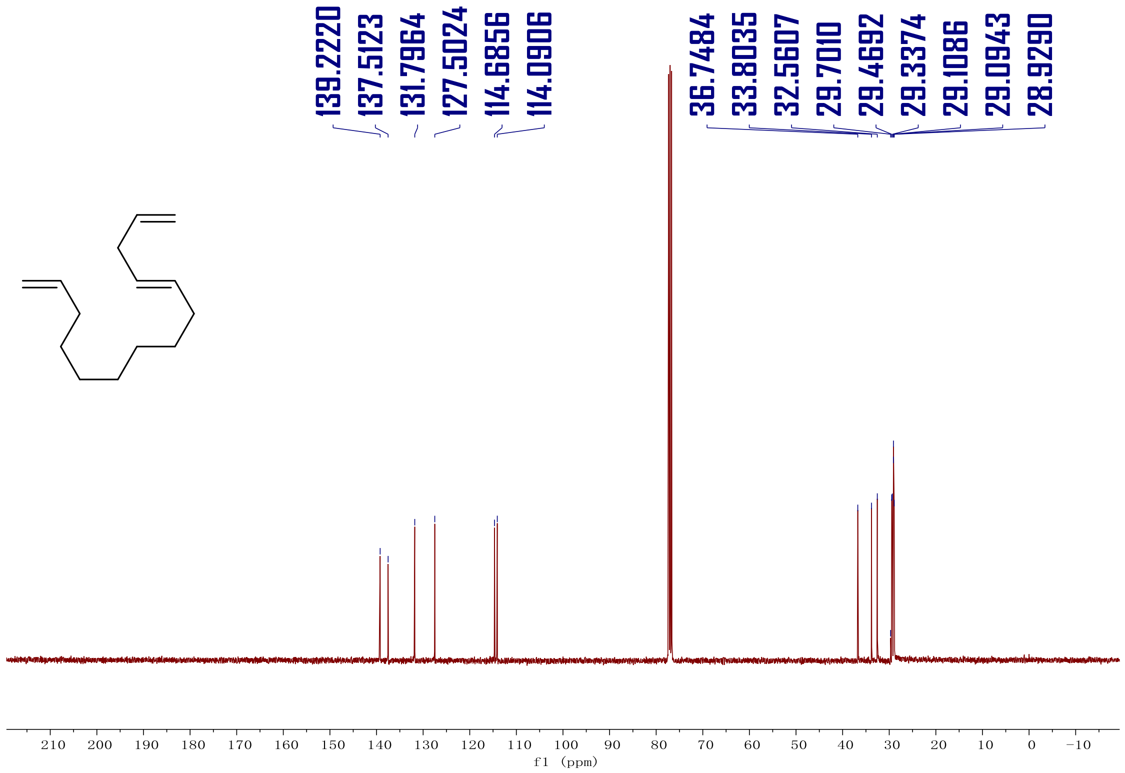

Supplementary Figure 182. ^{13}**C NMR (101 MHz, CDCl**$_3$**) of 42b**

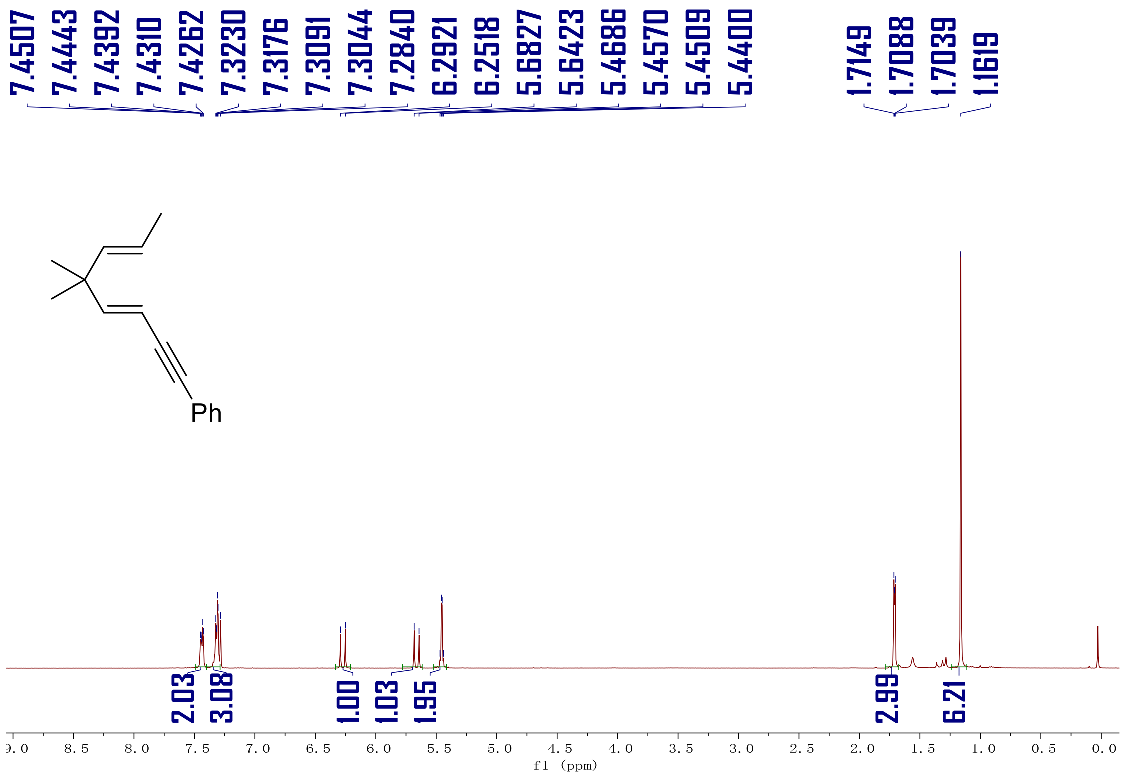

Supplementary Figure 183. ¹H NMR (400 MHz, CDCl₃) of 43b

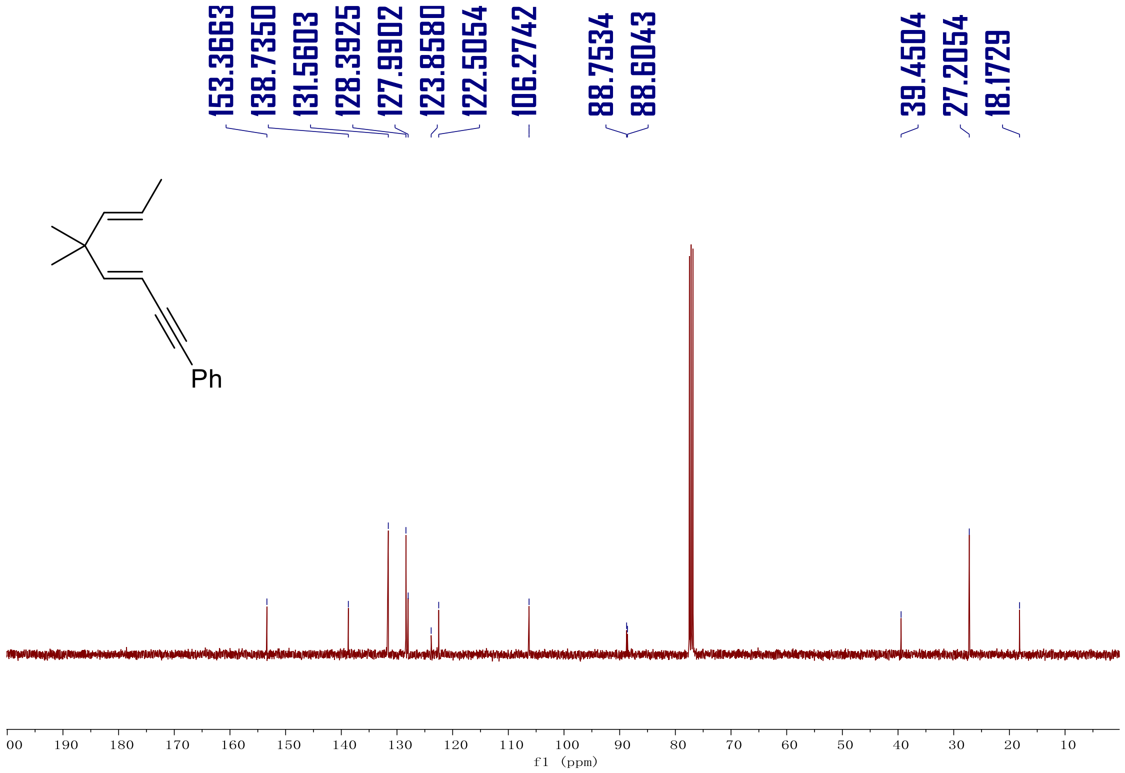

Supplementary Figure 184. ¹³C NMR (101 MHz, CDCl₃) of 43b

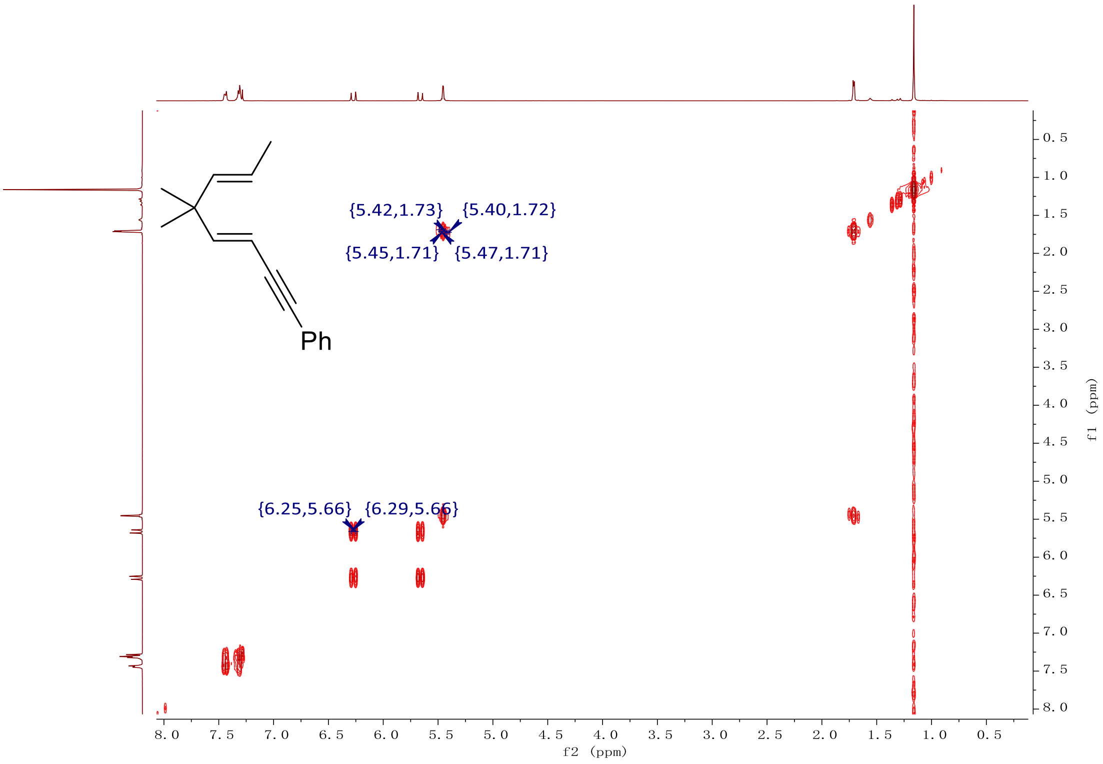

Supplementary Figure 185. COSY (400 MHz, $CDCl_3$) of 43b

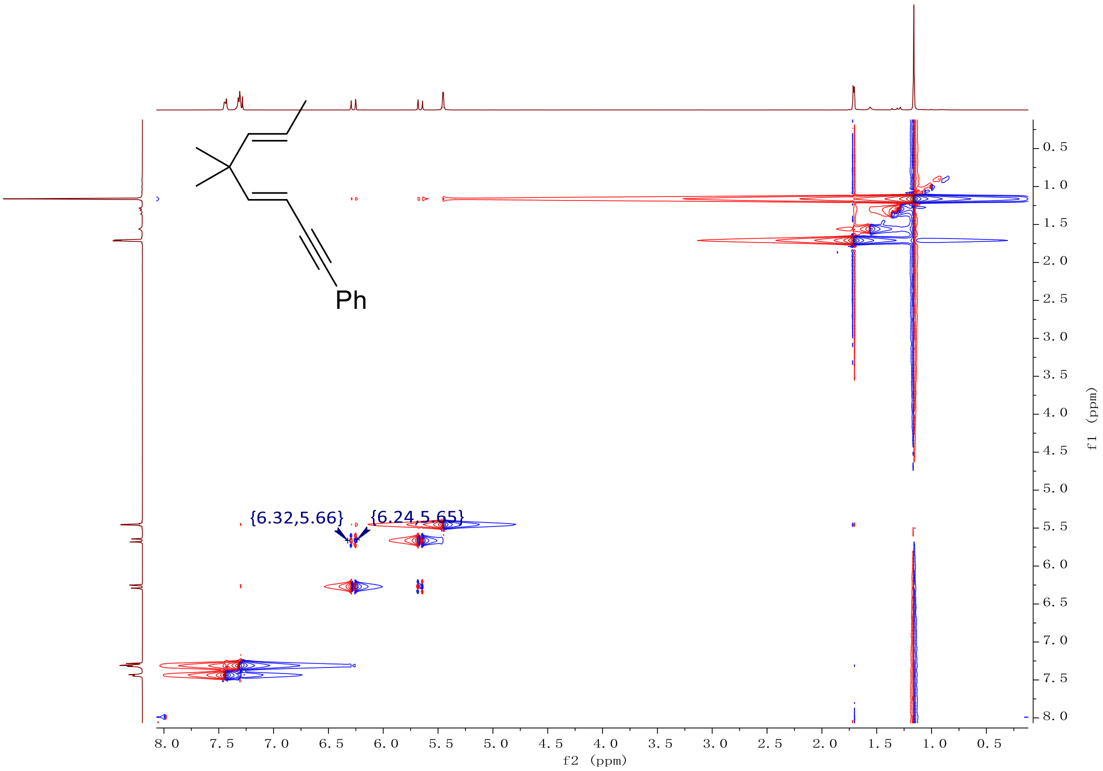

Supplementary Figure 186. NOESY (400 MHz, $CDCl_3$) of 43b

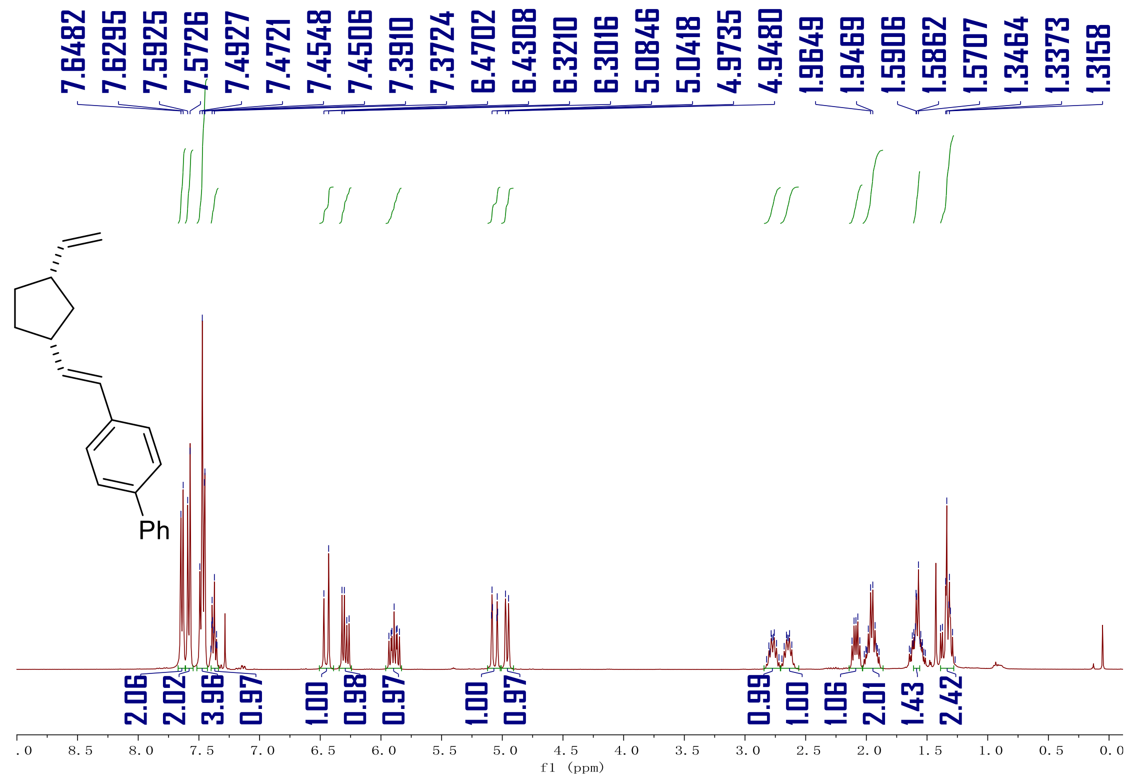

Supplementary Figure 187. ^{1}H NMR (400 MHz, $CDCl_3$) of 44b

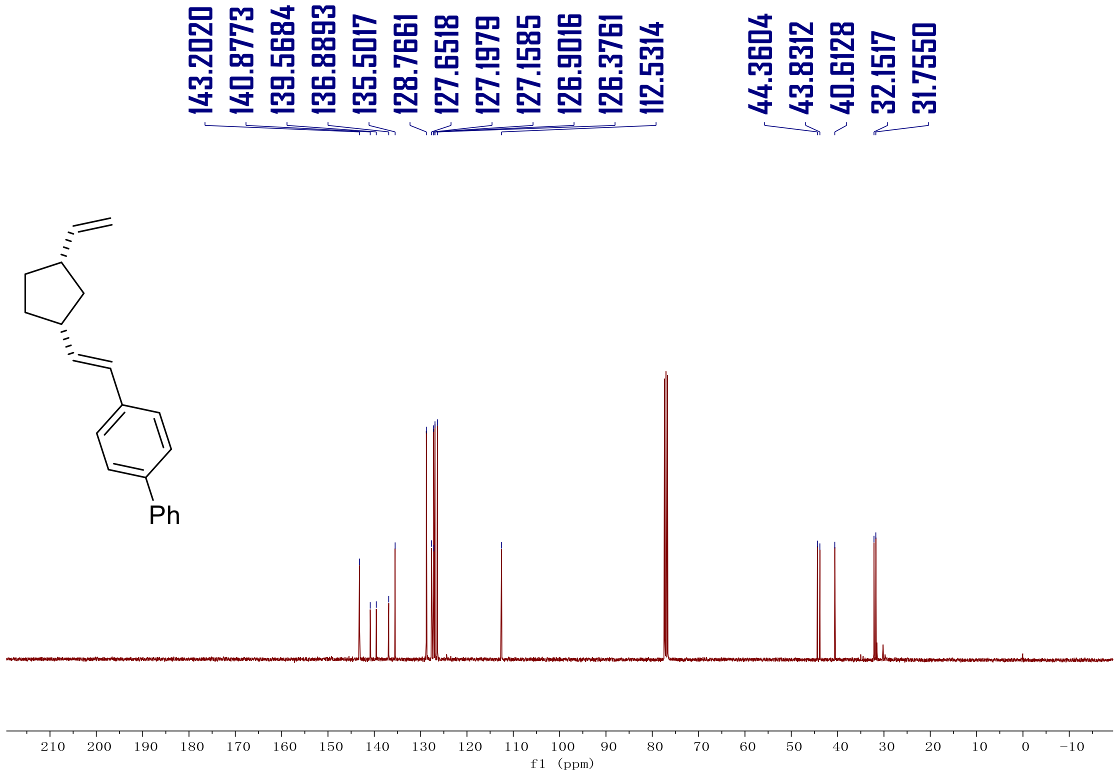

Supplementary Figure 188. ^{13}C NMR (101 MHz, $CDCl_3$) of 44b

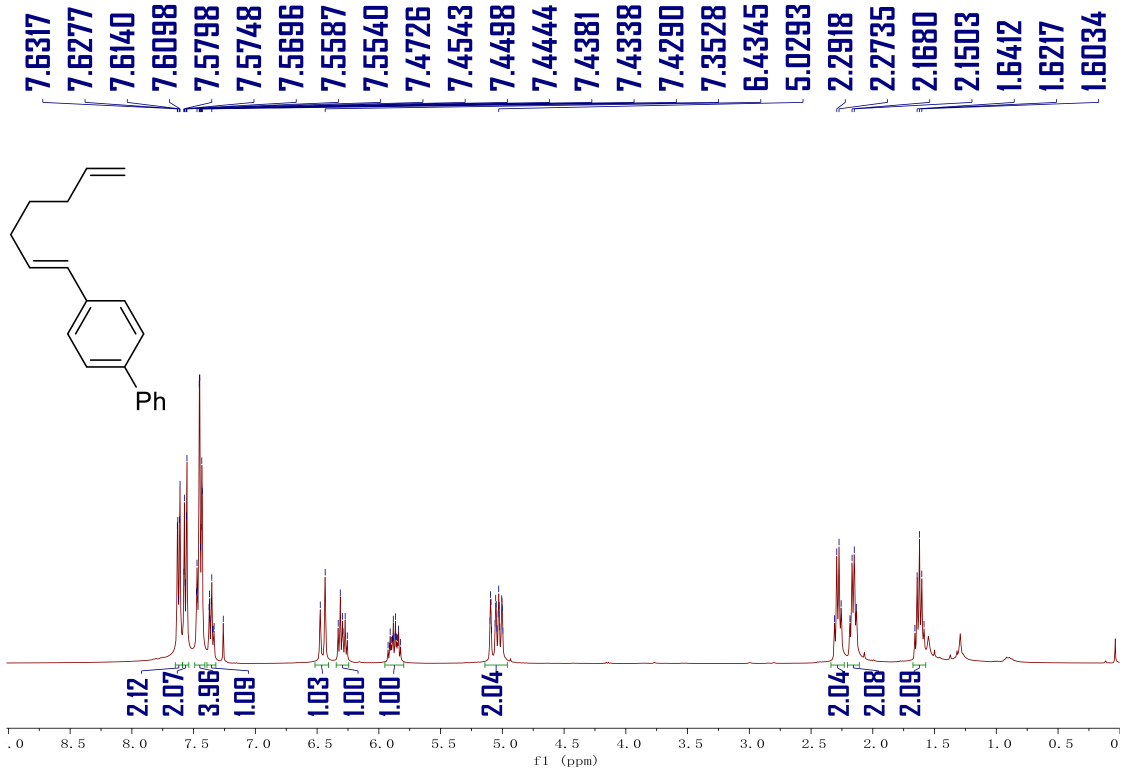

Supplementary Figure 189. ^{1}H NMR (400 MHz, $CDCl_3$) of 45b

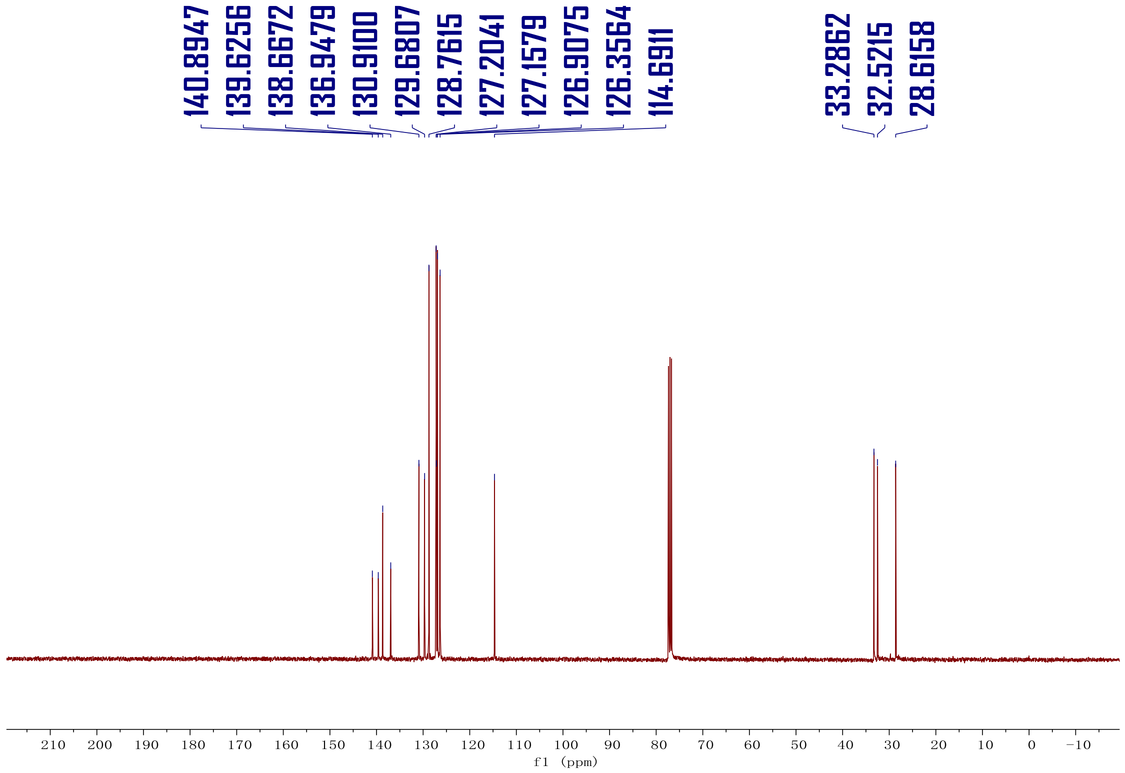

Supplementary Figure 190. ^{13}C NMR (101 MHz, $CDCl_3$) of 45b

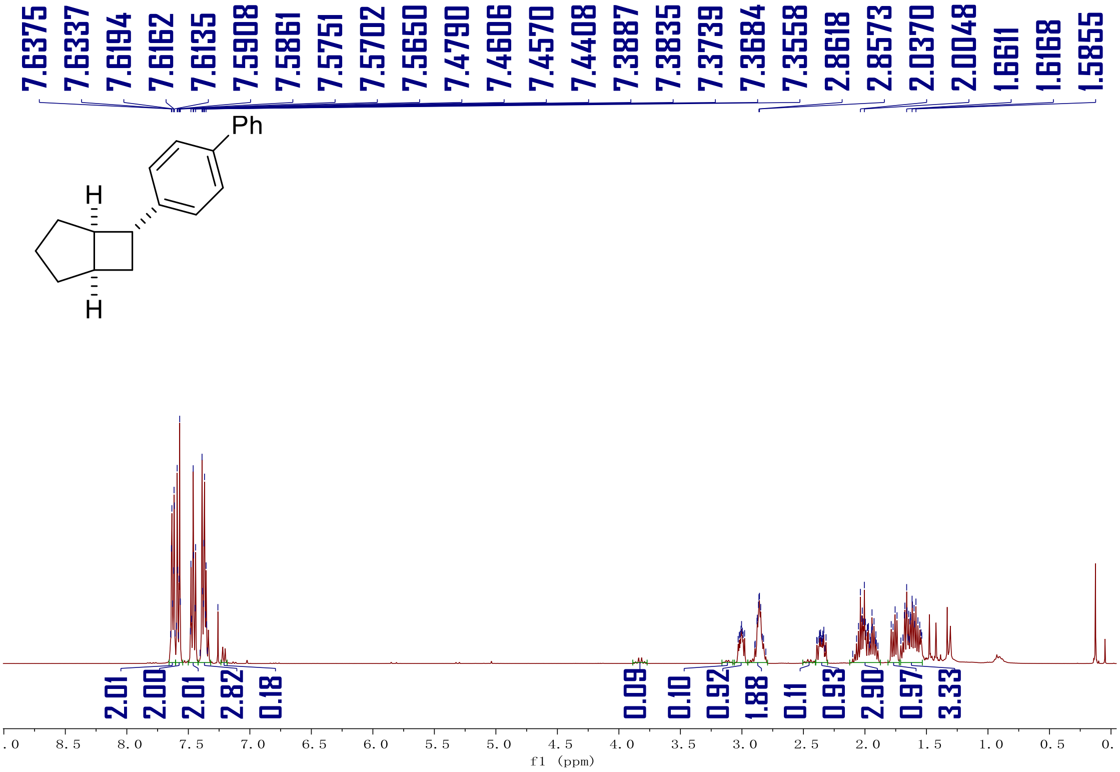

Supplementary Figure 191. ^{1}H NMR (400 MHz, $CDCl_3$) of 45c

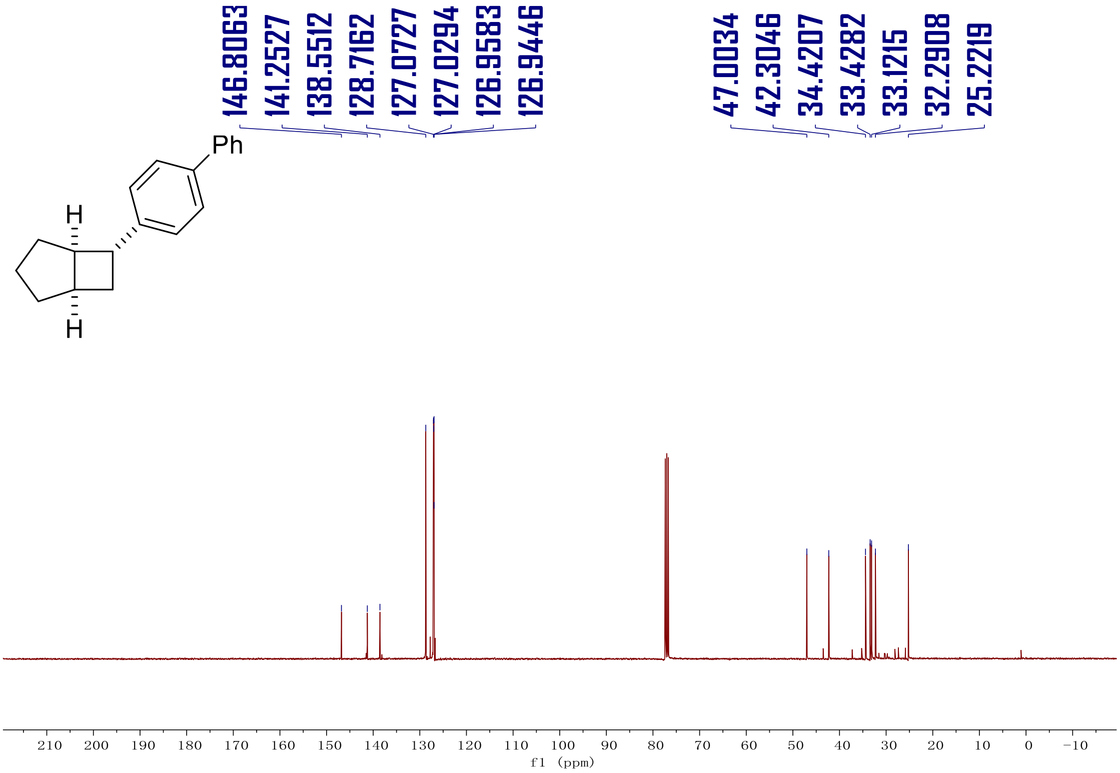

Supplementary Figure 192. ^{13}C NMR (101 MHz, $CDCl_3$) of 45c

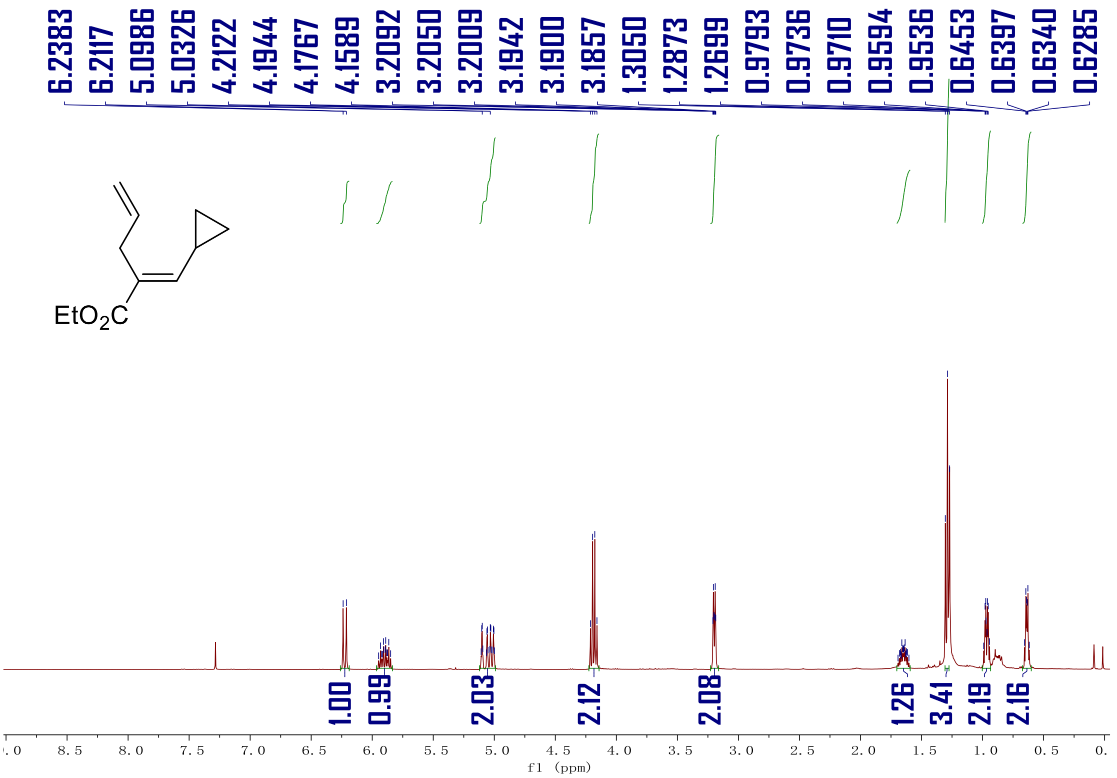

Supplementary Figure 193. ^{1}**H NMR (400 MHz,** $CDCl_3$**) of 46b**

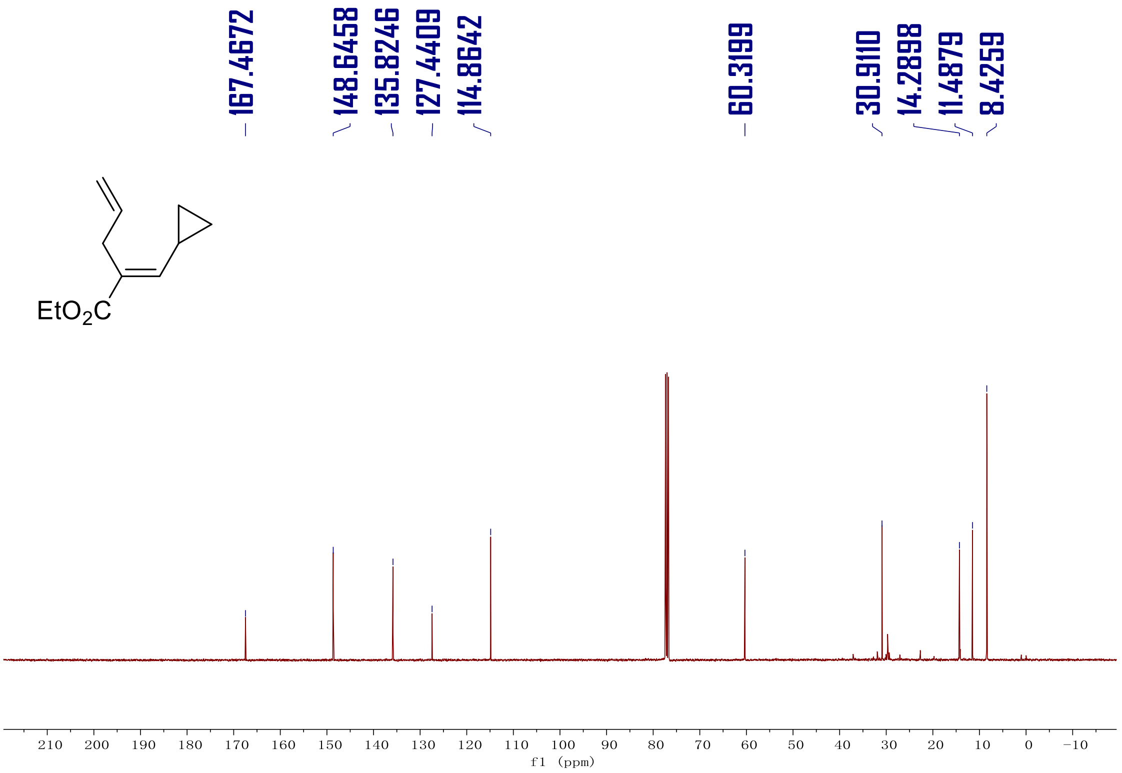

Supplementary Figure 194. ^{13}**C NMR (101 MHz,** $CDCl_3$**) of 46b**

Supplementary References

1 a) Toh, K. K., Wang, Y. F., Ng, E. P. J., Chiba, S. Copper-Mediated Aerobic Synthesis of 3-Azabicyclo[3.1.0]hex-2-enes and 4-Carbonylpyrroles from *N*-Allyl/Propargyl Enamine Carboxylates. *J. Am. Chem. Soc*. **133**, 13942–13945 (2011). b) Toh, K. K., Biswas, A., Wang, Y. F., Tan, Y. Y., Chiba, S. Copper-Mediated Oxidative Transformation of *N*-Allyl Enamine Carboxylates toward Synthesis of Azaheterocycles. *J. Am. Chem. Soc.* **136**, 6011–6020 (2014).

2 Komine, T., Kojima, A., Asahina, Y., Saito, T., Takano, H., Shibue, T., Fukuda, Y. Synthesis and Structure-Activity Relationship Studies of Highly Potent Novel Oxazolidinone Antibacterials. *J. Med. Chem.* **51**, 6558–6562 (2008).

3 Jirásek, M.; Straková, K.; Neveselý, T.; Svobodová, E.; Rottnerová, Z.; Cibulka, R. Flavin-Mediated Visible-Light [2+2] Photocycloaddition of Nitrogen- and Sulfur-Containing Dienes. *Eur. J. Org. Chem*. 2139–2146 (2017).

4 a) Paul, A., Seidel, D. α-Functionalization of Cyclic Secondary Amines: Lewis Acid Promoted Addition of Organometallics to Transient Imines. *J. Am. Chem. Soc.* **141**, 8778–8782 (2019). b) Chen, W. J., Ma, L., Paul, A., Seidel, D. Direct α-C–H bond functionalization of unprotected cyclic amines. *Nat. Chem*. **10**, 165–169 (2018).

5 Lu, Z., Yoon, T. P. Visible Light Photocatalysis of [2+2] Styrene Cycloadditions by Energy Transfer. *Angew. Chem. Int. Ed.* **51**, 10329–10332 (2012).

6 Nakajima, M., Anselme, J. P. Reaction of *N*-Nitrosamides with Metal Hydrides. *J. Org. Chem*. **45**, 3673–3676 (1980).

7 Carpino, L. A., Oxidation of N-Aminodihydroisoindoles Synthesis of cis- and trans-1,2-Diphenylbenzocyclobutenes. *J. Am. Chem. Soc.* **84**, 2196–2201 (1962).

8 a) Zou, X. D., Zou, J. Q., Yang, L. Z., Li, G. G., Lu, H. J., Thermal Rearrangement of Sulfamoyl Azides: Reactivity and Mechanistic Study. *J. Org. Chem.* **82**, 4677–4688 (2017). b) Hui, C., Brieger, L., Strohmann, C., Antonchick, A. P., Stereoselective Synthesis of Cyclobutanes by Contraction of Pyrrolidines. *J. Am. Chem. Soc.* **143**, 18864–18870 (2021).

9 Miller, R. D., Golitz, P., Janssen, J., Lemmens, J. Alternative Precursors to 1,4-Acyl Alkyl Biradicals: Cyclic *N*-Acyl-1,1-diazenes., *J. Am. Chem. Soc.* **106**, 7277–7279 (1984).

10 Middleton, W. J., Gale, D. M. Bis(trifluoromethyl)diazomethane. *Org. Synth.* **50**, 6–7 (1970).

11 Huang, P. Q., Lang, Q. W., Hu, X. N. One-Pot Reductive 1,3-Dipolar Cycloaddition of Secondary Amides: A Two-Step Transformation of Primary Amides. *J. Org. Chem.* **81**, 10227−10235 (2016).

12 Zhang, R., Madalengoitia, J. Design, Synthesis and Evaluation of Poly-L-Proline Type-II Peptide Mimics Based on the 3-Azabicyclo[3.1.0]hexane System. *J. Org. Chem.* **64**, 330−331 (1999).

13 Xu, F., Murry, J. A., Simmons, B., Corley, E., K. Fitch, S. Karady, D. Tschaen. Stereocontrolled Synthesis of Trisubstituted Cyclopropanes: Expedient, Atom-Economical, Asymmetric Syntheses of (+)-Bicifadine and DOV21947. *Org. Lett.* **8**, 3885–3888 (2006).

14 Martín, R., Alcón, M., Pericàs, M. A., Riera, A. Ring-Closing Metathesis of Chiral Allylamines. Enantioselective Synthesis of (2S,3R,4S)-3,4-Dihydroxyproline. *J. Org. Chem.* **67**, 6896−6901 (2002).

15 Tayama, E., Kobayashi, Y., Toma, Y. Diaza [1,4] Wittig-type rearrangement of N-allylic-NBoc-hydrazines into γ-amino-N-Boc-enamines. *Chem. Commun.* **52**, 10570–10573 (2016).

16 (a) Nayal, O. S., Thakur, M. S., Bhatt, V., Kumar, M., Kumar, N., Singh, B., Sharma, U. Synthesis of tertiary arylamines: Lewis acid-catalyzed direct reductive N-alkylation of secondary amines with ketones through an alternative pathway. *Chem. Commun.*, **52**, 9648–9651 (2016). (b) Adak, L., Chattopadhyay, K., Ranu, B. C. Palladium Nanoparticle-Catalyzed C–N Bond Formation. A Highly Regio- and Stereoselective Allylic Amination by Allyl Acetates. *J. Org. Chem.* **74**, 3982–3985 (2009).

17 Uenishi, J., Kawahama, R., Izaki, Y., Yonemitsu, O. A Facile Preparation of Geometrically Pure Alkenyl, Alkynyl, and Aryl Conjugated Z-Alkenes: Stereospeci Synthesis of Bombykol. *Tetrahedron*. **56**, 3493–3500 (2000).

18 Liu, X. F., Liu, B. B., Liu, Q., Migratory Hydrogenation of Terminal Alkynes by Base/Cobalt Relay Catalysis. *Angew. Chem. Int. Ed*. **59**, 6750–6755 (2020).
